# DFT/TD-DFT Framework of Mixed-Metal Complexes with Symmetrical and Unsymmetrical Bridging Ligands—Step-By-Step Investigations: Mononuclear, Dinuclear Homometallic, and Heterometallic for Optoelectronic Applications

**DOI:** 10.3390/ma14247783

**Published:** 2021-12-16

**Authors:** Dawid Zych

**Affiliations:** Faculty of Chemistry, University of Opole, Oleska 48, 45-052 Opole, Poland; dawidzych92@gmail.com or dawid.zych@uni.opole.pl; Tel.: +48-77-452-71-17

**Keywords:** pyrene bridging ligands, complexes, osmium, ruthenium, DFT, TD-DFT

## Abstract

Recently, mono- and dinuclear complexes have been in the interest of scientists due to their potential application in optoelectronics. Herein, progressive theoretical investigations starting from mononuclear followed by homo- and heterometallic dinuclear osmium and/or ruthenium complexes with NCN-cyclometalating bridging ligands substituted by one or two kinds of heteroaryl groups (pyrazol-1-yl and 4-(2,2-dimethylpropyloxy)pyrid-2-yl) providing the short/long axial symmetry or asymmetry are presented. Step-by-step information about the particular part that built the mixed-metal complexes is crucial to understanding their behavior and checking the necessity of their eventual studies. Evaluation by using density functional theory (DFT) calculations allowed gaining information about the frontier orbitals, energy gaps, and physical parameters of complexes and their oxidized forms. Through time-dependent density functional theory (TD-DFT), calculations showed the optical properties, with a particular emphasis on the nature of low-energy bands. The presented results are a clear indication for other scientists in the field of chemistry and materials science.

## 1. Introduction

In recent years, mono- and dinuclear complexes containing bridging ligands have been in scientists’ interest [1,2,3,4,5]. The reason for such a comprehensive investigation is the wide possibility of applying those kinds of molecules; they can be used in the area of energy-conversion materials and molecular electronics, but the area of application strongly depends on the applied metal centers [6,7,8,9]. The subgroup of dinuclear complexes of this type is the mixed-metal complexes, which are thus far described only by a few scientific teams. In the reported examples of dinuclear heterometallic complexes, such as the bridging ligands, 2,3,5,6-tetrakis(2-pyridyl)pyrazine (tpp) was used most often. In 1993, Karen J. Brewer et al. initiated investigations in this area by reporting the Ir(III)/Ru(II) mixed-metal complex [(tpy)Ru(tpp)IrCl_3_]^2+^ and the monometallic fragments [Ir(tpp)Cl_3_] and [Ru(tpy)(tpp)]^2+^ [10]. The photochemical, electrochemical, and spectroelectrochemical studies proved the excellent communication between the metals in the case of a dinuclear, heterometallic complex. Later, the same authors presented bimetallic complexes [(tpy)Os(tpp)RuCl_3_]^+^ and [(tpy)Os(tpp)Ru(tpp)]^4+^ and their analogues containing only ruthenium [11], Ru(II)/Rh(III) [12], Ru(II)/Pt(II) [13], and multimetallic complexes [14]. The significant differences in the localization of frontier orbitals were evidence of the influence of the terminal ligands on the properties of the complexes. M. Haga and co-workers reported dinuclear ruthenium and osmium complexes with 1,3,4,6-tetrakis(2-pyridyl)benzene (tpb) as a bridging ligand [15]. Those kinds of complexes exhibited successive one-electron redox processes that correspond to M(II/III) and also a M(III/IV) couple (M = Ru, Os).

Dinuclear homometallic complexes bridging by NCN-cyclometalating pyrene ligands substituted by one kind of a heteroaryl group have been already discussed in terms of the differences between mono- and dinuclear complexes [1,16], symmetrical and unsymmetrical complexes [16], the influence of the various metal centers [3], and terminal ligands on the properties of complexes [17].

Moreover, it was recently presented that in the case of mononuclear osmium complexes with various NCN-cyclometalating pyrene ligands containing 4-(2,2-dimethylpropyloxy)pyrid-2-yl, pyrazol-1-yl, 1-decyl-1*H*-1,2,3-triazol-4-yl, and 2-butyl-*2H*-1,2,3,4-tetrazol-5-yl substituents, significant differences between the properties and behavior of complexes during oxidation were observed [18]. This was caused by the contribution of individual parts of molecules in the creation of frontier orbitals in reference to substituted heteroaryl groups at pyrene.

Furthermore, it was reported that in the case of disubstituted pyrenes at the non-K region, the substitution pattern does not play a role in the properties of the molecules [19]. Whereas studies in the area of tetrasubstituted pyrenes containing two kinds of substituents providing short axial symmetry, long axial symmetry or asymmetry showed significant differences among the examined group compared to analogues with the same four substituents [20,21].

Moreover, the possibility of synthesis of tetrasubstituted pyrenes substituted by two groups providing the short axial symmetry or asymmetry to the structure, i.e., pyrazol-1-yl, 4-(2,2-dimethylpropyloxy)pyrid-2-yl and 1-decyl-1,2,3-triazol-4-yl substituent, has been already presented [20,22]. The photophysical properties of obtained compounds, such as good solubility and thermal stability, allow them to be used as the NCN-cyclometalating ligands in the synthesis of target complexes.

Taking into account the fact that metal–metal interactions strongly depend, besides on the metal ion, on the nature of the bridging and terminal ligands [23], herein based on the previous research and already developed parameters of the theoretical calculations (DFT method), which correlate well with experimental data, the theoretical investigations of mono- and dinuclear homo- and heterometallic complexes containing osmium and/or ruthenium metals bridging by symmetrical or asymmetrical double NCN-cyclometalating pyrene ligands substituted by pyrazol-1-yl and 4-(2,2-dimethylpropyloxy)pyrid-2-yl group are presented.

## 2. Computational Methods

The DFT and TD-DFT calculations were performed with the B3LYP [24] exchange-correlation functional implemented in the Gaussian 09 program [25]. The Def2-TZVP basis set was used for osmium and ruthenium, and 6-31G(d,p) was employed for other atoms. The calculations were performed with acetonitrile as the solvent in the polarizable continuum model (PCM) [26]. The frequency calculations confirmed the energy minimum of the stationary state of all optimized geometries. All orbitals were computed at an isovalue of 0.02 e/bohr^3^ (spin-density—the isosurface contour value 0.002 e/bohr^3^). The contribution of each moiety in the creation of the selected orbitals was calculated by using Chemissian software (Version 4.60, Skripnikov Leonid 2005–2018). (https://www.chemissian.com/). The theoretical spectra and the extinction coefficients were based on Gaussian convolution by the GaussSum software (Version 3.0,Dublin, Ireland) [27]. The full-width half-maximum (FWHM) value used for the simulated spectra was 2000 cm^−1^. Cartesian coordinates of DFT-optimized structure of all complexes with values of charge and multiplicity are presented in Supplementary Materials.

## 3. Results and Discussion

**Figure 1:**
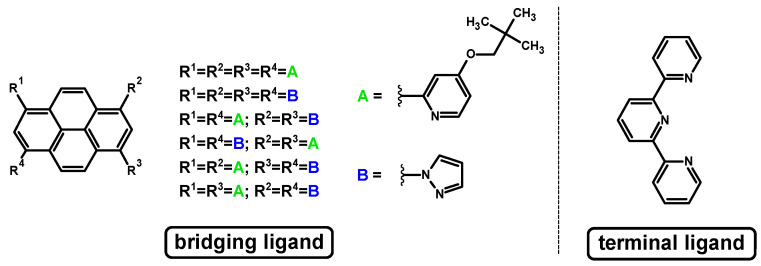
The structures of bridging ligands and terminal ligand.

As the NCN-cyclometalating ligands, six pyrene derivatives substituted at positions 1, 3, 6, and 8 by one or two kinds of substituents (pyrazol-1-yl and 4-(2,2-dimethylpropyloxy)pyrid-2-yl) providing symmetry or asymmetry to the whole structure were used. The synthesis way of these ligands and their nature has already been demonstrated. In the case of terminal ligand, 2,2′:6′,2′′-terpyridine was applied (Figure 1).

### 3.1. Mononuclear Osmium and Ruthenium Complexes

**Figure 2:**
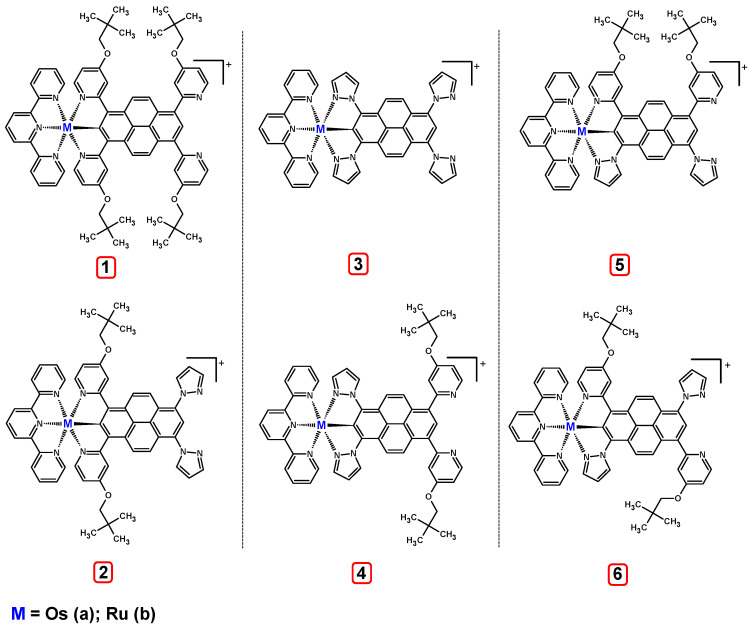
The structures of the mononuclear osmium **1a**-**6a** and ruthenium **1b**-**6b** complexes.

To check the influence of the NCN-cyclometalating pyrene ligands and coordinated metal on the properties of the target dinuclear complexes, first, the mononuclear complexes with osmium Os(I) **1a**-**6a** and ruthenium Ru(I) **1b**-**6b** were designed, as presented in Figure 2.

**Figure 3:**
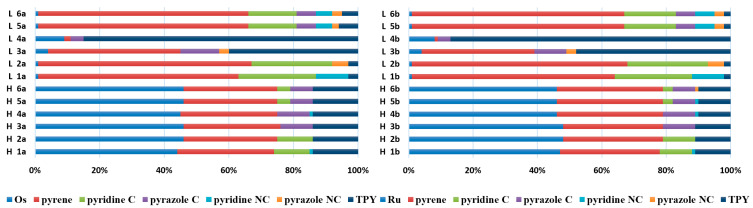
The contribution of the individual part of molecules (C = coordinating, NC = not coordinating) in the creation of frontier orbitals for molecules **1a**-**6a** and **1b**-**6b**.

The optimized structures of molecules **1a**-**6a** and **1b**-**6b** are presented in Table 1. The contribution of individual parts of molecules in the creation of frontier orbitals is presented in Figure 3.

The contribution of ruthenium (46–48%) in creating the highest-occupied molecular orbitals of molecules **1b**-**6b** is slightly higher than the contribution of osmium (44–46%) in the analogue molecules **1a**-**6a**. The sum of contribution in the creation of HOMOs by coordinating heteroaryl groups equals ≈11% for **1a**-**6a** and ≈10% for **1b**-**6b**. When two kinds of heteroaryl groups participate in the coordination of metal (**5a** and **6a**), the contribution of pyrazolyl groups is around two times higher than pyridyl substituents. The localization of the lowest unoccupied molecular orbitals for molecules **1a/b**, **2a/b**, **5a/b**, and **6a/b** does not show any significant differences; the contribution of pyrene is the highest, whereas LUMOs in **3a/b** and **4a/b**, where the coordination proceeds by pyrazolyl groups, are majorly localized on terpyridine. Moreover, for molecules **4a/b** with pyrene substituted by two kinds of groups providing long axial symmetry, the contribution of terminal ligand (TPY) is very high, i.e., 85% for **4a** and 87% for **4b**. The values of energy of HOMOs, LUMOs, energy gaps, and bond lengths M(II)-C for molecules **1a**-**6a** and **1b**-**6b** are listed in Table 2.

**Figure 4:**
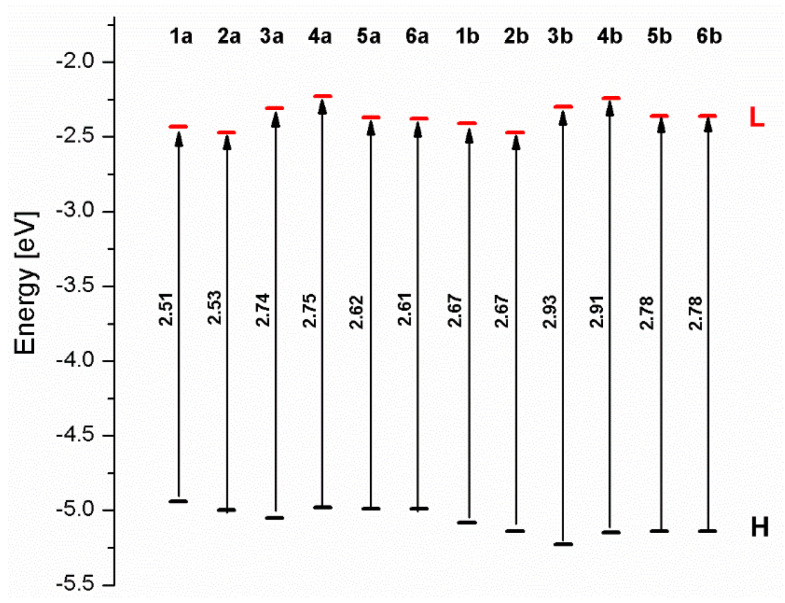
The energies of frontier orbitals with the values of energy gaps for molecules **1a**-**6a** and **1b**-**6b**.

The energy gaps for mononuclear osmium complexes **1a**-**6a** are lower than the values for corresponding ruthenium molecules **1b**-**6b** (Figure 4). The order of the increasing values of the energy gaps does not follow the same trend. In the case of osmium complexes, the lowest ΔE was achieved by complex **1a** followed by **2a < 6a < 5a < 3a < 4a,** whereas the change of energy gaps’ values for ruthenium complexes followed the trend **1b = 2b < 5b = 6b < 4b < 3b**.

The lengths of the bond metal–carbon for osmium complexes **1a**-**6a** are higher than for ruthenium complexes **1b**-**6b**. Among the groups, the lengths differ from each other; the longest was achieved for complexes **4a** (2.000 Å) and **4b** (1.981 Å), respectively, where the coordination proceeds by pyrazolyl groups, whereas the shortest was for **2a** (1.992 Å) and **2b** (1.972 Å) with coordination by pyridyl groups. It is worth emphasizing that NCN-cyclometalating pyrene ligands in the case of **2a**, **2b**, **4a**, and **4b** are substituted by two kinds of groups providing long axial symmetry.

**Figure 5:**
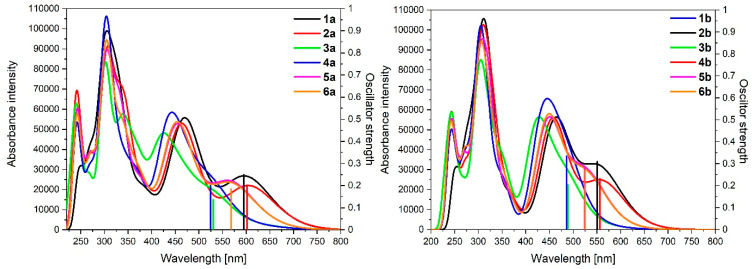
Theoretical absorption spectra of complexes **1a**-**6a** and **1b**-**6b**.

Absorption spectra of **1a**-**6a** and **1b**-**6b** were calculated by time-dependent density functional calculations (TD-DFT) presented in Figure 5.

The shape of the absorption spectra of complexes **1a**-**6a** and **1b**-**6b** is similar but with a noticeable difference in the area of low-energy bands 525–800 nm for **1a**-**6a** and 500–700 nm for **1b**-**6b**. It is caused by the character of the transition, which creates the lowest-energy bands dominated by transition H-1**→**LUMO (Table 3).

To better understand the nature of the lowest energy bands for complexes **1a**-**6a** and **1b**-**6b**, NTO analysis for complexes containing osmium is presented in Table 4. Analysis of complexes **1a**-**6a** showed that the lowest energy transitions were observed for complexes **1a** and **2a**, where the coordination proceeds by pyridyl substituents and can be described as the excited state S_4_. In the case of molecules **3a** and **4a**, where pyrazolyl groups coordinate the metal, the excited state S_6_ corresponds to the transition with the highest energy among the lowest-energy transitions. Excited-state S_5_ corresponds to the low-energy bands for compounds **5a**-**6a**. In contrast to molecules **1a**-**4a**, the significant contribution of terminal ligand (TPY) in the creation of LUTO for **5a** and **6a** was observed. Moreover, the behavior of the complexes **5a** and **6a** is similar; there are no meaningful differences in relation to pyrene ligand substituted in a long-axis symmetrical way or asymmetrical when the coordination proceeds by two various heteroaryl groups. The natural transition orbitals with pairs of holes–electrons, and the contribution of the particular part in their creation for ruthenium complexes **1b**-**6b** are listed in Table 5. The character of the lowest-energy transitions in the case of molecules **1b**-**6b** is similar to **1a**-**6a,** with a slightly higher contribution of the ruthenium than osmium. All the lowest-energy transitions can be assigned as metal-to-ligand-charge-transfer (MLCT).

The affinity of metallic centers, hence the whole molecule, for change can be identified by calculation of the spin-density distribution of the lowest energy triplet state (multiplicity = 3) with the values of spin distribution on metals (Mulliken population). Data obtained for molecules **1a**-**6a** and **1b**-**6b** are listed in Table 6. The tendency of changes does not follow the same trend for the osmium complexes **1a**-**6a** as for the ruthenium complexes **1b**-**6b**. In the case of the first group of molecules, the spin-density distribution on metal is the highest for **5a**, whereas for the second group, **6b**. The significant differences between **4a** vs. **4b** and **5a** vs. **5b** were noticed; the difference between the analogues molecules, which differ from each other only by coordinated metal, was even 20 times. In all cases besides compound **5b**, the spin density on metal is higher for ruthenium than for the corresponding osmium complex.

The higher spin distribution on the metal of complexes may cause the most significant differences before and after oxidation. The structures of oxidized Os(II) **1aox**-**6aox** and Ru(II) **1box**-**6box** complexes were optimized; the contours of selected α and β-spin orbitals (HOSO and LUSO) are presented in Table S1 in Supplementary Materials. Energies of α and β-spin orbitals (HOSO and LUSO) and bonds lengths of oxidized complexes are listed in Table 7. The bond M(III)-C lengths for oxidized complexes are lower than **1a**-**6a** and **1b**-**6b**. The most significant differences were observed for **4a**/**4aox** and **4b**/**4box** with the change 0.030 Å and 0.035 Å, respectively. The slightest change was observed for the shortest bonds **2a**/**2aox** and **2b**/**2box**.

**Figure 6:**
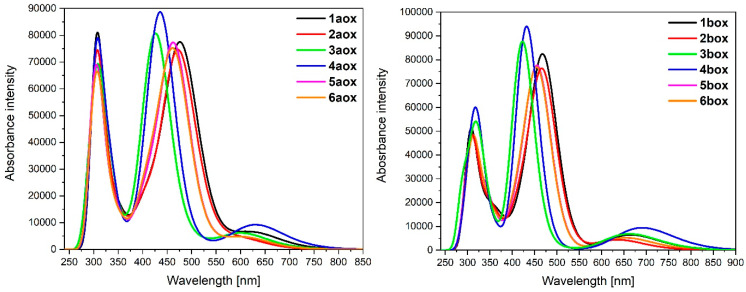
Theoretical absorption spectra of complexes **1aox**-**6aox** and **1box**-**6box**.

The calculated absorption spectra of oxidized complexes **1aox**-**6aox** and **1box**-**6box** are presented in Figure 6, and the calculated lowest energy transitions are listed in Table 8.

The intensity of the low-energy bands of oxidized complexes **1aox**-**6aox** and **1box**-**6box** decreased compared to non-oxidized complexes **1a**-**6a** and **1b**-**6b**, but significant differences in the behavior of respective complexes were observed. The greatest change of oscillator strength from 0.3345 to 0.0540 (0.2805) was observed for **4box**; the differences among osmium complexes **1a**-**6a** vs. **1aox**-**6aox** are lower in comparison to ruthenium analogues **1b**-**6b** vs. **1box**-**6box**. The most intense band for oxidized complexes was observed for **4aox** and **1box**, whereas it was the least intense for **2aox** and **4box**; this can be caused by the character of oxidation, which can be described as metal-dominated oxidation [18].

It can be noticed that β-spin orbitals take part in the creation of the low-energy bands, but **1box** also occupied α-spin orbital. All calculated low-energy bands of **1aox**-**6aox** and **1box**-**6box** are red-shifted up to 182 nm for **4b**/**4box**; among osmium complexes, the most significant shift was observed for **4a**/**4aox**.

### 3.2. Dinuclear Osmium and Ruthenium Complexes

**Figure 7:**
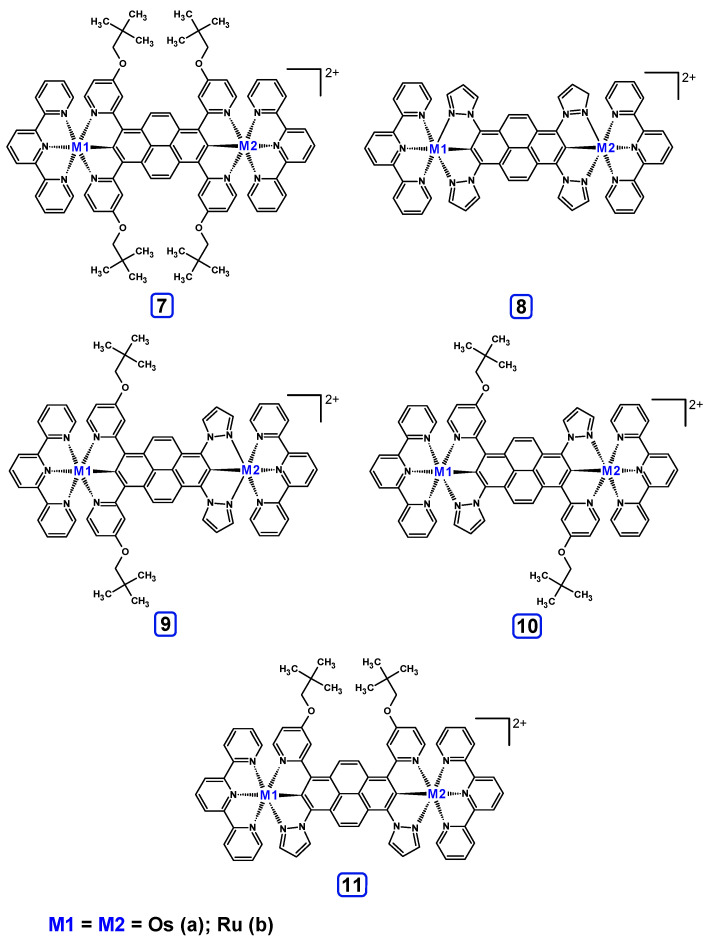
The structures of the dinuclear osmium **7a**-**11a** and ruthenium **7b**-**11b** complexes.

Optimized structures of mononuclear Os(II) **1a**-**6a** and Ru(II) **1b**-**6b** complexes were used in the next part of the investigations dedicated to dinuclear homometallic complexes containing osmium or ruthenium metals bridging by the same double NCN-cyclometalating pyrene ligands; the structures are presented in Figure 7.

**Figure 8:**
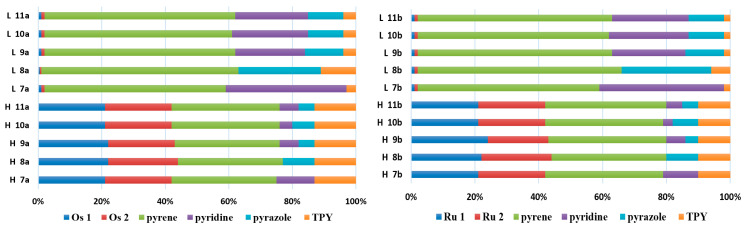
The contribution of the individual part of molecules in the creation of frontier orbitals for molecules **7a**-**11a** and **7b**-**11b**.

The optimized structures of molecules **7a**-**11a** and **7b**-**11b** are presented in Table 9. The contribution of individual parts of molecules in the creation of frontier orbitals is presented in Figure 8.

The sum of the percentage contribution of ruthenium and osmium in the creation of HOMOs of **7a**-**11a** and **7b**-**11b** is the same (42–44%). Only in the case of complexes **9a** and **9b**, the contribution of the particular metal M1 and M2 differs from each other, with the dominance of metal where the coordination proceeds from one side by a pyridyl group and from the second side by a pyrazolyl substituent. Moreover, the sum of the substituents’ contribution in HOMOs is in the range of 10–12%. In the case of the lowest unoccupied molecular orbitals, they are delocalized on the same part of complexes with the exception of **8a** and **8b**, where the contribution of the terpyridine ligand is significantly higher (11% for **8a** and 6% for **8b**) than for the rest of the osmium (3–4%) and ruthenium (2%) complexes. The energy values of HOMOs, LUMOs, energy gaps, and bond lengths M(II)-C for molecules **7a**-**11a** and **7b**-**11b** are listed in Table 10.

**Figure 9:**
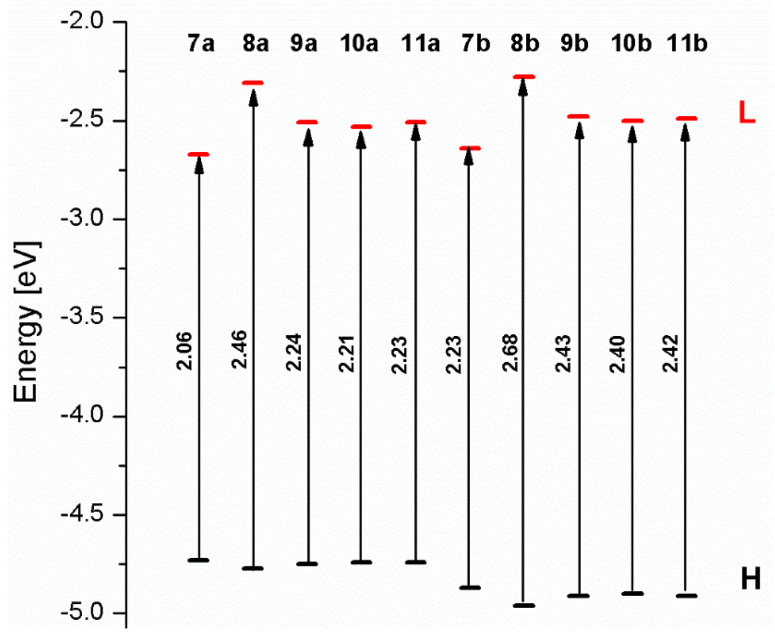
The energies of frontier orbitals with the values of energy gaps for molecules **7a**-**11a** and **7b**-**11b**.

The energy gaps for osmium complexes **7a**-**11a** are lower than the values for corresponding ruthenium molecules **7b**-**11b** (Figure 9). The order of the increasing values of the energy gaps follows the same trend; among two groups, the lowest ΔE was achieved by complex **7a** and **7b**, followed by **10a/10b < 11a/11b < 9a/9b < 8a/8b**.

The lengths of the bond of metal–carbon for osmium complexes **7a**-**11a** are higher than for ruthenium complexes **7b**-**11b**. The shortest was achieved for complexes **7a** (1.995 Å) and **7b** (1.975 Å), where the coordination proceeds by pyridyl groups, whereas the longest for **9a** (2.002 Å) and **9b** (1.983 Å) from the side where metal is coordinated by pyrazolyl groups, also compared to dinuclear homometallic complex with ligands containing only pyrazolyl groups **8a/b**, is a higher value. The same tendency was also observed for mononuclear complexes **1a**-**6a** and **1b**-**6b**.

**Figure 10:**
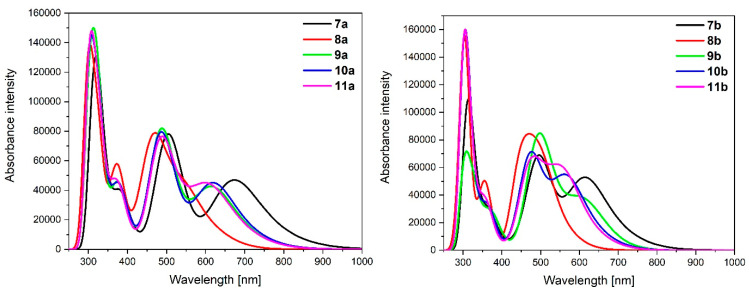
Normalized theoretical absorption spectra of complexes **7a**-**11a** and **7b**-**11b**.

TD-DFT calculated absorption spectra of **7a**-**11a** and **7b**-**11b** are presented in Figure 10.

Electronic absorptions in the UV region are associated with intraligand (IL) and ligand-to-ligand-charge-transfer (LLCT) π→π* transitions from the bridging and terpyridine ligands, similar to that already discussed in the literature [1,3]. The significant difference between the absorption of osmium **7a**-**11a** and ruthenium **7b**-**11b** complexes in the UV region can be observed in the weaker intensities of particular bands caused by the poorer orbital overlap of ruthenium. Furthermore, osmium complexes **7a**-**11a** absorb light up to *λ* = 1000 nm, whereas the absorption range for ruthenium analogues **7b**-**11b** is narrower, up to *λ* = 800–850 nm. The bands with the lowest energy can be mainly assigned to HOMO→LUMO transitions; thereby, metal-to-ligand charge-transfer (MLCT) transitions from the metal centers to the bridging pyrene ligands (Table 11).

The most intense MCLT transitions were observed for **7a** (*λ* = 771.43 nm, f = 0.1088) and **7b** (*λ* = 702.22 nm, f = 0.1203), which are red-shifted in comparison to the rest molecules; the lowest transitions were calculated for **8a** (*λ* = 620.45 nm, f = 0.0652) and **9b** (*λ* = 653.75.43 nm, f = 0.0574). The similar broad MLCT transition of ruthenium and osmium complexes as those for discussed molecules **7a**-**11a** and **7b**-**11b** with other bridging and terminal ligands have been already presented [3,28]. Hence, there is no impact of the various methods of substitution of pyrene by two kinds of heteroaryl groups on the optical properties of non-oxidized complexes.

The spin-density distribution of the lowest-energy triplet state with the values of spin distribution on metals (Mulliken population) for molecules **7a**-**11a** and **7b**-**11b** are presented in Table 12.

Complexes containing NCN-cyclometalating substituted by heteroaryl groups, providing a center of symmetry **7a**, **8a**, **10a**, **7b**, **8b**, **10b** have the same spin-density distribution of two metal centers. In contrast, substitution pattern with two kinds of heteroaryl substituents influences the various spin-density distribution; the most significant difference is observed for the derivatives **9a** and **9b** with a long axial, symmetric bridging ligand. Higher values are achieved from the side where the coordination proceeds by pyridyl groups.

The structures of oxidized Os(II)-Os(III) **7aox**-**11aox** and Ru(II)-Ru(III) **7box**-**11box** complexes were optimized; the contours of selected α and β-spin orbitals (β-HOSO and β-LUSO) are presented in Table 13, whereas energies of α-spin and β-spin orbitals (α-HOSO, β-HOSO, α-LUSO, β-LUSO) and bonds lengths of oxidized complexes are listed in Table 14.

Taking into account the contours of β-HOSO and β-LUSO for **9aox** and **9box**, the already observed blue-shifted low-energy band can also be caused by the significant differences of the contribution of particular coordinated metals; β-HOSO is mainly created by Os/Ru coordinated by pyrazolyl groups, whereas β-LUSO by Os/Ru is coordinated by pyridyl substituents. In the case of other molecules, the distribution of the frontier β-spin orbitals is symmetric. The M-C bond lengths for oxidized complexes are lower than for **7a**-**11a** and **7b**-**11b**, with differences up to 0.038 Å.

**Figure 11:**
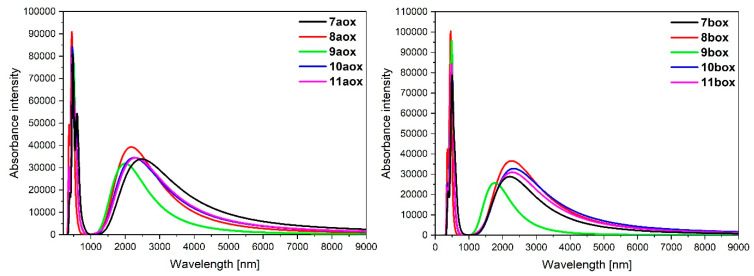
Theoretical absorption spectra of complexes **7aox**-**11aox** and **7box**-**11box**.

The calculated absorption spectra of oxidized complexes **7aox**-**11aox** and **7box**-**11box** are presented in Figure 11, and the calculated lowest-energy transitions are listed in Table 15.

The intensity of the low-energy bands of oxidized complexes **7aox**-**11aox** and **7box**-**11box** significantly increased compared to non-oxidized complexes **7a**-**11a** and **7b**-**11b**. The appeared bands are strongly red-shifted in the NIR region. The intensity of the band for osmium **7aox**-**11aox** is slightly higher than ruthenium complexes **7box**-**11box**. The most intense band was observed for **7aox** (*λ* = 2479.19 nm, f = 0.4520) and its analogue with ruthenium **7box**. Furthermore, the low-energy band for molecules **9aox** (*λ* = 1988.84 nm, f = 0.4308) and **9box** (*λ* = 1765.15 nm, f = 0.3460) is shifted to the shorter wavelength; it can be caused by the character of oxidation and significant differences between spin-density distributions on particular metals in reference to coordinating heteroaryls (Table 12). Intense bands for complexes **10aox** (*λ* = 2444.48 nm, f = 0.2490; *λ* = 2082.02 nm, f = 0.2460), **7box** (*λ* = 2265.79 nm, f = 0.1105; *λ* = 2189.37 nm, f = 0.2830), and **10box** (*λ* = 2431.54 nm, f = 0.3392; *λ* = 2069.85 nm, f = 0.1275) are built by two bands. Moreover, it can be noticed that in the creation of the low-energy band, only β-spin orbitals are involved; major transitions can be described as HOSO(β)**→**LUSO(β).

### 3.3. Dinuclear Mixed-Metal Osmium/Ruthenium Complexes

**Figure 12:**
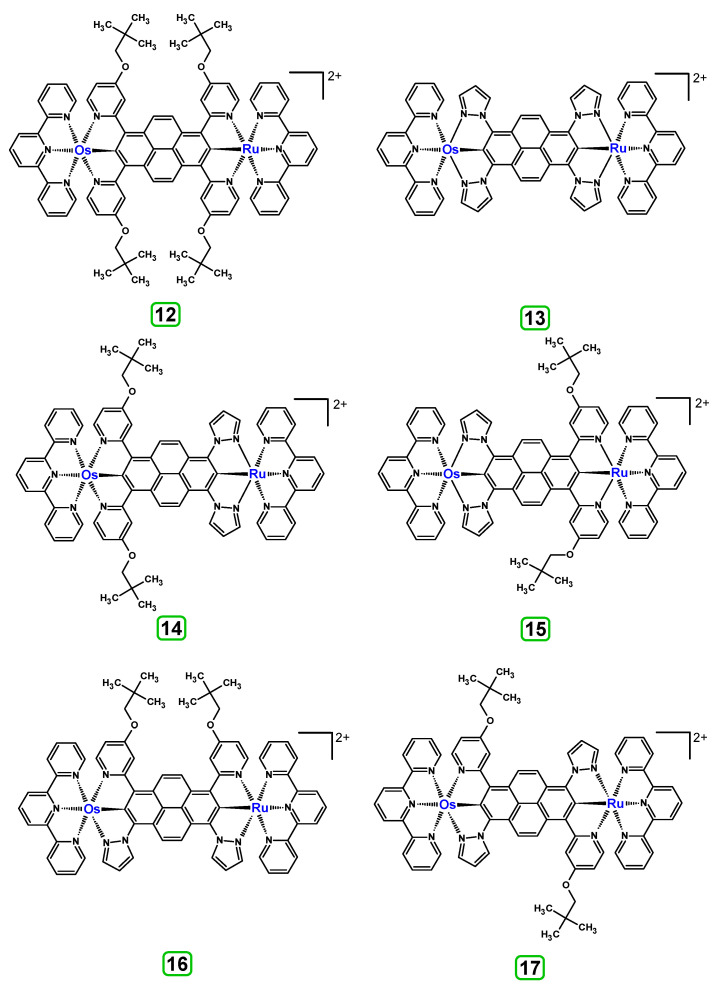
The structures of the mixed-valance Os/Ru complexes **12**-**17**.

Based on the optimized structures, a comprehensive evaluation of the mononuclear and dinuclear homometallic osmium and ruthenium complexes bridged by double NCN-cyclometalating pyrene ligands allowed us to conduct the calculations of dinuclear heterometallic complexes Os(II)-Ru(II) **12**-**17**, which is presented in Figure 12.

**Figure 13:**
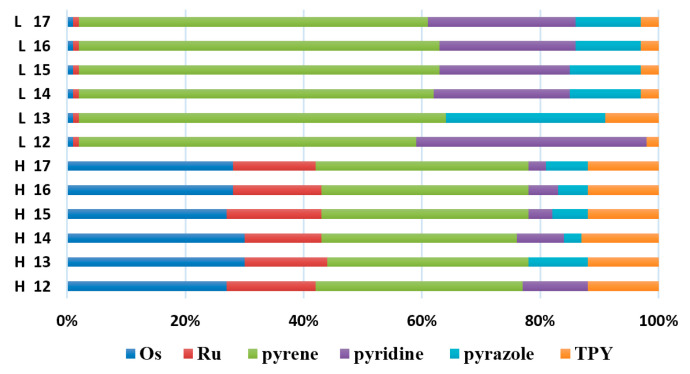
The contribution of the individual part of molecules in the creation of frontier orbitals for molecules **12**-**17**.

The optimized structures of molecules **12**-**17** are presented in Table 16. The contribution of individual parts of molecules in the creation of frontier orbitals is presented in Figure 13.

The sum of the percentage contribution of ruthenium and osmium in the creation of HOMOs of **12**-**17** is in the range of 42–44%, with significant dominance of osmium, up to 30% for molecules **13** and **14**. In contrast, ruthenium takes part in the creation of HOMOs up to 16% for complex **15**. Generally, the contribution of osmium is one time higher than that of ruthenium. There are no differences in the impact of bridging and terminal ligands in creating the highest occupied molecular orbitals. Furthermore, the lowest unoccupied molecular orbitals are created by 2% coordinated metals (1% Os and 1% Ru); major delocalization of LUMO is on pyrene up to 62% for **13**. The localization of the frontier orbitals on only bridging ligands is already published [20,22]. The energy values of HOMOs, LUMOs, energy gaps, and bond lengths M(II)-C for molecules **12**-**17** are listed in Table 17.

**Figure 14:**
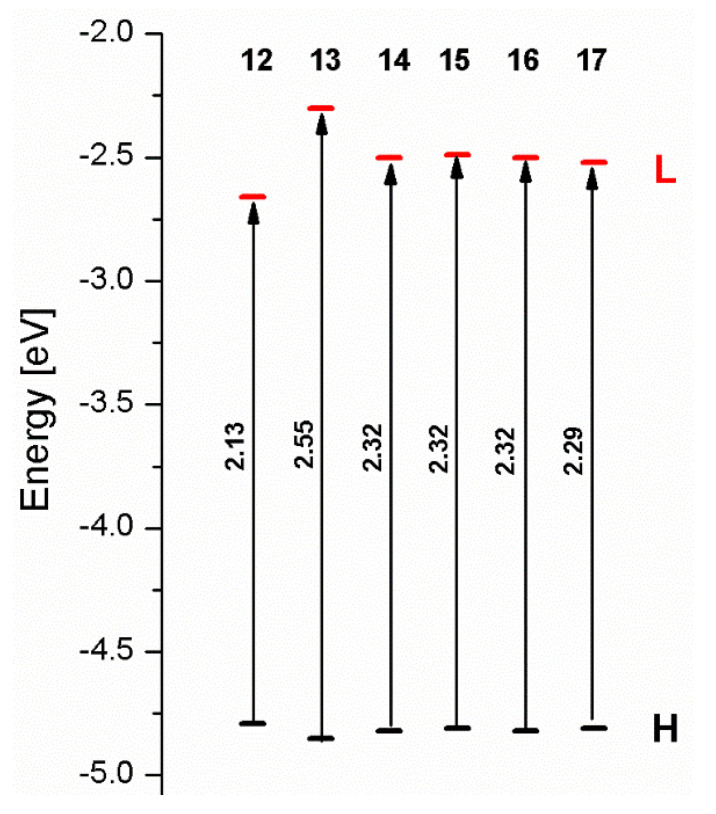
The energies of frontier orbitals with the values of energy gaps for molecules **12**-**17**.

The values of energy gaps for complexes **12**-**17** are lower than the values for corresponding ruthenium molecules **7b**-**11b** but higher than for osmium complexes **7a**-**11a** (Figure 14). Among the dinuclear heterometallic complexes, the lowest ΔE was achieved by complex **12,** followed by **17**; the same values of energy gaps are present for molecules **14**, **15**, and **16**, and the highest one was for **13**. Interestingly, substituted pyrene ligands containing a symmetric center decrease the value of the energy band; the same phenomenon was observed for dinuclear homometallic complexes **10b** and **10a**, containing the same NCN-cyclometalating ligand. The lengths and the tendency of change of the bond of metal–carbon for **12**-**17** are the same as for dinuclear homometallic complexes **7a**-**11a** and **7b**-**11b**. The longest bonds were achieved when the coordination proceeds by pyrazolyl groups, when the NCN-cyclometalating ligands contain two kinds of heteroaryl groups **14** (Ru(II)-C 1.983 Å) and **15** (Os(II)-C 2.002 Å); in the case of compound **13**, which contains only pyrazolyl groups, the bonds’ lengths are slightly shorter (Ru(II)-C 1.982 Å and Os(II)-C 2.001 Å).

T. Nagashima et al. reported that there are two electronic coupling mechanisms: electron transfer and hole transfer superexchange [15]. The mixing between metal dπ(M1 and M2) and bridging ligand π* is ascendant for the Os 5dπ level in comparison to the Ru 4dπ level; on the other hand, the mixing between metal dπ(M1 and M2) and bridging ligand π is dominant for the Ru 4dπ level. The HOMO and HOMO-1 of **12**-**17** are mainly localized on osmium dπ orbitals and bridging ligand π orbitals. In the case of HOMO, the contribution of the ruthenium dπ orbitals is more significant, especially for **15**, but still definitely lower than osmium. It can suggest that the metal−metal interaction takes place through a hole-transfer mechanism. The LUMOs of **12**-**17** are composed of the bridging ligand π* orbitals. In the case of **13**, the LUMO is localized significantly on terminal ligands π* orbitals—terpyridine. The LUMO+1 of **12**-**14** and **16**-**17** are composed of the ruthenium dπ orbitals with terminal ligand π* orbitals. Dinuclear complex **15** differ significantly; the LUMO+1 is composed of the osmium dπ orbitals with terpyridine π* orbitals. The ruthenium 4dπ orbitals interacted strongly with the terpyridine π* orbitals (except for **15**), whereas the osmium 5dπ orbitals strongly mixed with bridging ligand orbitals, determining the degree of strength of the metal−metal interaction. This suggests that the substitution pattern of the bridging ligand and the coordinated metals have a significant impact on the properties of the dinuclear heterometallic complexes.

**Figure 15:**
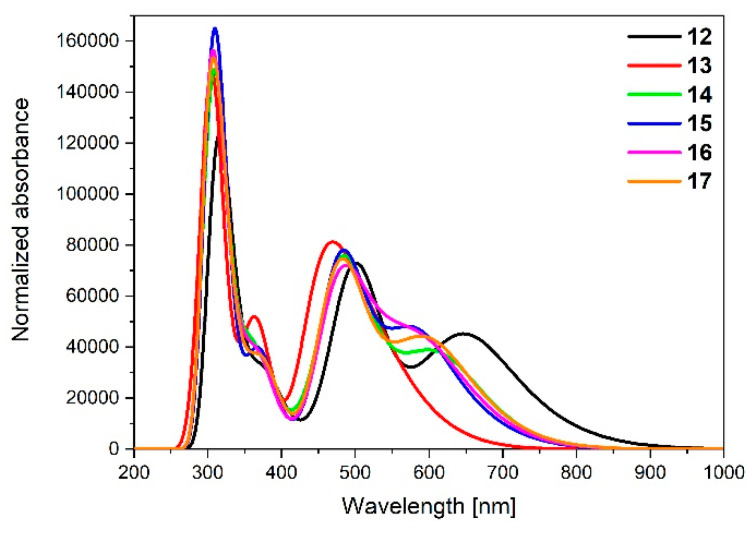
Theoretical absorption spectra of complexes **12**-**17**.

TD-DFT calculated absorption spectra of **12**-**17** are presented in Figure 15.

Calculated absorptions spectra for **12**-**17** have the same shape as the spectra calculated for dinuclear homometallic complexes **7a**-**11a** and **7b**-**11b**. The bands in the UV region can also be associated with IL and LLCT transitions. The intensities and the absorption range (up to *λ* = 1000 nm for **12** and up to *λ* = 800 nm for **13**-**17**) of the dinuclear heterometallic complexes **12**-**17** follow the behavior of osmium complexes **7a**-**11a**, which is in accordance with the higher contribution of osmium in the creation of frontier orbitals in comparison to ruthenium.

The bands with the lowest energy can be assigned mainly as HOMO→LUMO transitions—MLTC, the same as for **7a**-**11a** and **7b**-**11b** (Table 18). The most intense one was calculated for complex **12** (*λ* = 739.63 nm, f = 0.1111); the lowest was for **13** (*λ* = 588.50 nm, f = 0.0647).

The spin-density distribution of the lowest energy triplet state with the values of spin distribution on metals (Mulliken population) for molecules **12**-**17** are presented in Table 19.

Higher spin-density distribution of the lowest-energy triplet state takes place on osmium metals—up to 10 times higher in the case of molecule **14**. Complexes containing a bridging ligand derivative with the same four heteroaryl groups **12** and **13** cause the distribution difference between Os and Ru up to 6 times. The same differences were observed for the analogue complexes with the same ligands **9a** and **9b**.

The structures of oxidized complexes **12ox**-**17ox** were optimized; the contours of the highest occupied α and β-spin orbitals (α-HOSO and β-HOSO) and lowest unoccupied α and β-spin orbitals (α-LUSO and β-LUSO) are presented in Table 20. Energies of α-spin and β-spin orbitals and bonds lengths of oxidized complexes are listed in Table 21.

Among the oxidized complexes **12ox**-**17ox**, the energy gap value in the case of β-spin orbitals for **15ox** is the lowest (1.11 eV). The bond M-C lengths for oxidized complexes **12ox**-**17ox** are shorter than for **12**-**17**, with the highest difference for **15ox** vs. **15**, for osmium 0.037 Å and for ruthenium 0.023 Å.

**Figure 16:**
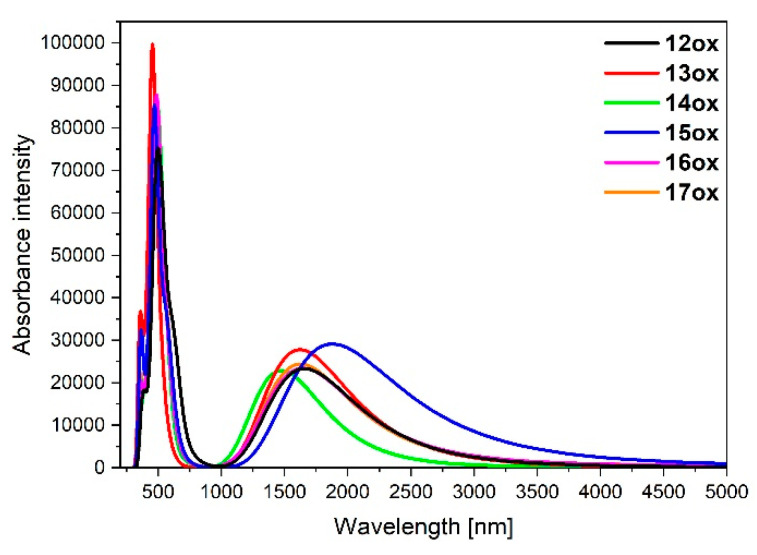
Normalized theoretical absorption spectra of complexes **12ox**-**17ox**.

The calculated absorption spectra of oxidized complexes **12ox**-**17ox** are presented in Figure 16, and the calculated lowest-energy transitions are listed in Table 22.

Similar to **7a**-**11a** and **7b**-**11b**, the intensity of the low-energy bands strongly red-shifted in the NIR region of oxidized complexes **12ox**-**17ox** significantly increased compared to non-oxidized complexes **12**-**17**. In the case of **12ox**-**17ox**, their intensities are lower than for analogues **7aox**-**11aox** and **7box**-**11box**. The most intense and red-shifted band was observed for **15ox** built by two components (*λ* = 1876.27.19 nm, f = 0.1030; *λ* = 1873.72 nm, f = 0.2977), where the coordination proceeds by pyrazolyl for Os and pyridyl for Ru, which is in accordance with the tendency observed among studied compounds **12**-**17** presented in Table 16. It is opposite to dinuclear homometallic complexes **9aox** (*λ* = 1988.84 nm, f = 0.4308) and **9box** (*λ* = 1765.15 nm, f = 0.3460) with the same bridging ligand, where the low-energy bands were the least intense and shifted to the shorter wavelength among all oxidized complexes **7aox**-**11aox** and **7box**-**11box**. Furthermore, only β-spin orbitals, the same as for **7aox**-**11aox** and **7box**-**11box**, are involved in the creation of the low-energy bands with major transitions HOSO(β)**→**LUSO(β).

## 4. Conclusions

Step-by-step investigations starting from mononuclear followed by homo- and heterometallic dinuclear osmium and/or ruthenium complexes with NCN-cyclometalating bridging ligands substituted by one or two kinds of heteroaryl groups (pyrazol-1-yl and 4-(2,2-dimethylpropyloxy)pyrid-2-yl) in a method providing the short/long axial symmetry or asymmetry has shown significant differences between and within the studied groups. The thorough knowledge of mononuclear and homometallic dinuclear osmium and/or ruthenium complexes was crucial to understanding the properties of heterometallic dinuclear Os/Ru coordination compounds. In the case of mononuclear complexes, when two kinds of heteroaryl groups participate in the coordination of metal, the contribution of pyrazolyl groups is around two times higher than pyridyl substituents. When these groups are substituted in a method providing long axial symmetry, the contribution of terminal ligand in the creation of LUMO is very high. The shape of the absorption spectra of mononuclear complexes is similar in the UV region, with a noticeable difference in the area of low-energy bands caused by the character of the transition, which creates the lowest-energy bands that can be assigned as metal-to-ligand-charge-transfer (MLCT). In the case of homometallic dinuclear osmium or ruthenium complexes, the contribution of the particular metal differs from each other, with the dominance of metal where the coordination proceeds from the one side by a pyridyl group and from the second side by a pyrazolyl substituent. Electronic absorptions in the UV region are associated with intraligand (IL) and ligand-to-ligand-charge-transfer (LLCT) transitions from the bridging and terminal ligands. Osmium complexes absorb light in a wider range in comparison to ruthenium analogues. The bands with the lowest energy can be assigned mainly as HOMO→LUMO, thereby MLCT transitions. There is no impact of the various method of substitution of pyrene by two kinds of heteroaryl groups on the optical properties of non-oxidized complexes. In contrast, in the case of oxidized complexes, the intensity of the low-energy bands significantly increased; bands were strongly red-shifted in the NIR region. For heterometallic dinuclear osmium and ruthenium complexes, the significant dominance of osmium in contrast to ruthenium in the creation of HOMOs was observed. The values of energy gaps for heterometallic dinuclear complexes are lower than the values for corresponding ruthenium molecules and higher than for osmium analogues. Complexes containing NCN-cyclometalating pyrene ligands substituted providing the symmetric center decrease the value of energy band. The calculated absorptions spectra have the same shape as the spectra calculated for dinuclear homometallic complexes and follow the behavior of osmium complexes, which is in accordance with the higher contribution of osmium in the creation of frontier orbitals in comparison to ruthenium. Definitely higher (up to 10 times) spin-density distribution of the lowest-energy triplet state takes place on osmium metals. The low-energy bands of oxidized complexes are strongly red-shifted in the NIR region, with higher intensities than non-oxidized but lower than homometallic analogues. The most intense and red-shifted band was observed where the coordination proceeds by pyrazolyl for Os and pyridyl for Ru. This is opposite to dinuclear homometallic complexes with the same bridging ligand, where the low-energy bands were the least intense and shifted to the shorter wavelength among all oxidized complexes. The presented results showed that the properties of studied compounds depend on the nature of the metals and the nature of bridging ligands. They demonstrate the necessity of synthesis and experimental studies, especially dinuclear heterometallic complexes with NCN-cyclometalating ligands, which are substituted by two kinds of the heteroaryl groups symmetrically with respect to the center of symmetry.

## Figures and Tables

**Table 1:** The optimized structures with HOMOs and LUMOs contours for molecules **1a**-**6a** and **1b**-**6b**.

	HOMO	LUMO		HOMO	LUMO
**1a**	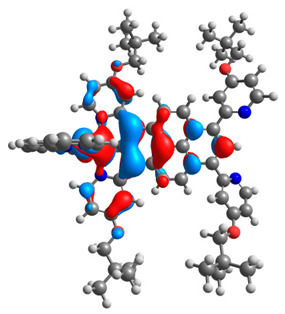	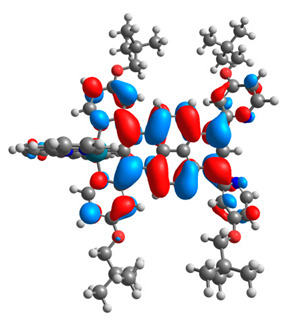	**1b**	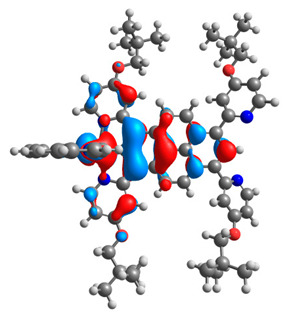	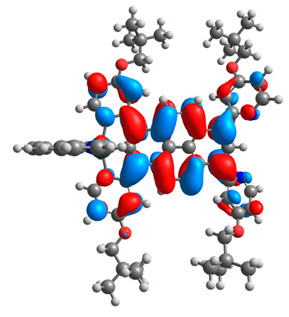
**2a**	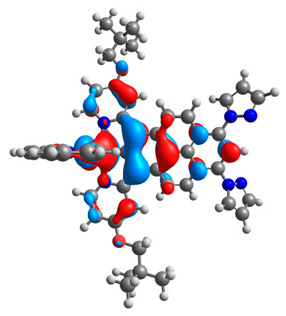	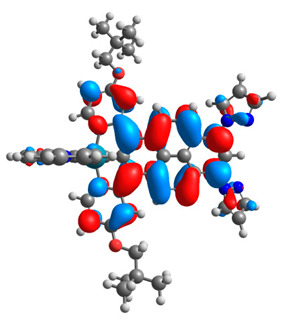	**2b**	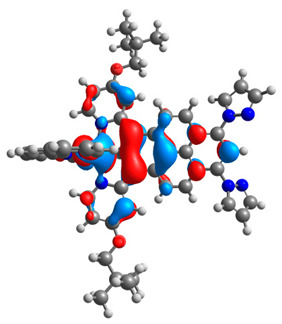	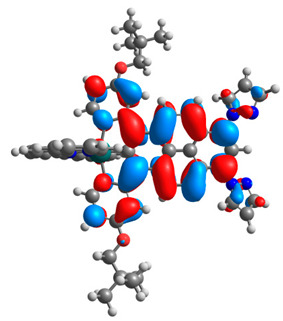
**3a**	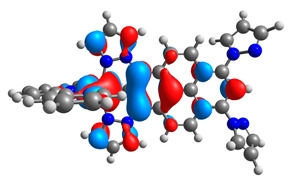	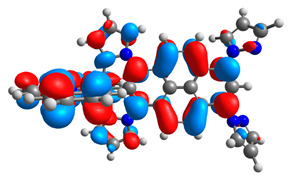	**3b**	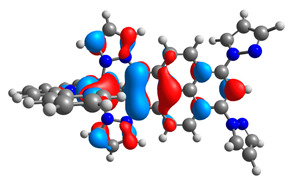	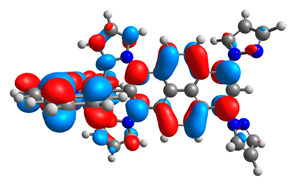
**4a**	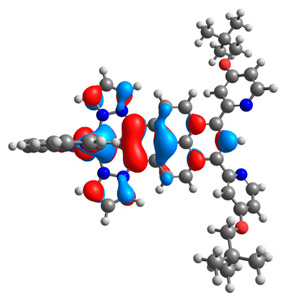	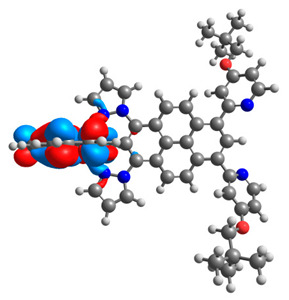	**4b**	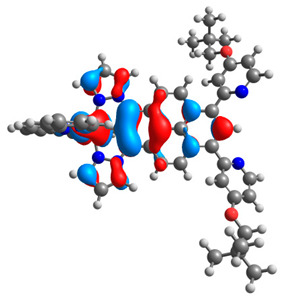	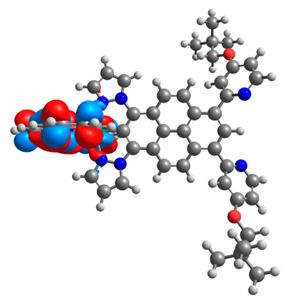
**5a**	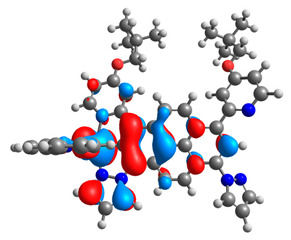	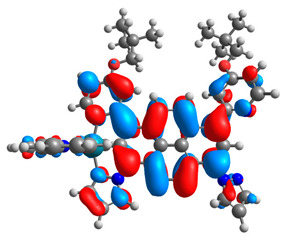	**5b**	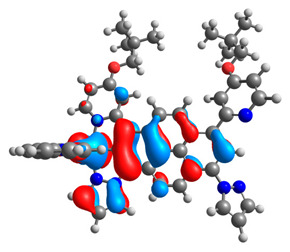	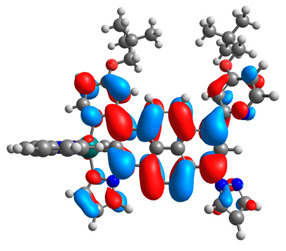
**6a**	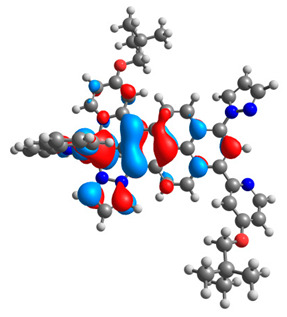	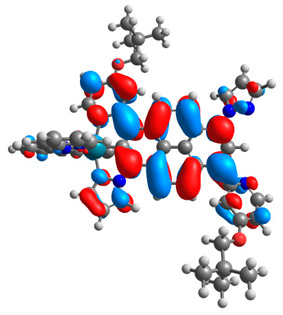	**6b**	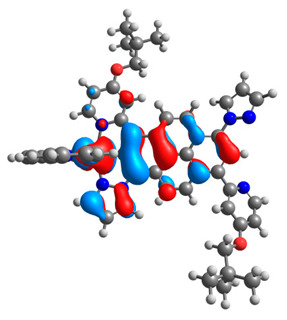	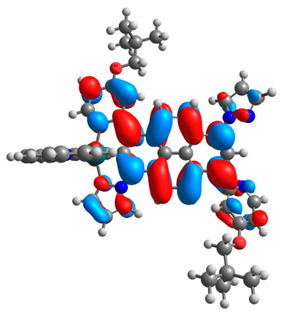

**Table 2:** Energies of HOMOs and LUMOs, values of energy gaps, and bond lengths M(II)-C for complexes **1a**-**6a** and **1b**-**6b**.

	1a	2a	3a	4a	5a	6a	1b	2b	3b	4b	5b	6b
**HOMO (eV)**	−4.94	−5.00	−5.05	−4.98	−4.99	−4.99	−5.08	−5.14	−5.23	−5.15	−5.14	−5.14
**LUMO (eV)**	−2.43	−2.47	−2.31	−2.23	−2.37	−2.38	−2.41	−2.47	−2.30	−2.24	−2.36	−2.36
**ΔE (eV)**	2.51	2.53	2.74	2.75	2.62	2.61	2.67	2.67	2.93	2.91	2.78	2.78
**M(II)-C (Å)**	1.994	1.992	1.998	2.000	1.995	1.995	1.974	1.972	1.980	1.981	1.976	1.976

**Table 3:** Calculated TD-DFT low-energy wavelengths in absorption spectra with oscillator strengths and dominant transitions (>10%) of molecules **1a**-**6a** and **1b**-**6b**.

	Calculated Wavelengths [nm]	Oscillator Strengths	Dominant Transitions (Contribution)
**1a**	595.16	0.2499	H-1→LUMO (93%)
**2a**	601.37	0.2030	H-1→LUMO (95%)
**3a**	529.94	0.1365	H-1→LUMO (37%), H-1→L + 1 (31%), H-1→L + 2 (19%)
**4a**	524.38	0.1993	H-1→L + 1 (72%), H-1→L + 2 (16%)
**5a**	567.98	0.1843	H-1→LUMO (80%), H-1→L + 2 (10%)
**6a**	568.11	0.1790	H-1→LUMO (79%), H-1→L + 2 (10%)
**1b**	550.77	0.3083	H-1→LUMO (84%), H-3→LUMO (10%)
**2b**	556.08	0.2317	H-1→LUMO (88%)
**3b**	488.65	0.2036	H-1→L + 1 (44%), H-1→LUMO (36%)
**4b**	485.74	0.3345	H-1→L + 1 (66%), H-2→L + 1 (21%)
**5b**	524.27	0.2768	H-1→LUMO (78%), H-3→LUMO (11%)
**6b**	524.56	0.2686	H-1→LUMO (77%), H-3→LUMO (11%)

**Table 4:** Natural transition orbitals (NTOs) with pairs occupied (holes) and unoccupied (electrons) of **1a**-**6a** with the contribution of particular parts of molecules: Os/pyrene/pyridine C/pyrazole C/pyridine NC/pyrazole NC/TPY (C = coordinating, NC = not coordinating). The respective number of the state, transition energy, and oscillator strength is listed for each state.

	Hole (HOTO)	Electron (LUTO)
**1a** S_4_ 2.083 eV (0.250) 97%	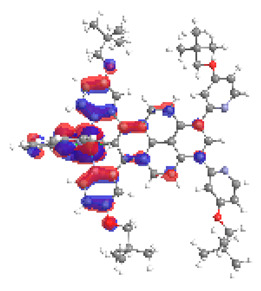	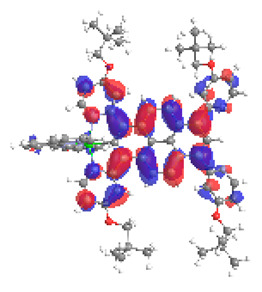
	0.57/0.09/0.18/‒/0.00/‒/0.16	0.02/0.64/0.23/‒/0.08/‒/0.03
**2a** S_4_ 2.062 eV (0.203) 98%	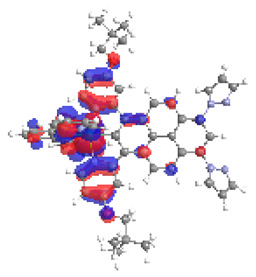	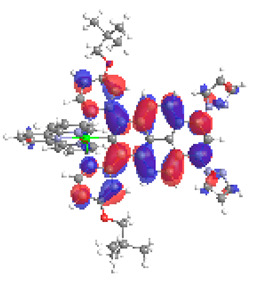
	0.58/0.07/0.18/‒/‒/0.00/0.17	0.01/0.67/0.24/‒/‒/0.05/0.03
**3a** S_6_ 2.340 eV (0.137) 87%	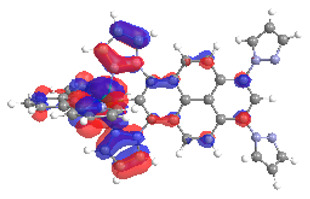	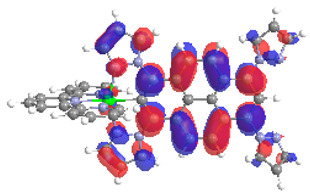
	0.57/0.08/‒/0.19/‒/0.00/0.16	0.02/0.77/‒/0.15/‒0.05/0.01
**4a** S_6_ 2.364 eV (0.199) 88%	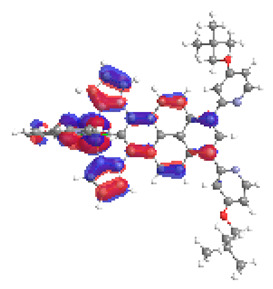	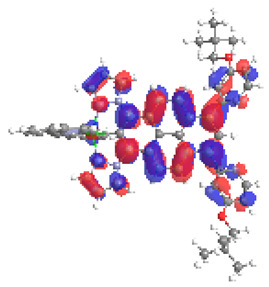
	0.54/0.14/‒/0.17/0.00/‒/0.15	0.01/0.72/‒/0.15/0.12/‒/0.00
**5a** S_5_ 2.183 eV (0.184) 95%	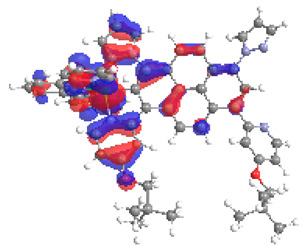	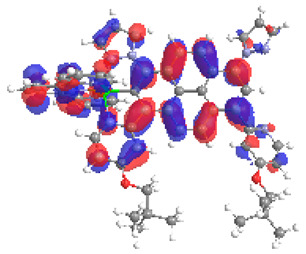
	0.57/0.13/0.10/0.06/0.00/0.00/0.14	0.00/0.51/0.12/0.05/0.04/0.02/0.26
**6a** S_5_ 2.182 eV (0.179) 95%	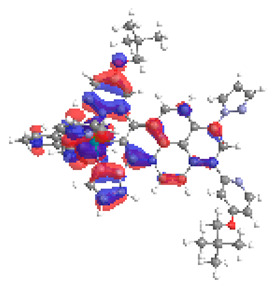	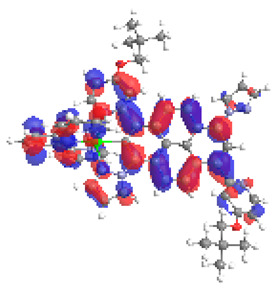
	0.56/0.13/0.10/0.06/0.00/0.00/0.15	0.00/0.52/0.12/0.05/0.04/0.02/0.25

**Table 5:** Natural transition orbitals (NTOs) with pairs occupied (holes) and unoccupied (electrons) of **1b**-**6b** with the contribution of particular parts of molecules: Ru/pyrene/pyridine C/pyrazole C/pyridine NC/pyrazole NC/TPY (C = coordinating, NC = not coordinating). The respective number of the state, transition energy, and oscillator strength is listed for each state.

	Hole (HOTO)	Electron (LUTO)
**1b** S_5_ 2.251 eV (0.308) 94%	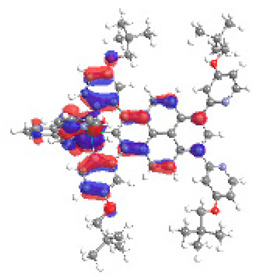	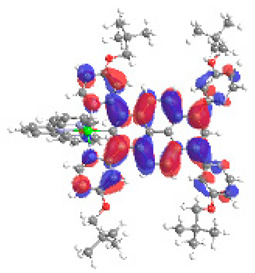
	0.58/0.13/0.16/‒/0.00/‒/0.12	0.00/0.66/0.23/‒/0.09/‒/0.02
**2b** S_4_ 2.230 eV (0.232) 96%	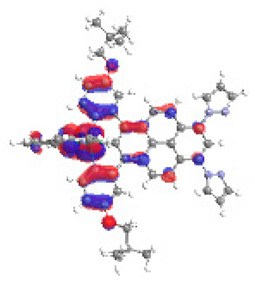	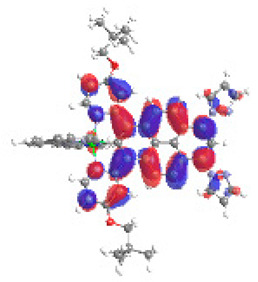
	0.61/0.09/0.17/‒/‒/0.00/0.12	0.01/0.69/0.24/‒/‒/0.05/0.02
**3b** S_6_ 2.537 eV (0.204) 91%	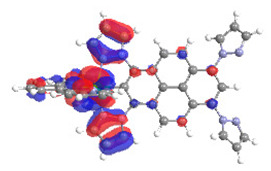	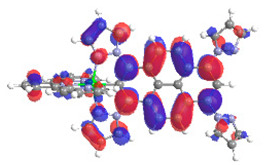
	0.62/0.06/‒/0.18/‒/0.00/0.13	0.00/0.72/‒/0.14/‒0.05/0.08
**4b** S_6_ 2.553 eV (0.335) 88%	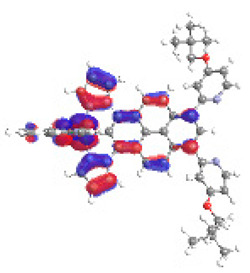	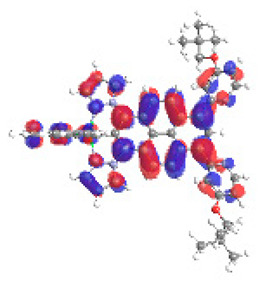
	0.58/0.13/‒/0.17/0.01/‒/0.11	0.00/0.65/‒/0.14/0.10/‒/0.10
**5b** S_5_ 2.365 eV (0.277) 96%	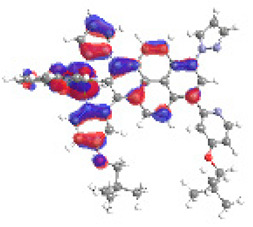	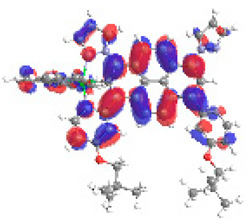
	0.58/0.14/0.09/0.07/0.00/0.00/0.11	0.00/0.64/0.14/0.05/0.05/0.02/0.08
**6b** S_5_ 2.364 eV (0.269) 96%	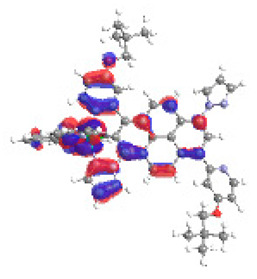	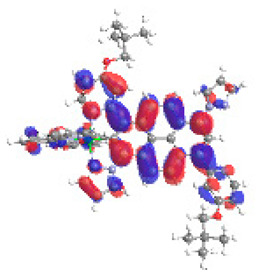
	0.58/0.14/0.09/0.07/0.00/0.00/0.11	0.00/0.65/0.14/0.06/0.05/0.02/0.08

**Table 6:** Spin-density distribution of the lowest energy triplet state for molecules **1a**-**6a** and **1b**-**6b**.

**1a**	**2a**	**3a**	**4a**	**5a**	**6a**
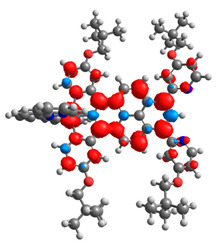	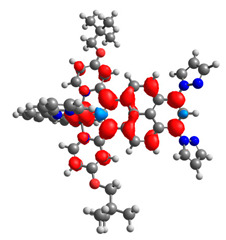	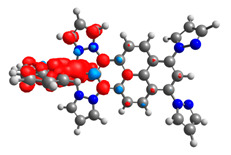	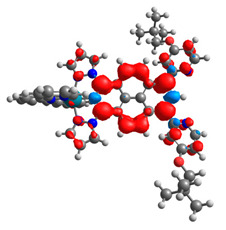	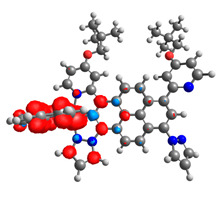	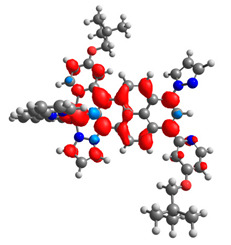
0.410	0.750	0.838	0.043	0.860	0.728
**1b**	**2b**	**3b**	**4b**	**5b**	**6b**
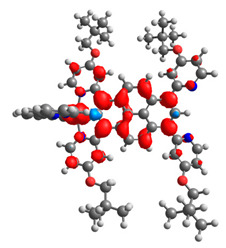	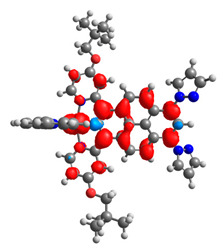	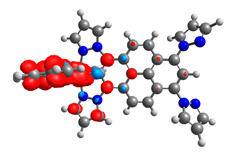	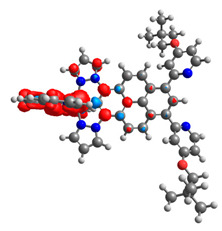	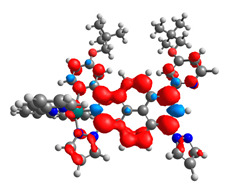	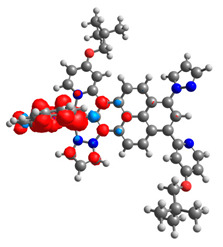
0.751	0.767	0.877	0.866	0.092	0.889

**Table 7:** Energies of HOSOs and LUSOs and bond lengths M-C for complexes **1aox**-**6aox** and **1box**-**6box**.

		1aox	2aox	3aox	4aox	5aox	6aox	1box	2box	3box	4box	5box	6box
**HOSO [eV]**	**α**	−5.75	−5.81	−5.86	−5.77	−5.77	−5.78	−5.78	−5.84	−5.87	−5.78	−5.80	−5.80
	**β**	−5.64	−5.71	−5.79	−5.68	−5.69	−5.69	−5.68	−5.75	−5.81	−5.70	−5.73	−5.73
**LUSO [eV]**	**α**	−2.82	−2.88	−2.90	−2.86	−2.86	−2.86	−2.83	−2.89	−2.88	−2.84	−2.85	−2.85
	**β**	−4.12	−4.15	−4.28	−4.23	−4.16	−4.17	−4.24	−4.27	−4.46	−4.40	−4.31	−4.31
**ΔE [eV]**	**α**	2.93	2.93	2.96	2.91	2.91	2.92	2.95	2.95	2.99	2.94	2.95	2.95
	**β**	1.52	1.56	1.51	1.45	1.53	1.52	1.44	1.48	1.35	1.30	1.42	1.42
**M-C [Å]**		1.975	1.981	1.980	1.970	1.982	1.982	1.951	1.957	1.953	1.946	1.958	1.957

**Table 8:** Calculated TD-DFT low-energy wavelengths in absorption spectra with oscillator strengths and dominant transitions (>10%) of molecules **1aox**-**6aox** and **1box**-**6box**.

	Calculated Wavelengths (nm)	Oscillator Strengths	Dominant Transitions (Contribution)
**1aox**	626.88	0.0811	H-3(β)→LUSO(β) (64%), H-7(β)→LUSO(β) (11%)
**2aox**	601.78	0.0488	H-3(β)→LUSO(β) (55%), H-5(β)→LUSO(β) (12%), H-7(β)→LUSO(β) (12%)
**3aox**	601.75	0.0785	H-3(β)→LUSO(β) (47%), H-7(β)→LUSO(β) (27%)
**4aox**	628.12	0.1183	H-5(β)→LUSO(β) (16%), H-3(β)→LUSO(β) (53%)
**5aox**	607.38	0.0594	H-3(β)→LUSO(β) (58%), H-7(β)→LUSO(β) (15%)
**6aox**	607.08	0.0600	H-3(β)→LUSO(β) (44%), H-4(β)→LUSO(β) (13%), H-6(β)→LUSO(β) (10%), H-7(β)→LUSO(β) (10%)
**1box**	662.66	0.0862	H-3(β)→LUSO(β) (57%), HOSO(α)→LUSO(α) (11%)
**2box**	636.34	0.0592	H-3(β)→LUSO(β) (49%), H-5(β)→LUSO(β) (13%), H-7(β)→LUSO(β) (10%)
**3box**	645.95	0.0616	H-3(β)→LUSO(β) (32%), HOSO(α)→L + 1(α) (23%), H-7(β)→LUSO(β) (17%), HOSO(β)→L + 3(β) (11%)
**4box**	667.77	0.0540	HOSO(α)→L + 1(α) (36%), HOSO(β)→L + 3(β) (24%), H-2(β)→LUSO(β) (24%)
**5box**	650.87	0.0683	H-3(β)→LUSO(β) (48%), H-7(β)→LUSO(β) (12%)
**6box**	650.09	0.0687	H-3(β)→LUSO(β) (33%), H-6(β)→LUSO(β) (14%), H-4(β)→LUSO(β) (13%)

**Table 9:** The optimized structures with HOMOs and LUMOs contours for molecules **7a**-**11a** and **7b**-**11b**.

	HOMO	LUMO		HOMO	LUMO
**7a**	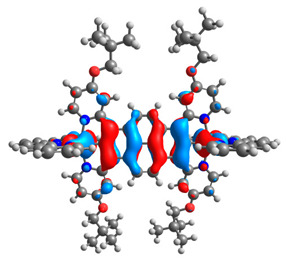	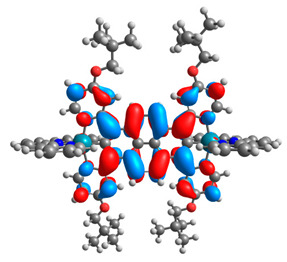	**7b**	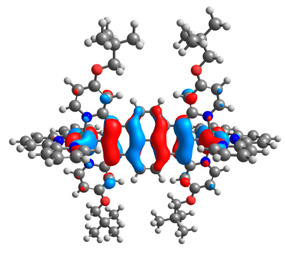	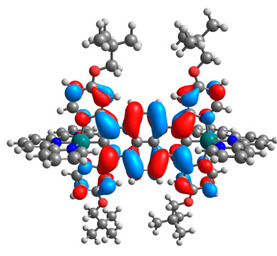
**8a**	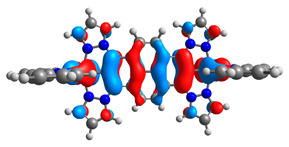	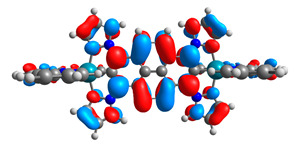	**8b**	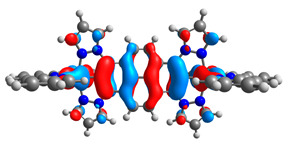	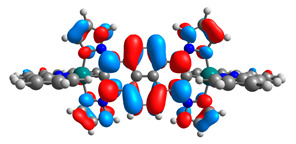
**9a**	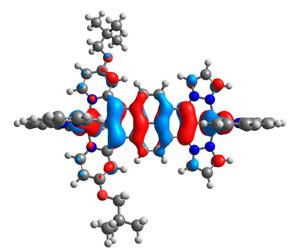	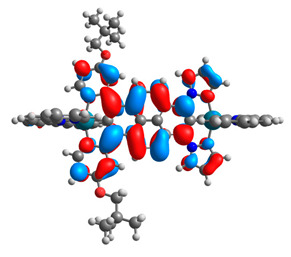	**9b**	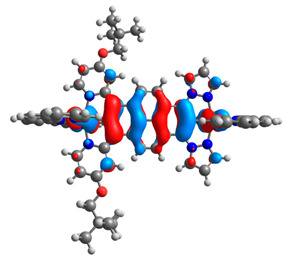	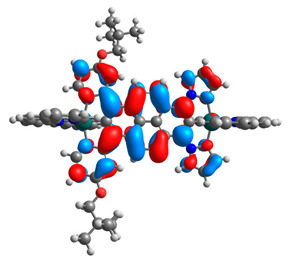
**10a**	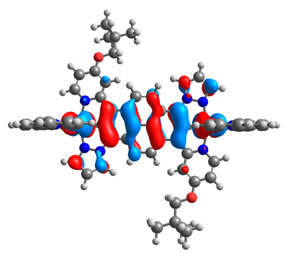	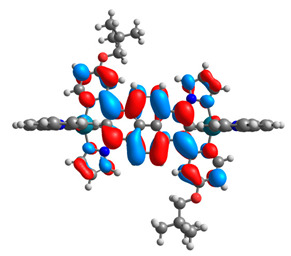	**10b**	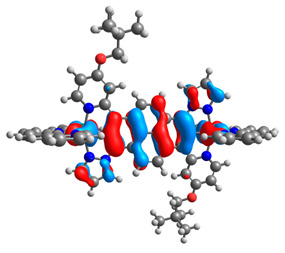	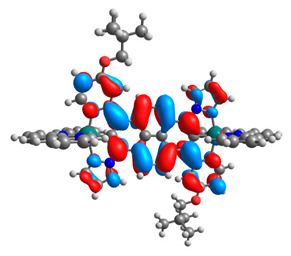
**11a**	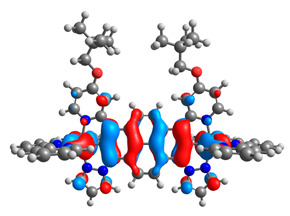	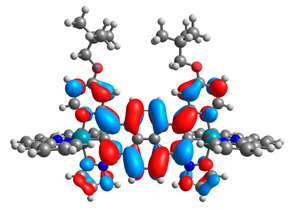	**11b**	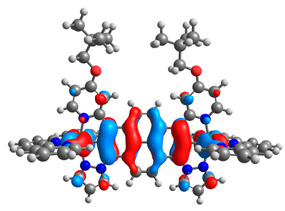	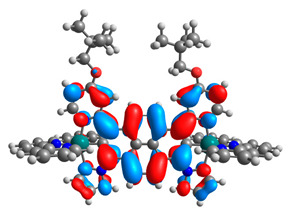

**Table 10:** Energies of HOMOs and LUMOs, values of energy gaps, and bond lengths M-C for complexes **7a**-**11a** and **7b**-**11b**.

	7a	8a	9a	10a	11a	7b	8b	9b	10b	11b
**HOMO [eV]**	−4.73	−4.77	−4.75	−4.74	−4.74	−4.87	−4.96	−4.91	−4.90	−4.91
**LUMO [eV]**	−2.67	−2.31	−2.51	−2.53	−2.51	−2.64	−2.28	−2.48	−2.50	−2.49
**ΔE [eV]**	2.06	2.46	2.24	2.21	2.23	2.23	2.68	2.43	2.40	2.42
**M1-C/M2-C [Å]**	1.995/ 1.995	2.001/ 2.001	1.995/ 2.002	1.997/ 1.997	1.997/ 1.997	1.975/ 1.975	1.982/ 1.982	1.974/ 1.983	1.978/ 1.978	1.978/ 1.978

**Table 11:** Calculated TD-DFT low-energy wavelengths in absorption spectra with oscillator strengths and dominant transitions (>10%) of molecules **7a**-**11a** and **7b**-**11b**.

	Calculated Wavelengths (nm)	Oscillator Strengths	Dominant Transitions (Contribution)
**7a**	771.43	0.1088	HOMO→LUMO (99%)
**8a**	620.45	0.0652	HOMO→LUMO (89%)
**9a**	696.97	0.0975	HOMO→LUMO (97%)
**10a**	705.26	0.0882	HOMO→LUMO (89%)
**11a**	699.02	0.0736	HOMO→LUMO (77%), HOMO→L + 1 (11%)
**7b**	702.22	0.1203	HOMO→LUMO (98%)
**8b**	562.93	0.0695	HOMO→LUMO (85%)
**9b**	653.75	0.0574	HOMO→LUMO (96%)
**10b**	641.54	0.0903	HOMO→LUMO (78%), HOMO→L + 1 (14%)
**11b**	634.74	0.0849	HOMO→LUMO (76%), HOMO→L + 2 (16%)

**Table 12:** Spin-density distribution of the lowest-energy triplet state for molecules **7a**-**11a** and **7b**-**11b**.

**7a**	**8a**	**9a**	**10a**	**11a**
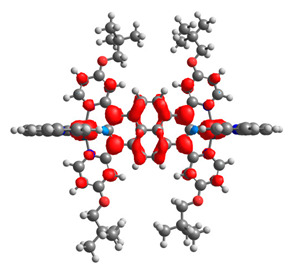	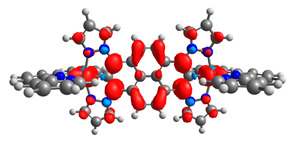	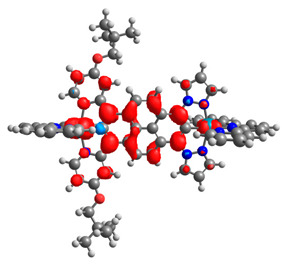	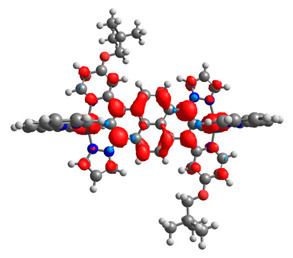	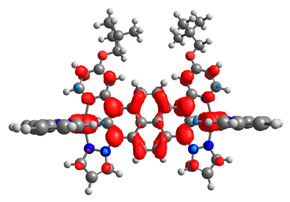
0.345/0.345	0.331/0.331	0.540/0.155	0.320/0.320	0.306/0.376
**7b**	**8b**	**9b**	**10b**	**11b**
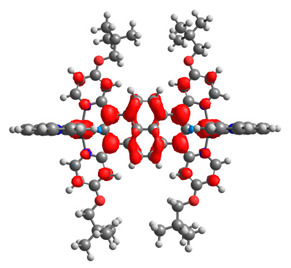	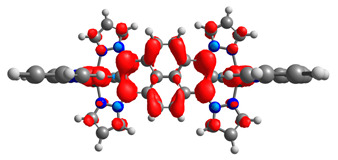	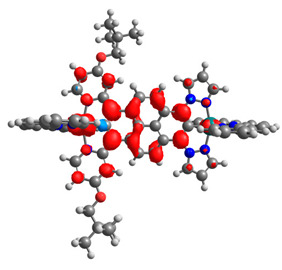	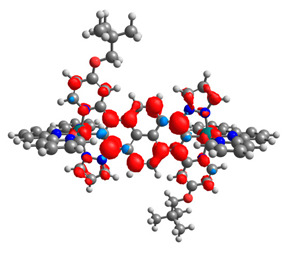	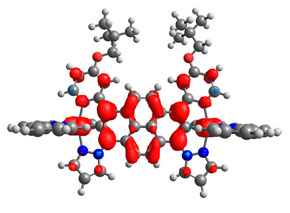
0.333/0.333	0.316/0.316	0.610/0.090	0.167/0.167	0.320/0.331

**Table 13:** The β-HOSO and β-LUSO contours for the complexes **7aox**-**11aox** and **7box**-**11box**.

	β-HOSO	β-LUSO		β-HOSO	β-LUSO
**7aox**	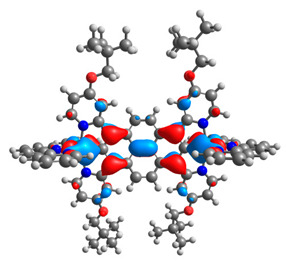	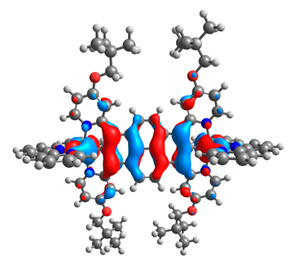	**7box**	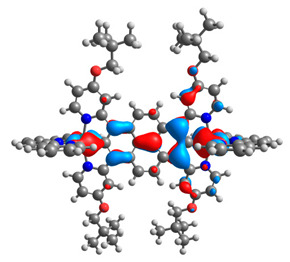	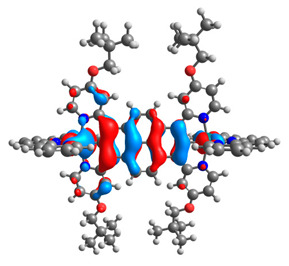
**8aox**	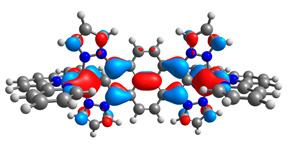	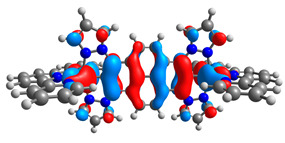	**8box**	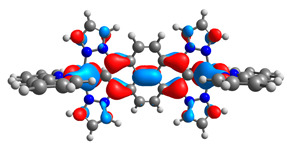	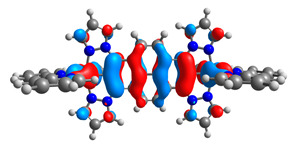
**9aox**	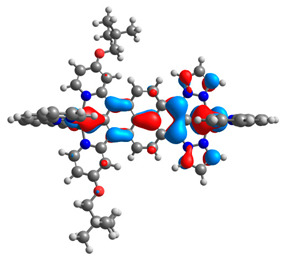	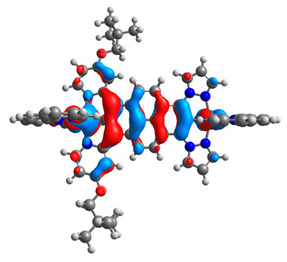	**9box**	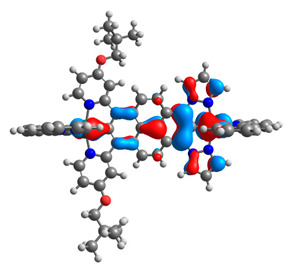	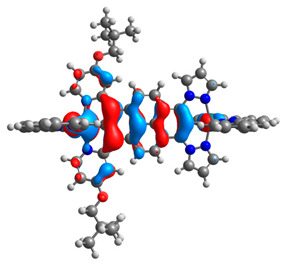
**10aox**	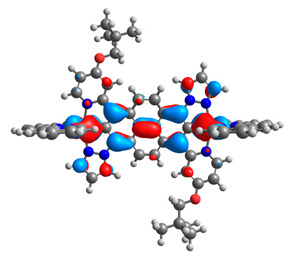	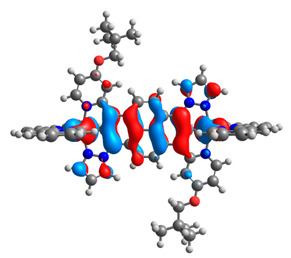	**10box**	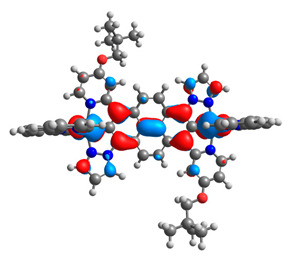	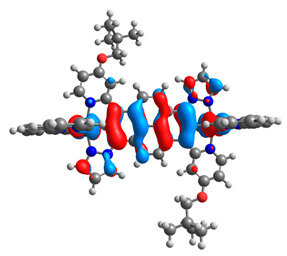
**11aox**	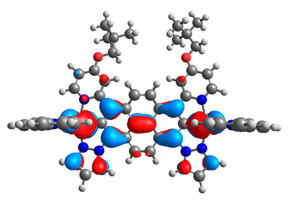	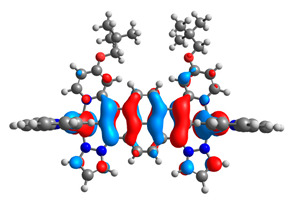	**11box**	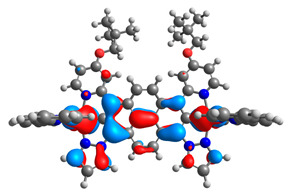	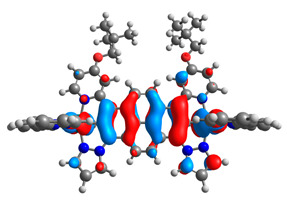

**Table 14:** Energies of HOSOs and LUSOs and bond lengths M-C for complexes **7aox**-**11aox** and **7box**-**11box**.

		7aox	8aox	9aox	10aox	11aox	7box	8box	9box	10box	11box
**HOSO [eV]**	**α**	−5.35	−5.45	−5.31	−5.39	−5.38	−5.47	−5.70	−5.46	−5.61	−5.57
	**β**	−5.13	−5.22	−5.20	−5.19	−5.17	−5.28	−5.39	−5.39	−5.34	−5.32
**LUSO [eV]**	**α**	−3.02	−2.70	−2.88	−2.90	−2.88	−3.02	−2.71	−2.88	−2.90	−2.88
	**β**	−4.13	−4.20	−4.13	−4.16	−4.16	−4.25	−4.40	−4.25	−4.33	−4.32
**ΔE [eV]**	**α**	2.33	2.75	2.43	2.49	2.50	2.45	2.99	2.58	2.71	2.69
	**β**	1.00	1.02	1.07	1.03	1.01	1.03	0.99	1.14	1.01	1.00
**M-C [Å]**		1.965/ 1.965	1.966/ 1.966	1.963/ 1.977	1.965/ 1.965	1.967/ 1.965	1.938/ 1.938	1.945/ 1.945	1.940/ 1.962	1.942/ 1.942	1.948/ 1.940

**Table 15:** Calculated TD-DFT low-energy wavelengths in absorption spectra with oscillator strengths and dominant transitions (>10%) of molecules **7aox**-**11aox** and **7box**-**11box**.

	Calculated Wavelengths (nm)	Oscillator Strengths	Dominant Transitions (Contribution)
**7aox**	2479.19	0.4520	HOSO(β)→LUSO(β) (93%)
**8aox**	2172.11	0.5407	HOSO(β)→LUSO(β) (96%)
**9aox**	1988.84	0.4308	HOSO(β)→LUSO(β) (94%)
**10aox**	2444.48	0.2490	HOSO(β)→LUSO(β) (61%), H-2(β)→LUSO(β) (36%)
	2082.02	0.2460	H-2(β)→LUSO(β) (63%), HOSO(β)→LUSO(β) (34%)
**11aox**	2289.64	0.4195	HOSO(β)→LUSO(β) (83%), H-2(β)→LUSO(β) (13%)
**7box**	2265.79	0.1105	H-5(β)→LUSO(β) (70%), HOSO(β)→LUSO(β) (27%)
	2189.37	0.2830	HOSO(β)→LUSO(β) (69%), H-5(β)→LUSO(β) (27%)
**8box**	2260.42	0.5043	HOSO(β)→LUSO(β) (96%)
**9box**	1765.15	0.3460	HOSO(β)→LUSO(β) (95%)
**10box**	2431.54	0.3392	HOSO(β)→LUSO(β) (78%), H-2(β)→LUSO(β) (19%)
	2069.85	0.1275	H-2(β)→LUSO(β) (79%), HOSO(β)→LUSO(β) (18%)
**11box**	2295.15	0.4071	HOSO(β)→LUSO(β) (92%)

**Table 16:** The optimized structures with HOMO-1, HOMO, LUMO, and LUMO+1 contours for molecules **12**-**17**.

	HOMO-1	HOMO	LUMO	LUMO+1
**12**	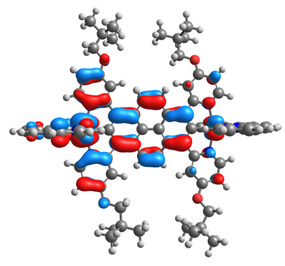	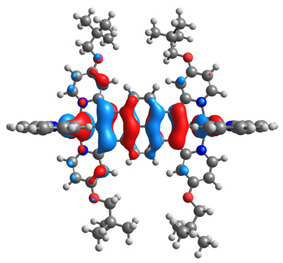	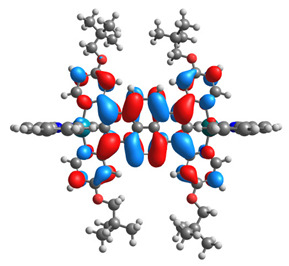	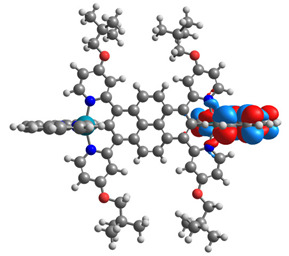
**13**	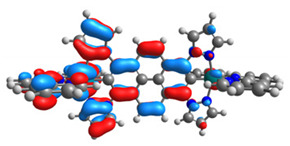	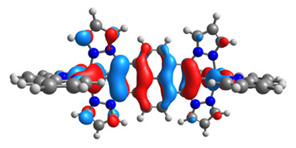	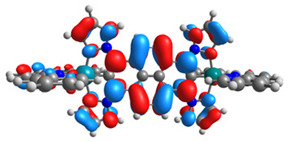	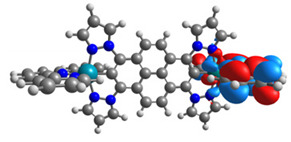
**14**	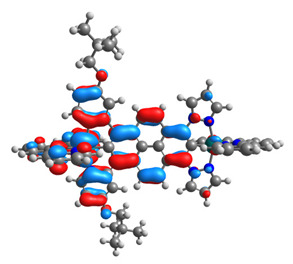	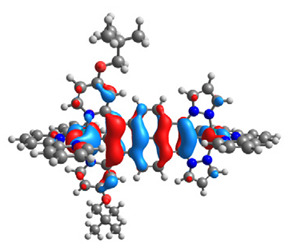	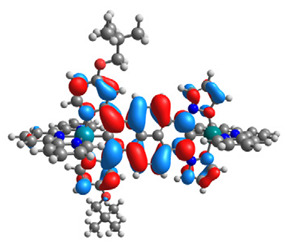	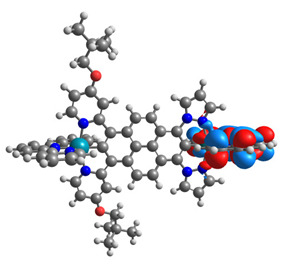
**15**	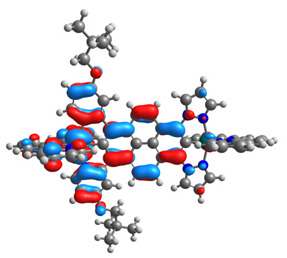	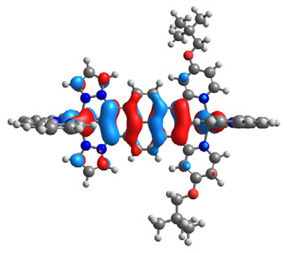	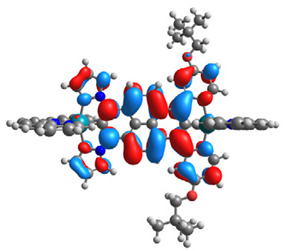	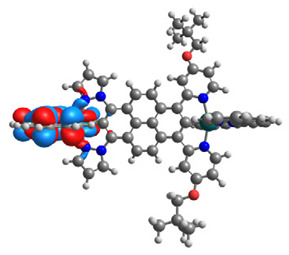
**16**	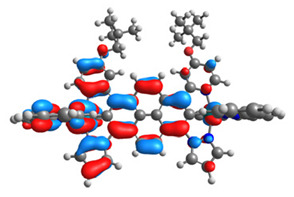	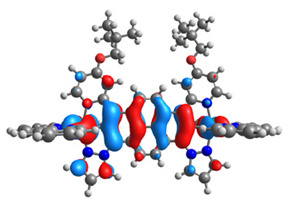	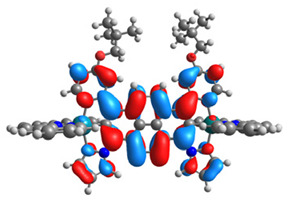	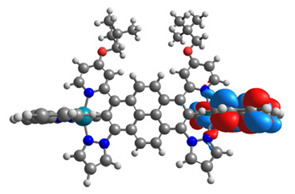
**17**	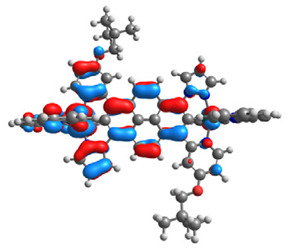	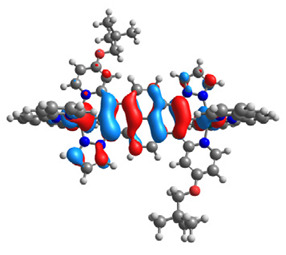	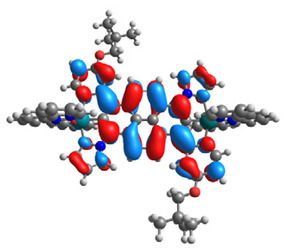	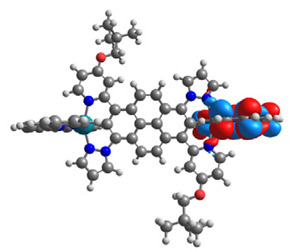

**Table 17:** Energies of HOMOs and LUMOs, values of energy gaps, and bond lengths M-C for complexes **12**-**17**.

	12	13	14	15	16	17
**HOMO [eV]**	−4.79	−4.85	−4.82	−4.81	−4.82	−4.81
**LUMO [eV]**	−2.66	−2.30	−2.50	−2.49	−2.50	−2.52
**ΔE [eV]**	2.13	2.55	2.32	2.32	2.32	2.29
**Os(II)-C [Å]**	1.995	2.001	1.994	2.002	1.997	1.997
**Ru(II)-C [Å]**	1.975	1.982	1.983	1.974	1.978	1.978

**Table 18:** Calculated TD-DFT low-energy wavelengths in absorption spectra with oscillator strengths and dominant transitions (>10%) of molecules **12**-**17**.

	Calculated Wavelengths (nm)	Oscillator Strengths	Dominant Transitions (Contribution)
**12**	739.63	0.1111	HOMO→LUMO (98%)
**13**	588.50	0.0647	HOMO→LUMO (53%), HOMO→L + 4 (41%)
**14**	673.24	0.0982	HOMO→LUMO (97%)
**15**	664.69	0.1011	HOMO→LUMO (98%)
**16**	670.55	0.0950	HOMO→LUMO (97%)
**17**	674.93	0.1012	HOMO→LUMO (91%)

**Table 19:** Spin-density distribution of the lowest energy triplet state (Os/Ru) for molecules **12**-**17** (left side Os, right side Ru).

12	13	14	15	16	17
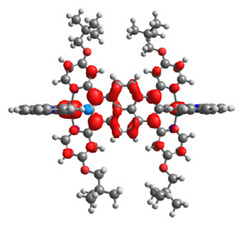	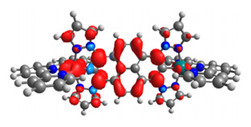	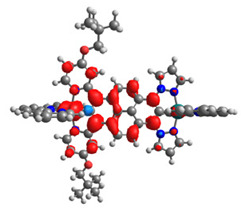	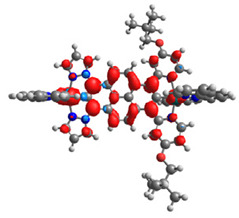	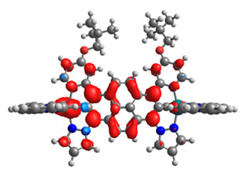	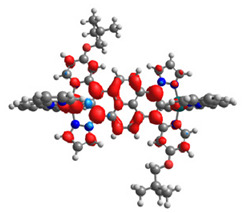
0.593/0.106	0.579/0.089	0.643/0.064	0.500/0.164	0.594/0.096	0.539/0.110

**Table 20:** The α-HOSO, β-HOSO, α-LUSO, and β-LUSO contours for the complexes **12ox**-**17ox** (left side, Os; right side, Ru).

12ox		13ox	
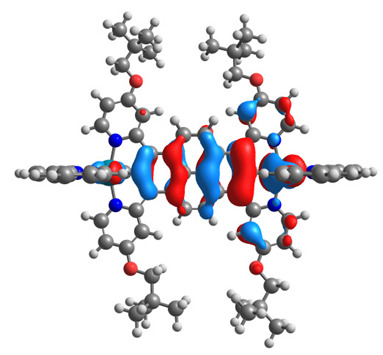	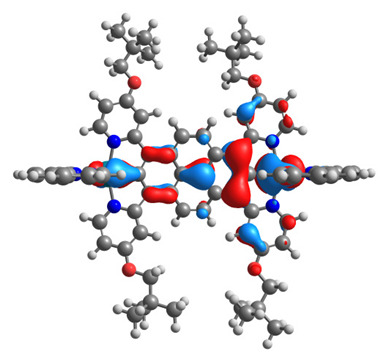	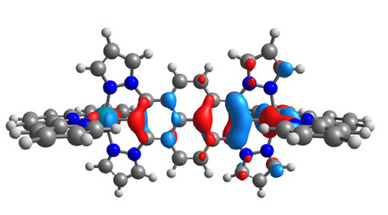	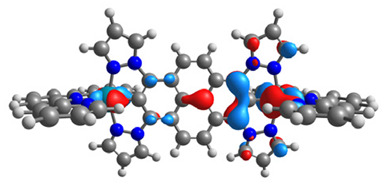
**α-HOSO**	**β-HOSO**	**α-HOSO**	**β-HOSO**
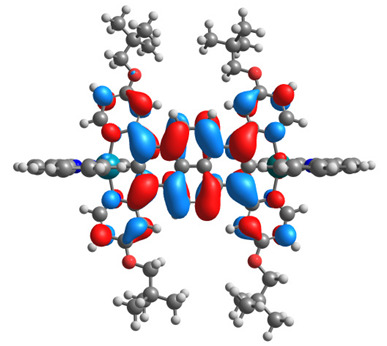	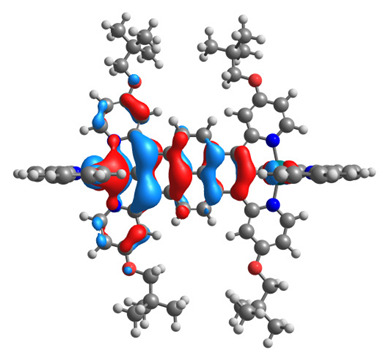	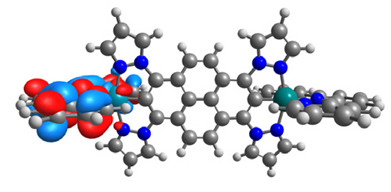	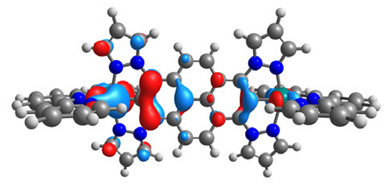
**α-LUSO**	**β-LUSO**	**α-LUSO**	**β-LUSO**
**14ox**		**15ox**	
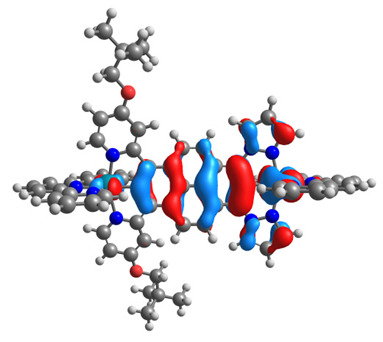	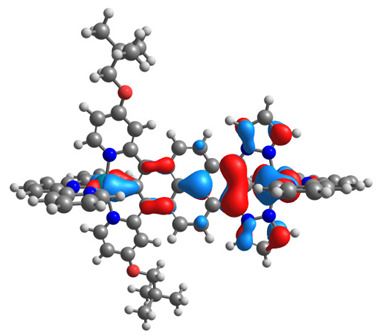	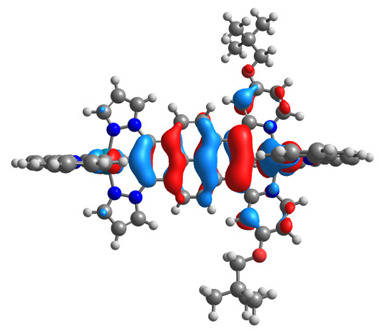	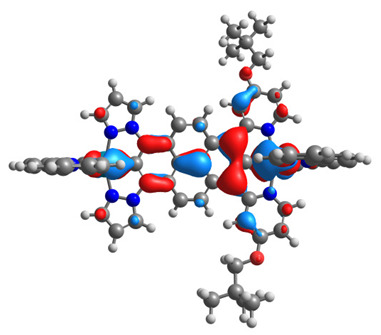
**α-HOSO**	**β-HOSO**	**α-HOSO**	**β-HOSO**
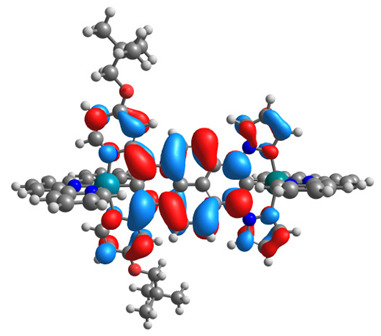	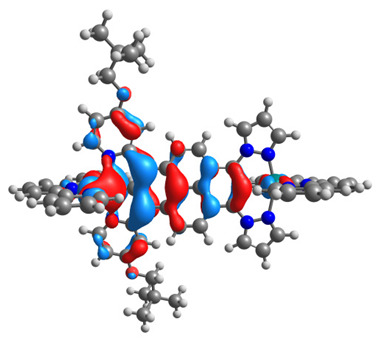	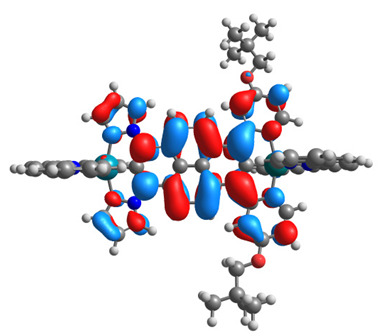	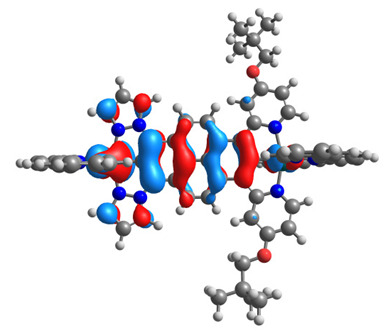
**α-LUSO**	**β-LUSO**	**α-LUSO**	**β-LUSO**
**16ox**		**17ox**	
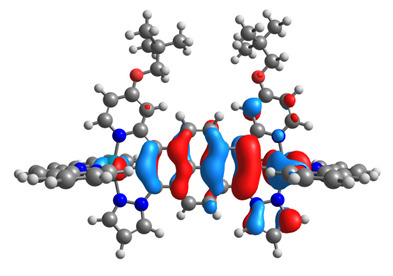	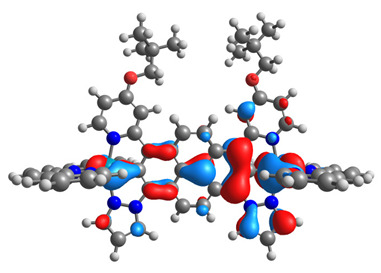	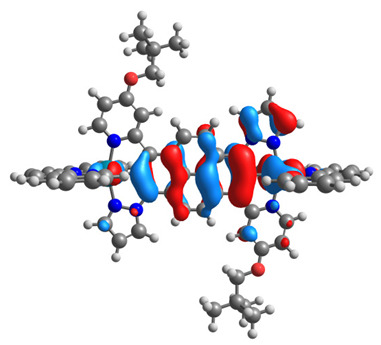	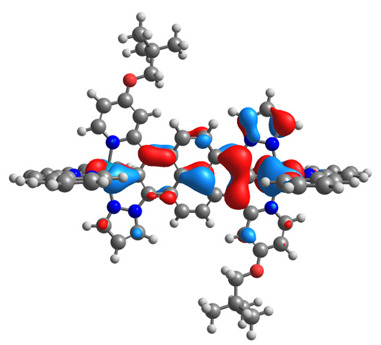
**α-HOSO**	**β-HOSO**	**α-HOSO**	**β-HOSO**
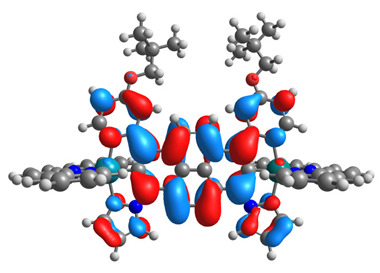	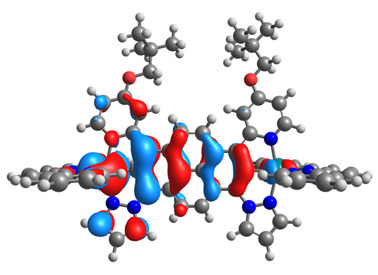	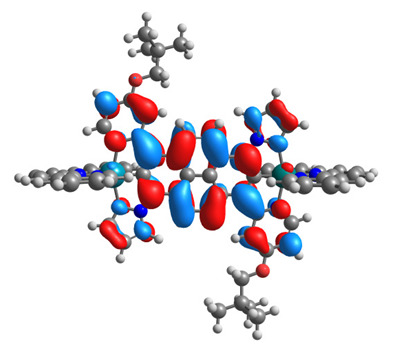	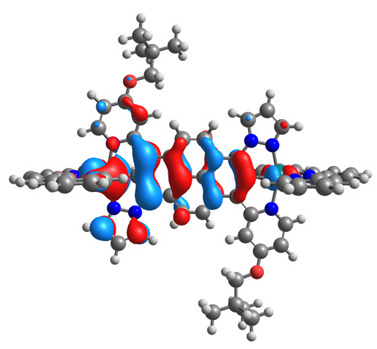
**α-LUSO**	**β-LUSO**	**α-LUSO**	**β-LUSO**

**Table 21:** Energies of HOSOs and LUSOs and bond lengths M-C for complexes **12ox**-**17ox**.

		12ox	13ox	14ox	15ox	16ox	17ox
**HOSO [eV]**	**α**	−5.32	−5.45	−5.38	−5.40	−5.37	−5.38
	**β**	−5.31	−5.41	−5.39	−5.31	−5.34	−5.36
**LUSO [eV]**	**α**	−3.01	−2.80	−2.88	−2.87	−2.88	−2.90
	**β**	−4.11	−4.22	−4.12	−4.20	−4.16	−4.16
**ΔE [eV]**	**α**	2.31	2.65	2.50	2.53	2.49	2.48
	**β**	1.20	1.19	1.27	1.11	1.18	1.20
**Os-C [Å]**		1.965	1.965	1.967	1.965	1.966	1.964
**Ru-C [Å]**		1.957	1.961	1.966	1.951	1.960	1.958

**Table 22:** Calculated TD-DFT low-energy wavelengths (nm) in absorption spectra with oscillator strengths and dominant transitions (>10%) of molecules **12ox**-**17ox**.

	Calculated Wavelengths (nm)	Oscillator Strengths	Dominant Transitions (Contribution)
**12ox**	1654.67	0.3155	HOSO(β)→LUSO(β) (95%)
**13ox**	1618.17	0.3813	HOSO(β)→LUSO(β) (97%)
**14ox**	1476.53	0.3011	HOSO(β)→LUSO(β) (93%)
**15ox**	1876.27	0.1030	H-1(β)→LUSO(β) (63%), HOSO(β)→LUSO(β) (26%)
	1873.72	0.2977	HOSO(β)→LUSO(β) (70%), H-1(β)→LUSO(β) (23%)
**16ox**	1668.25	0.2636	HOSO(β)→LUSO(β) (75%), H-1(β)→LUSO(β) (19%)
**17ox**	1630.30	0.3009	HOSO(β)→LUSO(β) (84%), H-1(β)→LUSO(β) (11%)

## Supplementary Materials

The following are available online at https://www.mdpi.com/article/10.3390/ma14247783/s1, Table S1: The α-HOSO, β-HOSO and α-LUSO, β-LUSO contours for the complexes 1aox-6aox and 1box-6box. Cartesian coordinates of DFT-optimized structure of complexes 1a-6a, 1b-6b, 1aox-6aox, 1box-6box, 7a-11a, 7b-11b, 7aox-11aox, 7box-11box, 12-17, 12ox-17ox with values of charge and multiplicity.
