# Supplementary material for: DFT/TD-DFT Framework of Mixed-Metal Complexes with Symmetrical and Unsymmetrical Bridging Ligands—Step-By-Step Investigations: Mononuclear, Dinuclear Homometallic, and Heterometallic for Optoelectronic Applications
Source: Materials (Basel). 2021 Dec 16;14(24):7783. doi: 10.3390/ma14247783 (PMC8709181; doi:10.3390/ma14247783)
Supplement: Supplementary file 1 [file materials-14-07783-s001.zip › materials-1468410-supplementary.pdf]

**Table S1.** The $\alpha$-HOSO, $\beta$-HOSO, $\alpha$-LUSO, $\beta$-LUSO contours for the complexes **1aox-6aox** and **1box-6box.**

| **1aox** | | **1box** | |
|---|---|---|---|
| 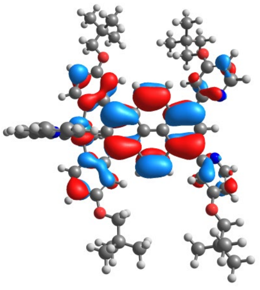 | 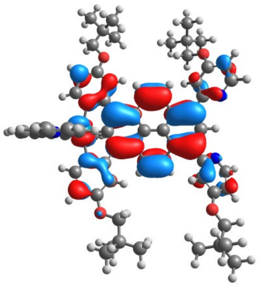 | 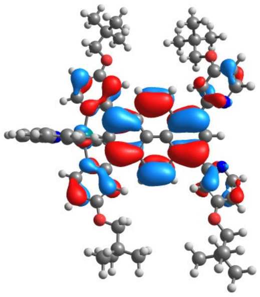 | 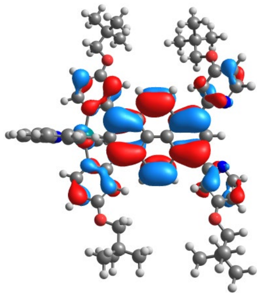 |
| **α-HOSO** | **β-HOSO** | **α-HOSO** | **β-HOSO** |
| 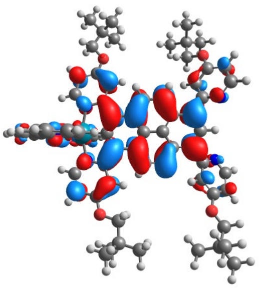 | 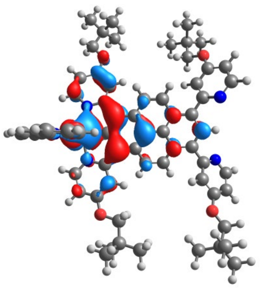 | 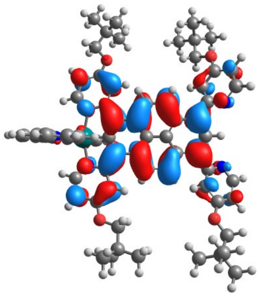 | 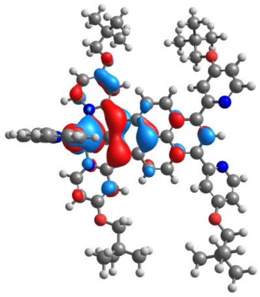 |
| **α-LUSO** | **β-LUSO** | **α-LUSO** | **β-LUSO** |
| **2aox** | | **2box** | |
| 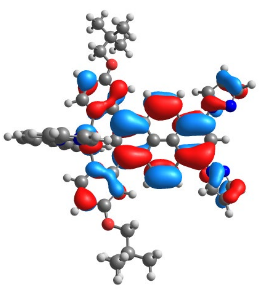 | 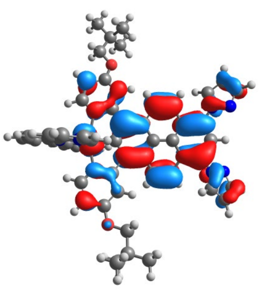 | 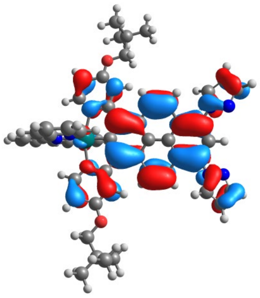 | 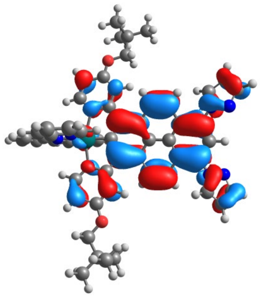 |
| **α-HOSO** | **β-HOSO** | **α-HOSO** | **β-HOSO** |
| 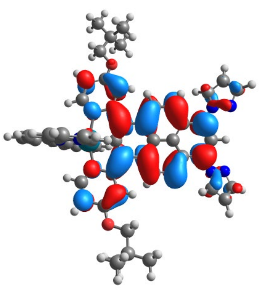 | 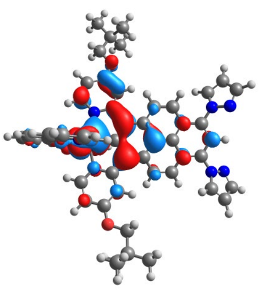 | 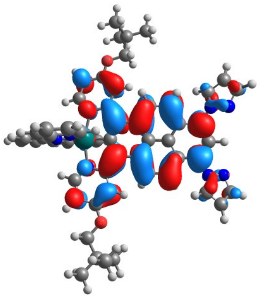 | 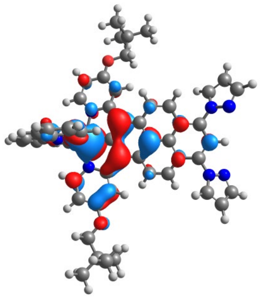 |
| **α-LUSO** | **β-LUSO** | **α-LUSO** | **β-LUSO** |
| **3aox** | | **3box** | |
| 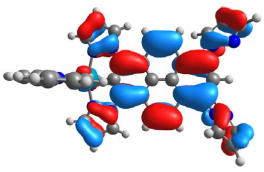 | 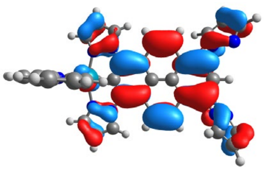 | 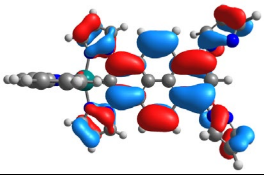 | 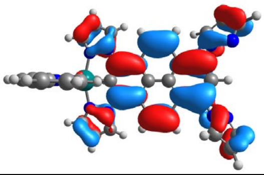 |
| **α-HOSO** | **β-HOSO** | **α-HOSO** | **β-HOSO** |

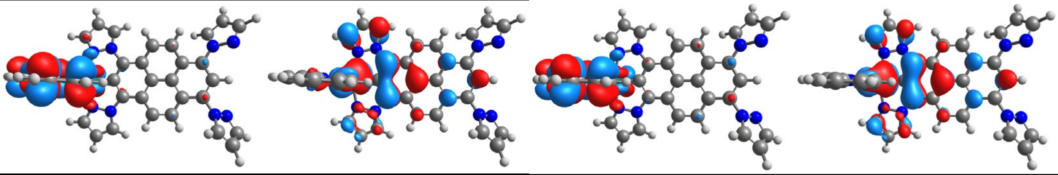

| α-LUSO | β-LUSO | α-LUSO | β-LUSO |
|---|---|---|---|
| **4aox** | | **4box** | |

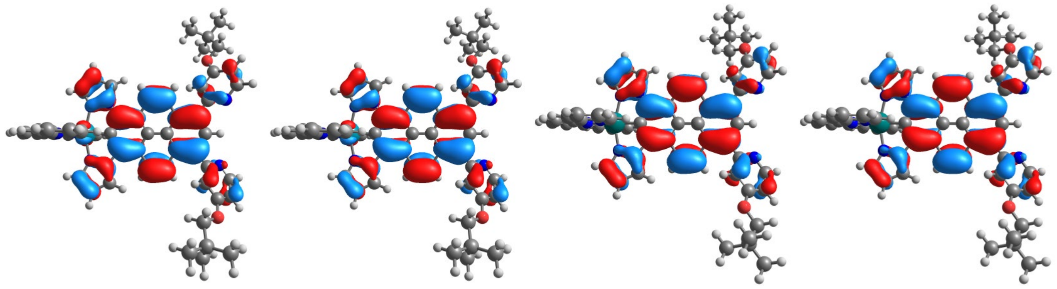

| α-HOSO | β-HOSO | α-HOSO | β-HOSO |
|---|---|---|---|

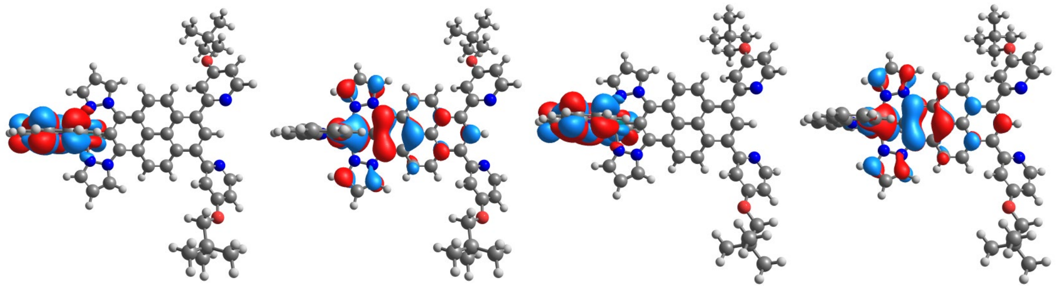

| α-LUSO | β-LUSO | α-LUSO | β-LUSO |
|---|---|---|---|
| **5aox** | | **5box** | |

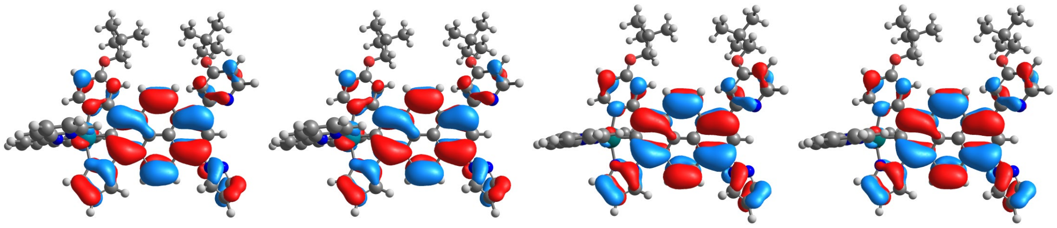

| α-HOSO | β-HOSO | α-HOSO | β-HOSO |
|---|---|---|---|

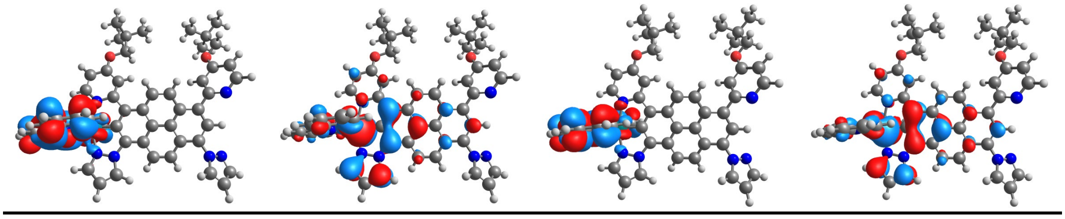

| α-LUSO | β-LUSO | α-LUSO | β-LUSO |
|---|---|---|---|
| **6aox** | | **6box** | |

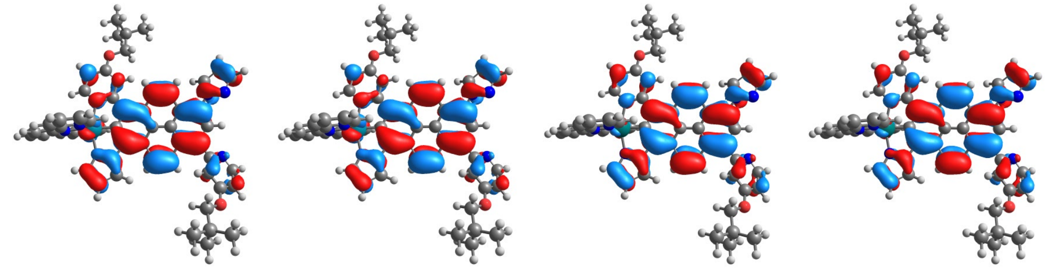

| α-HOSO | β-HOSO | α-HOSO | β-HOSO |
|---|---|---|---|

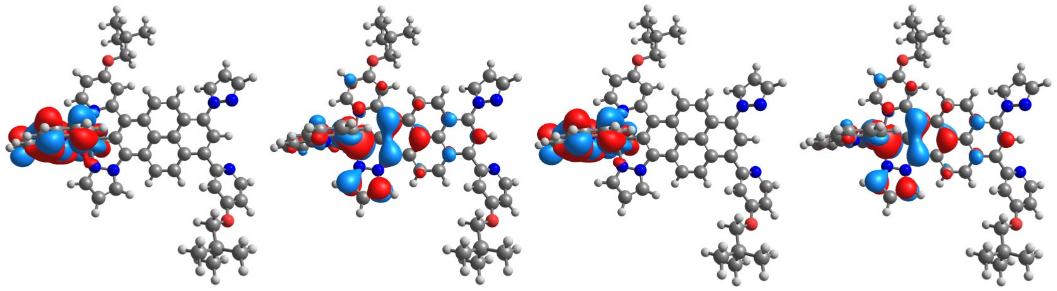

| α-LUSO | β-LUSO | α-LUSO | β-LUSO |
|---|---|---|---|

Cartesian coordinates of DFT-optimized structure of **1a** complex by B3LYP/Def2-TZVP; 6-31G(d,p)/$CH_3CN$, charge = 1, multiplicity = 1

| | | | |
|---|---|---|---|
| Os | 2.222519000 | -0.045200000 | 0.045462000 |
| N | -7.923901000 | 2.604759000 | -1.745530000 |
| N | 1.667812000 | -2.059780000 | 0.320821000 |
| N | 2.612038000 | 0.262951000 | 2.081448000 |
| N | 4.263912000 | -0.153684000 | 0.122264000 |
| N | 2.728491000 | -0.396500000 | -1.957588000 |
| N | -8.215371000 | -1.502655000 | 1.406863000 |
| N | 1.902279000 | 2.017318000 | -0.266390000 |
| C | 1.701868000 | 0.471849000 | 3.061826000 |
| H | 0.667278000 | 0.471661000 | 2.741752000 |
| C | 2.052347000 | 0.672925000 | 4.389151000 |
| H | 1.274342000 | 0.836128000 | 5.126834000 |
| C | 3.403614000 | 0.660994000 | 4.743736000 |
| H | 3.711972000 | 0.815661000 | 5.772314000 |
| C | 4.351923000 | 0.445361000 | 3.750452000 |
| H | 5.406925000 | 0.430143000 | 3.998207000 |
| C | 3.950713000 | 0.247231000 | 2.426146000 |
| C | 4.891047000 | 0.007968000 | 1.321528000 |
| C | 6.283388000 | -0.068680000 | 1.394521000 |
| H | 6.798435000 | 0.056546000 | 2.340004000 |
| C | 7.011135000 | -0.311704000 | 0.227161000 |
| C | 6.351052000 | -0.474751000 | -0.993202000 |
| H | 6.918856000 | -0.662741000 | -1.897106000 |
| C | 4.957779000 | -0.390521000 | -1.026823000 |
| C | 4.083075000 | -0.526437000 | -2.200846000 |
| C | 4.558925000 | -0.767626000 | -3.492963000 |
| H | 5.625060000 | -0.866015000 | -3.661426000 |
| C | 3.669999000 | -0.880253000 | -4.555801000 |
| H | 4.036131000 | -1.067713000 | -5.559670000 |
| C | 2.302457000 | -0.746721000 | -4.303180000 |
| H | 1.569079000 | -0.826296000 | -5.098135000 |
| C | 1.876936000 | -0.508051000 | -3.004344000 |
| H | 0.826965000 | -0.397706000 | -2.762477000 |
| C | -8.652843000 | 3.688701000 | -2.055743000 |
| C | 2.526980000 | -3.102550000 | 0.398649000 |
| H | -9.518989000 | 3.514139000 | -2.691938000 |

| | | | |
|---|---|---|---|
| H | 3.578769000 | -2.844569000 | 0.438798000 |
| C | -8.369245000 | 4.976570000 | -1.623889000 |
| C | 2.131053000 | -4.422720000 | 0.416921000 |
| H | -8.991342000 | 5.820244000 | -1.902727000 |
| H | 2.858660000 | -5.223289000 | 0.480485000 |
| C | -7.252175000 | 5.167078000 | -0.795635000 |
| C | 0.758929000 | -4.710437000 | 0.315428000 |
| C | -6.476531000 | 4.050717000 | -0.460196000 |
| C | -0.144703000 | -3.647245000 | 0.243872000 |
| H | -5.621064000 | 4.137162000 | 0.196279000 |
| H | -1.197933000 | -3.839558000 | 0.115118000 |
| C | -6.842338000 | 2.791659000 | -0.965122000 |
| C | 0.307257000 | -2.317744000 | 0.287256000 |
| C | -6.085145000 | 1.563532000 | -0.581807000 |
| C | -0.532961000 | -1.115112000 | 0.197256000 |
| C | -6.829429000 | 0.457019000 | -0.169377000 |
| C | 0.232586000 | 0.057109000 | -0.024768000 |
| C | -6.235618000 | -0.730529000 | 0.261423000 |
| C | -0.392120000 | 1.300205000 | -0.292301000 |
| C | -7.148943000 | -1.846413000 | 0.646585000 |
| C | 0.569229000 | 2.412194000 | -0.327905000 |
| C | -6.953134000 | -3.148719000 | 0.181762000 |
| C | 0.264615000 | 3.774852000 | -0.319912000 |
| H | -6.116651000 | -3.399935000 | -0.460567000 |
| H | -0.760033000 | 4.113909000 | -0.271633000 |
| C | -7.872974000 | -4.152073000 | 0.525375000 |
| C | 1.272275000 | 4.747254000 | -0.330688000 |
| C | -8.968239000 | -3.803952000 | 1.325718000 |
| C | 2.610355000 | 4.327396000 | -0.334975000 |
| H | -9.717391000 | -4.523793000 | 1.630136000 |
| H | 3.442241000 | 5.018884000 | -0.350113000 |
| C | -9.079870000 | -2.470625000 | 1.722279000 |
| C | 2.859719000 | 2.964480000 | -0.286486000 |
| H | -9.926991000 | -2.171561000 | 2.338023000 |
| H | 3.880968000 | 2.602736000 | -0.251611000 |
| C | -4.819916000 | -0.815947000 | 0.323182000 |
| C | -1.797753000 | 1.347960000 | -0.490845000 |
| C | -4.022789000 | 0.287690000 | -0.131561000 |
| C | -2.586841000 | 0.205605000 | -0.103789000 |
| C | -4.669155000 | 1.476857000 | -0.608767000 |
| C | -1.946380000 | -1.014789000 | 0.316695000 |
| C | -3.844509000 | 2.506757000 | -1.166372000 |
| C | -2.779228000 | -2.030428000 | 0.891953000 |
| H | -4.314768000 | 3.343236000 | -1.670481000 |
| H | -2.317733000 | -2.872840000 | 1.391787000 |
| C | -2.484573000 | 2.446014000 | -1.105819000 |
| C | -4.139153000 | -1.935948000 | 0.899602000 |
| H | -1.909377000 | 3.225428000 | -1.589263000 |
| H | -4.720279000 | -2.715022000 | 1.379232000 |
| O | -7.017993000 | 6.428189000 | -0.372629000 |
| O | 0.861438000 | 6.030602000 | -0.327149000 |

| | | | |
|---|---|---|---|
| O | 0.422782000 | -6.014534000 | 0.286245000 |
| O | -7.624863000 | -5.387185000 | 0.038387000 |
| C | -8.547806000 | -6.450485000 | 0.332599000 |
| C | -8.060751000 | -7.743604000 | -0.337262000 |
| C | -9.083192000 | -8.844530000 | 0.005908000 |
| C | -6.674920000 | -8.133350000 | 0.210759000 |
| C | -7.993188000 | -7.555049000 | -1.864595000 |
| C | -0.961378000 | -6.390623000 | 0.172687000 |
| C | -1.068198000 | -7.922535000 | 0.194366000 |
| C | -2.562778000 | -8.273874000 | 0.062430000 |
| C | -0.286330000 | -8.524314000 | -0.988704000 |
| C | -0.520746000 | -8.471203000 | 1.525808000 |
| C | -5.885768000 | 6.686539000 | 0.474974000 |
| C | -5.851724000 | 8.180235000 | 0.831336000 |
| C | -5.719779000 | 9.027905000 | -0.448145000 |
| C | -7.132236000 | 8.568857000 | 1.594740000 |
| C | -4.621646000 | 8.403449000 | 1.732402000 |
| C | 1.844470000 | 7.082565000 | -0.317099000 |
| C | 1.128925000 | 8.441202000 | -0.307053000 |
| C | 0.252316000 | 8.568495000 | 0.953352000 |
| C | 0.263270000 | 8.592631000 | -1.572296000 |
| C | 2.221614000 | 9.528060000 | -0.291715000 |
| H | -9.542920000 | -6.176394000 | -0.041857000 |
| H | -8.609508000 | -6.582482000 | 1.420910000 |
| H | -10.083244000 | -8.594351000 | -0.368821000 |
| H | -9.155386000 | -9.000404000 | 1.089243000 |
| H | -8.783072000 | -9.794882000 | -0.449866000 |
| H | -6.706995000 | -8.280826000 | 1.297457000 |
| H | -5.931237000 | -7.359441000 | -0.004794000 |
| H | -6.333222000 | -9.069732000 | -0.245375000 |
| H | -8.976044000 | -7.289357000 | -2.273417000 |
| H | -7.666816000 | -8.482157000 | -2.350064000 |
| H | -7.288156000 | -6.762701000 | -2.135724000 |
| H | -1.521753000 | -5.954447000 | 1.009966000 |
| H | -1.365461000 | -5.990273000 | -0.766283000 |
| H | -2.698017000 | -9.361301000 | 0.073228000 |
| H | -2.982500000 | -7.893339000 | -0.876758000 |
| H | -3.147716000 | -7.855234000 | 0.890420000 |
| H | -0.671162000 | -8.152387000 | -1.946406000 |
| H | -0.378585000 | -9.616583000 | -0.990917000 |
| H | 0.777585000 | -8.273455000 | -0.929493000 |
| H | -1.074271000 | -8.060309000 | 2.379286000 |
| H | 0.536897000 | -8.219527000 | 1.654190000 |
| H | -0.616783000 | -9.562671000 | 1.557264000 |
| H | -4.967910000 | 6.395461000 | -0.053100000 |
| H | -5.970822000 | 6.079736000 | 1.385910000 |
| H | -4.803768000 | 8.775028000 | -0.996438000 |
| H | -5.677219000 | 10.094286000 | -0.197472000 |
| H | -6.571173000 | 8.869941000 | -1.117780000 |
| H | -8.024306000 | 8.405436000 | 0.981690000 |
| H | -7.102252000 | 9.627617000 | 1.877161000 |

| | | | |
|---|---|---|---|
| H | -7.238461000 | 7.978905000 | 2.513704000 |
| H | -3.691501000 | 8.138482000 | 1.214648000 |
| H | -4.682557000 | 7.804740000 | 2.649464000 |
| H | -4.553106000 | 9.457366000 | 2.024908000 |
| H | 2.478202000 | 6.988770000 | -1.208544000 |
| H | 2.475498000 | 6.973375000 | 0.574587000 |
| H | -0.237964000 | 9.548497000 | 0.978713000 |
| H | 0.854467000 | 8.470015000 | 1.865125000 |
| H | -0.525893000 | 7.798595000 | 0.975536000 |
| H | -0.508742000 | 7.817921000 | -1.619910000 |
| H | 0.874602000 | 8.520262000 | -2.480396000 |
| H | -0.234049000 | 9.569443000 | -1.579283000 |
| H | 2.856623000 | 9.446301000 | 0.598826000 |
| H | 1.763316000 | 10.523434000 | -0.285803000 |
| H | 2.866172000 | 9.461557000 | -1.176638000 |
| H | -7.910793000 | 0.525033000 | -0.179303000 |
| H | 8.093165000 | -0.374223000 | 0.268404000 |

Cartesian coordinates of DFT-optimized structure of **2a** complex by B3LYP/Def2-TZVP; 6-31G(d,p)/$CH_3CN$, charge = 1, multiplicity = 1

| | | | |
|---|---|---|---|
| Os | 2.194111000 | -0.984051000 | 0.014047000 |
| N | 0.972237000 | -2.694704000 | 0.186749000 |
| N | 2.685401000 | -0.969422000 | 2.051781000 |
| N | 4.074634000 | -1.793636000 | 0.018704000 |
| N | 2.528390000 | -1.357594000 | -2.021623000 |
| N | 2.614902000 | 1.075718000 | -0.163155000 |
| C | 1.910069000 | -0.533002000 | 3.072415000 |
| H | 0.935690000 | -0.155757000 | 2.787609000 |
| C | 2.318518000 | -0.561055000 | 4.398165000 |
| H | 1.649531000 | -0.195749000 | 5.169593000 |
| C | 3.586111000 | -1.059963000 | 4.707510000 |
| H | 3.936624000 | -1.095356000 | 5.733583000 |
| C | 4.395037000 | -1.514201000 | 3.672230000 |
| H | 5.382145000 | -1.908115000 | 3.884659000 |
| C | 3.939691000 | -1.466718000 | 2.351508000 |
| C | 4.731576000 | -1.933928000 | 1.204067000 |
| C | 6.017433000 | -2.477713000 | 1.226910000 |
| H | 6.554712000 | -2.595890000 | 2.160741000 |
| C | 6.608220000 | -2.871791000 | 0.023971000 |
| C | 5.919118000 | -2.720488000 | -1.181713000 |
| H | 6.380547000 | -3.026036000 | -2.113630000 |
| C | 4.635695000 | -2.170798000 | -1.164145000 |
| C | 3.754820000 | -1.923510000 | -2.315251000 |
| C | 4.107414000 | -2.226301000 | -3.633442000 |
| H | 5.074601000 | -2.669318000 | -3.840761000 |
| C | 3.222666000 | -1.961177000 | -4.672399000 |
| H | 3.493119000 | -2.195597000 | -5.696533000 |
| C | 1.985030000 | -1.388898000 | -4.369105000 |
| H | 1.260323000 | -1.162592000 | -5.143478000 |

| | | | |
|---|---|---|---|
| C | 1.679488000 | -1.105061000 | -3.045648000 |
| H | 0.732954000 | -0.659525000 | -2.765676000 |
| C | 1.412485000 | -3.974607000 | 0.182949000 |
| H | 2.488229000 | -4.102795000 | 0.213334000 |
| C | 0.579633000 | -5.072191000 | 0.133266000 |
| H | 0.981793000 | -6.078502000 | 0.132446000 |
| C | -0.806794000 | -4.856485000 | 0.049343000 |
| C | -1.280941000 | -3.542146000 | 0.063312000 |
| H | -2.335213000 | -3.347199000 | -0.050678000 |
| C | -0.392329000 | -2.460157000 | 0.172263000 |
| C | -5.033445000 | 3.405545000 | -0.406461000 |
| C | -0.760035000 | -1.036775000 | 0.172991000 |
| C | -6.131938000 | 2.622423000 | -0.066631000 |
| C | 0.366398000 | -0.192134000 | 0.005334000 |
| C | -5.958282000 | 1.289266000 | 0.289434000 |
| C | 0.214654000 | 1.205771000 | -0.174731000 |
| C | 1.503999000 | 1.913415000 | -0.164085000 |
| C | 1.692401000 | 3.292435000 | -0.059559000 |
| H | 0.850290000 | 3.962196000 | 0.039342000 |
| C | 2.976187000 | 3.852137000 | -0.033756000 |
| C | 4.083533000 | 2.994391000 | -0.103143000 |
| H | 5.104214000 | 3.353029000 | -0.096624000 |
| C | 3.842599000 | 1.629866000 | -0.149061000 |
| H | 4.673611000 | 0.933990000 | -0.164868000 |
| C | -4.669826000 | 0.711554000 | 0.361663000 |
| C | -1.084232000 | 1.750932000 | -0.348652000 |
| C | -3.539494000 | 1.500451000 | -0.034626000 |
| C | -2.222111000 | 0.929269000 | -0.020032000 |
| C | -3.729382000 | 2.861285000 | -0.445961000 |
| C | -2.049554000 | -0.459761000 | 0.322547000 |
| C | -2.597428000 | 3.584461000 | -0.938922000 |
| C | -3.186843000 | -1.155667000 | 0.851764000 |
| H | -2.745228000 | 4.559921000 | -1.387578000 |
| H | -3.047402000 | -2.129390000 | 1.303871000 |
| C | -1.343327000 | 3.054236000 | -0.889054000 |
| C | -4.432401000 | -0.603076000 | 0.873007000 |
| H | -0.527829000 | 3.616083000 | -1.326012000 |
| H | -5.253221000 | -1.157465000 | 1.313107000 |
| O | 3.038585000 | 5.193738000 | 0.067674000 |
| O | -1.580164000 | -5.955000000 | -0.048006000 |
| C | -3.009914000 | -5.810686000 | -0.132957000 |
| C | -3.652896000 | -7.202613000 | -0.220550000 |
| C | -5.177113000 | -6.991502000 | -0.308826000 |
| C | -3.156082000 | -7.935242000 | -1.481258000 |
| C | -3.313688000 | -8.022833000 | 1.038491000 |
| C | 4.326298000 | 5.836930000 | 0.111325000 |
| C | 4.127354000 | 7.352550000 | 0.256833000 |
| C | 3.385271000 | 7.664457000 | 1.570177000 |
| C | 3.333226000 | 7.900010000 | -0.944328000 |
| C | 5.529646000 | 7.991550000 | 0.287588000 |
| H | -3.368156000 | -5.275371000 | 0.756256000 |

| | | | |
|---|---|---|---|
| H | -3.258470000 | -5.218234000 | -1.022970000 |
| H | -5.689174000 | -7.958014000 | -0.375908000 |
| H | -5.450188000 | -6.405996000 | -1.195224000 |
| H | -5.561691000 | -6.469332000 | 0.575733000 |
| H | -3.400595000 | -7.369296000 | -2.388763000 |
| H | -3.630168000 | -8.920241000 | -1.561572000 |
| H | -2.071502000 | -8.081228000 | -1.453953000 |
| H | -3.673262000 | -7.521464000 | 1.945649000 |
| H | -2.233394000 | -8.169812000 | 1.137423000 |
| H | -3.787676000 | -9.010025000 | 0.990077000 |
| H | 4.871457000 | 5.607595000 | -0.813418000 |
| H | 4.895866000 | 5.440746000 | 0.961907000 |
| H | 3.258018000 | 8.746717000 | 1.688932000 |
| H | 3.945949000 | 7.294689000 | 2.437656000 |
| H | 2.392974000 | 7.202336000 | 1.584727000 |
| H | 2.335686000 | 7.452330000 | -0.998275000 |
| H | 3.851447000 | 7.691377000 | -1.888490000 |
| H | 3.214323000 | 8.986345000 | -0.859234000 |
| H | 6.122082000 | 7.617846000 | 1.131670000 |
| H | 5.446757000 | 9.079203000 | 0.392501000 |
| H | 6.084892000 | 7.784935000 | -0.635504000 |
| H | -7.124886000 | 3.051842000 | -0.080035000 |
| H | 7.606159000 | -3.296640000 | 0.026042000 |
| N | -5.275133000 | 4.766286000 | -0.744235000 |
| C | -4.646920000 | 5.882138000 | -0.259725000 |
| C | -5.298619000 | 6.974383000 | -0.791738000 |
| C | -6.327683000 | 6.419695000 | -1.586947000 |
| N | -6.322558000 | 5.088700000 | -1.555557000 |
| N | -7.122546000 | 0.535989000 | 0.607844000 |
| N | -8.092816000 | 1.084360000 | 1.393393000 |
| C | -9.068068000 | 0.178104000 | 1.406924000 |
| C | -8.750700000 | -0.956429000 | 0.625200000 |
| C | -7.495988000 | -0.687931000 | 0.120917000 |
| H | -6.859401000 | -1.242607000 | -0.551927000 |
| H | -9.350694000 | -1.837200000 | 0.448778000 |
| H | -9.964908000 | 0.369106000 | 1.982590000 |
| H | -7.068433000 | 6.935678000 | -2.184651000 |
| H | -5.069228000 | 8.016697000 | -0.624941000 |
| H | -3.819952000 | 5.806086000 | 0.430118000 |

Cartesian coordinates of DFT-optimized structure of **3a** complex by B3LYP/Def2-TZVP; 6-31G(d,p)/$CH_3CN$, charge = 1, multiplicity = 1

| | | | |
|---|---|---|---|
| C | 4.055649000 | 2.468887000 | -0.171154000 |
| C | 5.413802000 | 2.473468000 | -0.258118000 |
| C | 6.168145000 | 1.262223000 | -0.187373000 |
| C | 5.459823000 | 0.023858000 | -0.033253000 |
| C | 4.024677000 | 0.012564000 | 0.043564000 |
| C | 3.300393000 | 1.263663000 | 0.008256000 |
| C | 6.197027000 | -1.206554000 | 0.031828000 |

| | | | |
|---|---|---|---|
| C | 5.469913000 | -2.437547000 | 0.004991000 |
| C | 4.110294000 | -2.454605000 | 0.040359000 |
| C | 3.333919000 | -1.252940000 | 0.142016000 |
| C | 1.893486000 | 1.202535000 | 0.117337000 |
| C | 1.206712000 | -0.017315000 | 0.198150000 |
| C | 1.929904000 | -1.216672000 | 0.260912000 |
| C | 7.579394000 | 1.234178000 | -0.264935000 |
| C | 8.287532000 | 0.048231000 | -0.114205000 |
| C | 7.608758000 | -1.154884000 | 0.049661000 |
| H | 3.546983000 | 3.414364000 | -0.279246000 |
| H | 5.930946000 | 3.417135000 | -0.381395000 |
| H | 3.612688000 | -3.407906000 | -0.065097000 |
| H | 6.010802000 | -3.371812000 | -0.088486000 |
| N | 0.978508000 | 2.305927000 | 0.130526000 |
| N | -0.370656000 | 1.983264000 | 0.037013000 |
| C | -1.046509000 | 3.132967000 | 0.129734000 |
| C | -0.165222000 | 4.210435000 | 0.304231000 |
| C | 1.098487000 | 3.654321000 | 0.313022000 |
| N | 1.056474000 | -2.339212000 | 0.419233000 |
| N | -0.303037000 | -2.082988000 | 0.294080000 |
| C | -0.932237000 | -3.236015000 | 0.544794000 |
| C | -0.008315000 | -4.244979000 | 0.857350000 |
| C | 1.232551000 | -3.643605000 | 0.783359000 |
| N | 8.331902000 | 2.428010000 | -0.465342000 |
| N | 9.369135000 | 2.724532000 | 0.367722000 |
| C | 9.909402000 | 3.827886000 | -0.147257000 |
| C | 8.236851000 | 3.321237000 | -1.496904000 |
| C | 9.241097000 | 4.249996000 | -1.318964000 |
| N | 8.386184000 | -2.336708000 | 0.200684000 |
| N | 9.470046000 | -2.544577000 | -0.600145000 |
| C | 10.019776000 | -3.666050000 | -0.138956000 |
| C | 9.311532000 | -4.189983000 | 0.966859000 |
| C | 8.271959000 | -3.305551000 | 1.161280000 |
| C | -0.106334000 | -0.261369000 | -2.943151000 |
| C | -0.389315000 | -0.358913000 | -4.298825000 |
| C | -1.722113000 | -0.402451000 | -4.711288000 |
| C | -2.721696000 | -0.346779000 | -3.745605000 |
| C | -2.386696000 | -0.247637000 | -2.393047000 |
| N | -1.065620000 | -0.204725000 | -1.991606000 |
| C | -3.387282000 | -0.185758000 | -1.314617000 |
| N | -2.828776000 | -0.100124000 | -0.078580000 |
| C | -3.580141000 | -0.037334000 | 1.052741000 |
| C | -4.973439000 | -0.057916000 | 0.962285000 |
| C | -5.567678000 | -0.142959000 | -0.298839000 |
| C | -4.777191000 | -0.207603000 | -1.448304000 |
| C | -2.766925000 | 0.051595000 | 2.277720000 |
| N | -1.398551000 | 0.063266000 | 2.091549000 |
| C | -0.603866000 | 0.151444000 | 3.181966000 |
| C | -1.101209000 | 0.224944000 | 4.476246000 |
| C | -2.483086000 | 0.209252000 | 4.672249000 |
| C | -3.315072000 | 0.122992000 | 3.560779000 |

| | | | |
|---|---|---|---|
| H | 0.913401000 | -0.226469000 | -2.580300000 |
| H | 0.426722000 | -0.399926000 | -5.011844000 |
| H | -1.978958000 | -0.479203000 | -5.762470000 |
| H | -3.764341000 | -0.381535000 | -4.039087000 |
| H | -5.590369000 | -0.008125000 | 1.851745000 |
| H | -5.242136000 | -0.273148000 | -2.424948000 |
| H | 0.460559000 | 0.161860000 | 2.982366000 |
| H | -0.410960000 | 0.293079000 | 5.309806000 |
| H | -2.905692000 | 0.264125000 | 5.669881000 |
| H | -4.391416000 | 0.110757000 | 3.686530000 |
| Os | -0.788013000 | -0.060325000 | 0.087190000 |
| H | -2.125584000 | 3.122278000 | 0.079358000 |
| H | -0.412986000 | 5.254802000 | 0.419033000 |
| H | 2.048504000 | 4.128815000 | 0.476840000 |
| H | -2.010878000 | -3.276130000 | 0.500278000 |
| H | -0.215185000 | -5.272968000 | 1.113656000 |
| H | 2.204082000 | -4.047089000 | 1.005125000 |
| H | 10.758828000 | 4.284749000 | 0.344671000 |
| H | 7.490557000 | 3.208998000 | -2.269432000 |
| H | 9.461828000 | 5.101539000 | -1.945941000 |
| H | 10.904421000 | -4.062988000 | -0.620668000 |
| H | 9.529788000 | -5.077914000 | 1.542021000 |
| H | 7.488237000 | -3.270732000 | 1.903197000 |
| H | 9.369519000 | 0.058577000 | -0.133937000 |
| H | -6.648679000 | -0.158858000 | -0.385770000 |

Cartesian coordinates of DFT-optimized structure of **4a** complex by B3LYP/Def2-TZVP; 6-31G(d,p)/$CH_3CN$, charge = 1, multiplicity = 1

| | | | |
|---|---|---|---|
| Os | 1.814688000 | -3.093382000 | 0.017988000 |
| N | -1.564150000 | 6.844250000 | -1.646444000 |
| N | -0.140932000 | -3.697764000 | 0.458508000 |
| N | 2.445760000 | -3.203508000 | 2.017880000 |
| N | 2.853081000 | -4.857221000 | 0.049662000 |
| N | 1.633874000 | -3.750878000 | -1.967627000 |
| N | -5.229020000 | 4.778381000 | 1.386036000 |
| N | 3.301061000 | -1.693293000 | -0.439674000 |
| C | 2.201069000 | -2.309472000 | 3.002840000 |
| H | 1.611162000 | -1.448181000 | 2.714397000 |
| C | 2.664870000 | -2.468435000 | 4.301724000 |
| H | 2.433958000 | -1.714364000 | 5.046091000 |
| C | 3.419746000 | -3.599291000 | 4.618091000 |
| H | 3.797555000 | -3.754924000 | 5.623077000 |
| C | 3.680547000 | -4.529229000 | 3.617197000 |
| H | 4.264177000 | -5.415776000 | 3.835846000 |
| C | 3.191861000 | -4.324903000 | 2.324377000 |
| C | 3.424617000 | -5.263424000 | 1.214270000 |
| C | 4.138163000 | -6.463122000 | 1.256000000 |
| H | 4.601334000 | -6.804541000 | 2.174386000 |
| C | 4.248850000 | -7.224547000 | 0.090381000 |

| | | | |
|---|---|---|---|
| C | 3.653621000 | -6.789206000 | -1.095817000 |
| H | 3.742271000 | -7.382579000 | -1.998273000 |
| C | 2.948956000 | -5.583661000 | -1.095625000 |
| C | 2.256231000 | -4.955354000 | -2.232872000 |
| C | 2.213326000 | -5.517540000 | -3.511094000 |
| H | 2.707741000 | -6.463697000 | -3.697903000 |
| C | 1.538820000 | -4.866667000 | -4.538760000 |
| H | 1.503621000 | -5.300634000 | -5.532441000 |
| C | 0.911689000 | -3.650197000 | -4.263758000 |
| H | 0.372351000 | -3.104151000 | -5.029908000 |
| C | 0.981760000 | -3.131638000 | -2.977797000 |
| H | 0.508930000 | -2.191815000 | -2.720308000 |
| C | -1.066196000 | 8.055244000 | -1.943901000 |
| C | -0.829814000 | -4.838054000 | 0.580907000 |
| H | -1.678754000 | 8.674973000 | -2.596667000 |
| C | 0.148079000 | 8.540928000 | -1.480471000 |
| H | 0.506871000 | 9.528125000 | -1.751169000 |
| C | 0.908782000 | 7.722790000 | -0.630594000 |
| C | 0.408645000 | 6.455617000 | -0.306504000 |
| H | 0.940619000 | 5.795873000 | 0.366222000 |
| C | -0.825025000 | 6.053517000 | -0.845330000 |
| N | -1.075347000 | -2.670566000 | 0.469052000 |
| C | -1.428651000 | 4.737056000 | -0.482001000 |
| C | -0.567367000 | -1.348681000 | 0.274692000 |
| C | -2.769683000 | 4.734048000 | -0.096307000 |
| C | 0.802104000 | -1.369383000 | -0.013534000 |
| C | -3.435289000 | 3.574301000 | 0.304199000 |
| C | 1.456971000 | -0.171200000 | -0.321094000 |
| C | -4.883676000 | 3.702593000 | 0.639970000 |
| N | 2.861025000 | -0.377340000 | -0.492318000 |
| C | -5.831465000 | 2.807291000 | 0.139655000 |
| H | -5.543965000 | 1.973627000 | -0.490940000 |
| C | -7.190440000 | 3.002176000 | 0.430983000 |
| C | -7.550655000 | 4.105712000 | 1.214490000 |
| H | -8.578681000 | 4.321043000 | 1.477096000 |
| C | -6.527306000 | 4.948876000 | 1.650016000 |
| C | 4.634176000 | -1.652028000 | -0.544345000 |
| H | -6.780712000 | 5.818690000 | 2.254484000 |
| C | -2.718579000 | 2.351370000 | 0.371683000 |
| C | 0.758696000 | 1.046401000 | -0.463067000 |
| C | -1.350372000 | 2.306838000 | -0.063935000 |
| C | -0.624259000 | 1.065714000 | -0.048059000 |
| C | -0.713444000 | 3.512258000 | -0.516100000 |
| C | -1.289599000 | -0.141411000 | 0.382417000 |
| C | 0.608866000 | 3.411058000 | -1.057341000 |
| C | -2.608199000 | -0.022706000 | 0.931075000 |
| H | 1.054285000 | 4.280510000 | -1.526344000 |
| H | -3.065019000 | -0.874277000 | 1.418002000 |
| C | 1.307236000 | 2.242636000 | -1.030211000 |
| C | -3.284448000 | 1.159045000 | 0.928246000 |
| H | 2.277279000 | 2.213097000 | -1.508585000 |

| | | | |
|---|---|---|---|
| H | -4.266430000 | 1.205322000 | 1.383955000 |
| O | 2.073747000 | 8.235585000 | -0.179117000 |
| O | -8.050120000 | 2.098205000 | -0.086640000 |
| C | -9.459541000 | 2.260765000 | 0.147996000 |
| C | -10.221320000 | 1.118884000 | -0.540721000 |
| C | -11.721318000 | 1.344598000 | -0.268379000 |
| C | -9.784954000 | -0.237087000 | 0.045901000 |
| C | -9.960246000 | 1.148474000 | -2.058592000 |
| C | 2.899630000 | 7.446301000 | 0.693697000 |
| C | 4.156265000 | 8.249082000 | 1.061828000 |
| C | 4.958761000 | 8.588748000 | -0.208494000 |
| C | 3.760014000 | 9.541990000 | 1.799704000 |
| C | 5.006273000 | 7.360579000 | 1.990839000 |
| H | -9.780351000 | 3.231840000 | -0.251635000 |
| H | -9.648539000 | 2.249639000 | 1.229514000 |
| H | -12.062186000 | 2.304531000 | -0.675260000 |
| H | -11.940524000 | 1.333630000 | 0.806230000 |
| H | -12.314555000 | 0.552281000 | -0.738999000 |
| H | -9.978115000 | -0.281031000 | 1.125013000 |
| H | -8.716564000 | -0.413379000 | -0.115003000 |
| H | -10.339928000 | -1.055047000 | -0.428101000 |
| H | -10.273488000 | 2.105490000 | -2.494165000 |
| H | -10.523499000 | 0.351710000 | -2.558234000 |
| H | -8.898095000 | 1.005287000 | -2.281448000 |
| H | 3.175745000 | 6.514402000 | 0.182617000 |
| H | 2.329941000 | 7.192342000 | 1.597276000 |
| H | 5.259759000 | 7.677770000 | -0.740703000 |
| H | 5.868612000 | 9.142075000 | 0.051543000 |
| H | 4.370982000 | 9.205050000 | -0.896252000 |
| H | 3.138844000 | 10.184791000 | 1.167757000 |
| H | 4.654182000 | 10.107909000 | 2.085851000 |
| H | 3.197131000 | 9.318000000 | 2.714447000 |
| H | 5.305736000 | 6.430564000 | 1.492257000 |
| H | 4.458922000 | 7.096013000 | 2.903874000 |
| H | 5.918758000 | 7.889091000 | 2.289330000 |
| H | -3.316810000 | 5.669344000 | -0.107520000 |
| H | 4.799882000 | -8.158638000 | 0.106314000 |
| C | 3.939626000 | 0.452197000 | -0.596811000 |
| C | 5.080668000 | -0.325426000 | -0.645385000 |
| C | -2.327608000 | -3.204271000 | 0.565148000 |
| C | -2.207141000 | -4.578007000 | 0.651275000 |
| H | -0.301833000 | -5.780415000 | 0.599223000 |
| H | 5.199069000 | -2.572758000 | -0.527824000 |
| H | 3.833603000 | 1.522599000 | -0.593764000 |
| H | 6.098631000 | 0.023026000 | -0.732786000 |
| H | -3.209429000 | -2.589292000 | 0.530120000 |
| H | -3.009755000 | -5.294072000 | 0.742864000 |

Cartesian coordinates of DFT-optimized structure of **5a** complex by B3LYP/Def2-TZVP; 6-31G(d,p)/$CH_3CN$, charge = 1, multiplicity = 1

| | | | |
|---|---|---|---|
| Os | 1.911837000 | -3.000474000 | 0.005771000 |
| N | -0.062718000 | -3.671240000 | 0.306657000 |
| N | 2.425574000 | -3.168181000 | 2.033689000 |
| N | 2.989017000 | -4.741584000 | 0.041192000 |
| N | 1.855923000 | -3.602618000 | -2.003244000 |
| N | -5.136897000 | 4.854166000 | 1.419563000 |
| C | 2.092101000 | -2.318596000 | 3.032667000 |
| H | 1.496055000 | -1.464163000 | 2.736916000 |
| C | 2.477603000 | -2.513080000 | 4.351759000 |
| H | 2.178019000 | -1.793811000 | 5.106047000 |
| C | 3.244989000 | -3.633752000 | 4.675781000 |
| H | 3.563463000 | -3.815909000 | 5.696702000 |
| C | 3.595487000 | -4.518362000 | 3.661750000 |
| H | 4.189684000 | -5.396565000 | 3.886270000 |
| C | 3.183216000 | -4.280328000 | 2.347913000 |
| C | 3.508737000 | -5.171656000 | 1.223197000 |
| C | 4.258848000 | -6.348744000 | 1.265246000 |
| H | 4.682179000 | -6.705712000 | 2.196805000 |
| C | 4.458825000 | -7.066426000 | 0.083792000 |
| C | 3.915621000 | -6.609342000 | -1.119169000 |
| H | 4.073320000 | -7.168703000 | -2.033948000 |
| C | 3.172903000 | -5.426976000 | -1.120162000 |
| C | 2.524981000 | -4.781323000 | -2.272779000 |
| C | 2.564669000 | -5.302107000 | -3.568895000 |
| H | 3.095465000 | -6.227779000 | -3.758546000 |
| C | 1.924930000 | -4.637301000 | -4.609383000 |
| H | 1.953785000 | -5.039080000 | -5.616708000 |
| C | 1.247436000 | -3.448873000 | -4.329378000 |
| H | 0.731344000 | -2.894206000 | -5.105279000 |
| C | 1.236465000 | -2.970135000 | -3.026586000 |
| H | 0.722812000 | -2.053151000 | -2.765225000 |
| C | -0.440871000 | -4.968853000 | 0.375460000 |
| H | 0.365487000 | -5.692243000 | 0.405214000 |
| C | -1.754638000 | -5.385655000 | 0.394427000 |
| H | -2.005891000 | -6.438398000 | 0.448190000 |
| C | -2.765487000 | -4.413356000 | 0.305894000 |
| C | -2.395819000 | -3.066728000 | 0.249874000 |
| H | -3.149867000 | -2.305278000 | 0.132814000 |
| C | -1.043547000 | -2.691067000 | 0.288342000 |
| C | -1.360763000 | 4.786845000 | -0.460493000 |
| C | -0.524781000 | -1.315797000 | 0.207510000 |
| C | -2.695617000 | 4.813395000 | -0.069915000 |
| C | 0.869790000 | -1.299555000 | -0.018919000 |
| C | -3.366932000 | 3.649729000 | 0.305849000 |
| C | 1.531897000 | -0.086690000 | -0.272309000 |
| C | -4.811444000 | 3.785880000 | 0.654705000 |
| C | -5.772306000 | 2.907514000 | 0.150299000 |
| H | -5.499561000 | 2.081451000 | -0.496520000 |
| C | -7.126621000 | 3.112800000 | 0.458163000 |
| C | -7.466264000 | 4.206706000 | 1.264166000 |

| | | | |
|---|---|---|---|
| H | -8.488751000 | 4.428206000 | 1.542718000 |
| C | -6.430099000 | 5.033159000 | 1.701312000 |
| H | -6.668104000 | 5.895834000 | 2.321881000 |
| C | -2.656657000 | 2.419737000 | 0.353355000 |
| C | 0.831941000 | 1.121589000 | -0.440150000 |
| C | -1.285852000 | 2.373034000 | -0.071782000 |
| C | -0.563441000 | 1.131004000 | -0.061775000 |
| C | -0.638579000 | 3.575331000 | -0.512923000 |
| C | -1.230343000 | -0.083706000 | 0.328193000 |
| C | 0.692008000 | 3.486855000 | -1.035269000 |
| C | -2.547693000 | 0.038817000 | 0.879058000 |
| H | 1.140985000 | 4.360523000 | -1.493246000 |
| H | -3.003400000 | -0.818472000 | 1.357707000 |
| C | 1.391618000 | 2.320345000 | -0.994754000 |
| C | -3.225205000 | 1.222085000 | 0.891475000 |
| H | 2.372671000 | 2.290826000 | -1.451001000 |
| H | -4.206474000 | 1.263951000 | 1.349417000 |
| O | -4.032252000 | -4.868703000 | 0.271452000 |
| O | -8.002256000 | 2.228782000 | -0.066512000 |
| C | -9.407192000 | 2.409663000 | 0.183451000 |
| C | -10.196675000 | 1.296518000 | -0.520914000 |
| C | -11.688849000 | 1.545990000 | -0.226597000 |
| C | -9.781523000 | -0.079286000 | 0.033904000 |
| C | -9.950637000 | 1.352227000 | -2.040411000 |
| C | -5.122966000 | -3.933932000 | 0.182501000 |
| C | -6.448280000 | -4.709566000 | 0.162394000 |
| C | -7.580685000 | -3.667905000 | 0.074418000 |
| C | -6.498084000 | -5.637155000 | -1.066476000 |
| C | -6.599737000 | -5.533345000 | 1.455316000 |
| H | -9.714187000 | 3.394319000 | -0.193060000 |
| H | -9.586758000 | 2.378998000 | 1.266122000 |
| H | -12.014803000 | 2.521148000 | -0.608556000 |
| H | -11.895214000 | 1.516281000 | 0.850243000 |
| H | -12.302883000 | 0.775760000 | -0.706694000 |
| H | -9.959591000 | -0.138909000 | 1.114866000 |
| H | -8.720013000 | -0.277159000 | -0.146737000 |
| H | -10.362058000 | -0.875558000 | -0.446331000 |
| H | -10.251963000 | 2.322772000 | -2.453717000 |
| H | -10.532324000 | 0.575049000 | -2.549682000 |
| H | -8.893381000 | 1.195835000 | -2.277341000 |
| H | -5.084889000 | -3.257370000 | 1.046295000 |
| H | -5.012798000 | -3.339761000 | -0.733986000 |
| H | -8.554999000 | -4.169270000 | 0.058248000 |
| H | -7.500140000 | -3.063210000 | -0.837096000 |
| H | -7.568031000 | -2.988683000 | 0.935588000 |
| H | -6.405474000 | -5.064154000 | -1.997443000 |
| H | -7.450860000 | -6.178183000 | -1.098133000 |
| H | -5.689607000 | -6.374786000 | -1.039698000 |
| H | -6.575748000 | -4.886525000 | 2.341109000 |
| H | -5.797482000 | -6.271875000 | 1.551957000 |
| H | -7.556401000 | -6.068438000 | 1.456073000 |

| | | | |
|---|---|---|---|
| H | -3.223617000 | 5.758814000 | -0.062897000 |
| H | 5.039068000 | -7.982630000 | 0.100369000 |
| N | 2.944990000 | -0.264005000 | -0.374514000 |
| C | 4.010976000 | 0.587912000 | -0.394609000 |
| C | 5.167432000 | -0.168393000 | -0.399585000 |
| C | 4.740678000 | -1.505047000 | -0.359106000 |
| N | 3.404673000 | -1.571249000 | -0.330325000 |
| N | -0.752673000 | 6.022910000 | -0.825118000 |
| C | 0.413354000 | 6.561067000 | -0.352893000 |
| C | 0.535346000 | 7.815230000 | -0.913763000 |
| C | -0.621989000 | 7.958825000 | -1.712425000 |
| N | -1.405480000 | 6.883054000 | -1.656851000 |
| H | 5.320472000 | -2.416253000 | -0.336513000 |
| H | 6.182126000 | 0.199483000 | -0.419950000 |
| H | 3.882333000 | 1.655834000 | -0.368352000 |
| H | 1.035267000 | 6.024421000 | 0.347950000 |
| H | 1.337083000 | 8.523326000 | -0.762712000 |
| H | -0.914143000 | 8.799524000 | -2.329019000 |

Cartesian coordinates of DFT-optimized structure of **6a** complex by B3LYP/Def2-TZVP; 6-31G(d,p)/$CH_3CN$, charge = 1, multiplicity = 1

| | | | |
|---|---|---|---|
| Os | 1.866360000 | -2.493946000 | -0.286779000 |
| N | -0.057794000 | -3.216416000 | 0.181507000 |
| N | 2.479813000 | -2.435807000 | 1.718929000 |
| N | 3.030477000 | -4.171475000 | -0.136969000 |
| N | 1.750788000 | -3.292542000 | -2.223188000 |
| C | 2.147082000 | -1.508532000 | 2.646429000 |
| H | 1.492116000 | -0.718868000 | 2.299556000 |
| C | 2.603540000 | -1.551207000 | 3.956471000 |
| H | 2.300899000 | -0.775983000 | 4.651782000 |
| C | 3.445131000 | -2.594678000 | 4.347605000 |
| H | 3.820809000 | -2.657806000 | 5.363382000 |
| C | 3.794548000 | -3.558258000 | 3.407898000 |
| H | 4.444346000 | -4.379831000 | 3.685920000 |
| C | 3.308967000 | -3.473109000 | 2.100386000 |
| C | 3.626985000 | -4.456287000 | 1.052688000 |
| C | 4.437100000 | -5.587608000 | 1.170537000 |
| H | 4.921108000 | -5.830491000 | 2.109372000 |
| C | 4.618503000 | -6.409148000 | 0.055575000 |
| C | 3.996967000 | -6.099983000 | -1.156516000 |
| H | 4.141544000 | -6.739168000 | -2.019705000 |
| C | 3.194367000 | -4.960015000 | -1.234141000 |
| C | 2.461539000 | -4.462853000 | -2.409455000 |
| C | 2.464205000 | -5.109535000 | -3.648204000 |
| H | 3.027413000 | -6.027024000 | -3.773078000 |
| C | 1.746830000 | -4.579484000 | -4.715085000 |
| H | 1.746711000 | -5.079308000 | -5.677930000 |
| C | 1.030638000 | -3.396713000 | -4.519821000 |
| H | 0.455285000 | -2.943819000 | -5.319768000 |

| | | | |
|---|---|---|---|
| C | 1.057866000 | -2.790662000 | -3.271460000 |
| H | 0.516730000 | -1.873156000 | -3.075288000 |
| C | -0.369813000 | -4.513855000 | 0.407820000 |
| H | 0.469877000 | -5.196240000 | 0.468478000 |
| C | -1.659501000 | -4.980796000 | 0.546606000 |
| H | -1.856265000 | -6.031012000 | 0.727302000 |
| C | -2.719320000 | -4.067539000 | 0.413253000 |
| C | -2.417668000 | -2.721555000 | 0.188991000 |
| H | -3.211670000 | -2.009765000 | 0.030786000 |
| C | -1.084788000 | -2.287074000 | 0.114341000 |
| C | -1.814395000 | 5.065493000 | -1.408818000 |
| C | -0.638128000 | -0.906772000 | -0.133965000 |
| C | -3.146419000 | 5.035408000 | -0.986649000 |
| C | 0.742336000 | -0.851860000 | -0.429787000 |
| C | -3.705752000 | 3.889073000 | -0.437962000 |
| C | 1.331146000 | 0.357034000 | -0.836602000 |
| C | -2.941107000 | 2.711705000 | -0.266437000 |
| C | 0.565658000 | 1.509224000 | -1.091727000 |
| C | -1.593765000 | 2.694265000 | -0.757267000 |
| C | -0.810499000 | 1.494444000 | -0.650747000 |
| C | -1.036045000 | 3.882000000 | -1.342553000 |
| C | -1.397199000 | 0.298406000 | -0.103978000 |
| C | 0.277482000 | 3.792837000 | -1.911305000 |
| C | -2.690543000 | 0.417060000 | 0.502754000 |
| H | 0.663427000 | 4.631696000 | -2.478309000 |
| H | -3.075721000 | -0.404371000 | 1.093014000 |
| C | 1.038186000 | 2.670931000 | -1.787438000 |
| C | -3.428129000 | 1.560953000 | 0.426831000 |
| H | 1.999009000 | 2.641641000 | -2.285043000 |
| H | -4.388105000 | 1.609502000 | 0.927730000 |
| O | -3.963226000 | -4.574659000 | 0.506045000 |
| C | -5.099520000 | -3.700117000 | 0.378711000 |
| C | -6.386456000 | -4.517412000 | 0.564148000 |
| C | -7.568214000 | -3.539269000 | 0.415979000 |
| C | -6.478402000 | -5.611941000 | -0.515894000 |
| C | -6.410769000 | -5.153327000 | 1.967157000 |
| H | -5.031964000 | -2.911752000 | 1.139708000 |
| H | -5.081305000 | -3.232086000 | -0.614136000 |
| H | -8.517712000 | -4.072549000 | 0.538081000 |
| H | -7.574854000 | -3.065510000 | -0.573278000 |
| H | -7.529736000 | -2.746265000 | 1.172775000 |
| H | -6.476426000 | -5.175100000 | -1.522245000 |
| H | -7.405580000 | -6.185087000 | -0.400215000 |
| H | -5.637262000 | -6.309078000 | -0.446284000 |
| H | -6.354344000 | -4.386155000 | 2.749404000 |
| H | -5.571138000 | -5.841956000 | 2.105771000 |
| H | -7.339831000 | -5.715880000 | 2.114990000 |
| H | 5.245416000 | -7.291013000 | 0.131100000 |
| N | 2.745468000 | 0.233918000 | -0.988018000 |
| C | 3.766193000 | 1.126325000 | -1.143949000 |
| C | 4.957383000 | 0.426136000 | -1.126880000 |

| | | | |
|---|---|---|---|
| C | 4.599395000 | -0.917128000 | -0.932134000 |
| N | 3.270648000 | -1.039874000 | -0.834351000 |
| H | 5.224514000 | -1.793924000 | -0.844399000 |
| H | 5.950880000 | 0.835206000 | -1.231791000 |
| H | 3.586127000 | 2.184390000 | -1.219706000 |
| N | -5.070077000 | 3.943888000 | -0.031634000 |
| N | -5.527604000 | 5.016543000 | 0.674117000 |
| C | -6.835817000 | 4.802680000 | 0.803488000 |
| C | -7.242548000 | 3.602383000 | 0.177430000 |
| C | -6.081864000 | 3.080893000 | -0.354519000 |
| C | -1.298280000 | 6.368977000 | -1.922811000 |
| C | -0.068184000 | 6.883935000 | -1.481760000 |
| C | 0.344452000 | 8.139679000 | -1.944858000 |
| C | -0.495947000 | 8.831330000 | -2.831111000 |
| C | -1.698656000 | 8.240299000 | -3.190849000 |
| N | -2.114238000 | 7.039338000 | -2.758071000 |
| O | 1.494556000 | 8.754476000 | -1.595796000 |
| C | 2.396403000 | 8.098836000 | -0.687874000 |
| C | 3.608703000 | 9.007777000 | -0.435920000 |
| C | 4.541830000 | 8.259171000 | 0.535566000 |
| C | 4.348431000 | 9.283563000 | -1.758715000 |
| C | 3.150753000 | 10.333313000 | 0.201635000 |
| H | -7.441240000 | 5.516847000 | 1.347300000 |
| H | -8.235646000 | 3.181608000 | 0.116762000 |
| H | -5.897389000 | 2.195879000 | -0.944845000 |
| H | 0.528195000 | 6.321029000 | -0.776139000 |
| H | -0.205796000 | 9.806072000 | -3.208155000 |
| H | -2.371969000 | 8.761388000 | -3.869304000 |
| H | 2.715884000 | 7.143834000 | -1.125904000 |
| H | 1.872619000 | 7.892270000 | 0.254561000 |
| H | 4.040357000 | 8.043118000 | 1.486902000 |
| H | 5.426722000 | 8.867469000 | 0.754402000 |
| H | 4.885642000 | 7.308915000 | 0.108990000 |
| H | 4.695257000 | 8.350093000 | -2.219137000 |
| H | 5.225787000 | 9.916361000 | -1.581140000 |
| H | 3.699559000 | 9.795636000 | -2.476347000 |
| H | 2.628239000 | 10.155758000 | 1.149925000 |
| H | 2.472787000 | 10.879580000 | -0.461903000 |
| H | 4.014416000 | 10.975597000 | 0.409488000 |
| H | -3.754473000 | 5.926392000 | -1.080844000 |

Cartesian coordinates of DFT-optimized structure of **1b** complex by B3LYP/Def2-TZVP; 6-31G(d,p)/$CH_3CN$, charge = 1, multiplicity = 1

| | | | |
|---|---|---|---|
| Ru | 1.811989000 | -3.049688000 | 0.021685000 |
| N | -1.652242000 | 6.835478000 | -1.648269000 |
| N | -0.160747000 | -3.732504000 | 0.292898000 |
| N | 2.288687000 | -3.235701000 | 2.054316000 |
| N | 2.871344000 | -4.795240000 | 0.055319000 |
| N | 1.786550000 | -3.617314000 | -1.995610000 |

| | | | |
|---|---|---|---|
| N | -5.207200000 | 4.754819000 | 1.513975000 |
| N | 3.354502000 | -1.644481000 | -0.266424000 |
| C | 1.946728000 | -2.391674000 | 3.048666000 |
| H | 1.362647000 | -1.529712000 | 2.748973000 |
| C | 2.309452000 | -2.597436000 | 4.375069000 |
| H | 2.004368000 | -1.881640000 | 5.130491000 |
| C | 3.062079000 | -3.726068000 | 4.700832000 |
| H | 3.363775000 | -3.918555000 | 5.725153000 |
| C | 3.421500000 | -4.607340000 | 3.685557000 |
| H | 4.005238000 | -5.491727000 | 3.913069000 |
| C | 3.029175000 | -4.352529000 | 2.368795000 |
| C | 3.364025000 | -5.239246000 | 1.237120000 |
| C | 4.099840000 | -6.426494000 | 1.283533000 |
| H | 4.502648000 | -6.799904000 | 2.217877000 |
| C | 4.311232000 | -7.132792000 | 0.097200000 |
| C | 3.794999000 | -6.656916000 | -1.110268000 |
| H | 3.963108000 | -7.207729000 | -2.028368000 |
| C | 3.065611000 | -5.464943000 | -1.106727000 |
| C | 2.444572000 | -4.795682000 | -2.266715000 |
| C | 2.504728000 | -5.302326000 | -3.567564000 |
| H | 3.026738000 | -6.232347000 | -3.760922000 |
| C | 1.894115000 | -4.613121000 | -4.610991000 |
| H | 1.937285000 | -5.001781000 | -5.623085000 |
| C | 1.229403000 | -3.419413000 | -4.328982000 |
| H | 0.738010000 | -2.846112000 | -5.107423000 |
| C | 1.200017000 | -2.960581000 | -3.016743000 |
| H | 0.695992000 | -2.039046000 | -2.750964000 |
| C | -1.163229000 | 8.043421000 | -1.970840000 |
| C | -0.542586000 | -5.025609000 | 0.341381000 |
| H | -1.799321000 | 8.661691000 | -2.602157000 |
| H | 0.259742000 | -5.753922000 | 0.381011000 |
| C | 0.070669000 | 8.527256000 | -1.559801000 |
| C | -1.859860000 | -5.439440000 | 0.330152000 |
| H | 0.422684000 | 9.510601000 | -1.852540000 |
| H | -2.117136000 | -6.491498000 | 0.369391000 |
| C | 0.861665000 | 7.711296000 | -0.735966000 |
| C | -2.863545000 | -4.461883000 | 0.227795000 |
| C | 0.367547000 | 6.450436000 | -0.379535000 |
| C | -2.486800000 | -3.115939000 | 0.190638000 |
| H | 0.921251000 | 5.795706000 | 0.280180000 |
| H | -3.233467000 | -2.347711000 | 0.066406000 |
| C | -0.885328000 | 6.046984000 | -0.871069000 |
| C | -1.131821000 | -2.753000000 | 0.262471000 |
| C | -1.475273000 | 4.730890000 | -0.485973000 |
| C | -0.599480000 | -1.381089000 | 0.204133000 |
| C | -2.808083000 | 4.724892000 | -0.070919000 |
| C | 0.797000000 | -1.357424000 | -0.012774000 |
| C | -3.461982000 | 3.564077000 | 0.345208000 |
| C | 1.480884000 | -0.145573000 | -0.256848000 |
| C | -4.897987000 | 3.694398000 | 0.730829000 |
| C | 2.942972000 | -0.320886000 | -0.289993000 |

| | | | |
|---|---|---|---|
| C | -5.869970000 | 2.818221000 | 0.243505000 |
| C | 3.900614000 | 0.693780000 | -0.243580000 |
| H | -5.611427000 | 1.997444000 | -0.415993000 |
| H | 3.609989000 | 1.731646000 | -0.166358000 |
| C | -7.216588000 | 3.018186000 | 0.586030000 |
| C | 5.270325000 | 0.397776000 | -0.250252000 |
| C | -7.538593000 | 4.103409000 | 1.410689000 |
| C | 5.666900000 | -0.945894000 | -0.292875000 |
| H | -8.554242000 | 4.319844000 | 1.716908000 |
| H | 6.704845000 | -1.251044000 | -0.308273000 |
| C | -6.493503000 | 4.927967000 | 1.829583000 |
| C | 4.670114000 | -1.912752000 | -0.282513000 |
| H | -6.717825000 | 5.784167000 | 2.464207000 |
| H | 4.938011000 | -2.963712000 | -0.275861000 |
| C | -2.743968000 | 2.340223000 | 0.388331000 |
| C | 0.738522000 | 1.052651000 | -0.437695000 |
| C | -1.382578000 | 2.302560000 | -0.064602000 |
| C | -0.650124000 | 1.064459000 | -0.054145000 |
| C | -0.755466000 | 3.508606000 | -0.525226000 |
| C | -1.306789000 | -0.154430000 | 0.343321000 |
| C | 0.561312000 | 3.406649000 | -1.080195000 |
| C | -2.617274000 | -0.041434000 | 0.913769000 |
| H | 0.994894000 | 4.270367000 | -1.570806000 |
| H | -3.061915000 | -0.905394000 | 1.391973000 |
| C | 1.270078000 | 2.243827000 | -1.032289000 |
| C | -3.296804000 | 1.140189000 | 0.940826000 |
| H | 2.241502000 | 2.208337000 | -1.509349000 |
| H | -4.270527000 | 1.179279000 | 1.415280000 |
| O | 2.049561000 | 8.218279000 | -0.341031000 |
| O | 6.106418000 | 1.453625000 | -0.204806000 |
| O | -4.132514000 | -4.910085000 | 0.163797000 |
| O | -8.103689000 | 2.137982000 | 0.073637000 |
| C | -9.501845000 | 2.309566000 | 0.364334000 |
| C | -10.306035000 | 1.212613000 | -0.348384000 |
| C | -11.790056000 | 1.438873000 | 0.000487000 |
| C | -9.862671000 | -0.176486000 | 0.148436000 |
| C | -10.109099000 | 1.318781000 | -1.872457000 |
| C | -5.212722000 | -3.969006000 | 0.028578000 |
| C | -6.542985000 | -4.734720000 | -0.026646000 |
| C | -7.661739000 | -3.685628000 | -0.177561000 |
| C | -6.555643000 | -5.685826000 | -1.238333000 |
| C | -6.748923000 | -5.531844000 | 1.275393000 |
| C | 2.907943000 | 7.428167000 | 0.499402000 |
| C | 4.221693000 | 8.188117000 | 0.735907000 |
| C | 4.941841000 | 8.424891000 | -0.605131000 |
| C | 3.938440000 | 9.533453000 | 1.430633000 |
| C | 5.097298000 | 7.306896000 | 1.648085000 |
| C | 7.527716000 | 1.224887000 | -0.182619000 |
| C | 8.253519000 | 2.574790000 | -0.087689000 |
| C | 7.851160000 | 3.300562000 | 1.210173000 |
| C | 7.912174000 | 3.447170000 | -1.310471000 |

| | | | |
|---|---|---|---|
| C | 9.765194000 | 2.274717000 | -0.067758000 |
| H | -9.820978000 | 3.302149000 | 0.020291000 |
| H | -9.652858000 | 2.251891000 | 1.450377000 |
| H | -12.136076000 | 2.423118000 | -0.337955000 |
| H | -11.962293000 | 1.371090000 | 1.081581000 |
| H | -12.412922000 | 0.679656000 | -0.485964000 |
| H | -10.003748000 | -0.272238000 | 1.232251000 |
| H | -8.806311000 | -0.359080000 | -0.073611000 |
| H | -10.452954000 | -0.962072000 | -0.337546000 |
| H | -10.434409000 | 2.298321000 | -2.244474000 |
| H | -10.697579000 | 0.551102000 | -2.388220000 |
| H | -9.058191000 | 1.183616000 | -2.147579000 |
| H | -5.199921000 | -3.281906000 | 0.884848000 |
| H | -5.067057000 | -3.386356000 | -0.890315000 |
| H | -8.639601000 | -4.178625000 | -0.218352000 |
| H | -7.541955000 | -3.102203000 | -1.098592000 |
| H | -7.674058000 | -2.986920000 | 0.667933000 |
| H | -6.419635000 | -5.132591000 | -2.175894000 |
| H | -7.512998000 | -6.216434000 | -1.296979000 |
| H | -5.757693000 | -6.431533000 | -1.164830000 |
| H | -6.755080000 | -4.867484000 | 2.148429000 |
| H | -5.954789000 | -6.271890000 | 1.417287000 |
| H | -7.707635000 | -6.062752000 | 1.250515000 |
| H | 3.107295000 | 6.466308000 | 0.009386000 |
| H | 2.397848000 | 7.234631000 | 1.452457000 |
| H | 5.162518000 | 7.474830000 | -1.107670000 |
| H | 5.891716000 | 8.947180000 | -0.442459000 |
| H | 4.331382000 | 9.032224000 | -1.281073000 |
| H | 3.301233000 | 10.173061000 | 0.811622000 |
| H | 4.875587000 | 10.069274000 | 1.621439000 |
| H | 3.435596000 | 9.382406000 | 2.393907000 |
| H | 5.313936000 | 6.337569000 | 1.182298000 |
| H | 4.608961000 | 7.119366000 | 2.612138000 |
| H | 6.053932000 | 7.802705000 | 1.848348000 |
| H | 7.818780000 | 0.693712000 | -1.098246000 |
| H | 7.775617000 | 0.595307000 | 0.681631000 |
| H | 8.389859000 | 4.251087000 | 1.297726000 |
| H | 8.092026000 | 2.694790000 | 2.092595000 |
| H | 6.777649000 | 3.515014000 | 1.228589000 |
| H | 6.838718000 | 3.656078000 | -1.361019000 |
| H | 8.206655000 | 2.951924000 | -2.244027000 |
| H | 8.442687000 | 4.404919000 | -1.256593000 |
| H | 10.037860000 | 1.647353000 | 0.789700000 |
| H | 10.334971000 | 3.207862000 | 0.006007000 |
| H | 10.083765000 | 1.758697000 | -0.981679000 |
| H | -3.356451000 | 5.659481000 | -0.071504000 |
| H | 4.880183000 | -8.056248000 | 0.113608000 |

Cartesian coordinates of DFT-optimized structure of **2b** complex by B3LYP/Def2-TZVP; 6-31G(d,p)/$CH_3CN$, charge = 1, multiplicity = 1

| | | | |
|---|---|---|---|
| Ru | 1.958137000 | -1.358021000 | -0.018038000 |
| N | 0.459712000 | -2.827121000 | 0.147701000 |
| N | 2.427155000 | -1.430042000 | 2.024022000 |
| N | 3.650721000 | -2.502142000 | -0.006308000 |
| N | 2.221672000 | -1.774984000 | -2.055932000 |
| N | 2.763109000 | 0.580174000 | -0.191838000 |
| C | 1.747615000 | -0.849304000 | 3.033378000 |
| H | 0.865499000 | -0.291350000 | 2.742752000 |
| C | 2.136691000 | -0.949307000 | 4.364513000 |
| H | 1.548239000 | -0.458811000 | 5.132137000 |
| C | 3.281271000 | -1.682132000 | 4.678989000 |
| H | 3.615225000 | -1.781153000 | 5.706577000 |
| C | 3.992784000 | -2.288352000 | 3.647960000 |
| H | 4.885191000 | -2.863178000 | 3.866775000 |
| C | 3.556744000 | -2.155660000 | 2.327047000 |
| C | 4.252256000 | -2.768483000 | 1.178151000 |
| C | 5.407160000 | -3.554690000 | 1.211927000 |
| H | 5.904036000 | -3.780228000 | 2.148265000 |
| C | 5.917031000 | -4.052000000 | 0.010422000 |
| C | 5.281196000 | -3.765083000 | -1.199669000 |
| H | 5.679933000 | -4.153985000 | -2.129361000 |
| C | 4.130075000 | -2.972947000 | -1.182914000 |
| C | 3.318865000 | -2.554650000 | -2.343202000 |
| C | 3.624012000 | -2.909314000 | -3.659867000 |
| H | 4.493832000 | -3.522255000 | -3.865767000 |
| C | 2.811591000 | -2.474529000 | -4.702714000 |
| H | 3.043189000 | -2.746829000 | -5.727171000 |
| C | 1.700279000 | -1.685933000 | -4.404124000 |
| H | 1.036767000 | -1.323081000 | -5.181397000 |
| C | 1.443646000 | -1.361609000 | -3.076561000 |
| H | 0.593095000 | -0.750823000 | -2.798485000 |
| C | 0.660521000 | -4.161136000 | 0.122195000 |
| H | 1.695154000 | -4.484437000 | 0.147936000 |
| C | -0.358329000 | -5.090969000 | 0.057848000 |
| H | -0.146408000 | -6.153683000 | 0.041832000 |
| C | -1.681402000 | -4.625443000 | -0.019456000 |
| C | -1.908313000 | -3.245972000 | 0.011402000 |
| H | -2.909016000 | -2.859740000 | -0.099840000 |
| C | -0.834206000 | -2.350095000 | 0.135025000 |
| C | -4.321307000 | 4.281284000 | -0.297789000 |
| C | -0.932586000 | -0.880056000 | 0.155359000 |
| C | -5.540928000 | 3.708655000 | 0.048116000 |
| C | 0.323929000 | -0.254429000 | -0.016611000 |
| C | -5.612859000 | 2.359783000 | 0.379562000 |
| C | 0.431842000 | 1.145869000 | -0.176884000 |
| C | 1.831855000 | 1.606484000 | -0.168240000 |
| C | 2.270681000 | 2.925853000 | -0.046885000 |
| H | 1.569189000 | 3.739357000 | 0.069701000 |
| C | 3.637812000 | 3.235438000 | -0.030043000 |
| C | 4.564304000 | 2.187976000 | -0.121559000 |

| | | | |
|---|---|---|---|
| H | 5.634058000 | 2.349590000 | -0.121184000 |
| C | 4.069021000 | 0.892126000 | -0.181801000 |
| H | 4.755668000 | 0.053276000 | -0.213591000 |
| C | -4.452825000 | 1.552677000 | 0.421666000 |
| C | -0.746485000 | 1.925368000 | -0.326435000 |
| C | -3.201485000 | 2.126844000 | 0.020448000 |
| C | -2.011924000 | 1.322440000 | 0.007026000 |
| C | -3.140807000 | 3.506541000 | -0.366359000 |
| C | -2.094025000 | -0.079443000 | 0.328727000 |
| C | -1.900441000 | 4.017838000 | -0.863905000 |
| C | -3.334399000 | -0.564373000 | 0.861701000 |
| H | -1.870252000 | 5.013043000 | -1.292270000 |
| H | -3.371664000 | -1.555985000 | 1.294722000 |
| C | -0.765531000 | 3.263956000 | -0.841569000 |
| C | -4.456739000 | 0.207238000 | 0.908068000 |
| H | 0.135593000 | 3.674374000 | -1.279028000 |
| H | -5.361554000 | -0.194764000 | 1.349082000 |
| O | 3.950555000 | 4.540739000 | 0.083385000 |
| O | -2.642162000 | -5.563216000 | -0.126718000 |
| C | -4.022932000 | -5.162901000 | -0.197369000 |
| C | -4.907683000 | -6.416517000 | -0.261377000 |
| C | -6.369430000 | -5.934435000 | -0.340985000 |
| C | -4.564273000 | -7.243488000 | -1.514856000 |
| C | -4.709718000 | -7.267980000 | 1.007080000 |
| C | 5.336412000 | 4.931485000 | 0.107035000 |
| C | 5.427646000 | 6.457498000 | 0.250451000 |
| C | 4.774879000 | 6.903643000 | 1.572548000 |
| C | 4.734630000 | 7.143740000 | -0.941902000 |
| C | 6.925487000 | 6.820695000 | 0.261135000 |
| H | -4.268523000 | -4.565888000 | 0.690477000 |
| H | -4.171052000 | -4.541919000 | -1.090414000 |
| H | -7.048498000 | -6.793480000 | -0.383839000 |
| H | -6.542746000 | -5.325589000 | -1.236714000 |
| H | -6.642466000 | -5.335054000 | 0.536099000 |
| H | -4.711622000 | -6.654843000 | -2.428899000 |
| H | -5.209044000 | -8.127782000 | -1.577372000 |
| H | -3.523689000 | -7.582855000 | -1.493498000 |
| H | -4.957364000 | -6.695241000 | 1.909488000 |
| H | -3.674445000 | -7.612290000 | 1.096710000 |
| H | -5.360356000 | -8.149660000 | 0.979831000 |
| H | 5.815782000 | 4.603248000 | -0.824297000 |
| H | 5.833565000 | 4.436391000 | 0.951104000 |
| H | 4.854587000 | 7.990576000 | 1.689834000 |
| H | 5.267739000 | 6.435433000 | 2.433713000 |
| H | 3.713819000 | 6.635732000 | 1.601506000 |
| H | 3.670589000 | 6.889743000 | -0.982433000 |
| H | 5.192827000 | 6.842193000 | -1.892002000 |
| H | 4.820983000 | 8.233242000 | -0.858135000 |
| H | 7.448633000 | 6.341749000 | 1.097850000 |
| H | 7.051368000 | 7.904411000 | 0.364115000 |
| H | 7.419006000 | 6.513397000 | -0.668970000 |

| | | | |
|---|---|---|---|
| H | -6.437059000 | 4.314882000 | 0.059440000 |
| H | 6.812009000 | -4.664976000 | 0.017160000 |
| N | -4.311589000 | 5.669363000 | -0.610271000 |
| C | -3.486590000 | 6.641742000 | -0.112174000 |
| C | -3.932049000 | 7.845076000 | -0.616559000 |
| C | -5.051004000 | 7.503580000 | -1.410479000 |
| N | -5.288685000 | 6.193547000 | -1.403835000 |
| N | -6.892043000 | 1.828652000 | 0.704926000 |
| N | -7.731351000 | 2.528587000 | 1.520138000 |
| C | -8.856325000 | 1.816678000 | 1.536988000 |
| C | -8.766223000 | 0.660419000 | 0.728330000 |
| C | -7.492028000 | 0.704699000 | 0.203700000 |
| H | -6.978964000 | 0.057158000 | -0.491399000 |
| H | -9.520783000 | -0.091352000 | 0.548463000 |
| H | -9.692999000 | 2.156553000 | 2.134196000 |
| H | -5.689588000 | 8.157972000 | -1.990285000 |
| H | -3.515078000 | 8.824537000 | -0.433067000 |
| H | -2.682038000 | 6.401872000 | 0.566680000 |

Cartesian coordinates of DFT-optimized structure of **3b** complex by B3LYP/Def2-TZVP; 6-31G(d,p)/$CH_3CN$, charge = 1, multiplicity = 1

| | | | |
|---|---|---|---|
| C | 2.334824000 | -2.455716000 | -0.301344000 |
| C | 3.694698000 | -2.454482000 | -0.243992000 |
| C | 4.432833000 | -1.237959000 | -0.113385000 |
| C | 3.707160000 | -0.003167000 | -0.029082000 |
| C | 2.270337000 | -0.001375000 | -0.073893000 |
| C | 1.562801000 | -1.249300000 | -0.248032000 |
| C | 4.426481000 | 1.231189000 | 0.111796000 |
| C | 3.684808000 | 2.425796000 | 0.372823000 |
| C | 2.324486000 | 2.426393000 | 0.368707000 |
| C | 1.562301000 | 1.249027000 | 0.070298000 |
| C | 0.151972000 | -1.190866000 | -0.316031000 |
| C | -0.548279000 | 0.009672000 | -0.160454000 |
| C | 0.155117000 | 1.210354000 | -0.023604000 |
| C | 5.845164000 | -1.201363000 | -0.061062000 |
| C | 6.534550000 | 0.004056000 | -0.011802000 |
| C | 5.838065000 | 1.206249000 | 0.056176000 |
| H | 1.838372000 | -3.412712000 | -0.352317000 |
| H | 4.225834000 | -3.396716000 | -0.300554000 |
| H | 1.816046000 | 3.337094000 | 0.651582000 |
| H | 4.216215000 | 3.337112000 | 0.620300000 |
| N | -0.747174000 | -2.290256000 | -0.510311000 |
| N | -2.090726000 | -2.015276000 | -0.312215000 |
| C | -2.755504000 | -3.133645000 | -0.607829000 |
| C | -1.870073000 | -4.143086000 | -1.020229000 |
| C | -0.613992000 | -3.572950000 | -0.959692000 |
| N | -0.733363000 | 2.330624000 | 0.039883000 |
| N | -2.079557000 | 2.032675000 | 0.165589000 |
| C | -2.729995000 | 3.197388000 | 0.126749000 |

| | | | |
|---|---|---|---|
| C | -1.830956000 | 4.263148000 | -0.046538000 |
| C | -0.581422000 | 3.678960000 | -0.112531000 |
| N | 6.617020000 | -2.399083000 | -0.093367000 |
| N | 7.638258000 | -2.524468000 | -0.987439000 |
| C | 8.203914000 | -3.693271000 | -0.690231000 |
| C | 6.557169000 | -3.464136000 | 0.762857000 |
| C | 7.568476000 | -4.329349000 | 0.400337000 |
| N | 6.597568000 | 2.408164000 | 0.103169000 |
| N | 7.696986000 | 2.486857000 | 0.906037000 |
| C | 8.222277000 | 3.681389000 | 0.641981000 |
| C | 7.482483000 | 4.383182000 | -0.337376000 |
| C | 6.449531000 | 3.531185000 | -0.665806000 |
| C | -1.737266000 | -0.343860000 | 3.003713000 |
| C | -1.975200000 | -0.498561000 | 4.365458000 |
| C | -3.293436000 | -0.550913000 | 4.816557000 |
| C | -4.324526000 | -0.446424000 | 3.887013000 |
| C | -4.027610000 | -0.293789000 | 2.530744000 |
| N | -2.724728000 | -0.242534000 | 2.093184000 |
| C | -5.063219000 | -0.175254000 | 1.483830000 |
| N | -4.547753000 | -0.027026000 | 0.242169000 |
| C | -5.326447000 | 0.102667000 | -0.856767000 |
| C | -6.717995000 | 0.084131000 | -0.728821000 |
| C | -7.272831000 | -0.070273000 | 0.543389000 |
| C | -6.448995000 | -0.201703000 | 1.663195000 |
| C | -4.548387000 | 0.250382000 | -2.104406000 |
| N | -3.180601000 | 0.228013000 | -1.962914000 |
| C | -2.417067000 | 0.351488000 | -3.065769000 |
| C | -2.949733000 | 0.505311000 | -4.341504000 |
| C | -4.335583000 | 0.532612000 | -4.492865000 |
| C | -5.137060000 | 0.402638000 | -3.361961000 |
| H | -0.729122000 | -0.296644000 | 2.609744000 |
| H | -1.137415000 | -0.574865000 | 5.049754000 |
| H | -3.517693000 | -0.669847000 | 5.871423000 |
| H | -5.357238000 | -0.481838000 | 4.213645000 |
| H | -7.361945000 | 0.185916000 | -1.594331000 |
| H | -6.884696000 | -0.321970000 | 2.648039000 |
| H | -1.346780000 | 0.323245000 | -2.899396000 |
| H | -2.283860000 | 0.600815000 | -5.192027000 |
| H | -4.787099000 | 0.651808000 | -5.472214000 |
| H | -6.216622000 | 0.419518000 | -3.455483000 |
| Ru | -2.520415000 | -0.002630000 | 0.014441000 |
| H | -3.832516000 | -3.153524000 | -0.522268000 |
| H | -2.110524000 | -5.148789000 | -1.330264000 |
| H | 0.336465000 | -3.986729000 | -1.243698000 |
| H | -3.807129000 | 3.211927000 | 0.211997000 |
| H | -2.059431000 | 5.315366000 | -0.123952000 |
| H | 0.378254000 | 4.128571000 | -0.295347000 |
| H | 9.047239000 | -4.040696000 | -1.273620000 |
| H | 5.827948000 | -3.504384000 | 1.558454000 |
| H | 7.814329000 | -5.275746000 | 0.859499000 |
| H | 9.112925000 | 4.000021000 | 1.168692000 |

| | | | |
|---|---|---|---|
| H | 7.676655000 | 5.363087000 | -0.748406000 |
| H | 5.649478000 | 3.617022000 | -1.385826000 |
| H | 7.616780000 | 0.007808000 | -0.016558000 |
| H | -8.350885000 | -0.088292000 | 0.662373000 |

Cartesian coordinates of DFT-optimized structure of **4b** complex by B3LYP/Def2-TZVP; 6-31G(d,p)/$CH_3CN$, charge = 1, multiplicity = 1

| | | | |
|---|---|---|---|
| Ru | 3.872531000 | -0.355490000 | -0.005114000 |
| N | -6.128117000 | 2.700728000 | -1.769440000 |
| N | 3.222129000 | -2.270149000 | 0.528422000 |
| N | 4.339971000 | 0.176583000 | 1.971314000 |
| N | 5.901867000 | -0.558808000 | 0.019288000 |
| N | 4.270073000 | -0.975171000 | -1.970660000 |
| N | -6.614810000 | -1.334702000 | 1.448916000 |
| N | 3.631419000 | 1.648006000 | -0.551509000 |
| C | 3.480842000 | 0.548450000 | 2.940122000 |
| H | 2.435412000 | 0.570411000 | 2.656125000 |
| C | 3.889486000 | 0.884571000 | 4.226538000 |
| H | 3.149863000 | 1.176494000 | 4.963918000 |
| C | 5.248462000 | 0.836459000 | 4.535270000 |
| H | 5.604126000 | 1.091362000 | 5.528146000 |
| C | 6.147909000 | 0.453380000 | 3.544212000 |
| H | 7.209070000 | 0.408304000 | 3.759368000 |
| C | 5.681910000 | 0.126613000 | 2.268572000 |
| C | 6.570258000 | -0.289565000 | 1.164236000 |
| C | 7.961081000 | -0.420637000 | 1.205198000 |
| H | 8.517762000 | -0.210165000 | 2.110882000 |
| C | 8.630529000 | -0.830875000 | 0.050300000 |
| C | 7.919365000 | -1.103764000 | -1.120225000 |
| H | 8.443628000 | -1.421370000 | -2.013836000 |
| C | 6.529522000 | -0.957753000 | -1.110997000 |
| C | 5.601835000 | -1.194150000 | -2.236110000 |
| C | 6.022520000 | -1.614021000 | -3.500146000 |
| H | 7.076082000 | -1.782047000 | -3.690829000 |
| C | 5.087772000 | -1.816230000 | -4.511758000 |
| H | 5.408401000 | -2.142390000 | -5.495682000 |
| C | 3.739528000 | -1.592421000 | -4.235033000 |
| H | 2.973592000 | -1.736609000 | -4.989060000 |
| C | 3.376463000 | -1.175172000 | -2.958751000 |
| H | 2.340982000 | -0.990074000 | -2.698532000 |
| C | -6.813590000 | 3.802219000 | -2.115591000 |
| C | 3.735763000 | -3.492067000 | 0.688568000 |
| H | -7.676466000 | 3.643348000 | -2.760248000 |
| C | -6.489484000 | 5.088995000 | -1.710296000 |
| H | -7.076095000 | 5.947612000 | -2.018890000 |
| C | -5.378634000 | 5.259498000 | -0.869304000 |
| C | -4.648991000 | 4.124359000 | -0.495109000 |
| H | -3.801241000 | 4.195450000 | 0.173525000 |
| C | -5.052239000 | 2.867753000 | -0.977050000 |

| | | | |
|---|---|---|---|
| N | 1.846180000 | -2.419663000 | 0.543403000 |
| C | -4.343698000 | 1.621869000 | -0.558511000 |
| C | 1.081304000 | -1.235516000 | 0.305636000 |
| C | -5.130646000 | 0.555339000 | -0.122222000 |
| C | 1.901765000 | -0.157081000 | -0.031097000 |
| C | -4.585893000 | -0.646994000 | 0.331826000 |
| C | 1.322485000 | 1.062592000 | -0.386680000 |
| C | -5.542957000 | -1.724434000 | 0.719197000 |
| N | 2.314124000 | 2.069383000 | -0.602733000 |
| C | -5.379028000 | -3.039820000 | 0.279794000 |
| H | -4.536983000 | -3.328368000 | -0.339059000 |
| C | -6.337010000 | -4.008028000 | 0.618188000 |
| C | -7.440011000 | -3.612184000 | 1.385060000 |
| H | -8.219593000 | -4.302590000 | 1.681295000 |
| C | -7.517565000 | -2.269482000 | 1.757942000 |
| C | 4.383188000 | 2.742148000 | -0.693962000 |
| H | -8.368999000 | -1.933412000 | 2.348087000 |
| C | -3.175324000 | -0.786285000 | 0.401934000 |
| C | -0.074056000 | 1.211912000 | -0.532260000 |
| C | -2.334580000 | 0.271926000 | -0.084468000 |
| C | -0.903816000 | 0.126817000 | -0.066676000 |
| C | -2.932771000 | 1.475920000 | -0.589643000 |
| C | -0.320274000 | -1.101829000 | 0.415629000 |
| C | -2.072345000 | 2.458903000 | -1.177568000 |
| C | -1.190700000 | -2.071237000 | 1.012006000 |
| H | -2.513054000 | 3.309718000 | -1.683715000 |
| H | -0.769469000 | -2.920071000 | 1.534780000 |
| C | -0.716717000 | 2.334656000 | -1.148054000 |
| C | -2.543991000 | -1.920269000 | 1.008248000 |
| H | -0.120563000 | 3.078635000 | -1.660134000 |
| H | -3.158869000 | -2.664300000 | 1.500596000 |
| O | -5.105634000 | 6.521904000 | -0.474721000 |
| O | -6.113660000 | -5.258292000 | 0.158760000 |
| C | -7.074689000 | -6.289347000 | 0.443953000 |
| C | -6.600345000 | -7.609067000 | -0.182054000 |
| C | -7.665788000 | -8.674338000 | 0.142219000 |
| C | -5.247284000 | -8.025918000 | 0.425108000 |
| C | -6.470890000 | -7.453918000 | -1.709175000 |
| C | -3.979469000 | 6.763012000 | 0.385815000 |
| C | -3.886880000 | 8.266341000 | 0.686626000 |
| C | -3.691715000 | 9.057078000 | -0.620939000 |
| C | -5.163178000 | 8.740661000 | 1.407175000 |
| C | -2.665789000 | 8.470959000 | 1.604269000 |
| H | -8.048715000 | -5.996571000 | 0.030217000 |
| H | -7.176360000 | -6.396386000 | 1.531939000 |
| H | -8.642463000 | -8.404842000 | -0.278121000 |
| H | -7.785871000 | -8.805879000 | 1.224462000 |
| H | -7.374516000 | -9.641937000 | -0.282160000 |
| H | -5.325324000 | -8.155878000 | 1.511729000 |
| H | -4.475759000 | -7.274749000 | 0.227797000 |
| H | -4.912923000 | -8.977784000 | -0.003706000 |

| | | | |
|---|---|---|---|
| H | -7.429195000 | -7.165780000 | -2.159005000 |
| H | -6.157760000 | -8.401024000 | -2.163790000 |
| H | -5.730477000 | -6.690668000 | -1.969106000 |
| H | -3.064912000 | 6.414368000 | -0.112083000 |
| H | -4.108082000 | 6.194254000 | 1.316116000 |
| H | -2.779493000 | 8.739997000 | -1.141535000 |
| H | -3.602269000 | 10.128834000 | -0.408922000 |
| H | -4.537588000 | 8.914535000 | -1.301023000 |
| H | -6.050254000 | 8.588433000 | 0.783993000 |
| H | -5.093332000 | 9.808562000 | 1.644651000 |
| H | -5.311074000 | 8.196223000 | 2.348180000 |
| H | -1.739090000 | 8.143660000 | 1.117128000 |
| H | -2.771802000 | 7.913583000 | 2.543049000 |
| H | -2.554924000 | 9.531812000 | 1.855948000 |
| H | -6.208705000 | 0.664352000 | -0.135790000 |
| H | 9.709903000 | -0.938232000 | 0.062405000 |
| C | 2.276340000 | 3.425927000 | -0.744864000 |
| C | 3.576983000 | 3.886052000 | -0.819838000 |
| C | 1.534240000 | -3.740822000 | 0.680848000 |
| C | 2.714130000 | -4.451441000 | 0.789795000 |
| H | 4.808317000 | -3.622198000 | 0.710831000 |
| H | 5.460315000 | 2.655397000 | -0.685682000 |
| H | 1.348731000 | 3.970995000 | -0.748950000 |
| H | 3.896659000 | 4.910160000 | -0.939999000 |
| H | 0.516664000 | -4.089387000 | 0.658037000 |
| H | 2.819278000 | -5.518455000 | 0.915737000 |

Cartesian coordinates of DFT-optimized structure of **5b** complex by B3LYP/Def2-TZVP; 6-31G(d,p)/$CH_3CN$, charge = 1, multiplicity = 1

| | | | |
|---|---|---|---|
| Ru | 3.561205000 | 0.039823000 | 0.007787000 |
| N | 2.355658000 | -1.664576000 | 0.263702000 |
| N | 4.085576000 | 0.133072000 | 2.037355000 |
| N | 5.443663000 | -0.750869000 | -0.001666000 |
| N | 3.837255000 | -0.409492000 | -2.022902000 |
| N | -6.696511000 | 2.307218000 | 1.598039000 |
| C | 3.328679000 | 0.593918000 | 3.052778000 |
| H | 2.344644000 | 0.953485000 | 2.776638000 |
| C | 3.763921000 | 0.613839000 | 4.373506000 |
| H | 3.108291000 | 0.997897000 | 5.147251000 |
| C | 5.040513000 | 0.136419000 | 4.669263000 |
| H | 5.413804000 | 0.136345000 | 5.688016000 |
| C | 5.833300000 | -0.344083000 | 3.631050000 |
| H | 6.828545000 | -0.721435000 | 3.835548000 |
| C | 5.345554000 | -0.340945000 | 2.321888000 |
| C | 6.121414000 | -0.836859000 | 1.167196000 |
| C | 7.419028000 | -1.355752000 | 1.183195000 |
| H | 7.980305000 | -1.431959000 | 2.107031000 |
| C | 7.987940000 | -1.778395000 | -0.020186000 |
| C | 7.270148000 | -1.680817000 | -1.214328000 |

| | | | |
|---|---|---|---|
| H | 7.716633000 | -2.009327000 | -2.145564000 |
| C | 5.976530000 | -1.153416000 | -1.179442000 |
| C | 5.060291000 | -0.964273000 | -2.322117000 |
| C | 5.387437000 | -1.317160000 | -3.633673000 |
| H | 6.355978000 | -1.752739000 | -3.849962000 |
| C | 4.468687000 | -1.110347000 | -4.658661000 |
| H | 4.716872000 | -1.382519000 | -5.679255000 |
| C | 3.229845000 | -0.550492000 | -4.347280000 |
| H | 2.480200000 | -0.371237000 | -5.110111000 |
| C | 2.956832000 | -0.217030000 | -3.024966000 |
| H | 2.007807000 | 0.219666000 | -2.737380000 |
| C | 2.801794000 | -2.937753000 | 0.277744000 |
| H | 3.878892000 | -3.059742000 | 0.301446000 |
| C | 1.974439000 | -4.042718000 | 0.250140000 |
| H | 2.380672000 | -5.047353000 | 0.261201000 |
| C | 0.587869000 | -3.834173000 | 0.170294000 |
| C | 0.106368000 | -2.520878000 | 0.169549000 |
| H | -0.949788000 | -2.332974000 | 0.061409000 |
| C | 0.993728000 | -1.436570000 | 0.253590000 |
| C | -3.594851000 | 4.504970000 | -0.231609000 |
| C | 0.622364000 | -0.008932000 | 0.231003000 |
| C | -4.695242000 | 3.740219000 | 0.141024000 |
| C | 1.745037000 | 0.817509000 | 0.025729000 |
| C | -4.567059000 | 2.389437000 | 0.465745000 |
| C | 1.584588000 | 2.195132000 | -0.173405000 |
| C | -5.821305000 | 1.652905000 | 0.799285000 |
| C | -6.103041000 | 0.400686000 | 0.249989000 |
| H | -5.409618000 | -0.090937000 | -0.422784000 |
| C | -7.323926000 | -0.225869000 | 0.546554000 |
| C | -8.225110000 | 0.439528000 | 1.387239000 |
| H | -9.184833000 | 0.018984000 | 1.659655000 |
| C | -7.852605000 | 1.695511000 | 1.868383000 |
| H | -8.539127000 | 2.238082000 | 2.516651000 |
| C | -3.276192000 | 1.795598000 | 0.479427000 |
| C | 0.312482000 | 2.783410000 | -0.309532000 |
| C | -2.135179000 | 2.566422000 | 0.072461000 |
| C | -0.827330000 | 1.971453000 | 0.049014000 |
| C | -2.305371000 | 3.936883000 | -0.316702000 |
| C | -0.665592000 | 0.581455000 | 0.385695000 |
| C | -1.173097000 | 4.653135000 | -0.822638000 |
| C | -1.806024000 | -0.102532000 | 0.919870000 |
| H | -1.315713000 | 5.642052000 | -1.242529000 |
| H | -1.677451000 | -1.084098000 | 1.358010000 |
| C | 0.072488000 | 4.104581000 | -0.813226000 |
| C | -3.042751000 | 0.469818000 | 0.965283000 |
| H | 0.887202000 | 4.664271000 | -1.254543000 |
| H | -3.863800000 | -0.080877000 | 1.409055000 |
| O | -0.180381000 | -4.937189000 | 0.090085000 |
| O | -7.533058000 | -1.432688000 | -0.022088000 |
| C | -8.781538000 | -2.106411000 | 0.215329000 |
| C | -8.790007000 | -3.443384000 | -0.540327000 |

| | | | |
|---|---|---|---|
| C | -10.148806000 | -4.113248000 | -0.256891000 |
| C | -7.650365000 | -4.346314000 | -0.031440000 |
| C | -8.637428000 | -3.198018000 | -2.053275000 |
| C | -1.610915000 | -4.804528000 | 0.003412000 |
| C | -2.240687000 | -6.202828000 | -0.077786000 |
| C | -3.767231000 | -6.007300000 | -0.159642000 |
| C | -1.741529000 | -6.933863000 | -1.338521000 |
| C | -1.888226000 | -7.016712000 | 1.181798000 |
| H | -9.604949000 | -1.466707000 | -0.128665000 |
| H | -8.899162000 | -2.276260000 | 1.293606000 |
| H | -10.982311000 | -3.491934000 | -0.606611000 |
| H | -10.288649000 | -4.297715000 | 0.815238000 |
| H | -10.210042000 | -5.077723000 | -0.773449000 |
| H | -7.749694000 | -4.538683000 | 1.044137000 |
| H | -6.671629000 | -3.887577000 | -0.205334000 |
| H | -7.669227000 | -5.312786000 | -0.548425000 |
| H | -9.448482000 | -2.565064000 | -2.434250000 |
| H | -8.668434000 | -4.148347000 | -2.598845000 |
| H | -7.686724000 | -2.705703000 | -2.281721000 |
| H | -1.974498000 | -4.268657000 | 0.889916000 |
| H | -1.863766000 | -4.218149000 | -0.889493000 |
| H | -4.270127000 | -6.979179000 | -0.218489000 |
| H | -4.050996000 | -5.429160000 | -1.047474000 |
| H | -4.152112000 | -5.484247000 | 0.724343000 |
| H | -1.997535000 | -6.374077000 | -2.246658000 |
| H | -2.203379000 | -7.925133000 | -1.413251000 |
| H | -0.655067000 | -7.066379000 | -1.316102000 |
| H | -2.244442000 | -6.514086000 | 2.089572000 |
| H | -0.806541000 | -7.157782000 | 1.273665000 |
| H | -2.357847000 | -8.006261000 | 1.139587000 |
| H | -5.672626000 | 4.205306000 | 0.174417000 |
| H | 8.993916000 | -2.184190000 | -0.027677000 |
| N | 2.837979000 | 2.874589000 | -0.257580000 |
| C | 3.210631000 | 4.186598000 | -0.213869000 |
| C | 4.591305000 | 4.239948000 | -0.227773000 |
| C | 5.014059000 | 2.899981000 | -0.258403000 |
| N | 3.964713000 | 2.074265000 | -0.263497000 |
| N | -3.816983000 | 5.877403000 | -0.544027000 |
| C | -3.173061000 | 6.972947000 | -0.036713000 |
| C | -3.803706000 | 8.086237000 | -0.551764000 |
| C | -4.837573000 | 7.563347000 | -1.361452000 |
| N | -4.854015000 | 6.231454000 | -1.354567000 |
| H | -2.349439000 | 6.869457000 | 0.653864000 |
| H | -3.557972000 | 9.121425000 | -0.364680000 |
| H | -5.567341000 | 8.102203000 | -1.952540000 |
| H | 6.014868000 | 2.492541000 | -0.264658000 |
| H | 5.206499000 | 5.126882000 | -0.208855000 |
| H | 2.486831000 | 4.979813000 | -0.142617000 |

Cartesian coordinates of DFT-optimized structure of **6b** complex by B3LYP/Def2-TZVP; 6-31G(d,p)/$CH_3CN$, charge = 1, multiplicity = 1

| | | | |
|---|---|---|---|
| Ru | -2.701375000 | -1.586957000 | -0.041974000 |
| N | -3.359705000 | 0.385480000 | 0.286522000 |
| N | -2.834636000 | -2.107424000 | 1.985878000 |
| N | -4.443044000 | -2.650419000 | 0.011433000 |
| N | -3.321972000 | -1.509563000 | -2.044308000 |
| C | -1.969629000 | -1.783178000 | 2.967190000 |
| H | -1.118294000 | -1.186843000 | 2.661398000 |
| C | -2.141769000 | -2.177854000 | 4.289559000 |
| H | -1.409720000 | -1.886338000 | 5.034624000 |
| C | -3.258678000 | -2.943437000 | 4.623983000 |
| H | -3.425786000 | -3.269699000 | 5.645192000 |
| C | -4.162210000 | -3.283787000 | 3.621488000 |
| H | -5.038911000 | -3.875930000 | 3.856594000 |
| C | -3.939912000 | -2.860366000 | 2.308904000 |
| C | -4.851594000 | -3.175449000 | 1.190568000 |
| C | -6.029538000 | -3.925428000 | 1.247836000 |
| H | -6.374927000 | -4.355986000 | 2.180502000 |
| C | -6.762375000 | -4.115189000 | 0.074150000 |
| C | -6.321776000 | -3.564388000 | -1.131343000 |
| H | -6.893369000 | -3.715638000 | -2.039565000 |
| C | -5.137534000 | -2.822469000 | -1.137976000 |
| C | -4.503532000 | -2.166569000 | -2.299498000 |
| C | -5.045389000 | -2.196460000 | -3.586847000 |
| H | -5.977297000 | -2.719097000 | -3.768528000 |
| C | -4.387518000 | -1.555244000 | -4.632532000 |
| H | -4.803258000 | -1.574759000 | -5.634529000 |
| C | -3.190078000 | -0.891475000 | -4.366235000 |
| H | -2.640553000 | -0.377287000 | -5.147052000 |
| C | -2.695867000 | -0.893258000 | -3.066273000 |
| H | -1.769694000 | -0.390901000 | -2.813192000 |
| C | -4.648984000 | 0.772768000 | 0.378048000 |
| H | -5.380189000 | -0.026553000 | 0.424604000 |
| C | -5.056136000 | 2.092039000 | 0.401671000 |
| H | -6.105323000 | 2.352956000 | 0.476407000 |
| C | -4.077435000 | 3.093062000 | 0.289804000 |
| C | -2.734713000 | 2.710407000 | 0.204720000 |
| H | -1.968223000 | 3.456193000 | 0.067729000 |
| C | -2.376345000 | 1.353983000 | 0.243647000 |
| C | 5.118369000 | 1.592724000 | -0.609783000 |
| C | -1.005983000 | 0.817141000 | 0.141453000 |
| C | 5.130199000 | 2.943566000 | -0.252315000 |
| C | -1.006439000 | -0.572909000 | -0.088997000 |
| C | 3.973410000 | 3.594403000 | 0.156264000 |
| C | 0.192174000 | -1.247413000 | -0.357303000 |
| C | 2.740607000 | 2.907390000 | 0.247268000 |
| C | 1.408631000 | -0.560899000 | -0.537053000 |
| C | 2.687257000 | 1.540791000 | -0.183570000 |
| C | 1.437354000 | 0.832159000 | -0.157759000 |

| | | | |
|---|---|---|---|
| C | 3.887653000 | 0.888041000 | -0.626958000 |
| C | 0.234235000 | 1.510510000 | 0.249103000 |
| C | 3.771269000 | -0.443427000 | -1.146650000 |
| C | 0.373392000 | 2.824615000 | 0.804634000 |
| H | 4.634036000 | -0.903008000 | -1.614105000 |
| H | -0.475200000 | 3.284710000 | 1.294399000 |
| C | 2.597680000 | -1.131617000 | -1.099527000 |
| C | 1.561591000 | 3.492735000 | 0.804361000 |
| H | 2.555108000 | -2.111929000 | -1.556696000 |
| H | 1.621344000 | 4.473164000 | 1.262601000 |
| O | -4.519197000 | 4.364934000 | 0.265407000 |
| C | -3.574003000 | 5.444101000 | 0.146099000 |
| C | -4.329194000 | 6.780947000 | 0.174746000 |
| C | -3.275397000 | 7.897637000 | 0.041293000 |
| C | -5.318182000 | 6.855423000 | -1.004036000 |
| C | -5.082579000 | 6.934012000 | 1.509772000 |
| H | -2.860047000 | 5.388792000 | 0.978150000 |
| H | -3.023195000 | 5.334803000 | -0.797170000 |
| H | -3.761415000 | 8.879681000 | 0.055438000 |
| H | -2.718434000 | 7.812940000 | -0.899842000 |
| H | -2.554301000 | 7.868661000 | 0.867223000 |
| H | -4.796108000 | 6.760534000 | -1.964290000 |
| H | -5.843632000 | 7.817434000 | -1.001038000 |
| H | -6.067351000 | 6.059376000 | -0.944505000 |
| H | -4.390813000 | 6.893215000 | 2.360312000 |
| H | -5.826862000 | 6.141721000 | 1.639143000 |
| H | -5.602688000 | 7.898204000 | 1.545855000 |
| H | -7.679013000 | -4.694767000 | 0.098812000 |
| N | 0.001600000 | -2.659365000 | -0.457415000 |
| C | 0.844676000 | -3.732340000 | -0.474991000 |
| C | 0.076047000 | -4.880624000 | -0.473574000 |
| C | -1.256271000 | -4.435335000 | -0.431741000 |
| N | -1.307069000 | -3.101221000 | -0.408955000 |
| H | -2.173477000 | -5.006160000 | -0.404039000 |
| H | 0.432227000 | -5.899607000 | -0.491087000 |
| H | 1.914033000 | -3.614247000 | -0.453244000 |
| N | 4.073255000 | 4.972221000 | 0.504194000 |
| N | 5.099157000 | 5.403187000 | 1.291416000 |
| C | 4.955224000 | 6.726407000 | 1.339254000 |
| C | 3.848296000 | 7.168473000 | 0.579381000 |
| C | 3.309741000 | 6.013760000 | 0.051475000 |
| C | 6.430409000 | 0.975124000 | -0.965729000 |
| C | 6.818680000 | -0.259170000 | -0.419248000 |
| C | 8.084557000 | -0.769533000 | -0.733165000 |
| C | 8.912907000 | -0.019045000 | -1.582145000 |
| C | 8.439347000 | 1.196771000 | -2.054144000 |
| N | 7.231093000 | 1.705431000 | -1.764635000 |
| O | 8.586439000 | -1.934571000 | -0.271356000 |
| C | 7.789418000 | -2.746333000 | 0.608085000 |
| C | 8.587539000 | -3.999008000 | 0.999532000 |
| C | 7.691579000 | -4.833227000 | 1.935654000 |

| | | | |
|---|---|---|---|
| C | 8.933284000 | -4.820291000 | -0.257088000 |
| C | 9.876523000 | -3.593743000 | 1.739497000 |
| H | 5.649783000 | 7.317088000 | 1.923191000 |
| H | 3.497622000 | 8.179572000 | 0.432138000 |
| H | 2.476186000 | 5.850003000 | -0.614962000 |
| H | 6.151297000 | -0.783344000 | 0.251802000 |
| H | 9.899169000 | -0.386167000 | -1.844735000 |
| H | 9.067258000 | 1.801744000 | -2.706019000 |
| H | 6.860316000 | -3.027384000 | 0.094611000 |
| H | 7.531368000 | -2.163296000 | 1.501786000 |
| H | 7.423783000 | -4.272256000 | 2.839420000 |
| H | 8.215896000 | -5.742863000 | 2.249713000 |
| H | 6.763280000 | -5.136988000 | 1.436508000 |
| H | 8.024603000 | -5.130605000 | -0.787842000 |
| H | 9.486984000 | -5.725515000 | 0.017993000 |
| H | 9.551133000 | -4.241726000 | -0.951196000 |
| H | 9.647828000 | -3.015552000 | 2.643470000 |
| H | 10.525298000 | -2.984345000 | 1.102288000 |
| H | 10.438250000 | -4.484449000 | 2.044092000 |
| H | 6.062014000 | 3.494173000 | -0.285115000 |

Cartesian coordinates of DFT-optimized structure of **1aox** complex by B3LYP/Def2-TZVP; 6-31G(d,p)/$CH_3CN$, charge = 2, multiplicity = 2

| | | | |
|---|---|---|---|
| Os | 2.191900000 | -0.046596000 | 0.031683000 |
| N | -7.886651000 | 2.597263000 | -1.795872000 |
| N | 1.675118000 | -2.076997000 | 0.312582000 |
| N | 2.651903000 | 0.205856000 | 2.068977000 |
| N | 4.297253000 | -0.132922000 | 0.094845000 |
| N | 2.745932000 | -0.439111000 | -1.958566000 |
| N | -8.195490000 | -1.469563000 | 1.400422000 |
| N | 1.917681000 | 2.039303000 | -0.283978000 |
| C | 1.731624000 | 0.368597000 | 3.045442000 |
| H | 0.695952000 | 0.334499000 | 2.733587000 |
| C | 2.083138000 | 0.570233000 | 4.373971000 |
| H | 1.302491000 | 0.698076000 | 5.114905000 |
| C | 3.432940000 | 0.599164000 | 4.721028000 |
| H | 3.740087000 | 0.752879000 | 5.749749000 |
| C | 4.387377000 | 0.423716000 | 3.723191000 |
| H | 5.442358000 | 0.438372000 | 3.968127000 |
| C | 3.986147000 | 0.228563000 | 2.402098000 |
| C | 4.923060000 | 0.027059000 | 1.283735000 |
| C | 6.316961000 | -0.020171000 | 1.351033000 |
| H | 6.835679000 | 0.103345000 | 2.293901000 |
| C | 7.037392000 | -0.235168000 | 0.175072000 |
| C | 6.372654000 | -0.406214000 | -1.039885000 |
| H | 6.934659000 | -0.580641000 | -1.949185000 |
| C | 4.977266000 | -0.351639000 | -1.054275000 |
| C | 4.093840000 | -0.521827000 | -2.219786000 |
| C | 4.555792000 | -0.763256000 | -3.513403000 |

| | | | |
|---|---|---|---|
| H | 5.620801000 | -0.824352000 | -3.701441000 |
| C | 3.648968000 | -0.925434000 | -4.556535000 |
| H | 4.003145000 | -1.112728000 | -5.564329000 |
| C | 2.284784000 | -0.844621000 | -4.280821000 |
| H | 1.539327000 | -0.965426000 | -5.058334000 |
| C | 1.872216000 | -0.603689000 | -2.976785000 |
| H | 0.823227000 | -0.531344000 | -2.720997000 |
| C | -8.614619000 | 3.677713000 | -2.120032000 |
| C | 2.534701000 | -3.116007000 | 0.379736000 |
| H | -9.475363000 | 3.497487000 | -2.761543000 |
| H | 3.587047000 | -2.861269000 | 0.416238000 |
| C | -8.335959000 | 4.968605000 | -1.693578000 |
| C | 2.134510000 | -4.436017000 | 0.394394000 |
| H | -8.958037000 | 5.809034000 | -1.981898000 |
| H | 2.861279000 | -5.237452000 | 0.451011000 |
| C | -7.226480000 | 5.167797000 | -0.857334000 |
| C | 0.762919000 | -4.722467000 | 0.300155000 |
| C | -6.449998000 | 4.054883000 | -0.509500000 |
| C | -0.142046000 | -3.653425000 | 0.236272000 |
| H | -5.600397000 | 4.147021000 | 0.153901000 |
| H | -1.196456000 | -3.842581000 | 0.114257000 |
| C | -6.811995000 | 2.793864000 | -1.009905000 |
| C | 0.318037000 | -2.333166000 | 0.281184000 |
| C | -6.057031000 | 1.568019000 | -0.614470000 |
| C | -0.527018000 | -1.124221000 | 0.194726000 |
| C | -6.803713000 | 0.465151000 | -0.194432000 |
| C | 0.220534000 | 0.054536000 | -0.035331000 |
| C | -6.215757000 | -0.721059000 | 0.246541000 |
| C | -0.380970000 | 1.303384000 | -0.313001000 |
| C | -7.133694000 | -1.829518000 | 0.642640000 |
| C | 0.585530000 | 2.425412000 | -0.337534000 |
| C | -6.944322000 | -3.136019000 | 0.189536000 |
| C | 0.265205000 | 3.776140000 | -0.307638000 |
| H | -6.111690000 | -3.398655000 | -0.453310000 |
| H | -0.761803000 | 4.107323000 | -0.257008000 |
| C | -7.870180000 | -4.130601000 | 0.545107000 |
| C | 1.271644000 | 4.760397000 | -0.299253000 |
| C | -8.960957000 | -3.766949000 | 1.344553000 |
| C | 2.610827000 | 4.350088000 | -0.307939000 |
| H | -9.714001000 | -4.478959000 | 1.657631000 |
| H | 3.437957000 | 5.046927000 | -0.310226000 |
| C | -9.064712000 | -2.429084000 | 1.728280000 |
| C | 2.869648000 | 2.987126000 | -0.283442000 |
| H | -9.908614000 | -2.118796000 | 2.342479000 |
| H | 3.893129000 | 2.632482000 | -0.254671000 |
| C | -4.800741000 | -0.808569000 | 0.312438000 |
| C | -1.780695000 | 1.347487000 | -0.523886000 |
| C | -4.002186000 | 0.289318000 | -0.152489000 |
| C | -2.568075000 | 0.203772000 | -0.122022000 |
| C | -4.643005000 | 1.476404000 | -0.641252000 |
| C | -1.938102000 | -1.017823000 | 0.317442000 |

| | | | |
|---|---|---|---|
| C | -3.819460000 | 2.501742000 | -1.209885000 |
| C | -2.769993000 | -2.028420000 | 0.898803000 |
| H | -4.291533000 | 3.332932000 | -1.720240000 |
| H | -2.310461000 | -2.867143000 | 1.405612000 |
| C | -2.460816000 | 2.443537000 | -1.148082000 |
| C | -4.128677000 | -1.925841000 | 0.902632000 |
| H | -1.881664000 | 3.216832000 | -1.635889000 |
| H | -4.715891000 | -2.696876000 | 1.386845000 |
| O | -6.999372000 | 6.430742000 | -0.439844000 |
| O | 0.849404000 | 6.030168000 | -0.275454000 |
| O | 0.422445000 | -6.018099000 | 0.271025000 |
| O | -7.630449000 | -5.370619000 | 0.070028000 |
| C | -8.558856000 | -6.426833000 | 0.375928000 |
| C | -8.080577000 | -7.727843000 | -0.284688000 |
| C | -9.107972000 | -8.820289000 | 0.070833000 |
| C | -6.695198000 | -8.120621000 | 0.262362000 |
| C | -8.017013000 | -7.552340000 | -1.813749000 |
| C | -0.965791000 | -6.396214000 | 0.160794000 |
| C | -1.069224000 | -7.927818000 | 0.180635000 |
| C | -2.564279000 | -8.279174000 | 0.051866000 |
| C | -0.290071000 | -8.526582000 | -1.005752000 |
| C | -0.518000000 | -8.477575000 | 1.510074000 |
| C | -5.879074000 | 6.697062000 | 0.421973000 |
| C | -5.858494000 | 8.191373000 | 0.776482000 |
| C | -5.715075000 | 9.038188000 | -0.502331000 |
| C | -7.150891000 | 8.573363000 | 1.523018000 |
| C | -4.641369000 | 8.422667000 | 1.693051000 |
| C | 1.819046000 | 7.101022000 | -0.245314000 |
| C | 1.080984000 | 8.446171000 | -0.206413000 |
| C | 0.207573000 | 8.534409000 | 1.059545000 |
| C | 0.209000000 | 8.609196000 | -1.465848000 |
| C | 2.158440000 | 9.548054000 | -0.173014000 |
| H | -9.553044000 | -6.149748000 | 0.001664000 |
| H | -8.617662000 | -6.548768000 | 1.465457000 |
| H | -10.107828000 | -8.567789000 | -0.302804000 |
| H | -9.177500000 | -8.967018000 | 1.155594000 |
| H | -8.814431000 | -9.775889000 | -0.378151000 |
| H | -6.724709000 | -8.259226000 | 1.350283000 |
| H | -5.947794000 | -7.352696000 | 0.038234000 |
| H | -6.360223000 | -9.062526000 | -0.187264000 |
| H | -8.999630000 | -7.284203000 | -2.221474000 |
| H | -7.697932000 | -8.485511000 | -2.292317000 |
| H | -7.308101000 | -6.766648000 | -2.094081000 |
| H | -1.520382000 | -5.960477000 | 1.001368000 |
| H | -1.368929000 | -5.993617000 | -0.776901000 |
| H | -2.697997000 | -9.366666000 | 0.061464000 |
| H | -2.986368000 | -7.897893000 | -0.885887000 |
| H | -3.147512000 | -7.862532000 | 0.881988000 |
| H | -0.677316000 | -8.152939000 | -1.961725000 |
| H | -0.382711000 | -9.618663000 | -1.009388000 |
| H | 0.774205000 | -8.276655000 | -0.948610000 |

| | | | |
|---|---|---|---|
| H | -1.068661000 | -8.067418000 | 2.365665000 |
| H | 0.540440000 | -8.227627000 | 1.635624000 |
| H | -0.614959000 | -9.568863000 | 1.540107000 |
| H | -4.953296000 | 6.410486000 | -0.094524000 |
| H | -5.972766000 | 6.091001000 | 1.332448000 |
| H | -4.790281000 | 8.790762000 | -1.038216000 |
| H | -5.682838000 | 10.105063000 | -0.252388000 |
| H | -6.556568000 | 8.873991000 | -1.182897000 |
| H | -8.034176000 | 8.404872000 | 0.898738000 |
| H | -7.130192000 | 9.632426000 | 1.804970000 |
| H | -7.265823000 | 7.983492000 | 2.440979000 |
| H | -3.703098000 | 8.161991000 | 1.187924000 |
| H | -4.710977000 | 7.825348000 | 2.610409000 |
| H | -4.582478000 | 9.477428000 | 1.984447000 |
| H | 2.449785000 | 7.030311000 | -1.140000000 |
| H | 2.449775000 | 6.979225000 | 0.643987000 |
| H | -0.295676000 | 9.506824000 | 1.106620000 |
| H | 0.814285000 | 8.425176000 | 1.966959000 |
| H | -0.560820000 | 7.754310000 | 1.068329000 |
| H | -0.554172000 | 7.826454000 | -1.525088000 |
| H | 0.817525000 | 8.561597000 | -2.377351000 |
| H | -0.300322000 | 9.579582000 | -1.452362000 |
| H | 2.797998000 | 9.457721000 | 0.713339000 |
| H | 1.685051000 | 10.535800000 | -0.145212000 |
| H | 2.799976000 | 9.509145000 | -1.061659000 |
| H | -7.884769000 | 0.536058000 | -0.206819000 |
| H | 8.120607000 | -0.274600000 | 0.206616000 |

Cartesian coordinates of DFT-optimized structure of **2aox** complex by B3LYP/Def2-TZVP; 6-31G(d,p)/$CH_3CN$, charge = 2, multiplicity = 2

| | | | |
|---|---|---|---|
| Os | 2.165957000 | -0.971767000 | 0.012002000 |
| N | 0.967734000 | -2.698114000 | 0.209981000 |
| N | 2.712098000 | -1.057154000 | 2.041885000 |
| N | 4.116145000 | -1.762367000 | -0.021330000 |
| N | 2.489032000 | -1.439216000 | -2.013042000 |
| N | 2.638744000 | 1.092842000 | -0.192092000 |
| C | 1.909142000 | -0.694685000 | 3.066565000 |
| H | 0.910073000 | -0.376101000 | 2.798957000 |
| C | 2.332769000 | -0.721115000 | 4.389080000 |
| H | 1.648063000 | -0.415089000 | 5.171502000 |
| C | 3.628852000 | -1.146279000 | 4.676447000 |
| H | 3.988756000 | -1.180976000 | 5.698945000 |
| C | 4.458984000 | -1.533302000 | 3.627914000 |
| H | 5.467733000 | -1.874194000 | 3.826965000 |
| C | 3.990732000 | -1.484678000 | 2.315571000 |
| C | 4.788791000 | -1.890317000 | 1.145510000 |
| C | 6.096848000 | -2.378463000 | 1.146735000 |
| H | 6.650773000 | -2.489837000 | 2.070855000 |
| C | 6.681954000 | -2.729528000 | -0.071148000 |

| | | | |
|---|---|---|---|
| C | 5.965757000 | -2.603473000 | -1.262090000 |
| H | 6.418521000 | -2.888547000 | -2.203929000 |
| C | 4.659573000 | -2.111243000 | -1.209776000 |
| C | 3.733832000 | -1.925926000 | -2.339593000 |
| C | 4.054727000 | -2.231511000 | -3.661741000 |
| H | 5.039300000 | -2.613884000 | -3.901629000 |
| C | 3.109797000 | -2.046585000 | -4.666804000 |
| H | 3.354956000 | -2.281586000 | -5.696821000 |
| C | 1.848790000 | -1.560381000 | -4.324820000 |
| H | 1.078538000 | -1.402612000 | -5.070819000 |
| C | 1.574454000 | -1.272842000 | -2.994063000 |
| H | 0.609674000 | -0.891396000 | -2.686078000 |
| C | 1.409887000 | -3.975075000 | 0.207109000 |
| H | 2.485168000 | -4.106042000 | 0.231438000 |
| C | 0.573033000 | -5.070360000 | 0.167450000 |
| H | 0.973879000 | -6.076890000 | 0.167132000 |
| C | -0.813458000 | -4.854716000 | 0.093247000 |
| C | -1.287137000 | -3.536170000 | 0.107777000 |
| H | -2.342029000 | -3.339993000 | 0.004529000 |
| C | -0.394242000 | -2.462726000 | 0.202910000 |
| C | -5.001180000 | 3.422595000 | -0.459293000 |
| C | -0.762722000 | -1.033901000 | 0.196031000 |
| C | -6.105293000 | 2.644618000 | -0.120943000 |
| C | 0.349398000 | -0.181875000 | 0.015913000 |
| C | -5.943756000 | 1.313961000 | 0.246417000 |
| C | 0.225425000 | 1.210664000 | -0.186617000 |
| C | 1.525099000 | 1.922185000 | -0.175889000 |
| C | 1.698300000 | 3.293234000 | -0.052885000 |
| H | 0.853083000 | 3.957596000 | 0.055288000 |
| C | 2.986767000 | 3.861573000 | -0.019757000 |
| C | 4.097852000 | 3.011965000 | -0.103961000 |
| H | 5.116266000 | 3.375880000 | -0.094608000 |
| C | 3.863369000 | 1.646789000 | -0.170964000 |
| H | 4.697459000 | 0.955676000 | -0.201039000 |
| C | -4.655992000 | 0.733276000 | 0.338099000 |
| C | -1.067419000 | 1.754065000 | -0.378511000 |
| C | -3.522603000 | 1.513214000 | -0.063022000 |
| C | -2.208111000 | 0.935385000 | -0.035400000 |
| C | -3.701395000 | 2.869190000 | -0.491747000 |
| C | -2.050669000 | -0.447520000 | 0.337232000 |
| C | -2.567409000 | 3.581091000 | -0.997771000 |
| C | -3.188486000 | -1.132285000 | 0.872819000 |
| H | -2.713318000 | 4.548685000 | -1.462887000 |
| H | -3.053932000 | -2.097445000 | 1.343200000 |
| C | -1.315399000 | 3.050360000 | -0.938769000 |
| C | -4.430432000 | -0.571031000 | 0.876669000 |
| H | -0.495410000 | 3.599672000 | -1.382555000 |
| H | -5.256642000 | -1.111980000 | 1.322368000 |
| O | 3.035799000 | 5.191924000 | 0.100209000 |
| O | -1.586457000 | -5.945945000 | 0.005670000 |
| C | -3.020721000 | -5.805752000 | -0.078263000 |

| | | | |
|---|---|---|---|
| C | -3.656085000 | -7.200004000 | -0.175609000 |
| C | -5.181326000 | -6.993822000 | -0.260626000 |
| C | -3.157643000 | -7.920196000 | -1.442876000 |
| C | -3.311797000 | -8.028279000 | 1.076737000 |
| C | 4.318054000 | 5.857698000 | 0.154940000 |
| C | 4.092092000 | 7.365553000 | 0.328933000 |
| C | 3.347095000 | 7.640280000 | 1.648992000 |
| C | 3.287742000 | 7.921867000 | -0.861308000 |
| C | 5.484773000 | 8.025350000 | 0.369986000 |
| H | -3.376954000 | -5.277897000 | 0.815561000 |
| H | -3.268034000 | -5.208721000 | -0.964841000 |
| H | -5.688517000 | -7.962178000 | -0.335672000 |
| H | -5.457830000 | -6.402210000 | -1.141827000 |
| H | -5.567402000 | -6.481355000 | 0.628837000 |
| H | -3.404817000 | -7.347911000 | -2.345591000 |
| H | -3.629103000 | -8.905696000 | -1.530158000 |
| H | -2.072688000 | -8.063998000 | -1.417782000 |
| H | -3.671941000 | -7.535603000 | 1.988295000 |
| H | -2.230909000 | -8.172939000 | 1.172954000 |
| H | -3.782504000 | -9.016478000 | 1.020580000 |
| H | 4.860466000 | 5.650668000 | -0.775593000 |
| H | 4.889910000 | 5.451142000 | 0.997968000 |
| H | 3.204506000 | 8.717992000 | 1.788075000 |
| H | 3.913935000 | 7.262314000 | 2.508765000 |
| H | 2.361011000 | 7.164632000 | 1.655718000 |
| H | 2.297278000 | 7.459545000 | -0.922995000 |
| H | 3.808244000 | 7.740439000 | -1.809721000 |
| H | 3.151146000 | 9.004003000 | -0.754431000 |
| H | 6.084005000 | 7.645887000 | 1.206565000 |
| H | 5.383920000 | 9.109222000 | 0.495015000 |
| H | 6.041944000 | 7.845059000 | -0.557348000 |
| H | -7.095585000 | 3.079217000 | -0.144832000 |
| H | 7.697217000 | -3.109926000 | -0.090914000 |
| N | -5.235725000 | 4.779349000 | -0.803799000 |
| C | -4.587551000 | 5.896481000 | -0.344109000 |
| C | -5.245212000 | 6.986594000 | -0.869972000 |
| C | -6.297899000 | 6.431625000 | -1.635113000 |
| N | -6.302317000 | 5.102100000 | -1.591229000 |
| N | -7.112090000 | 0.571150000 | 0.559333000 |
| N | -8.103583000 | 1.149287000 | 1.297241000 |
| C | -9.080460000 | 0.246516000 | 1.314082000 |
| C | -8.744974000 | -0.915457000 | 0.580023000 |
| C | -7.476582000 | -0.669083000 | 0.102745000 |
| H | -6.826227000 | -1.247305000 | -0.536184000 |
| H | -9.343756000 | -1.799344000 | 0.416057000 |
| H | -9.992329000 | 0.458486000 | 1.857708000 |
| H | -7.049055000 | 6.947816000 | -2.219308000 |
| H | -5.006178000 | 8.028871000 | -0.717710000 |
| H | -3.747036000 | 5.822495000 | 0.329325000 |

Cartesian coordinates of DFT-optimized structure of **3aox** complex by B3LYP/Def2-TZVP; 6-31G(d,p)/$CH_3CN$, charge = 2, multiplicity = 2

| | | | |
|---|---|---|---|
| C | 4.065967000 | 2.476116000 | -0.136506000 |
| C | 5.422778000 | 2.470428000 | -0.231096000 |
| C | 6.166686000 | 1.252520000 | -0.186931000 |
| C | 5.455415000 | 0.016791000 | -0.038811000 |
| C | 4.020171000 | 0.012803000 | 0.047942000 |
| C | 3.307714000 | 1.272169000 | 0.026407000 |
| C | 6.183763000 | -1.218436000 | 0.014353000 |
| C | 5.454331000 | -2.448405000 | -0.034664000 |
| C | 4.096678000 | -2.463536000 | 0.006080000 |
| C | 3.331930000 | -1.257668000 | 0.137707000 |
| C | 1.899719000 | 1.220570000 | 0.139174000 |
| C | 1.228847000 | -0.009628000 | 0.236749000 |
| C | 1.934804000 | -1.221578000 | 0.284635000 |
| C | 7.578196000 | 1.218079000 | -0.278770000 |
| C | 8.277673000 | 0.027389000 | -0.131338000 |
| C | 7.595084000 | -1.174593000 | 0.034695000 |
| H | 3.564942000 | 3.427251000 | -0.216917000 |
| H | 5.946502000 | 3.412106000 | -0.336576000 |
| H | 3.590005000 | -3.409739000 | -0.118320000 |
| H | 5.993540000 | -3.380610000 | -0.151782000 |
| N | 0.984549000 | 2.315792000 | 0.139808000 |
| N | -0.365423000 | 1.987021000 | 0.043768000 |
| C | -1.045376000 | 3.131248000 | 0.115128000 |
| C | -0.168400000 | 4.215834000 | 0.275397000 |
| C | 1.096311000 | 3.668218000 | 0.299072000 |
| N | 1.063892000 | -2.343458000 | 0.448930000 |
| N | -0.294127000 | -2.103205000 | 0.304114000 |
| C | -0.915903000 | -3.254942000 | 0.581848000 |
| C | 0.012415000 | -4.244895000 | 0.930457000 |
| C | 1.248236000 | -3.632328000 | 0.853388000 |
| N | 8.336014000 | 2.403876000 | -0.482521000 |
| N | 9.412655000 | 2.661901000 | 0.314205000 |
| C | 9.951859000 | 3.765919000 | -0.197504000 |
| C | 8.217943000 | 3.322213000 | -1.491874000 |
| C | 9.245913000 | 4.226405000 | -1.333125000 |
| N | 8.367029000 | -2.355304000 | 0.186778000 |
| N | 9.478916000 | -2.540897000 | -0.582232000 |
| C | 10.019434000 | -3.667094000 | -0.125241000 |
| C | 9.279535000 | -4.216249000 | 0.948411000 |
| C | 8.228922000 | -3.343642000 | 1.126922000 |
| C | -0.095024000 | -0.098929000 | -2.911658000 |
| C | -0.361185000 | -0.192575000 | -4.271952000 |
| C | -1.682702000 | -0.317859000 | -4.695803000 |
| C | -2.696156000 | -0.342664000 | -3.741397000 |
| C | -2.379089000 | -0.247271000 | -2.387323000 |
| N | -1.071561000 | -0.130809000 | -1.979286000 |
| C | -3.383924000 | -0.249710000 | -1.310457000 |
| N | -2.839780000 | -0.155542000 | -0.077633000 |

| | | | |
|---|---|---|---|
| C | -3.592890000 | -0.112160000 | 1.043390000 |
| C | -4.984338000 | -0.183145000 | 0.951610000 |
| C | -5.567047000 | -0.291855000 | -0.311667000 |
| C | -4.770748000 | -0.321061000 | -1.457080000 |
| C | -2.791445000 | 0.028937000 | 2.271642000 |
| N | -1.431628000 | 0.109641000 | 2.086195000 |
| C | -0.631005000 | 0.266178000 | 3.162340000 |
| C | -1.130119000 | 0.334016000 | 4.457226000 |
| C | -2.506302000 | 0.245686000 | 4.656953000 |
| C | -3.339570000 | 0.094856000 | 3.551652000 |
| H | 0.917060000 | -0.002513000 | -2.541263000 |
| H | 0.462391000 | -0.165614000 | -4.975956000 |
| H | -1.924099000 | -0.393508000 | -5.750379000 |
| H | -3.730934000 | -0.438383000 | -4.046597000 |
| H | -5.605236000 | -0.148830000 | 1.838173000 |
| H | -5.226239000 | -0.390987000 | -2.437100000 |
| H | 0.430763000 | 0.329014000 | 2.963757000 |
| H | -0.441587000 | 0.455266000 | 5.285328000 |
| H | -2.927923000 | 0.295847000 | 5.654896000 |
| H | -4.412730000 | 0.028620000 | 3.682558000 |
| Os | -0.745640000 | -0.063962000 | 0.101224000 |
| H | -2.124194000 | 3.117811000 | 0.059050000 |
| H | -0.423015000 | 5.260057000 | 0.371643000 |
| H | 2.042056000 | 4.153481000 | 0.455045000 |
| H | -1.993350000 | -3.309336000 | 0.524419000 |
| H | -0.187814000 | -5.267407000 | 1.210789000 |
| H | 2.222753000 | -4.019421000 | 1.094443000 |
| H | 10.827935000 | 4.196931000 | 0.270179000 |
| H | 7.443997000 | 3.239828000 | -2.240555000 |
| H | 9.459381000 | 5.086877000 | -1.950181000 |
| H | 10.921168000 | -4.050333000 | -0.585646000 |
| H | 9.486826000 | -5.112680000 | 1.514169000 |
| H | 7.425486000 | -3.326112000 | 1.847987000 |
| H | 9.359407000 | 0.031419000 | -0.155277000 |
| H | -6.646051000 | -0.346372000 | -0.404116000 |

Cartesian coordinates of DFT-optimized structure of **4aox** complex by B3LYP/Def2-TZVP; 6-31G(d,p)/$CH_3CN$, charge = 2, multiplicity = 2

| | | | |
|---|---|---|---|
| Os | 1.790862000 | -3.056808000 | 0.026577000 |
| N | -1.561513000 | 6.817513000 | -1.668648000 |
| N | -0.164209000 | -3.703618000 | 0.439352000 |
| N | 2.418142000 | -3.263811000 | 2.027576000 |
| N | 2.849307000 | -4.879283000 | 0.043162000 |
| N | 1.683125000 | -3.726837000 | -1.968596000 |
| N | -5.213434000 | 4.776221000 | 1.374500000 |
| N | 3.318876000 | -1.677811000 | -0.390527000 |
| C | 2.157521000 | -2.372578000 | 3.008852000 |
| H | 1.583634000 | -1.500702000 | 2.723556000 |

| | | | |
|---|---|---|---|
| C | 2.593014000 | -2.554635000 | 4.315480000 |
| H | 2.353761000 | -1.806364000 | 5.062171000 |
| C | 3.326084000 | -3.696504000 | 4.632404000 |
| H | 3.679865000 | -3.868494000 | 5.643079000 |
| C | 3.599472000 | -4.619664000 | 3.627040000 |
| H | 4.168394000 | -5.514582000 | 3.847082000 |
| C | 3.141184000 | -4.392568000 | 2.330021000 |
| C | 3.385938000 | -5.311011000 | 1.205212000 |
| C | 4.087192000 | -6.517824000 | 1.244523000 |
| H | 4.524053000 | -6.880738000 | 2.166740000 |
| C | 4.217412000 | -7.254734000 | 0.066895000 |
| C | 3.655329000 | -6.790305000 | -1.122771000 |
| H | 3.758243000 | -7.363526000 | -2.035838000 |
| C | 2.962899000 | -5.577822000 | -1.107790000 |
| C | 2.300423000 | -4.922463000 | -2.248347000 |
| C | 2.278534000 | -5.451185000 | -3.538276000 |
| H | 2.770472000 | -6.394630000 | -3.741180000 |
| C | 1.625650000 | -4.766394000 | -4.559366000 |
| H | 1.605641000 | -5.173556000 | -5.564373000 |
| C | 1.002768000 | -3.555079000 | -4.265414000 |
| H | 0.481468000 | -2.983891000 | -5.024838000 |
| C | 1.052389000 | -3.068292000 | -2.965146000 |
| H | 0.581902000 | -2.131587000 | -2.696777000 |
| C | -1.069600000 | 8.027441000 | -1.979649000 |
| C | -0.858471000 | -4.839929000 | 0.531166000 |
| H | -1.685383000 | 8.637413000 | -2.638141000 |
| C | 0.142835000 | 8.522713000 | -1.521245000 |
| H | 0.496575000 | 9.508870000 | -1.801963000 |
| C | 0.908484000 | 7.718074000 | -0.663114000 |
| C | 0.414599000 | 6.451105000 | -0.325440000 |
| H | 0.949176000 | 5.802361000 | 0.355970000 |
| C | -0.816710000 | 6.040482000 | -0.860902000 |
| N | -1.092118000 | -2.671681000 | 0.455158000 |
| C | -1.417027000 | 4.724837000 | -0.488027000 |
| C | -0.580930000 | -1.353682000 | 0.285126000 |
| C | -2.759338000 | 4.720708000 | -0.105964000 |
| C | 0.796142000 | -1.356019000 | 0.004989000 |
| C | -3.427794000 | 3.563460000 | 0.297921000 |
| C | 1.472656000 | -0.161536000 | -0.294842000 |
| C | -4.875997000 | 3.695337000 | 0.633853000 |
| N | 2.874086000 | -0.364076000 | -0.438738000 |
| C | -5.825051000 | 2.798907000 | 0.140295000 |
| H | -5.542579000 | 1.962606000 | -0.489158000 |
| C | -7.183051000 | 2.998377000 | 0.436863000 |
| C | -7.536242000 | 4.106643000 | 1.216700000 |
| H | -8.562623000 | 4.325679000 | 1.482448000 |
| C | -6.509978000 | 4.950799000 | 1.643776000 |
| C | 4.651369000 | -1.629686000 | -0.449528000 |
| H | -6.759015000 | 5.824296000 | 2.244303000 |
| C | -2.710579000 | 2.342962000 | 0.374148000 |
| C | 0.769779000 | 1.047450000 | -0.450744000 |

| | | | |
|---|---|---|---|
| C | -1.341927000 | 2.298742000 | -0.058952000 |
| C | -0.616547000 | 1.058890000 | -0.036461000 |
| C | -0.702693000 | 3.500847000 | -0.515865000 |
| C | -1.292411000 | -0.144689000 | 0.398614000 |
| C | 0.620141000 | 3.402402000 | -1.057757000 |
| C | -2.610631000 | -0.026872000 | 0.947009000 |
| H | 1.060618000 | 4.271239000 | -1.531578000 |
| H | -3.065220000 | -0.876468000 | 1.438417000 |
| C | 1.322659000 | 2.238536000 | -1.023327000 |
| C | -3.280918000 | 1.156303000 | 0.939365000 |
| H | 2.292973000 | 2.203695000 | -1.500487000 |
| H | -4.261653000 | 1.210318000 | 1.395886000 |
| O | 2.070148000 | 8.240673000 | -0.218336000 |
| O | -8.045919000 | 2.094195000 | -0.071587000 |
| C | -9.454471000 | 2.257163000 | 0.173208000 |
| C | -10.220427000 | 1.110878000 | -0.503138000 |
| C | -11.718277000 | 1.336825000 | -0.219242000 |
| C | -9.777779000 | -0.241513000 | 0.087065000 |
| C | -9.972182000 | 1.132847000 | -2.023290000 |
| C | 2.903656000 | 7.464055000 | 0.659590000 |
| C | 4.156405000 | 8.277945000 | 1.016023000 |
| C | 4.951671000 | 8.612224000 | -0.260278000 |
| C | 3.754594000 | 9.574160000 | 1.745022000 |
| C | 5.015711000 | 7.402426000 | 1.948842000 |
| H | -9.778349000 | 3.225765000 | -0.229544000 |
| H | -9.634096000 | 2.251887000 | 1.256245000 |
| H | -12.063334000 | 2.294469000 | -0.627909000 |
| H | -11.928404000 | 1.330856000 | 0.857190000 |
| H | -12.314651000 | 0.541738000 | -0.681066000 |
| H | -9.961493000 | -0.279794000 | 1.168031000 |
| H | -8.710699000 | -0.417951000 | -0.082332000 |
| H | -10.336242000 | -1.062412000 | -0.377611000 |
| H | -10.289625000 | 2.087420000 | -2.461117000 |
| H | -10.539281000 | 0.333262000 | -2.513929000 |
| H | -8.911898000 | 0.988941000 | -2.254530000 |
| H | 3.183465000 | 6.529451000 | 0.155604000 |
| H | 2.337843000 | 7.215418000 | 1.566971000 |
| H | 5.255740000 | 7.698902000 | -0.786708000 |
| H | 5.859335000 | 9.172673000 | -0.008146000 |
| H | 4.357714000 | 9.219947000 | -0.950332000 |
| H | 3.127166000 | 10.208127000 | 1.110355000 |
| H | 4.646250000 | 10.147857000 | 2.023245000 |
| H | 3.196571000 | 9.353850000 | 2.663618000 |
| H | 5.319059000 | 6.470228000 | 1.456727000 |
| H | 4.473953000 | 7.142200000 | 2.866436000 |
| H | 5.925997000 | 7.939257000 | 2.238939000 |
| H | -3.306870000 | 5.655524000 | -0.123229000 |
| H | 4.758788000 | -8.194251000 | 0.076186000 |
| C | 3.951563000 | 0.472746000 | -0.494649000 |
| C | 5.094969000 | -0.298603000 | -0.517904000 |
| C | -2.349137000 | -3.200251000 | 0.523946000 |

| | | | |
|---|---|---|---|
| C | -2.236312000 | -4.573640000 | 0.588022000 |
| H | -0.337358000 | -5.786263000 | 0.542545000 |
| H | 5.221800000 | -2.547030000 | -0.429398000 |
| H | 3.839186000 | 1.542840000 | -0.481390000 |
| H | 6.113789000 | 0.053363000 | -0.568300000 |
| H | -3.227678000 | -2.580082000 | 0.490150000 |
| H | -3.042181000 | -5.287922000 | 0.657101000 |

Cartesian coordinates of DFT-optimized structure of **5aox** complex by B3LYP/Def2-TZVP; 6-31G(d,p)/$CH_3CN$, charge = 2, multiplicity = 2

| | | | |
|---|---|---|---|
| Os | 1.883703000 | -2.975778000 | 0.009239000 |
| N | -0.081111000 | -3.668440000 | 0.311749000 |
| N | 2.339809000 | -3.289725000 | 2.041168000 |
| N | 3.062340000 | -4.713196000 | -0.004756000 |
| N | 1.777721000 | -3.636564000 | -1.985557000 |
| N | -5.131675000 | 4.877333000 | 1.362798000 |
| C | 1.882964000 | -2.528689000 | 3.059358000 |
| H | 1.204983000 | -1.729384000 | 2.789779000 |
| C | 2.261385000 | -2.746176000 | 4.378163000 |
| H | 1.869721000 | -2.101292000 | 5.156195000 |
| C | 3.134681000 | -3.793407000 | 4.666971000 |
| H | 3.447727000 | -3.990845000 | 5.686418000 |
| C | 3.598233000 | -4.592923000 | 3.625305000 |
| H | 4.270210000 | -5.417997000 | 3.827818000 |
| C | 3.194178000 | -4.332709000 | 2.316943000 |
| C | 3.611244000 | -5.137576000 | 1.155408000 |
| C | 4.462904000 | -6.243967000 | 1.159200000 |
| H | 4.912706000 | -6.600195000 | 2.077772000 |
| C | 4.724604000 | -6.891402000 | -0.049617000 |
| C | 4.134233000 | -6.446144000 | -1.233298000 |
| H | 4.330065000 | -6.958207000 | -2.167486000 |
| C | 3.288023000 | -5.336221000 | -1.182871000 |
| C | 2.549982000 | -4.729951000 | -2.304155000 |
| C | 2.584165000 | -5.219690000 | -3.608880000 |
| H | 3.194263000 | -6.083261000 | -3.843992000 |
| C | 1.830962000 | -4.600512000 | -4.602776000 |
| H | 1.855730000 | -4.976630000 | -5.619758000 |
| C | 1.044271000 | -3.499738000 | -4.267412000 |
| H | 0.437252000 | -2.988536000 | -5.005555000 |
| C | 1.038773000 | -3.050962000 | -2.952906000 |
| H | 0.442586000 | -2.200512000 | -2.648411000 |
| C | -0.456839000 | -4.965906000 | 0.370727000 |
| H | 0.346551000 | -5.692324000 | 0.384872000 |
| C | -1.771672000 | -5.377106000 | 0.401551000 |
| H | -2.024601000 | -6.429453000 | 0.448900000 |
| C | -2.782394000 | -4.402791000 | 0.335830000 |
| C | -2.409779000 | -3.053503000 | 0.285434000 |
| H | -3.163175000 | -2.289467000 | 0.185303000 |
| C | -1.059029000 | -2.687464000 | 0.310231000 |

| | | | |
|---|---|---|---|
| C | -1.336507000 | 4.788597000 | -0.468379000 |
| C | -0.537131000 | -1.308685000 | 0.236878000 |
| C | -2.676230000 | 4.816557000 | -0.092710000 |
| C | 0.853841000 | -1.281824000 | 0.003733000 |
| C | -3.353347000 | 3.657532000 | 0.285434000 |
| C | 1.538117000 | -0.084841000 | -0.252453000 |
| C | -4.801513000 | 3.797751000 | 0.617431000 |
| C | -5.755604000 | 2.912014000 | 0.114855000 |
| H | -5.477620000 | 2.077701000 | -0.519052000 |
| C | -7.113442000 | 3.123060000 | 0.405820000 |
| C | -7.459176000 | 4.228401000 | 1.193238000 |
| H | -8.484217000 | 4.454753000 | 1.458057000 |
| C | -6.427303000 | 5.060850000 | 1.629690000 |
| H | -6.670917000 | 5.932178000 | 2.235446000 |
| C | -2.643289000 | 2.428993000 | 0.354054000 |
| C | 0.841621000 | 1.123426000 | -0.426853000 |
| C | -1.271712000 | 2.378097000 | -0.063960000 |
| C | -0.553278000 | 1.133982000 | -0.042551000 |
| C | -0.618305000 | 3.573978000 | -0.510698000 |
| C | -1.232217000 | -0.072902000 | 0.360044000 |
| C | 0.710725000 | 3.480165000 | -1.035064000 |
| C | -2.548260000 | 0.055410000 | 0.909853000 |
| H | 1.159311000 | 4.349133000 | -1.501436000 |
| H | -3.005247000 | -0.794929000 | 1.398369000 |
| C | 1.408421000 | 2.313591000 | -0.989249000 |
| C | -3.218498000 | 1.241757000 | 0.907448000 |
| H | 2.387372000 | 2.275593000 | -1.448899000 |
| H | -4.200095000 | 1.295163000 | 1.362714000 |
| O | -4.044361000 | -4.851782000 | 0.317615000 |
| O | -7.983454000 | 2.233128000 | -0.115233000 |
| C | -9.391762000 | 2.418018000 | 0.116564000 |
| C | -10.173358000 | 1.296054000 | -0.582326000 |
| C | -11.668646000 | 1.549316000 | -0.307459000 |
| C | -9.764675000 | -0.072617000 | -0.005518000 |
| C | -9.910674000 | 1.332568000 | -2.099626000 |
| C | -5.142024000 | -3.916718000 | 0.257253000 |
| C | -6.463730000 | -4.697708000 | 0.263812000 |
| C | -7.600226000 | -3.658526000 | 0.201543000 |
| C | -6.536427000 | -5.623166000 | -0.965523000 |
| C | -6.584776000 | -5.523633000 | 1.558605000 |
| H | -9.693076000 | 3.397846000 | -0.276402000 |
| H | -9.582914000 | 2.401394000 | 1.197452000 |
| H | -11.990475000 | 2.519250000 | -0.705791000 |
| H | -11.886589000 | 1.533716000 | 0.767384000 |
| H | -12.277271000 | 0.772670000 | -0.784029000 |
| H | -9.955010000 | -0.118686000 | 1.074001000 |
| H | -8.701253000 | -0.272969000 | -0.171696000 |
| H | -10.339944000 | -0.874761000 | -0.482254000 |
| H | -10.207139000 | 2.297854000 | -2.528419000 |
| H | -10.487140000 | 0.549179000 | -2.605228000 |
| H | -8.850956000 | 1.172891000 | -2.323043000 |

| | | | |
|---|---|---|---|
| H | -5.083335000 | -3.246550000 | 1.124149000 |
| H | -5.050851000 | -3.320486000 | -0.659360000 |
| H | -8.572852000 | -4.163142000 | 0.205518000 |
| H | -7.541112000 | -3.052647000 | -0.710752000 |
| H | -7.571319000 | -2.980688000 | 1.063307000 |
| H | -6.463300000 | -5.048728000 | -1.897244000 |
| H | -7.489198000 | -6.164672000 | -0.978347000 |
| H | -5.727468000 | -6.360727000 | -0.956194000 |
| H | -6.545469000 | -4.878039000 | 2.444658000 |
| H | -5.778316000 | -6.259715000 | 1.637814000 |
| H | -7.539086000 | -6.062305000 | 1.577680000 |
| H | -3.203407000 | 5.762232000 | -0.097683000 |
| H | 5.385338000 | -7.750998000 | -0.067776000 |
| N | 2.952234000 | -0.269976000 | -0.353222000 |
| C | 4.014636000 | 0.579765000 | -0.340384000 |
| C | 5.175136000 | -0.174313000 | -0.352157000 |
| C | 4.750733000 | -1.508009000 | -0.347529000 |
| N | 3.412066000 | -1.575475000 | -0.334342000 |
| N | -0.725437000 | 6.020353000 | -0.829348000 |
| C | 0.453658000 | 6.547768000 | -0.372574000 |
| C | 0.568851000 | 7.807540000 | -0.919172000 |
| C | -0.604246000 | 7.966980000 | -1.692987000 |
| N | -1.391805000 | 6.895666000 | -1.635515000 |
| H | 5.332666000 | -2.418379000 | -0.344717000 |
| H | 6.188725000 | 0.195623000 | -0.356038000 |
| H | 3.884606000 | 1.647350000 | -0.292354000 |
| H | 1.085693000 | 6.002422000 | 0.312177000 |
| H | 1.375765000 | 8.510889000 | -0.774318000 |
| H | -0.903997000 | 8.816950000 | -2.292814000 |

Cartesian coordinates of DFT-optimized structure of **6aox** complex by B3LYP/Def2-TZVP; 6-31G(d,p)/$CH_3CN$, charge = 2, multiplicity = 2

| | | | |
|---|---|---|---|
| Os | 1.841355000 | -2.463836000 | -0.313701000 |
| N | -0.063457000 | -3.210305000 | 0.185598000 |
| N | 2.424576000 | -2.505241000 | 1.709002000 |
| N | 3.102170000 | -4.138093000 | -0.191372000 |
| N | 1.667660000 | -3.360851000 | -2.208633000 |
| C | 1.990249000 | -1.642866000 | 2.653784000 |
| H | 1.263715000 | -0.908722000 | 2.330597000 |
| C | 2.448369000 | -1.683248000 | 3.964524000 |
| H | 2.071075000 | -0.963068000 | 4.681160000 |
| C | 3.380762000 | -2.654526000 | 4.325313000 |
| H | 3.756253000 | -2.714472000 | 5.341046000 |
| C | 3.822429000 | -3.557229000 | 3.361440000 |
| H | 4.538985000 | -4.326702000 | 3.621730000 |
| C | 3.337917000 | -3.473680000 | 2.057120000 |
| C | 3.728136000 | -4.398279000 | 0.978271000 |
| C | 4.625436000 | -5.464449000 | 1.065677000 |
| H | 5.137099000 | -5.690198000 | 1.993278000 |

| | | | |
|---|---|---|---|
| C | 4.849620000 | -6.245575000 | -0.069377000 |
| C | 4.180274000 | -5.968775000 | -1.262251000 |
| H | 4.345830000 | -6.585607000 | -2.137045000 |
| C | 3.293602000 | -4.890300000 | -1.297989000 |
| C | 2.476353000 | -4.449448000 | -2.441600000 |
| C | 2.475106000 | -5.082374000 | -3.683813000 |
| H | 3.118718000 | -5.936924000 | -3.853670000 |
| C | 1.644976000 | -4.615785000 | -4.699630000 |
| H | 1.641458000 | -5.103875000 | -5.668132000 |
| C | 0.818825000 | -3.521510000 | -4.448229000 |
| H | 0.151208000 | -3.127056000 | -5.205416000 |
| C | 0.853862000 | -2.924691000 | -3.194466000 |
| H | 0.230886000 | -2.072272000 | -2.955942000 |
| C | -0.367027000 | -4.507421000 | 0.417244000 |
| H | 0.472674000 | -5.189926000 | 0.471375000 |
| C | -1.655703000 | -4.972314000 | 0.568952000 |
| H | -1.849792000 | -6.022105000 | 0.753186000 |
| C | -2.718871000 | -4.061770000 | 0.442431000 |
| C | -2.420368000 | -2.712620000 | 0.212838000 |
| H | -3.215687000 | -2.001010000 | 0.061743000 |
| C | -1.091095000 | -2.284108000 | 0.124763000 |
| C | -1.806018000 | 5.070106000 | -1.398715000 |
| C | -0.648343000 | -0.899976000 | -0.132318000 |
| C | -3.134522000 | 5.042002000 | -0.967919000 |
| C | 0.723604000 | -0.833069000 | -0.453672000 |
| C | -3.697029000 | 3.895690000 | -0.420133000 |
| C | 1.327291000 | 0.360480000 | -0.874955000 |
| C | -2.931850000 | 2.718192000 | -0.252085000 |
| C | 0.559752000 | 1.511946000 | -1.123992000 |
| C | -1.590352000 | 2.698373000 | -0.756015000 |
| C | -0.809297000 | 1.496566000 | -0.656893000 |
| C | -1.033276000 | 3.882746000 | -1.345758000 |
| C | -1.399036000 | 0.307343000 | -0.092708000 |
| C | 0.270355000 | 3.792639000 | -1.935050000 |
| C | -2.683783000 | 0.428147000 | 0.529597000 |
| H | 0.649038000 | 4.632583000 | -2.504828000 |
| H | -3.061834000 | -0.389190000 | 1.129304000 |
| C | 1.029411000 | 2.668804000 | -1.826794000 |
| C | -3.415692000 | 1.574350000 | 0.455050000 |
| H | 1.980841000 | 2.633514000 | -2.341029000 |
| H | -4.368588000 | 1.631120000 | 0.967661000 |
| O | -3.955432000 | -4.566533000 | 0.545406000 |
| C | -5.100648000 | -3.697900000 | 0.411178000 |
| C | -6.380947000 | -4.524806000 | 0.595099000 |
| C | -7.568327000 | -3.554574000 | 0.438455000 |
| C | -6.460786000 | -5.623487000 | -0.481789000 |
| C | -6.405906000 | -5.155417000 | 2.000491000 |
| H | -5.037891000 | -2.908388000 | 1.170610000 |
| H | -5.079127000 | -3.235784000 | -0.583722000 |
| H | -8.514188000 | -4.094290000 | 0.559256000 |
| H | -7.574531000 | -3.085127000 | -0.552783000 |

| | | | |
|---|---|---|---|
| H | -7.538004000 | -2.758618000 | 1.192362000 |
| H | -6.457043000 | -5.190212000 | -1.489614000 |
| H | -7.385153000 | -6.201268000 | -0.367940000 |
| H | -5.616497000 | -6.316261000 | -0.406024000 |
| H | -6.358481000 | -4.385068000 | 2.780100000 |
| H | -5.562466000 | -5.838151000 | 2.145338000 |
| H | -7.331575000 | -5.723884000 | 2.145998000 |
| H | 5.542961000 | -7.077957000 | -0.021524000 |
| N | 2.741814000 | 0.231785000 | -1.039616000 |
| C | 3.757588000 | 1.125853000 | -1.182722000 |
| C | 4.953659000 | 0.429896000 | -1.179955000 |
| C | 4.600856000 | -0.913087000 | -1.004185000 |
| N | 3.270289000 | -1.040925000 | -0.905535000 |
| H | 5.230066000 | -1.788794000 | -0.936432000 |
| H | 5.944684000 | 0.843725000 | -1.284290000 |
| H | 3.575558000 | 2.184885000 | -1.245841000 |
| N | -5.056978000 | 3.953574000 | -0.011429000 |
| N | -5.515166000 | 5.045535000 | 0.665463000 |
| C | -6.821708000 | 4.833066000 | 0.802505000 |
| C | -7.228423000 | 3.615593000 | 0.208454000 |
| C | -6.069654000 | 3.080577000 | -0.311023000 |
| C | -1.288298000 | 6.373594000 | -1.911451000 |
| C | -0.056032000 | 6.882968000 | -1.472411000 |
| C | 0.356154000 | 8.140459000 | -1.933249000 |
| C | -0.486512000 | 8.833675000 | -2.816036000 |
| C | -1.691315000 | 8.245588000 | -3.174155000 |
| N | -2.107550000 | 7.044937000 | -2.741285000 |
| O | 1.506204000 | 8.753561000 | -1.585419000 |
| C | 2.406822000 | 8.099826000 | -0.673959000 |
| C | 3.616369000 | 9.011296000 | -0.418465000 |
| C | 4.547350000 | 8.264722000 | 0.556755000 |
| C | 4.360213000 | 9.287669000 | -1.738795000 |
| C | 3.153727000 | 10.336177000 | 0.217018000 |
| H | -7.427387000 | 5.560154000 | 1.328405000 |
| H | -8.221581000 | 3.193683000 | 0.159431000 |
| H | -5.887404000 | 2.182459000 | -0.881859000 |
| H | 0.541403000 | 6.318026000 | -0.769192000 |
| H | -0.196772000 | 9.809181000 | -3.191280000 |
| H | -2.365763000 | 8.768903000 | -3.849414000 |
| H | 2.729388000 | 7.145473000 | -1.110970000 |
| H | 1.879782000 | 7.892965000 | 0.266526000 |
| H | 4.043463000 | 8.049823000 | 1.507086000 |
| H | 5.431081000 | 8.874096000 | 0.777085000 |
| H | 4.893208000 | 7.314103000 | 0.132623000 |
| H | 4.709438000 | 8.354569000 | -2.198145000 |
| H | 5.236301000 | 9.921203000 | -1.557960000 |
| H | 3.713418000 | 9.799355000 | -2.458564000 |
| H | 2.628827000 | 10.158059000 | 1.163880000 |
| H | 2.476650000 | 10.880887000 | -0.448700000 |
| H | 4.015443000 | 10.980293000 | 0.427057000 |
| H | -3.740115000 | 5.935145000 | -1.055910000 |

Cartesian coordinates of DFT-optimized structure of **1box** complex by B3LYP/Def2-TZVP; 6-31G(d,p)/$CH_3CN$, charge = 2, multiplicity = 2

| | | | |
|---|---|---|---|
| Ru | 1.784655000 | -3.014718000 | 0.016152000 |
| N | -1.637184000 | 6.819290000 | -1.660949000 |
| N | -0.174306000 | -3.736029000 | 0.291244000 |
| N | 2.249667000 | -3.289295000 | 2.049453000 |
| N | 2.904102000 | -4.800980000 | 0.041808000 |
| N | 1.758493000 | -3.655396000 | -1.987596000 |
| N | -5.184153000 | 4.757752000 | 1.511248000 |
| N | 3.370115000 | -1.640525000 | -0.282687000 |
| C | 1.852450000 | -2.465907000 | 3.038743000 |
| H | 1.232331000 | -1.628814000 | 2.744325000 |
| C | 2.214314000 | -2.667705000 | 4.366769000 |
| H | 1.869104000 | -1.972779000 | 5.123515000 |
| C | 3.012512000 | -3.762761000 | 4.688810000 |
| H | 3.313183000 | -3.949550000 | 5.714110000 |
| C | 3.419844000 | -4.623096000 | 3.671830000 |
| H | 4.037760000 | -5.483234000 | 3.899170000 |
| C | 3.029476000 | -4.374559000 | 2.356581000 |
| C | 3.400140000 | -5.236939000 | 1.216350000 |
| C | 4.168939000 | -6.402922000 | 1.259518000 |
| H | 4.575858000 | -6.774071000 | 2.192198000 |
| C | 4.403042000 | -7.089771000 | 0.067119000 |
| C | 3.874373000 | -6.622334000 | -1.136889000 |
| H | 4.055061000 | -7.161862000 | -2.058443000 |
| C | 3.111483000 | -5.451671000 | -1.119605000 |
| C | 2.457739000 | -4.800414000 | -2.272161000 |
| C | 2.516127000 | -5.296040000 | -3.574165000 |
| H | 3.071769000 | -6.202103000 | -3.783541000 |
| C | 1.856976000 | -4.624077000 | -4.600716000 |
| H | 1.898742000 | -5.003696000 | -5.615908000 |
| C | 1.146254000 | -3.464471000 | -4.300162000 |
| H | 0.616016000 | -2.907493000 | -5.063986000 |
| C | 1.119084000 | -3.013507000 | -2.984441000 |
| H | 0.580404000 | -2.116180000 | -2.708116000 |
| C | -1.148826000 | 8.025515000 | -1.990428000 |
| C | -0.547907000 | -5.029694000 | 0.333366000 |
| H | -1.786400000 | 8.641465000 | -2.622109000 |
| H | 0.253847000 | -5.757973000 | 0.370404000 |
| C | 0.086415000 | 8.509880000 | -1.583657000 |
| C | -1.865773000 | -5.442207000 | 0.321939000 |
| H | 0.437313000 | 9.492427000 | -1.880174000 |
| H | -2.121115000 | -6.494511000 | 0.357635000 |
| C | 0.880118000 | 7.698093000 | -0.758434000 |
| C | -2.872012000 | -4.467267000 | 0.225958000 |
| C | 0.387343000 | 6.437376000 | -0.396609000 |
| C | -2.496020000 | -3.116483000 | 0.193962000 |
| H | 0.942225000 | 5.785870000 | 0.265460000 |

| | | | |
|---|---|---|---|
| H | -3.243574000 | -2.348323000 | 0.076727000 |
| C | -0.866593000 | 6.035353000 | -0.884344000 |
| C | -1.144979000 | -2.760393000 | 0.263652000 |
| C | -1.458191000 | 4.720895000 | -0.495505000 |
| C | -0.611700000 | -1.383939000 | 0.207611000 |
| C | -2.790683000 | 4.717562000 | -0.076955000 |
| C | 0.781238000 | -1.341564000 | -0.018162000 |
| C | -3.448680000 | 3.560276000 | 0.341247000 |
| C | 1.485390000 | -0.145334000 | -0.271068000 |
| C | -4.883673000 | 3.695176000 | 0.729219000 |
| C | 2.955010000 | -0.320991000 | -0.298677000 |
| C | -5.857645000 | 2.821275000 | 0.244036000 |
| C | 3.898848000 | 0.696063000 | -0.238812000 |
| H | -5.604376000 | 2.000235000 | -0.417259000 |
| H | 3.604600000 | 1.732481000 | -0.158744000 |
| C | -7.203141000 | 3.026683000 | 0.590523000 |
| C | 5.275770000 | 0.402695000 | -0.237248000 |
| C | -7.517343000 | 4.113227000 | 1.416310000 |
| C | 5.676282000 | -0.938553000 | -0.284349000 |
| H | -8.531171000 | 4.333482000 | 1.725676000 |
| H | 6.714951000 | -1.240258000 | -0.294286000 |
| C | -6.468617000 | 4.935164000 | 1.831796000 |
| C | 4.683302000 | -1.909698000 | -0.287692000 |
| H | -6.687996000 | 5.792380000 | 2.466386000 |
| H | 4.955803000 | -2.958962000 | -0.286641000 |
| C | -2.732847000 | 2.335798000 | 0.387472000 |
| C | 0.742571000 | 1.046650000 | -0.459917000 |
| C | -1.374307000 | 2.294973000 | -0.072347000 |
| C | -0.646284000 | 1.056343000 | -0.061991000 |
| C | -0.744657000 | 3.496923000 | -0.539066000 |
| C | -1.310714000 | -0.155590000 | 0.351026000 |
| C | 0.567440000 | 3.393958000 | -1.105384000 |
| C | -2.617205000 | -0.041885000 | 0.926182000 |
| H | 0.996087000 | 4.257098000 | -1.600485000 |
| H | -3.059858000 | -0.902874000 | 1.410222000 |
| C | 1.276680000 | 2.232826000 | -1.060418000 |
| C | -3.289568000 | 1.143030000 | 0.950116000 |
| H | 2.244251000 | 2.191801000 | -1.543806000 |
| H | -4.260862000 | 1.190520000 | 1.427825000 |
| O | 2.067369000 | 8.206800000 | -0.368115000 |
| O | 6.098552000 | 1.457158000 | -0.181702000 |
| O | -4.134573000 | -4.912092000 | 0.164071000 |
| O | -8.093928000 | 2.150353000 | 0.081194000 |
| C | -9.491401000 | 2.324988000 | 0.377297000 |
| C | -10.300354000 | 1.231623000 | -0.335345000 |
| C | -11.782430000 | 1.459388000 | 0.020839000 |
| C | -9.857144000 | -0.159855000 | 0.154993000 |
| C | -10.109918000 | 1.342570000 | -1.859919000 |
| C | -5.222896000 | -3.972747000 | 0.035888000 |
| C | -6.547772000 | -4.746162000 | -0.024585000 |
| C | -7.671222000 | -3.700372000 | -0.165382000 |

| | | | |
|---|---|---|---|
| C | -6.556489000 | -5.686204000 | -1.244918000 |
| C | -6.748303000 | -5.555383000 | 1.270827000 |
| C | 2.927563000 | 7.422639000 | 0.476979000 |
| C | 4.237862000 | 8.188598000 | 0.712668000 |
| C | 4.958980000 | 8.423834000 | -0.628095000 |
| C | 3.948090000 | 9.534982000 | 1.402664000 |
| C | 5.115446000 | 7.313818000 | 1.629183000 |
| C | 7.527513000 | 1.245280000 | -0.156617000 |
| C | 8.233591000 | 2.603882000 | -0.049456000 |
| C | 7.818602000 | 3.314002000 | 1.253130000 |
| C | 7.884639000 | 3.480810000 | -1.266851000 |
| C | 9.748818000 | 2.321270000 | -0.027742000 |
| H | -9.808438000 | 3.319176000 | 0.036358000 |
| H | -9.637832000 | 2.265179000 | 1.463734000 |
| H | -12.128079000 | 2.445442000 | -0.312568000 |
| H | -11.950185000 | 1.387869000 | 1.102368000 |
| H | -12.408583000 | 0.703037000 | -0.465787000 |
| H | -9.993438000 | -0.258966000 | 1.239095000 |
| H | -8.802165000 | -0.343763000 | -0.072601000 |
| H | -10.451062000 | -0.942723000 | -0.330878000 |
| H | -10.435299000 | 2.323840000 | -2.227212000 |
| H | -10.702000000 | 0.577557000 | -2.375445000 |
| H | -9.060474000 | 1.206518000 | -2.140283000 |
| H | -5.210124000 | -3.294618000 | 0.898517000 |
| H | -5.077468000 | -3.384917000 | -0.879001000 |
| H | -8.646314000 | -4.198272000 | -0.209077000 |
| H | -7.555560000 | -3.109108000 | -1.081847000 |
| H | -7.686174000 | -3.008969000 | 0.685981000 |
| H | -6.423729000 | -5.124116000 | -2.177580000 |
| H | -7.511666000 | -6.220003000 | -1.307113000 |
| H | -5.755878000 | -6.429799000 | -1.178706000 |
| H | -6.755852000 | -4.899096000 | 2.149832000 |
| H | -5.951943000 | -6.294539000 | 1.405046000 |
| H | -7.705146000 | -6.089150000 | 1.241820000 |
| H | 3.130833000 | 6.459900000 | -0.009680000 |
| H | 2.416155000 | 7.231282000 | 1.429665000 |
| H | 5.184027000 | 7.473101000 | -1.127398000 |
| H | 5.906568000 | 8.950189000 | -0.465488000 |
| H | 4.347362000 | 9.026921000 | -1.306783000 |
| H | 3.309493000 | 10.170238000 | 0.780628000 |
| H | 4.882870000 | 10.074918000 | 1.593194000 |
| H | 3.444362000 | 9.385013000 | 2.365626000 |
| H | 5.337480000 | 6.344090000 | 1.166763000 |
| H | 4.626099000 | 7.127209000 | 2.592903000 |
| H | 6.069402000 | 7.814553000 | 1.829741000 |
| H | 7.822293000 | 0.724506000 | -1.076130000 |
| H | 7.775135000 | 0.612510000 | 0.704450000 |
| H | 8.346201000 | 4.269746000 | 1.349052000 |
| H | 8.064098000 | 2.704386000 | 2.131513000 |
| H | 6.742746000 | 3.516930000 | 1.270268000 |
| H | 6.809241000 | 3.679759000 | -1.318053000 |

| | | | |
|---|---|---|---|
| H | 8.186208000 | 2.995681000 | -2.203317000 |
| H | 8.405101000 | 4.443351000 | -1.204366000 |
| H | 10.027011000 | 1.691060000 | 0.825728000 |
| H | 10.306019000 | 3.261084000 | 0.054730000 |
| H | 10.076140000 | 1.816620000 | -0.944804000 |
| H | -3.336144000 | 5.653720000 | -0.077287000 |
| H | 4.997789000 | -7.996647000 | 0.077042000 |

Cartesian coordinates of DFT-optimized structure of **2box** complex by B3LYP/Def2-TZVP; 6-31G(d,p)/$CH_3CN$, charge = 2, multiplicity = 2

| | | | |
|---|---|---|---|
| Ru | 1.918634000 | -1.340919000 | -0.028594000 |
| N | 0.442683000 | -2.826641000 | 0.119495000 |
| N | 2.389380000 | -1.556379000 | 2.011970000 |
| N | 3.695080000 | -2.470973000 | -0.038243000 |
| N | 2.196990000 | -1.818291000 | -2.057014000 |
| N | 2.780904000 | 0.588128000 | -0.195738000 |
| C | 1.639333000 | -1.076763000 | 3.022912000 |
| H | 0.720130000 | -0.578608000 | 2.742615000 |
| C | 2.016081000 | -1.204468000 | 4.355930000 |
| H | 1.377281000 | -0.796618000 | 5.130720000 |
| C | 3.208169000 | -1.857826000 | 4.659221000 |
| H | 3.531590000 | -1.975413000 | 5.687868000 |
| C | 3.983117000 | -2.366687000 | 3.619365000 |
| H | 4.910344000 | -2.884425000 | 3.832876000 |
| C | 3.560983000 | -2.208933000 | 2.300079000 |
| C | 4.304223000 | -2.730373000 | 1.135172000 |
| C | 5.507893000 | -3.439578000 | 1.154149000 |
| H | 6.015985000 | -3.660140000 | 2.084872000 |
| C | 6.047504000 | -3.867185000 | -0.060325000 |
| C | 5.392280000 | -3.597108000 | -1.262638000 |
| H | 5.810431000 | -3.939435000 | -2.201264000 |
| C | 4.191833000 | -2.882817000 | -1.220922000 |
| C | 3.339425000 | -2.510920000 | -2.367507000 |
| C | 3.636605000 | -2.840914000 | -3.689236000 |
| H | 4.542751000 | -3.388176000 | -3.919315000 |
| C | 2.763856000 | -2.467041000 | -4.708357000 |
| H | 2.988828000 | -2.719822000 | -5.738903000 |
| C | 1.602507000 | -1.770975000 | -4.381087000 |
| H | 0.891665000 | -1.461804000 | -5.138593000 |
| C | 1.352996000 | -1.467747000 | -3.046610000 |
| H | 0.463663000 | -0.926958000 | -2.749170000 |
| C | 0.652982000 | -4.157300000 | 0.077386000 |
| H | 1.688134000 | -4.477992000 | 0.083908000 |
| C | -0.366231000 | -5.086614000 | 0.024114000 |
| H | -0.151929000 | -6.148289000 | -0.002566000 |
| C | -1.692597000 | -4.625895000 | -0.022849000 |
| C | -1.923869000 | -3.243724000 | 0.016749000 |
| H | -2.927237000 | -2.859959000 | -0.074760000 |
| C | -0.850281000 | -2.352660000 | 0.125005000 |

| | | | |
|---|---|---|---|
| C | -4.285386000 | 4.304590000 | -0.330453000 |
| C | -0.947697000 | -0.879712000 | 0.152591000 |
| C | -5.512235000 | 3.736748000 | 0.002239000 |
| C | 0.299023000 | -0.242789000 | -0.017719000 |
| C | -5.599599000 | 2.388380000 | 0.328763000 |
| C | 0.439236000 | 1.150316000 | -0.183377000 |
| C | 1.847811000 | 1.609733000 | -0.162308000 |
| C | 2.275255000 | 2.923137000 | -0.023147000 |
| H | 1.572028000 | 3.734334000 | 0.097585000 |
| C | 3.648613000 | 3.236068000 | 0.002048000 |
| C | 4.577300000 | 2.191916000 | -0.094075000 |
| H | 5.646298000 | 2.355612000 | -0.086816000 |
| C | 4.085823000 | 0.896031000 | -0.172050000 |
| H | 4.774538000 | 0.060029000 | -0.211902000 |
| C | -4.442591000 | 1.575119000 | 0.386059000 |
| C | -0.732974000 | 1.929493000 | -0.349311000 |
| C | -3.187051000 | 2.141746000 | -0.008684000 |
| C | -2.003132000 | 1.328934000 | -0.013260000 |
| C | -3.112403000 | 3.519139000 | -0.397569000 |
| C | -2.103275000 | -0.068742000 | 0.322348000 |
| C | -1.869958000 | 4.020970000 | -0.900300000 |
| C | -3.345322000 | -0.546030000 | 0.851697000 |
| H | -1.836022000 | 5.012021000 | -1.336993000 |
| H | -3.389627000 | -1.532921000 | 1.293227000 |
| C | -0.738597000 | 3.264256000 | -0.872547000 |
| C | -4.460786000 | 0.235980000 | 0.885313000 |
| H | 0.165758000 | 3.664181000 | -1.312190000 |
| H | -5.370776000 | -0.154442000 | 1.325076000 |
| O | 3.951473000 | 4.533072000 | 0.123955000 |
| O | -2.648171000 | -5.560810000 | -0.110111000 |
| C | -4.037867000 | -5.171683000 | -0.139187000 |
| C | -4.910590000 | -6.434224000 | -0.171618000 |
| C | -6.378355000 | -5.964956000 | -0.207837000 |
| C | -4.598167000 | -7.262321000 | -1.432456000 |
| C | -4.665824000 | -7.278404000 | 1.093560000 |
| C | 5.338061000 | 4.941370000 | 0.142662000 |
| C | 5.410143000 | 6.467983000 | 0.281700000 |
| C | 4.756687000 | 6.910062000 | 1.604816000 |
| C | 4.706326000 | 7.142617000 | -0.911006000 |
| C | 6.904534000 | 6.846267000 | 0.286497000 |
| H | -4.257406000 | -4.575245000 | 0.755125000 |
| H | -4.215373000 | -4.556158000 | -1.029900000 |
| H | -7.049656000 | -6.830774000 | -0.227288000 |
| H | -6.584839000 | -5.360975000 | -1.099722000 |
| H | -6.630197000 | -5.365725000 | 0.675585000 |
| H | -4.775745000 | -6.677276000 | -2.343346000 |
| H | -5.239535000 | -8.150159000 | -1.473240000 |
| H | -3.555665000 | -7.596487000 | -1.440648000 |
| H | -4.890753000 | -6.704260000 | 2.000951000 |
| H | -3.625529000 | -7.614252000 | 1.152746000 |
| H | -5.309475000 | -8.165456000 | 1.089318000 |

| | | | |
|---|---|---|---|
| H | 5.812793000 | 4.613945000 | -0.790378000 |
| H | 5.837849000 | 4.451251000 | 0.987027000 |
| H | 4.825807000 | 7.997962000 | 1.718270000 |
| H | 5.257042000 | 6.449538000 | 2.465685000 |
| H | 3.698198000 | 6.632284000 | 1.637975000 |
| H | 3.644244000 | 6.879591000 | -0.947004000 |
| H | 5.164082000 | 6.842229000 | -1.861568000 |
| H | 4.783074000 | 8.232930000 | -0.830774000 |
| H | 7.435396000 | 6.375709000 | 1.122993000 |
| H | 7.018212000 | 7.931504000 | 0.385941000 |
| H | 7.398154000 | 6.541910000 | -0.644419000 |
| H | -6.403740000 | 4.349488000 | 0.006280000 |
| H | 6.981157000 | -4.418965000 | -0.069045000 |
| N | -4.264615000 | 5.691212000 | -0.632049000 |
| C | -3.414552000 | 6.652373000 | -0.149194000 |
| C | -3.862986000 | 7.862542000 | -0.630504000 |
| C | -5.008901000 | 7.538829000 | -1.394612000 |
| N | -5.260693000 | 6.232653000 | -1.391395000 |
| N | -6.883377000 | 1.867276000 | 0.636392000 |
| N | -7.742031000 | 2.595382000 | 1.407214000 |
| C | -8.869215000 | 1.889035000 | 1.414067000 |
| C | -8.763317000 | 0.708845000 | 0.641019000 |
| C | -7.476531000 | 0.730878000 | 0.150142000 |
| H | -6.951629000 | 0.062917000 | -0.516094000 |
| H | -9.517536000 | -0.043241000 | 0.461583000 |
| H | -9.719896000 | 2.248867000 | 1.978719000 |
| H | -5.656717000 | 8.204851000 | -1.950238000 |
| H | -3.432226000 | 8.836562000 | -0.450496000 |
| H | -2.595502000 | 6.401011000 | 0.507723000 |

Cartesian coordinates of DFT-optimized structure of **3box** complex by B3LYP/Def2-TZVP; 6-31G(d,p)/$CH_3CN$, charge = 2, multiplicity = 2

| | | | |
|---|---|---|---|
| C | 2.339396000 | -2.461287000 | -0.334656000 |
| C | 3.697547000 | -2.452446000 | -0.269761000 |
| C | 4.427820000 | -1.234003000 | -0.117720000 |
| C | 3.701018000 | -0.001318000 | -0.030661000 |
| C | 2.264554000 | -0.002821000 | -0.083875000 |
| C | 1.566537000 | -1.256903000 | -0.270203000 |
| C | 4.413823000 | 1.234978000 | 0.118711000 |
| C | 3.672716000 | 2.426173000 | 0.399440000 |
| C | 2.314221000 | 2.427720000 | 0.393465000 |
| C | 1.560600000 | 1.251692000 | 0.070361000 |
| C | 0.157356000 | -1.207741000 | -0.344818000 |
| C | -0.528766000 | 0.008616000 | -0.198536000 |
| C | 0.158994000 | 1.221758000 | -0.044128000 |
| C | 5.840086000 | -1.195467000 | -0.051508000 |
| C | 6.523027000 | 0.013520000 | -0.002722000 |
| C | 5.824967000 | 1.216047000 | 0.060148000 |
| H | 1.847588000 | -3.418351000 | -0.405930000 |

| | | | |
|---|---|---|---|
| H | 4.233745000 | -3.390405000 | -0.339062000 |
| H | 1.799846000 | 3.329777000 | 0.691958000 |
| H | 4.204675000 | 3.331528000 | 0.665371000 |
| N | -0.740027000 | -2.300181000 | -0.526543000 |
| N | -2.081131000 | -2.022645000 | -0.313422000 |
| C | -2.752716000 | -3.137835000 | -0.591157000 |
| C | -1.873206000 | -4.153150000 | -1.005232000 |
| C | -0.616493000 | -3.588523000 | -0.966134000 |
| N | -0.724689000 | 2.337612000 | 0.022317000 |
| N | -2.067661000 | 2.045856000 | 0.172345000 |
| C | -2.717275000 | 3.209765000 | 0.124398000 |
| C | -1.818200000 | 4.269899000 | -0.077736000 |
| C | -0.571899000 | 3.683041000 | -0.154862000 |
| N | 6.615941000 | -2.386586000 | -0.074902000 |
| N | 7.677078000 | -2.483550000 | -0.926532000 |
| C | 8.241179000 | -3.651422000 | -0.628047000 |
| C | 6.532353000 | -3.469557000 | 0.759715000 |
| C | 7.567225000 | -4.315223000 | 0.423317000 |
| N | 6.580763000 | 2.415804000 | 0.102669000 |
| N | 7.708625000 | 2.478107000 | 0.868401000 |
| C | 8.226387000 | 3.675110000 | 0.607161000 |
| C | 7.455524000 | 4.395231000 | -0.335768000 |
| C | 6.410127000 | 3.553141000 | -0.643899000 |
| C | -1.732716000 | -0.424866000 | 2.965297000 |
| C | -1.959981000 | -0.581800000 | 4.328760000 |
| C | -3.271711000 | -0.592217000 | 4.796212000 |
| C | -4.314220000 | -0.448079000 | 3.883662000 |
| C | -4.031007000 | -0.295398000 | 2.527127000 |
| N | -2.736266000 | -0.281192000 | 2.079151000 |
| C | -5.066884000 | -0.145673000 | 1.486798000 |
| N | -4.558441000 | -0.002173000 | 0.248063000 |
| C | -5.335903000 | 0.138523000 | -0.842553000 |
| C | -6.726868000 | 0.145580000 | -0.712073000 |
| C | -7.276552000 | 0.000548000 | 0.562264000 |
| C | -6.450979000 | -0.149171000 | 1.677136000 |
| C | -4.562298000 | 0.263187000 | -2.093538000 |
| N | -3.200226000 | 0.206256000 | -1.953504000 |
| C | -2.422619000 | 0.298088000 | -3.049024000 |
| C | -2.950968000 | 0.457554000 | -4.326085000 |
| C | -4.333485000 | 0.521707000 | -4.479649000 |
| C | -5.143521000 | 0.421697000 | -3.350797000 |
| H | -0.728622000 | -0.408787000 | 2.561586000 |
| H | -1.115263000 | -0.691914000 | 4.998832000 |
| H | -3.484634000 | -0.710781000 | 5.853086000 |
| H | -5.342387000 | -0.452284000 | 4.224324000 |
| H | -7.371084000 | 0.257016000 | -1.575353000 |
| H | -6.881536000 | -0.268384000 | 2.663668000 |
| H | -1.354106000 | 0.244651000 | -2.885861000 |
| H | -2.279759000 | 0.528373000 | -5.174070000 |
| H | -4.778956000 | 0.645973000 | -5.460733000 |
| H | -6.221475000 | 0.466603000 | -3.447060000 |

| | | | |
|---|---|---|---|
| Ru | -2.470085000 | -0.002339000 | 0.011055000 |
| H | -3.828498000 | -3.156546000 | -0.491790000 |
| H | -2.121918000 | -5.160099000 | -1.303064000 |
| H | 0.328616000 | -4.010217000 | -1.256096000 |
| H | -3.792554000 | 3.230612000 | 0.228600000 |
| H | -2.047062000 | 5.320736000 | -0.165733000 |
| H | 0.387125000 | 4.126595000 | -0.358091000 |
| H | 9.111380000 | -3.979664000 | -1.182122000 |
| H | 5.775317000 | -3.532009000 | 1.527363000 |
| H | 7.805374000 | -5.267033000 | 0.875050000 |
| H | 9.134694000 | 3.983650000 | 1.108895000 |
| H | 7.640029000 | 5.380690000 | -0.737584000 |
| H | 5.590213000 | 3.652270000 | -1.339368000 |
| H | 7.605023000 | 0.020780000 | -0.003253000 |
| H | -8.353873000 | 0.000966000 | 0.686591000 |

Cartesian coordinates of DFT-optimized structure of **4box** complex by B3LYP/Def2-TZVP; 6-31G(d,p)/$CH_3CN$, charge = 2, multiplicity = 2

| | | | |
|---|---|---|---|
| Ru | 3.823308000 | -0.349980000 | 0.002511000 |
| N | -6.109727000 | 2.687708000 | -1.779831000 |
| N | 3.212639000 | -2.284486000 | 0.516991000 |
| N | 4.363873000 | 0.125382000 | 1.980829000 |
| N | 5.915388000 | -0.559277000 | 0.013458000 |
| N | 4.277743000 | -0.935575000 | -1.967944000 |
| N | -6.603466000 | -1.327749000 | 1.440217000 |
| N | 3.624172000 | 1.668640000 | -0.517449000 |
| C | 3.493849000 | 0.472931000 | 2.948277000 |
| H | 2.449096000 | 0.503023000 | 2.666905000 |
| C | 3.901692000 | 0.777712000 | 4.242985000 |
| H | 3.158934000 | 1.051486000 | 4.983307000 |
| C | 5.258076000 | 0.721127000 | 4.553185000 |
| H | 5.610257000 | 0.951609000 | 5.552859000 |
| C | 6.162978000 | 0.361651000 | 3.557170000 |
| H | 7.222894000 | 0.311154000 | 3.774213000 |
| C | 5.700847000 | 0.066759000 | 2.275260000 |
| C | 6.583435000 | -0.321367000 | 1.157899000 |
| C | 7.973922000 | -0.452645000 | 1.194840000 |
| H | 8.531788000 | -0.266711000 | 2.104404000 |
| C | 8.637694000 | -0.831442000 | 0.027311000 |
| C | 7.923786000 | -1.074113000 | -1.146887000 |
| H | 8.442551000 | -1.368497000 | -2.050840000 |
| C | 6.534402000 | -0.928190000 | -1.124068000 |
| C | 5.603083000 | -1.141550000 | -2.249130000 |
| C | 6.009445000 | -1.531489000 | -3.524564000 |
| H | 7.060720000 | -1.690229000 | -3.731906000 |
| C | 5.060289000 | -1.715714000 | -4.527233000 |
| H | 5.369326000 | -2.019065000 | -5.521716000 |
| C | 3.716264000 | -1.504885000 | -4.230027000 |
| H | 2.940493000 | -1.635922000 | -4.975519000 |

| | | | |
|---|---|---|---|
| C | 3.364843000 | -1.115868000 | -2.941347000 |
| H | 2.331719000 | -0.941932000 | -2.669318000 |
| C | -6.797438000 | 3.784611000 | -2.135481000 |
| C | 3.727045000 | -3.505746000 | 0.655941000 |
| H | -7.657016000 | 3.619295000 | -2.782502000 |
| C | -6.478575000 | 5.074363000 | -1.735064000 |
| H | -7.067138000 | 5.929131000 | -2.050348000 |
| C | -5.372359000 | 5.254487000 | -0.890132000 |
| C | -4.639604000 | 4.123460000 | -0.506563000 |
| H | -3.796753000 | 4.201302000 | 0.167592000 |
| C | -5.038330000 | 2.865162000 | -0.985213000 |
| N | 1.837147000 | -2.431058000 | 0.538371000 |
| C | -4.328560000 | 1.621453000 | -0.561368000 |
| C | 1.076649000 | -1.250744000 | 0.317857000 |
| C | -5.115040000 | 0.552977000 | -0.127691000 |
| C | 1.887158000 | -0.154311000 | -0.016359000 |
| C | -4.573414000 | -0.650700000 | 0.327468000 |
| C | 1.321530000 | 1.080381000 | -0.371155000 |
| C | -5.532992000 | -1.726099000 | 0.714903000 |
| N | 2.306650000 | 2.085968000 | -0.566315000 |
| C | -5.369690000 | -3.042206000 | 0.280174000 |
| H | -4.529072000 | -3.335703000 | -0.338378000 |
| C | -6.331403000 | -4.006598000 | 0.622301000 |
| C | -7.433349000 | -3.603056000 | 1.386494000 |
| H | -8.215338000 | -4.289760000 | 1.684802000 |
| C | -7.508808000 | -2.258460000 | 1.753434000 |
| C | 4.373896000 | 2.765205000 | -0.625336000 |
| H | -8.359978000 | -1.917399000 | 2.340637000 |
| C | -3.164495000 | -0.788029000 | 0.403933000 |
| C | -0.070253000 | 1.217738000 | -0.527994000 |
| C | -2.324131000 | 0.270672000 | -0.081469000 |
| C | -0.895404000 | 0.126118000 | -0.059018000 |
| C | -2.919278000 | 1.474506000 | -0.590356000 |
| C | -0.319489000 | -1.107222000 | 0.430822000 |
| C | -2.061840000 | 2.458462000 | -1.182466000 |
| C | -1.188546000 | -2.077377000 | 1.027330000 |
| H | -2.506045000 | 3.304097000 | -1.693331000 |
| H | -0.767439000 | -2.922406000 | 1.555227000 |
| C | -0.707584000 | 2.340043000 | -1.149753000 |
| C | -2.539524000 | -1.921728000 | 1.018303000 |
| H | -0.108432000 | 3.079771000 | -1.663922000 |
| H | -3.159415000 | -2.660463000 | 1.511415000 |
| O | -5.105761000 | 6.518783000 | -0.501881000 |
| O | -6.111077000 | -5.258346000 | 0.169492000 |
| C | -7.073820000 | -6.287216000 | 0.461363000 |
| C | -6.600757000 | -7.611174000 | -0.156250000 |
| C | -7.667282000 | -8.673074000 | 0.175621000 |
| C | -5.247905000 | -8.025407000 | 0.453171000 |
| C | -6.471934000 | -7.466197000 | -1.684419000 |
| C | -3.981618000 | 6.770887000 | 0.359224000 |
| C | -3.895624000 | 8.276817000 | 0.648296000 |

| | | | |
|---|---|---|---|
| C | -3.703228000 | 9.058180000 | -0.665316000 |
| C | -5.174150000 | 8.751166000 | 1.364847000 |
| C | -2.675691000 | 8.493473000 | 1.564764000 |
| H | -8.047050000 | -5.995270000 | 0.045551000 |
| H | -7.175008000 | -6.386490000 | 1.550006000 |
| H | -8.643838000 | -8.405326000 | -0.246024000 |
| H | -7.786893000 | -8.797414000 | 1.258735000 |
| H | -7.377132000 | -9.643643000 | -0.242604000 |
| H | -5.325461000 | -8.147554000 | 1.540713000 |
| H | -4.475362000 | -7.276833000 | 0.249934000 |
| H | -4.915241000 | -8.980753000 | 0.030926000 |
| H | -7.430178000 | -7.180288000 | -2.135724000 |
| H | -6.159847000 | -8.416605000 | -2.132735000 |
| H | -5.730919000 | -6.705415000 | -1.949880000 |
| H | -3.065293000 | 6.421882000 | -0.135023000 |
| H | -4.110287000 | 6.208646000 | 1.293368000 |
| H | -2.789913000 | 8.740437000 | -1.183587000 |
| H | -3.617552000 | 10.131739000 | -0.461252000 |
| H | -4.548633000 | 8.907713000 | -1.344241000 |
| H | -6.060450000 | 8.590476000 | 0.742700000 |
| H | -5.108794000 | 9.821132000 | 1.593939000 |
| H | -5.319866000 | 8.213491000 | 2.310054000 |
| H | -1.747361000 | 8.166830000 | 1.080293000 |
| H | -2.779587000 | 7.942750000 | 2.507687000 |
| H | -2.569799000 | 9.556645000 | 1.808491000 |
| H | -6.192930000 | 0.661721000 | -0.144650000 |
| H | 9.716863000 | -0.938829000 | 0.032602000 |
| C | 2.265678000 | 3.446949000 | -0.672891000 |
| C | 3.563877000 | 3.909921000 | -0.728287000 |
| C | 1.524882000 | -3.755191000 | 0.659907000 |
| C | 2.703677000 | -4.465324000 | 0.751906000 |
| H | 4.799259000 | -3.638859000 | 0.671492000 |
| H | 5.451315000 | 2.683374000 | -0.614150000 |
| H | 1.335956000 | 3.989028000 | -0.670321000 |
| H | 3.883402000 | 4.936417000 | -0.821564000 |
| H | 0.506905000 | -4.103229000 | 0.640993000 |
| H | 2.810741000 | -5.533250000 | 0.864004000 |

Cartesian coordinates of DFT-optimized structure of **5box** complex by B3LYP/Def2-TZVP; 6-31G(d,p)/$CH_3CN$, charge = 2, multiplicity = 2

| | | | |
|---|---|---|---|
| Ru | 3.512508000 | 0.038942000 | 0.008739000 |
| N | 2.333041000 | -1.671329000 | 0.272136000 |
| N | 4.091112000 | -0.000304000 | 2.031093000 |
| N | 5.477444000 | -0.697434000 | -0.051659000 |
| N | 3.782630000 | -0.489703000 | -2.008322000 |
| N | -6.711598000 | 2.330284000 | 1.535086000 |
| C | 3.292682000 | 0.335511000 | 3.062199000 |
| H | 2.274635000 | 0.604801000 | 2.811380000 |
| C | 3.741984000 | 0.344812000 | 4.378758000 |

| | | | |
|---|---|---|---|
| H | 3.059817000 | 0.627220000 | 5.172201000 |
| C | 5.062050000 | -0.013256000 | 4.641948000 |
| H | 5.444483000 | -0.018332000 | 5.656939000 |
| C | 5.890085000 | -0.373093000 | 3.580703000 |
| H | 6.917637000 | -0.662914000 | 3.763837000 |
| C | 5.390523000 | -0.362640000 | 2.279468000 |
| C | 6.182873000 | -0.748350000 | 1.094476000 |
| C | 7.521414000 | -1.148141000 | 1.071571000 |
| H | 8.109033000 | -1.196647000 | 1.980051000 |
| C | 8.093074000 | -1.488049000 | -0.155860000 |
| C | 7.338966000 | -1.436541000 | -1.329280000 |
| H | 7.785537000 | -1.707712000 | -2.278111000 |
| C | 6.004353000 | -1.030880000 | -1.245300000 |
| C | 5.035900000 | -0.926222000 | -2.354682000 |
| C | 5.333215000 | -1.262006000 | -3.674563000 |
| H | 6.326161000 | -1.608593000 | -3.933960000 |
| C | 4.348269000 | -1.154681000 | -4.653936000 |
| H | 4.572918000 | -1.413072000 | -5.683176000 |
| C | 3.076802000 | -0.718124000 | -4.289288000 |
| H | 2.277680000 | -0.622885000 | -5.015257000 |
| C | 2.831948000 | -0.398876000 | -2.957719000 |
| H | 1.858907000 | -0.055553000 | -2.630017000 |
| C | 2.788417000 | -2.940458000 | 0.282221000 |
| H | 3.865408000 | -3.060479000 | 0.288120000 |
| C | 1.960760000 | -4.044304000 | 0.275095000 |
| H | 2.369149000 | -5.047716000 | 0.284611000 |
| C | 0.571668000 | -3.840712000 | 0.220504000 |
| C | 0.085267000 | -2.525831000 | 0.219445000 |
| H | -0.972835000 | -2.341202000 | 0.129944000 |
| C | 0.972193000 | -1.445531000 | 0.281113000 |
| C | -3.575653000 | 4.524508000 | -0.230641000 |
| C | 0.601404000 | -0.015509000 | 0.260871000 |
| C | -4.682023000 | 3.758517000 | 0.122880000 |
| C | 1.716749000 | 0.818023000 | 0.046432000 |
| C | -4.562981000 | 2.406331000 | 0.444868000 |
| C | 1.586670000 | 2.197729000 | -0.156057000 |
| C | -5.823888000 | 1.671219000 | 0.755661000 |
| C | -6.094530000 | 0.419053000 | 0.202442000 |
| H | -5.390229000 | -0.074452000 | -0.457565000 |
| C | -7.322904000 | -0.204746000 | 0.476201000 |
| C | -8.237911000 | 0.464363000 | 1.298628000 |
| H | -9.203574000 | 0.046221000 | 1.553052000 |
| C | -7.873333000 | 1.720833000 | 1.785128000 |
| H | -8.571222000 | 2.265597000 | 2.418892000 |
| C | -3.273922000 | 1.811707000 | 0.479933000 |
| C | 0.316826000 | 2.788219000 | -0.294649000 |
| C | -2.128428000 | 2.579089000 | 0.083175000 |
| C | -0.823568000 | 1.978385000 | 0.070047000 |
| C | -2.288198000 | 3.949501000 | -0.307816000 |
| C | -0.677445000 | 0.585932000 | 0.418300000 |
| C | -1.153687000 | 4.660330000 | -0.814681000 |

| | | | |
|---|---|---|---|
| C | -1.821833000 | -0.092224000 | 0.949400000 |
| H | -1.292790000 | 5.646798000 | -1.240487000 |
| H | -1.699780000 | -1.069485000 | 1.397267000 |
| C | 0.089591000 | 4.108418000 | -0.802753000 |
| C | -3.053675000 | 0.488540000 | 0.979082000 |
| H | 0.907872000 | 4.659607000 | -1.247447000 |
| H | -3.882794000 | -0.052559000 | 1.418841000 |
| O | -0.191633000 | -4.939782000 | 0.164700000 |
| O | -7.522723000 | -1.410819000 | -0.094404000 |
| C | -8.777443000 | -2.083004000 | 0.118068000 |
| C | -8.772537000 | -3.419027000 | -0.639019000 |
| C | -10.137172000 | -4.087869000 | -0.382148000 |
| C | -7.643733000 | -4.323693000 | -0.109445000 |
| C | -8.590988000 | -3.172209000 | -2.148552000 |
| C | -1.629068000 | -4.818694000 | 0.111545000 |
| C | -2.246760000 | -6.223268000 | 0.062530000 |
| C | -3.776266000 | -6.039894000 | 0.011874000 |
| C | -1.769230000 | -6.966403000 | -1.199505000 |
| C | -1.859805000 | -7.016892000 | 1.324943000 |
| H | -9.592220000 | -1.441163000 | -0.241704000 |
| H | -8.915822000 | -2.253216000 | 1.193710000 |
| H | -10.963270000 | -3.465771000 | -0.747615000 |
| H | -10.297725000 | -4.272770000 | 0.686990000 |
| H | -10.188959000 | -5.051958000 | -0.900383000 |
| H | -7.763973000 | -4.517544000 | 0.963712000 |
| H | -6.661307000 | -3.865818000 | -0.263852000 |
| H | -7.653676000 | -5.289366000 | -0.628110000 |
| H | -9.393991000 | -2.538038000 | -2.544203000 |
| H | -8.612748000 | -4.122007000 | -2.695406000 |
| H | -7.635565000 | -2.680739000 | -2.358413000 |
| H | -1.970715000 | -4.275542000 | 1.001633000 |
| H | -1.904404000 | -4.245027000 | -0.782289000 |
| H | -4.271917000 | -7.016503000 | -0.022890000 |
| H | -4.084157000 | -5.476367000 | -0.877241000 |
| H | -4.145778000 | -5.508304000 | 0.897216000 |
| H | -2.047791000 | -6.419807000 | -2.108939000 |
| H | -2.226846000 | -7.960906000 | -1.251572000 |
| H | -0.681846000 | -7.093289000 | -1.198463000 |
| H | -2.201836000 | -6.506101000 | 2.233516000 |
| H | -0.775214000 | -7.147659000 | 1.395831000 |
| H | -2.320942000 | -8.010960000 | 1.304763000 |
| H | -5.658564000 | 4.225567000 | 0.145164000 |
| H | 9.131155000 | -1.799189000 | -0.197416000 |
| N | 2.842359000 | 2.869223000 | -0.240842000 |
| C | 3.218142000 | 4.176327000 | -0.161402000 |
| C | 4.599781000 | 4.228446000 | -0.180308000 |
| C | 5.019529000 | 2.892174000 | -0.248388000 |
| N | 3.965879000 | 2.069321000 | -0.272825000 |
| N | -3.790475000 | 5.896518000 | -0.533509000 |
| C | -3.126721000 | 6.988127000 | -0.038360000 |
| C | -3.765165000 | 8.104803000 | -0.532619000 |

| | | | |
|---|---|---|---|
| C | -4.823593000 | 7.590169000 | -1.317088000 |
| N | -4.848348000 | 6.259573000 | -1.314142000 |
| H | -2.288745000 | 6.879605000 | 0.633776000 |
| H | -3.510263000 | 9.138070000 | -0.347786000 |
| H | -5.565056000 | 8.135515000 | -1.887095000 |
| H | 6.020106000 | 2.484619000 | -0.273387000 |
| H | 5.216469000 | 5.113123000 | -0.142816000 |
| H | 2.495979000 | 4.969185000 | -0.066560000 |

Cartesian coordinates of DFT-optimized structure of **6box** complex by B3LYP/Def2-TZVP; 6-31G(d,p)/$CH_3CN$, charge = 2, multiplicity = 2

| | | | |
|---|---|---|---|
| Ru | -2.671352000 | -1.551615000 | -0.037221000 |
| N | -3.353087000 | 0.404798000 | 0.280176000 |
| N | -2.935753000 | -2.008415000 | 1.998456000 |
| N | -4.424501000 | -2.706193000 | -0.011491000 |
| N | -3.358485000 | -1.455961000 | -2.022089000 |
| C | -2.139997000 | -1.566610000 | 2.991113000 |
| H | -1.333514000 | -0.905681000 | 2.700058000 |
| C | -2.331207000 | -1.936465000 | 4.318402000 |
| H | -1.659139000 | -1.556320000 | 5.078958000 |
| C | -3.387825000 | -2.787156000 | 4.634813000 |
| H | -3.566206000 | -3.094134000 | 5.659748000 |
| C | -4.222292000 | -3.237873000 | 3.614178000 |
| H | -5.055349000 | -3.892875000 | 3.838817000 |
| C | -3.984514000 | -2.839397000 | 2.299719000 |
| C | -4.825641000 | -3.245089000 | 1.155926000 |
| C | -5.942987000 | -4.083105000 | 1.189136000 |
| H | -6.284750000 | -4.527602000 | 2.115801000 |
| C | -6.620706000 | -4.335829000 | -0.004818000 |
| C | -6.191430000 | -3.758384000 | -1.200710000 |
| H | -6.726254000 | -3.949888000 | -2.122865000 |
| C | -5.066052000 | -2.930419000 | -1.174133000 |
| C | -4.462069000 | -2.215390000 | -2.316049000 |
| C | -4.964730000 | -2.267670000 | -3.615377000 |
| H | -5.835659000 | -2.873334000 | -3.834727000 |
| C | -4.344679000 | -1.536453000 | -4.626257000 |
| H | -4.729920000 | -1.572881000 | -5.639539000 |
| C | -3.232448000 | -0.758469000 | -4.313298000 |
| H | -2.720443000 | -0.168707000 | -5.064771000 |
| C | -2.771140000 | -0.742058000 | -3.001084000 |
| H | -1.909112000 | -0.153514000 | -2.713605000 |
| C | -4.644892000 | 0.782749000 | 0.363499000 |
| H | -5.375419000 | -0.016939000 | 0.400136000 |
| C | -5.050090000 | 2.101611000 | 0.392921000 |
| H | -6.099807000 | 2.360596000 | 0.461782000 |
| C | -4.073009000 | 3.106578000 | 0.295592000 |
| C | -2.725800000 | 2.726567000 | 0.216426000 |
| H | -1.958992000 | 3.473914000 | 0.091677000 |
| C | -2.371983000 | 1.373548000 | 0.247802000 |

| | | | |
|---|---|---|---|
| C | 5.119478000 | 1.570749000 | -0.610517000 |
| C | -0.999772000 | 0.835433000 | 0.152222000 |
| C | 5.134587000 | 2.922190000 | -0.259160000 |
| C | -0.988380000 | -0.553565000 | -0.083132000 |
| C | 3.980554000 | 3.581919000 | 0.147344000 |
| C | 0.195497000 | -1.252582000 | -0.350967000 |
| C | 2.747976000 | 2.896874000 | 0.247661000 |
| C | 1.411370000 | -0.566795000 | -0.533815000 |
| C | 2.691047000 | 1.530050000 | -0.179359000 |
| C | 1.439535000 | 0.825663000 | -0.148624000 |
| C | 3.886033000 | 0.871000000 | -0.624799000 |
| C | 0.242360000 | 1.516094000 | 0.266204000 |
| C | 3.767819000 | -0.458873000 | -1.145778000 |
| C | 0.384836000 | 2.828904000 | 0.822508000 |
| H | 4.629410000 | -0.918714000 | -1.614300000 |
| H | -0.459343000 | 3.288990000 | 1.318837000 |
| C | 2.593881000 | -1.145316000 | -1.098006000 |
| C | 1.576350000 | 3.488009000 | 0.815457000 |
| H | 2.544636000 | -2.124068000 | -1.556959000 |
| H | 1.645897000 | 4.466266000 | 1.276038000 |
| O | -4.513732000 | 4.371267000 | 0.279047000 |
| C | -3.572117000 | 5.460449000 | 0.171438000 |
| C | -4.336927000 | 6.790880000 | 0.208558000 |
| C | -3.288332000 | 7.914158000 | 0.087395000 |
| C | -5.322165000 | 6.868756000 | -0.973155000 |
| C | -5.095065000 | 6.928464000 | 1.542582000 |
| H | -2.863034000 | 5.399859000 | 1.006476000 |
| H | -3.020310000 | 5.358661000 | -0.771372000 |
| H | -3.780581000 | 8.892835000 | 0.108217000 |
| H | -2.728312000 | 7.840651000 | -0.852811000 |
| H | -2.569755000 | 7.882753000 | 0.915347000 |
| H | -4.796662000 | 6.783113000 | -1.932333000 |
| H | -5.851354000 | 7.828513000 | -0.964544000 |
| H | -6.068831000 | 6.069737000 | -0.921710000 |
| H | -4.406098000 | 6.883697000 | 2.395090000 |
| H | -5.836926000 | 6.132390000 | 1.662815000 |
| H | -5.619326000 | 7.890004000 | 1.584505000 |
| H | -7.491330000 | -4.982552000 | -0.002081000 |
| N | -0.000025000 | -2.662542000 | -0.442246000 |
| C | 0.842011000 | -3.733813000 | -0.425826000 |
| C | 0.074784000 | -4.883744000 | -0.417420000 |
| C | -1.255718000 | -4.440761000 | -0.404287000 |
| N | -1.306646000 | -3.104793000 | -0.407084000 |
| H | -2.171242000 | -5.014447000 | -0.386488000 |
| H | 0.431744000 | -5.902058000 | -0.414486000 |
| H | 1.911367000 | -3.615168000 | -0.391254000 |
| N | 4.086596000 | 4.958885000 | 0.482046000 |
| N | 5.139721000 | 5.394704000 | 1.231366000 |
| C | 4.998062000 | 6.717178000 | 1.274209000 |
| C | 3.867216000 | 7.155521000 | 0.546411000 |
| C | 3.309428000 | 5.999486000 | 0.045263000 |

| | | | |
|---|---|---|---|
| C | 6.428599000 | 0.949767000 | -0.972185000 |
| C | 6.817687000 | -0.283645000 | -0.426253000 |
| C | 8.081639000 | -0.794936000 | -0.749682000 |
| C | 8.902558000 | -0.045309000 | -1.606439000 |
| C | 8.426929000 | 1.170552000 | -2.076594000 |
| N | 7.221664000 | 1.680236000 | -1.777151000 |
| O | 8.586410000 | -1.958654000 | -0.291287000 |
| C | 7.797750000 | -2.770284000 | 0.596720000 |
| C | 8.600105000 | -4.022496000 | 0.980565000 |
| C | 7.713563000 | -4.856421000 | 1.925959000 |
| C | 8.933296000 | -4.844102000 | -0.279236000 |
| C | 9.896283000 | -3.616699000 | 1.707508000 |
| H | 5.711288000 | 7.311590000 | 1.831020000 |
| H | 3.514795000 | 8.166318000 | 0.401998000 |
| H | 2.457110000 | 5.833994000 | -0.596494000 |
| H | 6.156246000 | -0.806079000 | 0.252125000 |
| H | 9.886566000 | -0.413172000 | -1.876136000 |
| H | 9.049878000 | 1.774331000 | -2.733903000 |
| H | 6.864074000 | -3.051574000 | 0.091879000 |
| H | 7.548775000 | -2.186355000 | 1.492320000 |
| H | 7.455509000 | -4.295483000 | 2.832582000 |
| H | 8.240967000 | -5.766151000 | 2.234408000 |
| H | 6.780022000 | -5.160012000 | 1.436544000 |
| H | 8.019450000 | -5.154503000 | -0.801010000 |
| H | 9.489577000 | -5.749230000 | -0.009276000 |
| H | 9.544455000 | -4.265887000 | -0.979539000 |
| H | 9.676579000 | -3.038502000 | 2.613693000 |
| H | 10.538632000 | -3.007303000 | 1.063818000 |
| H | 10.461227000 | -4.507236000 | 2.006462000 |
| H | 6.067656000 | 3.469981000 | -0.297639000 |

Cartesian coordinates of DFT-optimized structure of **7a** complex by B3LYP/Def2-TZVP; 6-31G(d,p)/$CH_3CN$, charge = 2, multiplicity = 1

| | | | |
|---|---|---|---|
| Os | -5.552880000 | -0.049698000 | 0.032965000 |
| Os | 5.543806000 | 0.094285000 | 0.000328000 |
| N | -5.081891000 | -2.089341000 | 0.290784000 |
| N | 5.072890000 | 2.134032000 | -0.256985000 |
| N | -6.014375000 | 0.241079000 | 2.055692000 |
| N | 6.003229000 | -0.196598000 | -2.022826000 |
| N | -7.597146000 | -0.080488000 | 0.023406000 |
| N | 7.588113000 | 0.124734000 | 0.007854000 |
| N | -5.986108000 | -0.351662000 | -1.995803000 |
| N | 5.979145000 | 0.396220000 | 2.028648000 |
| N | -5.141320000 | 2.003401000 | -0.220231000 |
| N | 5.132162000 | -1.958734000 | 0.253926000 |
| C | -5.138178000 | 0.404028000 | 3.074985000 |
| C | 5.125982000 | -0.359410000 | -3.041244000 |
| H | 4.079959000 | -0.329750000 | -2.762120000 |
| C | -5.535314000 | 0.596683000 | 4.390275000 |

| | | | |
|---|---|---|---|
| C | 5.521763000 | -0.552221000 | -4.356919000 |
| H | 4.768623000 | -0.676914000 | -5.127219000 |
| C | -6.899542000 | 0.626338000 | 4.690006000 |
| C | 6.885685000 | -0.582218000 | -4.658004000 |
| C | -7.813693000 | 0.461156000 | 3.655819000 |
| C | 7.800897000 | -0.417171000 | -3.624735000 |
| H | -8.877807000 | 0.480668000 | 3.860621000 |
| C | -7.365940000 | 0.269303000 | 2.345453000 |
| C | 7.354501000 | -0.225116000 | -2.313941000 |
| C | -8.267537000 | 0.084735000 | 1.198779000 |
| C | 8.257285000 | -0.040641000 | -1.168192000 |
| C | -9.663488000 | 0.061623000 | 1.212459000 |
| C | 9.653224000 | -0.017705000 | -1.183284000 |
| H | -10.212921000 | 0.190746000 | 2.137815000 |
| H | 10.201714000 | -0.146909000 | -2.109190000 |
| C | -10.350574000 | -0.132349000 | 0.011645000 |
| C | 10.341553000 | 0.176212000 | 0.016829000 |
| C | -9.646599000 | -0.300285000 | -1.183207000 |
| C | 9.638814000 | 0.344272000 | 1.212389000 |
| C | -8.250833000 | -0.271075000 | -1.157300000 |
| C | 8.243020000 | 0.315269000 | 1.187883000 |
| C | -7.333699000 | -0.424443000 | -2.296081000 |
| C | 7.327047000 | 0.468827000 | 2.327573000 |
| C | -7.765014000 | -0.628769000 | -3.609988000 |
| C | 7.759726000 | 0.673161000 | 3.641029000 |
| H | -8.826369000 | -0.683291000 | -3.822500000 |
| C | -6.838305000 | -0.761893000 | -4.637575000 |
| C | 6.834084000 | 0.806450000 | 4.669552000 |
| C | -5.478233000 | -0.686097000 | -4.327507000 |
| C | 5.473689000 | 0.730810000 | 4.360868000 |
| H | 4.712533000 | 0.828546000 | 5.127200000 |
| C | -5.097584000 | -0.482231000 | -3.008929000 |
| C | 5.091674000 | 0.526949000 | 3.042683000 |
| H | 4.048845000 | 0.461553000 | 2.758029000 |
| C | -5.979308000 | -3.102057000 | 0.323996000 |
| C | 5.970365000 | 3.146687000 | -0.290453000 |
| H | -7.022450000 | -2.808149000 | 0.334872000 |
| H | 7.013476000 | 2.852696000 | -0.301940000 |
| C | -5.631982000 | -4.436331000 | 0.332702000 |
| C | 5.623128000 | 4.480989000 | -0.298711000 |
| H | -6.390252000 | -5.210075000 | 0.360707000 |
| H | 6.381440000 | 5.254684000 | -0.326952000 |
| C | -4.268623000 | -4.772965000 | 0.268836000 |
| C | 4.259826000 | 4.817716000 | -0.234107000 |
| C | -3.326662000 | -3.741783000 | 0.240455000 |
| C | 3.317803000 | 3.786596000 | -0.205438000 |
| H | -2.277913000 | -3.970009000 | 0.138647000 |
| H | 2.269116000 | 4.014859000 | -0.103048000 |
| C | -3.730919000 | -2.397447000 | 0.293090000 |
| C | 3.721950000 | 2.442246000 | -0.258512000 |
| C | -2.843339000 | -1.227333000 | 0.249900000 |

| | | | |
|---|---|---|---|
| C | 2.834291000 | 1.272208000 | -0.214947000 |
| C | -3.557619000 | -0.023480000 | 0.035636000 |
| C | 3.548546000 | 0.068300000 | -0.000889000 |
| C | -2.876373000 | 1.197367000 | -0.186295000 |
| C | 2.867274000 | -1.152495000 | 0.221232000 |
| C | -3.792130000 | 2.346395000 | -0.226822000 |
| C | 3.782940000 | -2.301606000 | 0.261352000 |
| C | -3.433585000 | 3.695116000 | -0.175476000 |
| C | 3.424256000 | -3.650308000 | 0.210421000 |
| H | -2.397919000 | 3.989117000 | -0.084159000 |
| H | 2.388514000 | -3.944244000 | 0.119777000 |
| C | -4.400701000 | 4.707674000 | -0.196016000 |
| C | 4.391298000 | -4.662943000 | 0.230546000 |
| C | -5.753361000 | 4.341726000 | -0.254365000 |
| C | 5.744025000 | -4.297105000 | 0.288037000 |
| H | -6.556551000 | 5.065932000 | -0.279495000 |
| H | 6.547167000 | -5.021376000 | 0.312799000 |
| C | -6.058655000 | 2.989491000 | -0.248119000 |
| C | 6.049429000 | -2.944898000 | 0.281414000 |
| H | -7.094478000 | 2.670267000 | -0.255393000 |
| H | 7.085284000 | -2.625762000 | 0.288017000 |
| C | -1.463300000 | 1.195122000 | -0.344655000 |
| C | 1.454226000 | -1.150148000 | 0.379883000 |
| C | -0.724643000 | 0.012630000 | 0.021123000 |
| C | 0.715571000 | 0.032363000 | 0.014172000 |
| C | -1.428196000 | -1.188346000 | 0.394866000 |
| C | 1.419127000 | 1.233323000 | -0.359618000 |
| C | -0.651659000 | -2.248665000 | 0.961822000 |
| C | 0.642591000 | 2.293707000 | -0.926455000 |
| H | -1.154458000 | -3.063333000 | 1.468484000 |
| H | 1.145399000 | 3.108427000 | -1.433025000 |
| C | 0.712758000 | -2.231337000 | 0.953366000 |
| C | -0.721822000 | 2.276370000 | -0.918020000 |
| H | 1.243719000 | -3.034103000 | 1.450066000 |
| H | -1.252778000 | 3.079172000 | -1.414666000 |
| O | -3.976122000 | -6.087651000 | 0.230525000 |
| O | 3.931449000 | -5.928776000 | 0.182491000 |
| O | 3.967441000 | 6.132414000 | -0.195395000 |
| O | -3.940980000 | 5.973532000 | -0.147481000 |
| C | -4.884019000 | 7.061854000 | -0.147151000 |
| C | -4.121592000 | 8.392727000 | -0.075958000 |
| C | -5.176361000 | 9.516575000 | -0.072331000 |
| C | -3.205962000 | 8.544306000 | -1.305416000 |
| C | -3.289010000 | 8.458555000 | 1.218371000 |
| C | 2.592634000 | 6.550619000 | -0.118188000 |
| C | 2.530402000 | 8.085106000 | -0.132206000 |
| C | 1.043992000 | 8.478123000 | -0.027164000 |
| C | 3.305512000 | 8.658313000 | 1.069292000 |
| C | 3.119402000 | 8.624282000 | -1.449609000 |
| C | -2.601263000 | -6.505773000 | 0.153867000 |
| C | -2.538936000 | -8.040255000 | 0.168168000 |

| | | | |
|---|---|---|---|
| C | -3.128312000 | -8.579233000 | 1.485486000 |
| C | -3.313645000 | -8.613721000 | -1.033465000 |
| C | -1.052474000 | -8.433206000 | 0.063655000 |
| C | 4.874418000 | -7.017158000 | 0.181795000 |
| C | 4.111868000 | -8.348008000 | 0.111498000 |
| C | 3.278413000 | -8.414165000 | -1.182251000 |
| C | 3.197067000 | -8.499187000 | 1.341622000 |
| C | 5.166579000 | -9.471909000 | 0.107481000 |
| H | -5.552752000 | 6.955185000 | 0.716771000 |
| H | -5.485484000 | 7.014088000 | -1.064171000 |
| H | -5.844997000 | 9.435380000 | 0.793268000 |
| H | -5.790624000 | 9.492966000 | -0.980671000 |
| H | -4.685646000 | 10.495241000 | -0.025607000 |
| H | -3.785754000 | 8.509792000 | -2.236215000 |
| H | -2.455646000 | 7.747718000 | -1.343367000 |
| H | -2.679706000 | 9.505320000 | -1.273012000 |
| H | -3.928759000 | 8.364126000 | 2.104650000 |
| H | -2.762886000 | 9.417865000 | 1.284696000 |
| H | -2.542344000 | 7.658559000 | 1.252208000 |
| H | 2.043488000 | 6.137345000 | -0.974280000 |
| H | 2.150147000 | 6.155759000 | 0.805758000 |
| H | 0.939956000 | 9.568950000 | -0.038076000 |
| H | 0.596076000 | 8.108416000 | 0.903344000 |
| H | 0.462562000 | 8.078355000 | -0.866960000 |
| H | 2.894673000 | 8.289085000 | 2.017161000 |
| H | 3.239697000 | 9.752469000 | 1.078035000 |
| H | 4.363885000 | 8.381862000 | 1.027233000 |
| H | 2.567731000 | 8.238344000 | -2.315923000 |
| H | 4.170053000 | 8.337360000 | -1.560682000 |
| H | 3.061258000 | 9.718530000 | -1.474678000 |
| H | -2.052438000 | -6.092323000 | 1.010080000 |
| H | -2.158485000 | -6.111029000 | -0.769991000 |
| H | -2.576919000 | -8.193120000 | 2.351899000 |
| H | -3.070121000 | -9.673474000 | 1.510758000 |
| H | -4.179009000 | -8.292340000 | 1.596190000 |
| H | -4.372051000 | -8.337342000 | -0.991766000 |
| H | -3.247745000 | -9.707874000 | -1.042005000 |
| H | -2.902554000 | -8.244622000 | -1.981275000 |
| H | -0.471313000 | -8.033224000 | 0.903535000 |
| H | -0.604302000 | -8.063676000 | -0.866800000 |
| H | -0.948376000 | -9.524024000 | 0.074834000 |
| H | 5.476523000 | -6.969188000 | 1.098383000 |
| H | 5.542556000 | -6.910756000 | -0.682620000 |
| H | 2.752172000 | -9.373454000 | -1.247938000 |
| H | 3.917579000 | -8.320047000 | -2.068984000 |
| H | 2.531784000 | -7.614122000 | -1.215828000 |
| H | 2.446818000 | -7.702551000 | 1.379859000 |
| H | 3.777497000 | -8.464435000 | 2.272015000 |
| H | 2.670739000 | -9.460184000 | 1.309856000 |
| H | 5.834632000 | -9.390990000 | -0.758594000 |
| H | 4.675786000 | -10.450565000 | 0.061366000 |

| | | | |
|---|---|---|---|
| H | 5.781456000 | -9.448073000 | 1.015400000 |
| H | -4.782957000 | 0.721505000 | 5.161334000 |
| H | -7.243948000 | 0.775159000 | 5.707956000 |
| H | -4.091863000 | 0.374607000 | 2.796880000 |
| H | -10.182285000 | -0.449986000 | -2.113565000 |
| H | -11.434977000 | -0.152566000 | 0.007113000 |
| H | -7.169910000 | -0.920868000 | -5.658239000 |
| H | -4.716274000 | -0.783711000 | -5.093073000 |
| H | -4.055049000 | -0.416690000 | -2.723172000 |
| H | 7.229052000 | -0.731199000 | -5.676282000 |
| H | 8.864815000 | -0.436976000 | -3.830590000 |
| H | 11.425962000 | 0.196288000 | 0.020256000 |
| H | 10.175450000 | 0.493896000 | 2.142210000 |
| H | 8.821317000 | 0.727561000 | 3.852458000 |
| H | 7.166750000 | 0.965431000 | 5.689870000 |

Cartesian coordinates of DFT-optimized structure of **8a** complex by B3LYP/Def2-TZVP; 6-31G(d,p)/$CH_3CN$, charge = 2, multiplicity = 1

| | | | |
|---|---|---|---|
| Os | 5.467858000 | 0.958425000 | 0.034981000 |
| N | 5.331113000 | -1.103713000 | 0.373277000 |
| N | 5.881237000 | 1.398070000 | 2.046569000 |
| N | 7.487346000 | 1.284181000 | 0.000963000 |
| N | 5.932627000 | 0.657012000 | -1.991054000 |
| C | 5.000725000 | 1.439116000 | 3.072286000 |
| H | 3.973340000 | 1.213896000 | 2.813640000 |
| C | 5.370093000 | 1.745618000 | 4.374970000 |
| H | 4.614990000 | 1.762590000 | 5.153158000 |
| C | 6.709480000 | 2.025824000 | 4.651360000 |
| H | 7.032447000 | 2.269509000 | 5.657945000 |
| C | 7.628862000 | 1.986407000 | 3.608405000 |
| H | 8.674886000 | 2.198731000 | 3.796358000 |
| C | 7.208491000 | 1.672850000 | 2.313340000 |
| C | 8.120954000 | 1.608889000 | 1.159410000 |
| C | 9.498508000 | 1.837658000 | 1.156692000 |
| H | 10.021740000 | 2.097257000 | 2.069509000 |
| C | 10.200149000 | 1.726078000 | -0.045752000 |
| C | 9.530300000 | 1.390838000 | -1.224568000 |
| H | 10.078033000 | 1.306294000 | -2.155891000 |
| C | 8.152141000 | 1.170373000 | -1.179742000 |
| C | 7.269418000 | 0.812580000 | -2.302559000 |
| C | 7.725189000 | 0.635459000 | -3.611351000 |
| H | 8.778190000 | 0.761798000 | -3.834345000 |
| C | 6.832187000 | 0.297874000 | -4.622886000 |
| H | 7.183030000 | 0.158831000 | -5.639983000 |
| C | 5.482517000 | 0.143393000 | -4.301258000 |
| H | 4.746752000 | -0.118785000 | -5.053565000 |
| C | 5.077548000 | 0.330250000 | -2.986552000 |
| H | 4.040684000 | 0.221609000 | -2.692872000 |
| N | 4.034848000 | -1.601731000 | 0.403409000 |

| | | | |
|---|---|---|---|
| C | -3.005598000 | 0.779702000 | -0.179813000 |
| C | 2.983524000 | -0.640510000 | 0.292540000 |
| C | -3.512164000 | -0.504904000 | 0.040960000 |
| C | 3.492025000 | 0.641438000 | 0.061046000 |
| C | -2.622824000 | -1.558077000 | 0.276461000 |
| C | 2.603613000 | 1.697734000 | -0.163718000 |
| C | -1.233511000 | -1.349238000 | 0.411573000 |
| C | 1.212253000 | 1.493814000 | -0.283889000 |
| C | -0.720249000 | -0.043172000 | 0.063733000 |
| C | 0.698499000 | 0.189031000 | 0.067805000 |
| C | -1.621176000 | 1.030078000 | -0.292021000 |
| C | 1.599390000 | -0.885588000 | 0.419698000 |
| C | -1.053962000 | 2.244847000 | -0.791606000 |
| C | 1.032015000 | -2.097846000 | 0.925168000 |
| H | -1.693828000 | 2.987895000 | -1.249438000 |
| H | 1.671785000 | -2.838982000 | 1.386131000 |
| C | 0.290181000 | 2.464838000 | -0.787748000 |
| C | -0.312117000 | -2.317835000 | 0.921312000 |
| H | 0.662333000 | 3.373520000 | -1.242674000 |
| H | -0.684804000 | -3.224678000 | 1.379368000 |
| H | 11.270464000 | 1.900699000 | -0.064226000 |
| N | -3.313474000 | -2.804398000 | 0.382347000 |
| N | -4.700682000 | -2.745564000 | 0.344551000 |
| C | -5.134439000 | -4.010577000 | 0.382079000 |
| C | -4.052162000 | -4.903215000 | 0.423284000 |
| C | -2.920300000 | -4.110954000 | 0.409431000 |
| H | -6.195882000 | -4.210785000 | 0.364748000 |
| H | -4.087566000 | -5.981802000 | 0.450424000 |
| H | -1.881796000 | -4.390253000 | 0.382552000 |
| Os | -5.485784000 | -0.834304000 | 0.003612000 |
| C | -5.668234000 | -0.060947000 | 4.343349000 |
| C | -5.213182000 | -0.232522000 | 3.043023000 |
| N | -6.028941000 | -0.551216000 | 2.012462000 |
| C | -7.376003000 | -0.714155000 | 2.271597000 |
| C | -7.881602000 | -0.552143000 | 3.564017000 |
| C | -7.028579000 | -0.222617000 | 4.612031000 |
| C | -8.214026000 | -1.064637000 | 1.112671000 |
| N | -7.503527000 | -1.169334000 | -0.041940000 |
| C | -8.090768000 | -1.488080000 | -1.226217000 |
| C | -9.467021000 | -1.718479000 | -1.278907000 |
| C | -10.215341000 | -1.615373000 | -0.104140000 |
| C | -9.592589000 | -1.287008000 | 1.102084000 |
| C | -7.133812000 | -1.544737000 | -2.343788000 |
| N | -5.818934000 | -1.266298000 | -2.024712000 |
| C | -4.898779000 | -1.302473000 | -3.015145000 |
| C | -5.215732000 | -1.607458000 | -4.331918000 |
| C | -6.542417000 | -1.891020000 | -4.661189000 |
| C | -7.502053000 | -1.856695000 | -3.654939000 |
| H | -4.166182000 | -0.118221000 | 2.790301000 |
| H | -4.962284000 | 0.195171000 | 5.125709000 |
| H | -7.417975000 | -0.095519000 | 5.616581000 |

| | | | |
|---|---|---|---|
| H | -8.941763000 | -0.684549000 | 3.745950000 |
| H | -10.176347000 | -1.208610000 | 2.011743000 |
| H | -11.285352000 | -1.791100000 | -0.128719000 |
| H | -9.954031000 | -1.972401000 | -2.213182000 |
| H | -8.539130000 | -2.072438000 | -3.883902000 |
| H | -6.824967000 | -2.133704000 | -5.680100000 |
| H | -4.430606000 | -1.620686000 | -5.079886000 |
| H | -3.883034000 | -1.075160000 | -2.715600000 |
| N | -4.059145000 | 1.735974000 | -0.309265000 |
| N | -5.352893000 | 1.230925000 | -0.317577000 |
| C | -6.171455000 | 2.287923000 | -0.368841000 |
| C | -5.435698000 | 3.483182000 | -0.373200000 |
| C | -4.109293000 | 3.099629000 | -0.321937000 |
| N | 4.685323000 | 2.873825000 | -0.288825000 |
| H | -3.218127000 | 3.699053000 | -0.259305000 |
| H | -5.817589000 | 4.492510000 | -0.400702000 |
| H | -7.240562000 | 2.133692000 | -0.386928000 |
| N | 3.298047000 | 2.940089000 | -0.288171000 |
| C | 5.124495000 | 4.136669000 | -0.336459000 |
| C | 4.046132000 | 5.035024000 | -0.345976000 |
| C | 2.910937000 | 4.248595000 | -0.301768000 |
| C | 4.078552000 | -2.965487000 | 0.429484000 |
| C | 5.403826000 | -3.355603000 | 0.450371000 |
| C | 6.145289000 | -2.164484000 | 0.414373000 |
| H | 4.086488000 | 6.113460000 | -0.372233000 |
| H | 1.874843000 | 4.532600000 | -0.244664000 |
| H | 6.187073000 | 4.331318000 | -0.348443000 |
| H | 7.215257000 | -2.015865000 | 0.403145000 |
| H | 5.781076000 | -4.366652000 | 0.478692000 |
| H | 3.183428000 | -3.561242000 | 0.397079000 |

Cartesian coordinates of DFT-optimized structure of **9a** complex by B3LYP/Def2-TZVP; 6-31G(d,p)/$CH_3CN$, charge = 2, multiplicity = 1

| | | | |
|---|---|---|---|
| Os | 5.546537000 | 0.888252000 | -0.247746000 |
| N | 5.423105000 | -1.175463000 | 0.171897000 |
| N | 5.966133000 | 1.407112000 | 1.738836000 |
| N | 7.569334000 | 1.190367000 | -0.291846000 |
| N | 6.012967000 | 0.506669000 | -2.255093000 |
| C | 5.081098000 | 1.505440000 | 2.758781000 |
| H | 4.051790000 | 1.286137000 | 2.503205000 |
| C | 5.449215000 | 1.861475000 | 4.048236000 |
| H | 4.690913000 | 1.922818000 | 4.821128000 |
| C | 6.792137000 | 2.134134000 | 4.319964000 |
| H | 7.113707000 | 2.415211000 | 5.317232000 |
| C | 7.714820000 | 2.039193000 | 3.284549000 |
| H | 8.762705000 | 2.246769000 | 3.467714000 |
| C | 7.296639000 | 1.676325000 | 2.000897000 |
| C | 8.210036000 | 1.552370000 | 0.855354000 |
| C | 9.591029000 | 1.758169000 | 0.845649000 |

| | | | |
|---|---|---|---|
| H | 10.116823000 | 2.045930000 | 1.748695000 |
| C | 10.293810000 | 1.587143000 | -0.349540000 |
| C | 9.620022000 | 1.216075000 | -1.515657000 |
| H | 10.167859000 | 1.084301000 | -2.441586000 |
| C | 8.238429000 | 1.019868000 | -1.466842000 |
| C | 7.352109000 | 0.633476000 | -2.574372000 |
| C | 7.802703000 | 0.404890000 | -3.877851000 |
| H | 8.856975000 | 0.510358000 | -4.106009000 |
| C | 6.904140000 | 0.044478000 | -4.875402000 |
| H | 7.250752000 | -0.133408000 | -5.887910000 |
| C | 5.552476000 | -0.082604000 | -4.545874000 |
| H | 4.812371000 | -0.362404000 | -5.287580000 |
| C | 5.151960000 | 0.154307000 | -3.238814000 |
| H | 4.115076000 | 0.067130000 | -2.938687000 |
| C | 6.477135000 | -2.019488000 | 0.263518000 |
| H | 7.456025000 | -1.554887000 | 0.239480000 |
| C | 6.357108000 | -3.388784000 | 0.370098000 |
| H | 7.233476000 | -4.022410000 | 0.439844000 |
| C | 5.068512000 | -3.950447000 | 0.349940000 |
| C | 3.967068000 | -3.094844000 | 0.269454000 |
| H | 2.969878000 | -3.500082000 | 0.204154000 |
| C | 4.142520000 | -1.701880000 | 0.220305000 |
| C | -2.922919000 | 0.731188000 | -0.513190000 |
| C | 3.073417000 | -0.700508000 | 0.108858000 |
| C | -3.441379000 | -0.505409000 | -0.115572000 |
| C | 3.575571000 | 0.584582000 | -0.209847000 |
| C | -2.562099000 | -1.523144000 | 0.267087000 |
| C | 2.699089000 | 1.655296000 | -0.508337000 |
| C | -1.169169000 | -1.312189000 | 0.367785000 |
| C | 1.306426000 | 1.406199000 | -0.643413000 |
| C | -0.645198000 | -0.069582000 | -0.149279000 |
| C | 0.776561000 | 0.150574000 | -0.169126000 |
| C | -1.534717000 | 0.953748000 | -0.647127000 |
| C | 1.674122000 | -0.882984000 | 0.285850000 |
| C | -0.950094000 | 2.087228000 | -1.294649000 |
| C | 1.095217000 | -2.006125000 | 0.958052000 |
| H | -1.576511000 | 2.765092000 | -1.859902000 |
| H | 1.736474000 | -2.684580000 | 1.506882000 |
| C | 0.396140000 | 2.302264000 | -1.289279000 |
| C | -0.252396000 | -2.209699000 | 0.999443000 |
| H | 0.786922000 | 3.145210000 | -1.845316000 |
| H | -0.628963000 | -3.039809000 | 1.582992000 |
| O | 5.000019000 | -5.294988000 | 0.401991000 |
| C | 3.714713000 | -5.941326000 | 0.371663000 |
| C | 3.913272000 | -7.461589000 | 0.460126000 |
| C | 2.513347000 | -8.104476000 | 0.414563000 |
| C | 4.751247000 | -7.956727000 | -0.733945000 |
| C | 4.609857000 | -7.826701000 | 1.784728000 |
| H | 3.113067000 | -5.582454000 | 1.217072000 |
| H | 3.201686000 | -5.674630000 | -0.561573000 |
| H | 2.596696000 | -9.195187000 | 0.478968000 |

| | | | |
|---|---|---|---|
| H | 1.991579000 | -7.863050000 | -0.519526000 |
| H | 1.889154000 | -7.766185000 | 1.250631000 |
| H | 4.267873000 | -7.707105000 | -1.686680000 |
| H | 4.868133000 | -9.045836000 | -0.691794000 |
| H | 5.749651000 | -7.507835000 | -0.731859000 |
| H | 4.018261000 | -7.493522000 | 2.646572000 |
| H | 5.599727000 | -7.364213000 | 1.852765000 |
| H | 4.735436000 | -8.912779000 | 1.863159000 |
| H | 11.366884000 | 1.743202000 | -0.372197000 |
| N | -3.264910000 | -2.733406000 | 0.552402000 |
| N | -4.652045000 | -2.665180000 | 0.512970000 |
| C | -5.098667000 | -3.906143000 | 0.736033000 |
| C | -4.025523000 | -4.795557000 | 0.901911000 |
| C | -2.885564000 | -4.026765000 | 0.768229000 |
| H | -6.162234000 | -4.094599000 | 0.752259000 |
| H | -4.072308000 | -5.858306000 | 1.085460000 |
| H | -1.850442000 | -4.319039000 | 0.778000000 |
| Os | -5.418819000 | -0.816810000 | -0.099183000 |
| C | -5.566172000 | 0.595527000 | 4.077872000 |
| C | -5.120837000 | 0.227278000 | 2.815520000 |
| N | -5.945416000 | -0.235781000 | 1.848608000 |
| C | -7.292278000 | -0.344796000 | 2.135843000 |
| C | -7.788373000 | 0.013987000 | 3.391692000 |
| C | -6.926033000 | 0.488066000 | 4.374626000 |
| C | -8.141172000 | -0.855735000 | 1.046817000 |
| N | -7.439999000 | -1.135655000 | -0.084016000 |
| C | -8.038040000 | -1.620322000 | -1.204710000 |
| C | -9.416314000 | -1.844616000 | -1.214597000 |
| C | -10.155232000 | -1.563340000 | -0.063226000 |
| C | -9.521363000 | -1.065812000 | 1.077645000 |
| C | -7.089516000 | -1.847630000 | -2.307361000 |
| N | -5.770389000 | -1.536404000 | -2.040292000 |
| C | -4.858187000 | -1.724077000 | -3.021163000 |
| C | -5.187617000 | -2.213397000 | -4.277917000 |
| C | -6.518788000 | -2.530547000 | -4.554074000 |
| C | -7.470135000 | -2.343522000 | -3.556861000 |
| H | -4.074996000 | 0.295961000 | 2.542402000 |
| H | -4.853211000 | 0.960008000 | 4.809240000 |
| H | -7.308104000 | 0.767472000 | 5.350804000 |
| H | -8.848584000 | -0.078850000 | 3.596107000 |
| H | -10.098123000 | -0.849691000 | 1.969325000 |
| H | -11.226602000 | -1.732203000 | -0.054949000 |
| H | -9.911915000 | -2.230009000 | -2.097976000 |
| H | -8.510430000 | -2.581817000 | -3.744948000 |
| H | -6.810987000 | -2.915338000 | -5.525425000 |
| H | -4.408334000 | -2.341180000 | -5.021182000 |
| H | -3.838082000 | -1.466143000 | -2.764434000 |
| N | -3.968046000 | 1.667848000 | -0.779382000 |
| N | -5.266719000 | 1.177717000 | -0.716726000 |
| C | -6.075780000 | 2.222327000 | -0.925343000 |
| C | -5.329732000 | 3.397880000 | -1.104006000 |

| | | | |
|---|---|---|---|
| C | -4.006892000 | 3.015358000 | -0.993790000 |
| C | 3.410032000 | 2.934071000 | -0.649145000 |
| N | 4.797542000 | 2.821001000 | -0.645787000 |
| C | 5.537927000 | 3.940318000 | -0.761515000 |
| C | 5.011638000 | 5.219787000 | -0.853848000 |
| C | 3.617511000 | 5.359267000 | -0.790525000 |
| C | 2.832434000 | 4.204661000 | -0.683209000 |
| O | 2.952908000 | 6.531122000 | -0.814664000 |
| C | 3.697506000 | 7.760552000 | -0.899320000 |
| C | 2.719681000 | 8.944415000 | -0.878184000 |
| C | 3.564920000 | 10.229904000 | -0.971116000 |
| C | 1.917064000 | 8.944354000 | 0.436699000 |
| C | 1.763716000 | 8.863093000 | -2.083305000 |
| H | 4.281962000 | 7.761771000 | -1.828597000 |
| H | 4.390050000 | 7.818853000 | -0.049657000 |
| H | 4.153335000 | 10.256906000 | -1.896413000 |
| H | 4.257406000 | 10.317137000 | -0.124934000 |
| H | 2.914170000 | 11.111597000 | -0.962769000 |
| H | 2.583142000 | 9.009454000 | 1.306047000 |
| H | 1.317766000 | 8.033645000 | 0.535778000 |
| H | 1.237827000 | 9.804031000 | 0.468886000 |
| H | 2.318908000 | 8.873655000 | -3.029458000 |
| H | 1.079328000 | 9.719337000 | -2.085734000 |
| H | 1.163168000 | 7.948351000 | -2.052089000 |
| H | 5.682006000 | 6.064008000 | -0.946366000 |
| H | 6.612159000 | 3.795021000 | -0.769083000 |
| H | 1.762985000 | 4.329466000 | -0.591209000 |
| H | -3.111233000 | 3.610698000 | -1.018372000 |
| H | -5.703141000 | 4.395267000 | -1.280400000 |
| H | -7.146183000 | 2.076200000 | -0.923652000 |

Cartesian coordinates of DFT-optimized structure of **10a** complex by B3LYP/Def2-TZVP; 6-31G(d,p)/$CH_3CN$, charge = 2, multiplicity = 1

| | | | |
|---|---|---|---|
| Os | -2.325683000 | -1.311965000 | 0.059560000 |
| N | -3.061700000 | 0.652485000 | 0.276067000 |
| N | -2.570052000 | -1.778548000 | 2.090816000 |
| N | -4.033096000 | -2.438454000 | 0.044052000 |
| N | -2.831183000 | -1.329170000 | -1.976380000 |
| C | -1.778314000 | -1.394390000 | 3.118739000 |
| H | -0.931349000 | -0.776320000 | 2.847462000 |
| C | -2.019740000 | -1.757093000 | 4.436473000 |
| H | -1.345371000 | -1.417199000 | 5.214812000 |
| C | -3.128538000 | -2.554302000 | 4.728302000 |
| H | -3.346071000 | -2.856526000 | 5.747280000 |
| C | -3.955191000 | -2.955204000 | 3.684562000 |
| H | -4.823805000 | -3.571969000 | 3.883614000 |
| C | -3.671414000 | -2.564299000 | 2.373284000 |
| C | -4.500865000 | -2.943294000 | 1.218586000 |
| C | -5.655856000 | -3.728105000 | 1.226542000 |

| | | | |
|---|---|---|---|
| H | -6.043190000 | -4.138951000 | 2.151696000 |
| C | -6.311924000 | -3.979818000 | 0.019294000 |
| C | -5.816051000 | -3.452807000 | -1.175514000 |
| H | -6.326630000 | -3.651665000 | -2.110622000 |
| C | -4.658151000 | -2.673269000 | -1.142295000 |
| C | -3.977286000 | -2.038310000 | -2.281507000 |
| C | -4.433964000 | -2.129126000 | -3.599220000 |
| H | -5.334895000 | -2.690903000 | -3.816588000 |
| C | -3.736502000 | -1.501405000 | -4.625517000 |
| H | -4.088235000 | -1.569880000 | -5.649495000 |
| C | -2.579835000 | -0.785206000 | -4.309855000 |
| H | -2.001109000 | -0.277851000 | -5.073887000 |
| C | -2.165089000 | -0.723966000 | -2.986863000 |
| H | -1.274021000 | -0.180767000 | -2.697098000 |
| C | -4.369742000 | 1.000294000 | 0.269247000 |
| H | -5.075354000 | 0.177778000 | 0.282088000 |
| C | -4.818450000 | 2.303636000 | 0.236422000 |
| H | -5.878475000 | 2.528795000 | 0.231381000 |
| C | -3.867613000 | 3.336758000 | 0.174831000 |
| C | -2.511791000 | 2.998837000 | 0.194818000 |
| H | -1.762608000 | 3.767867000 | 0.097912000 |
| C | -2.106255000 | 1.657830000 | 0.285260000 |
| C | 5.436941000 | 2.134756000 | 0.150680000 |
| C | -0.716004000 | 1.174528000 | 0.291363000 |
| C | 5.373186000 | 3.531634000 | 0.349579000 |
| C | -0.652217000 | -0.222357000 | 0.092508000 |
| C | 4.131114000 | 4.161857000 | 0.510936000 |
| C | 0.589832000 | -0.852516000 | -0.069204000 |
| C | 2.927987000 | 3.433411000 | 0.601419000 |
| C | 1.792931000 | -0.124025000 | -0.159860000 |
| C | 2.973040000 | 2.030939000 | 0.252443000 |
| C | 1.747870000 | 1.278442000 | 0.189149000 |
| C | 4.233973000 | 1.394005000 | -0.032991000 |
| C | 0.486951000 | 1.915360000 | 0.474709000 |
| C | 4.190320000 | 0.066633000 | -0.567673000 |
| C | 0.530611000 | 3.242786000 | 1.009272000 |
| H | 5.085960000 | -0.361209000 | -0.999687000 |
| H | -0.365021000 | 3.670651000 | 1.441280000 |
| C | 3.036387000 | -0.657294000 | -0.622846000 |
| C | 1.684526000 | 3.966741000 | 1.064328000 |
| H | 3.060429000 | -1.631451000 | -1.094768000 |
| H | 1.660505000 | 4.940962000 | 1.536128000 |
| O | -4.348370000 | 4.591983000 | 0.090771000 |
| C | -3.431815000 | 5.700582000 | 0.034343000 |
| C | -4.229641000 | 7.010380000 | -0.042047000 |
| C | -3.205426000 | 8.160846000 | -0.097497000 |
| C | -5.098843000 | 7.026187000 | -1.313773000 |
| C | -5.115580000 | 7.163713000 | 1.208754000 |
| H | -2.799081000 | 5.687019000 | 0.931651000 |
| H | -2.789990000 | 5.590656000 | -0.849397000 |
| H | -3.723318000 | 9.124889000 | -0.153792000 |

| | | | |
|---|---|---|---|
| H | -2.555613000 | 8.077544000 | -0.977255000 |
| H | -2.568998000 | 8.174350000 | 0.795790000 |
| H | -4.481565000 | 6.928145000 | -2.215479000 |
| H | -5.651950000 | 7.969722000 | -1.386258000 |
| H | -5.824379000 | 6.206431000 | -1.308153000 |
| H | -4.509772000 | 7.164786000 | 2.123395000 |
| H | -5.842029000 | 6.348189000 | 1.283508000 |
| H | -5.668870000 | 8.109194000 | 1.171103000 |
| H | -7.210045000 | -4.587825000 | 0.009467000 |
| N | 0.457431000 | -2.271464000 | -0.155067000 |
| C | 1.337948000 | -3.313542000 | -0.117781000 |
| C | 0.614370000 | -4.490859000 | -0.139255000 |
| C | -0.733706000 | -4.101383000 | -0.169920000 |
| N | -0.837438000 | -2.767184000 | -0.167390000 |
| H | -1.628847000 | -4.706235000 | -0.180766000 |
| H | 1.010399000 | -5.495037000 | -0.125400000 |
| H | 2.399460000 | -3.154454000 | -0.045130000 |
| N | 4.263537000 | 5.580781000 | 0.596920000 |
| N | 5.558427000 | 6.076447000 | 0.609928000 |
| C | 5.454730000 | 7.410650000 | 0.612045000 |
| C | 4.106684000 | 7.800169000 | 0.580423000 |
| C | 3.383076000 | 6.622878000 | 0.558813000 |
| C | 6.827158000 | 1.651348000 | 0.157269000 |
| C | 7.232594000 | 0.310294000 | 0.247455000 |
| C | 8.588387000 | -0.027707000 | 0.267882000 |
| C | 9.539308000 | 1.005379000 | 0.207072000 |
| C | 9.090684000 | 2.308759000 | 0.174497000 |
| N | 7.782664000 | 2.656635000 | 0.167188000 |
| O | 9.069019000 | -1.282995000 | 0.351713000 |
| C | 8.152358000 | -2.391562000 | 0.407015000 |
| C | 8.950043000 | -3.701475000 | 0.482863000 |
| C | 7.925704000 | -4.851889000 | 0.537185000 |
| C | 9.836442000 | -3.854074000 | -0.767699000 |
| C | 9.818759000 | -3.718184000 | 1.754910000 |
| H | 6.349879000 | 8.015484000 | 0.623278000 |
| H | 3.710700000 | 8.804358000 | 0.566102000 |
| H | 2.321596000 | 6.463781000 | 0.485629000 |
| H | 6.483341000 | -0.458734000 | 0.343827000 |
| H | 10.599319000 | 0.780161000 | 0.212499000 |
| H | 9.796351000 | 3.131234000 | 0.162253000 |
| H | 7.519994000 | -2.377343000 | -0.490546000 |
| H | 7.510166000 | -2.282178000 | 1.290558000 |
| H | 7.275555000 | -4.769094000 | 1.416746000 |
| H | 8.443494000 | -5.816009000 | 0.593072000 |
| H | 7.289612000 | -4.864775000 | -0.356353000 |
| H | 9.230986000 | -3.854471000 | -1.682574000 |
| H | 10.389621000 | -4.799635000 | -0.730464000 |
| H | 10.562994000 | -3.038569000 | -0.841629000 |
| H | 9.201143000 | -3.620675000 | 2.656440000 |
| H | 10.544361000 | -2.898481000 | 1.750096000 |
| H | 10.371764000 | -4.661808000 | 1.826991000 |

| | | | |
|---|---|---|---|
| Os | 7.046718000 | 4.621100000 | 0.383769000 |
| C | 7.297495000 | 4.092867000 | 4.753169000 |
| C | 6.883799000 | 4.032061000 | 3.429827000 |
| N | 7.550699000 | 4.637608000 | 2.420079000 |
| C | 8.696585000 | 5.346610000 | 2.726336000 |
| C | 9.152188000 | 5.437025000 | 4.044449000 |
| C | 8.453891000 | 4.808986000 | 5.069984000 |
| C | 9.378377000 | 5.981850000 | 1.587848000 |
| N | 8.754232000 | 5.747422000 | 0.400944000 |
| C | 9.222967000 | 6.252551000 | -0.773071000 |
| C | 10.378049000 | 7.037239000 | -0.779915000 |
| C | 11.033202000 | 7.288546000 | 0.427919000 |
| C | 10.536335000 | 6.761251000 | 1.622186000 |
| C | 8.394395000 | 5.873962000 | -1.928536000 |
| N | 7.292743000 | 5.088232000 | -1.647166000 |
| C | 6.501824000 | 4.704376000 | -2.675828000 |
| C | 6.744356000 | 5.067398000 | -3.993277000 |
| C | 7.853438000 | 5.864614000 | -4.283989000 |
| C | 8.679266000 | 6.265191000 | -3.239475000 |
| H | 5.992957000 | 3.488963000 | 3.139179000 |
| H | 6.718168000 | 3.585250000 | 5.516572000 |
| H | 8.804790000 | 4.877167000 | 6.094268000 |
| H | 10.052944000 | 5.998730000 | 4.262727000 |
| H | 11.046196000 | 6.959803000 | 2.557753000 |
| H | 11.931379000 | 7.896454000 | 0.438623000 |
| H | 10.766131000 | 7.448292000 | -1.704663000 |
| H | 9.548083000 | 6.881952000 | -3.437656000 |
| H | 8.071821000 | 6.167099000 | -5.302708000 |
| H | 6.070607000 | 4.727738000 | -4.772255000 |
| H | 5.654599000 | 4.086289000 | -2.405408000 |

Cartesian coordinates of DFT-optimized structure of **11a** complex by B3LYP/Def2-TZVP; 6-31G(d,p)/$CH_3CN$, charge = 2, multiplicity = 1

| | | | |
|---|---|---|---|
| Os | 2.896711000 | 0.434224000 | 0.036746000 |
| N | 1.753851000 | -1.326503000 | 0.235599000 |
| N | 3.356598000 | 0.535523000 | 2.081363000 |
| N | 4.822511000 | -0.257040000 | 0.086082000 |
| N | 3.273668000 | 0.017709000 | -1.984110000 |
| N | -7.804107000 | 2.417875000 | 0.024858000 |
| C | 2.543617000 | 0.949674000 | 3.080764000 |
| H | 1.552420000 | 1.260782000 | 2.774763000 |
| C | 2.935937000 | 0.980905000 | 4.411628000 |
| H | 2.236241000 | 1.324565000 | 5.165569000 |
| C | 4.227171000 | 0.568739000 | 4.747970000 |
| H | 4.566060000 | 0.580478000 | 5.778476000 |
| C | 5.076148000 | 0.140045000 | 3.733625000 |
| H | 6.083015000 | -0.185867000 | 3.967331000 |
| C | 4.635467000 | 0.125893000 | 2.407405000 |
| C | 5.471697000 | -0.317319000 | 1.280958000 |

| | | | |
|---|---|---|---|
| C | 6.793426000 | -0.764819000 | 1.332094000 |
| H | 7.327058000 | -0.817458000 | 2.273939000 |
| C | 7.425605000 | -1.145741000 | 0.146219000 |
| C | 6.742088000 | -1.077729000 | -1.070064000 |
| H | 7.236302000 | -1.372511000 | -1.988453000 |
| C | 5.421472000 | -0.624221000 | -1.079887000 |
| C | 4.537632000 | -0.475464000 | -2.246852000 |
| C | 4.921933000 | -0.803524000 | -3.549813000 |
| H | 5.917715000 | -1.189791000 | -3.733732000 |
| C | 4.030834000 | -0.637708000 | -4.604374000 |
| H | 4.326238000 | -0.891338000 | -5.616961000 |
| C | 2.754210000 | -0.141925000 | -4.331393000 |
| H | 2.022342000 | 0.003403000 | -5.118377000 |
| C | 2.418086000 | 0.169870000 | -3.021385000 |
| H | 1.438976000 | 0.555025000 | -2.764462000 |
| C | 2.257896000 | -2.581898000 | 0.276979000 |
| H | 3.337681000 | -2.652940000 | 0.338211000 |
| C | 1.484255000 | -3.722313000 | 0.234770000 |
| H | 1.937414000 | -4.706022000 | 0.270817000 |
| C | 0.091530000 | -3.580826000 | 0.108437000 |
| C | -0.449786000 | -2.293154000 | 0.076220000 |
| H | -1.508956000 | -2.155468000 | -0.070213000 |
| C | 0.377121000 | -1.162967000 | 0.180595000 |
| C | -4.481265000 | 4.564948000 | -0.306666000 |
| C | -0.066382000 | 0.238874000 | 0.143428000 |
| C | -5.589109000 | 3.716457000 | -0.146222000 |
| C | 1.022387000 | 1.122786000 | -0.006293000 |
| C | -5.396940000 | 2.331016000 | 0.026641000 |
| C | 0.793130000 | 2.495175000 | -0.198741000 |
| C | -6.674176000 | 1.601109000 | 0.022037000 |
| C | -6.837460000 | 0.218542000 | -0.075721000 |
| H | -5.982273000 | -0.433914000 | -0.176070000 |
| C | -8.109521000 | -0.366998000 | -0.092848000 |
| C | -9.233573000 | 0.468175000 | -0.019736000 |
| H | -10.246959000 | 0.089192000 | -0.018001000 |
| C | -9.019761000 | 1.837669000 | 0.020146000 |
| H | -9.864711000 | 2.516582000 | 0.038269000 |
| C | -4.075873000 | 1.814697000 | 0.181130000 |
| C | -0.500476000 | 3.019029000 | -0.374456000 |
| C | -2.956418000 | 2.671871000 | -0.113376000 |
| C | -1.617603000 | 2.146055000 | -0.085000000 |
| C | -3.170936000 | 4.067377000 | -0.428930000 |
| C | -1.391348000 | 0.761276000 | 0.240845000 |
| C | -2.051082000 | 4.831802000 | -0.892519000 |
| C | -2.510696000 | 0.016472000 | 0.724867000 |
| H | -2.213461000 | 5.806026000 | -1.335898000 |
| H | -2.347146000 | -0.946964000 | 1.191048000 |
| C | -0.784484000 | 4.334317000 | -0.867256000 |
| C | -3.782096000 | 0.514304000 | 0.694609000 |
| H | 0.012580000 | 4.931797000 | -1.291360000 |
| H | -4.576650000 | -0.075081000 | 1.134597000 |

| | | | |
|---|---|---|---|
| O | -0.621214000 | -4.720261000 | 0.018873000 |
| O | -8.144462000 | -1.710240000 | -0.189166000 |
| C | -9.418225000 | -2.380841000 | -0.225420000 |
| C | -9.187471000 | -3.892597000 | -0.365223000 |
| C | -10.576267000 | -4.560384000 | -0.395701000 |
| C | -8.383959000 | -4.419772000 | 0.838653000 |
| C | -8.437165000 | -4.194324000 | -1.676409000 |
| C | -2.053244000 | -4.653972000 | -0.109284000 |
| C | -2.621343000 | -6.079894000 | -0.154805000 |
| C | -4.150709000 | -5.953285000 | -0.299110000 |
| C | -2.046800000 | -6.840861000 | -1.364717000 |
| C | -2.282544000 | -6.825124000 | 1.150116000 |
| H | -9.999308000 | -2.000505000 | -1.075478000 |
| H | -9.965194000 | -2.159450000 | 0.700266000 |
| H | -11.173987000 | -4.205389000 | -1.244151000 |
| H | -11.138370000 | -4.358756000 | 0.524356000 |
| H | -10.471145000 | -5.646900000 | -0.492144000 |
| H | -8.908441000 | -4.221495000 | 1.781607000 |
| H | -7.396905000 | -3.949233000 | 0.893442000 |
| H | -8.240029000 | -5.503238000 | 0.755621000 |
| H | -8.999088000 | -3.831324000 | -2.545952000 |
| H | -8.296199000 | -5.274843000 | -1.794929000 |
| H | -7.450683000 | -3.719675000 | -1.687626000 |
| H | -2.464057000 | -4.104045000 | 0.747528000 |
| H | -2.306035000 | -4.110349000 | -1.028911000 |
| H | -4.610415000 | -6.947467000 | -0.332204000 |
| H | -4.423936000 | -5.427094000 | -1.221910000 |
| H | -4.590191000 | -5.409043000 | 0.545874000 |
| H | -2.283664000 | -6.326206000 | -2.304212000 |
| H | -2.472571000 | -7.849569000 | -1.417680000 |
| H | -0.958459000 | -6.935212000 | -1.294111000 |
| H | -2.697142000 | -6.303262000 | 2.021611000 |
| H | -1.199985000 | -6.910485000 | 1.288374000 |
| H | -2.704282000 | -7.836698000 | 1.132197000 |
| H | 8.452144000 | -1.495093000 | 0.169785000 |
| N | 2.012917000 | 3.236636000 | -0.231423000 |
| C | 2.319742000 | 4.565297000 | -0.173253000 |
| C | 3.695586000 | 4.689283000 | -0.138154000 |
| C | 4.190221000 | 3.375705000 | -0.155485000 |
| N | 3.183906000 | 2.494943000 | -0.198238000 |
| N | -4.870074000 | 5.937517000 | -0.366936000 |
| C | -4.193805000 | 7.121402000 | -0.303995000 |
| C | -5.119119000 | 8.147639000 | -0.309756000 |
| C | -6.373874000 | 7.520071000 | -0.356433000 |
| N | -6.233538000 | 6.189554000 | -0.377701000 |
| H | 5.209762000 | 3.019728000 | -0.126535000 |
| H | 4.262630000 | 5.606920000 | -0.095858000 |
| H | 1.554965000 | 5.320510000 | -0.125281000 |
| H | -3.121376000 | 7.156740000 | -0.225314000 |
| H | -4.912420000 | 9.206625000 | -0.274983000 |
| H | -7.363549000 | 7.953167000 | -0.363276000 |

| | | | |
|---|---|---|---|
| Os | -7.432977000 | 4.486056000 | -0.169640000 |
| C | -7.225188000 | 4.911689000 | 4.214602000 |
| C | -6.917655000 | 4.621197000 | 2.892811000 |
| N | -7.761843000 | 4.874359000 | 1.866016000 |
| C | -8.985836000 | 5.447990000 | 2.153379000 |
| C | -9.340301000 | 5.758838000 | 3.469029000 |
| C | -8.459980000 | 5.492488000 | 4.511890000 |
| C | -9.863736000 | 5.695117000 | 0.998861000 |
| C | -11.135262000 | 6.272210000 | 1.010340000 |
| C | -11.820110000 | 6.425555000 | -0.197345000 |
| C | -11.237777000 | 6.006466000 | -1.395880000 |
| C | -9.964524000 | 5.434128000 | -1.365927000 |
| N | -9.312249000 | 5.294210000 | -0.179299000 |
| C | -9.182489000 | 4.936470000 | -2.508790000 |
| N | -7.931328000 | 4.433455000 | -2.206651000 |
| C | -7.171249000 | 3.957658000 | -3.219966000 |
| C | -7.589699000 | 3.955952000 | -4.543286000 |
| C | -8.851836000 | 4.465313000 | -4.855993000 |
| C | -9.647557000 | 4.955911000 | -3.826504000 |
| H | -6.503832000 | 4.684662000 | 4.991926000 |
| H | -5.972256000 | 4.170982000 | 2.616410000 |
| H | -10.305565000 | 6.207893000 | 3.671921000 |
| H | -8.732193000 | 5.732383000 | 5.534284000 |
| H | -11.589674000 | 6.599011000 | 1.938482000 |
| H | -12.808307000 | 6.872532000 | -0.204590000 |
| H | -11.771765000 | 6.128153000 | -2.331144000 |
| H | -6.201153000 | 3.571417000 | -2.932146000 |
| H | -6.932368000 | 3.560214000 | -5.309700000 |
| H | -9.209873000 | 4.479204000 | -5.879983000 |
| H | -10.632421000 | 5.353891000 | -4.041418000 |

Cartesian coordinates of DFT-optimized structure of **7b** complex by B3LYP/Def2-TZVP; 6-31G(d,p)/$CH_3CN$, charge = 2, multiplicity = 1

| | | | |
|---|---|---|---|
| Ru | -5.528947000 | -0.048614000 | 0.031325000 |
| Ru | 5.519871000 | 0.093238000 | 0.002517000 |
| N | -5.076800000 | -2.087723000 | 0.301778000 |
| N | 5.067737000 | 2.132368000 | -0.267859000 |
| N | -5.983851000 | 0.247450000 | 2.054640000 |
| N | 5.974280000 | -0.202880000 | -2.020890000 |
| N | -7.569508000 | -0.076491000 | 0.018706000 |
| N | 7.560441000 | 0.121056000 | 0.014648000 |
| N | -5.950729000 | -0.357291000 | -1.999260000 |
| N | 5.942158000 | 0.401910000 | 2.032999000 |
| N | -5.134994000 | 2.002972000 | -0.234019000 |
| N | 5.125900000 | -1.958317000 | 0.268004000 |
| C | -5.108893000 | 0.408645000 | 3.067901000 |
| C | 5.099073000 | -0.364087000 | -3.033934000 |
| H | 4.052270000 | -0.332824000 | -2.755968000 |
| C | -5.504589000 | 0.602553000 | 4.386522000 |

| | | | |
|---|---|---|---|
| C | 5.494443000 | -0.558064000 | -4.352643000 |
| H | 4.742379000 | -0.682118000 | -5.124090000 |
| C | -6.867421000 | 0.633104000 | 4.683909000 |
| C | 6.857201000 | -0.588676000 | -4.650360000 |
| C | -7.782766000 | 0.467982000 | 3.648811000 |
| C | 7.772800000 | -0.423537000 | -3.615489000 |
| H | -8.846788000 | 0.487513000 | 3.853965000 |
| C | -7.329971000 | 0.276158000 | 2.340709000 |
| C | 7.320331000 | -0.231638000 | -2.307287000 |
| C | -8.233637000 | 0.090918000 | 1.188334000 |
| C | 8.224282000 | -0.046381000 | -1.155140000 |
| C | -9.630826000 | 0.071590000 | 1.205243000 |
| C | 9.621467000 | -0.027036000 | -1.172395000 |
| H | -10.181878000 | 0.203278000 | 2.129202000 |
| H | 10.172295000 | -0.158731000 | -2.096487000 |
| C | -10.315264000 | -0.122786000 | 0.003189000 |
| C | 10.306201000 | 0.167374000 | 0.029486000 |
| C | -9.611303000 | -0.294463000 | -1.190940000 |
| C | 9.602535000 | 0.339068000 | 1.223786000 |
| C | -8.214388000 | -0.267466000 | -1.157888000 |
| C | 8.205612000 | 0.312058000 | 1.191078000 |
| C | -7.292291000 | -0.426611000 | -2.299495000 |
| C | 7.283793000 | 0.471209000 | 2.332908000 |
| C | -7.725502000 | -0.633450000 | -3.611825000 |
| C | 7.717331000 | 0.678021000 | 3.645134000 |
| H | -8.786300000 | -0.685625000 | -3.827611000 |
| C | -6.795090000 | -0.772412000 | -4.637256000 |
| C | 6.787175000 | 0.816973000 | 4.670798000 |
| C | -5.437090000 | -0.699507000 | -4.325849000 |
| C | 5.429097000 | 0.744082000 | 4.359724000 |
| H | 4.665950000 | 0.845780000 | 5.123550000 |
| C | -5.061090000 | -0.491993000 | -3.003403000 |
| C | 5.052768000 | 0.536599000 | 3.037365000 |
| H | 4.010236000 | 0.472279000 | 2.749399000 |
| C | -5.975305000 | -3.093795000 | 0.332035000 |
| C | 5.966249000 | 3.138431000 | -0.298206000 |
| H | -7.018045000 | -2.796527000 | 0.344451000 |
| H | 7.008984000 | 2.841154000 | -0.310759000 |
| C | -5.633001000 | -4.431762000 | 0.336128000 |
| C | 5.623960000 | 4.476403000 | -0.302227000 |
| H | -6.392752000 | -5.204178000 | 0.363342000 |
| H | 6.383717000 | 5.248810000 | -0.329520000 |
| C | -4.271432000 | -4.770695000 | 0.269172000 |
| C | 4.262403000 | 4.815355000 | -0.235106000 |
| C | -3.326093000 | -3.741026000 | 0.240560000 |
| C | 3.317055000 | 3.785696000 | -0.206393000 |
| H | -2.277921000 | -3.971350000 | 0.135886000 |
| H | 2.268896000 | 4.016025000 | -0.101593000 |
| C | -3.732056000 | -2.397699000 | 0.298179000 |
| C | 3.723000000 | 2.442367000 | -0.264075000 |
| C | -2.843792000 | -1.223874000 | 0.255006000 |

| | | | |
|---|---|---|---|
| C | 2.834720000 | 1.268562000 | -0.220790000 |
| C | -3.554356000 | -0.022727000 | 0.037562000 |
| C | 3.545280000 | 0.067407000 | -0.003379000 |
| C | -2.876264000 | 1.195129000 | -0.187957000 |
| C | 2.867182000 | -1.150434000 | 0.222199000 |
| C | -3.792026000 | 2.347869000 | -0.230321000 |
| C | 3.782924000 | -2.303190000 | 0.264505000 |
| C | -3.431696000 | 3.695620000 | -0.171725000 |
| C | 3.422564000 | -3.650940000 | 0.206054000 |
| H | -2.396926000 | 3.991541000 | -0.074636000 |
| H | 2.387776000 | -3.946852000 | 0.109131000 |
| C | -4.402385000 | 4.706359000 | -0.193126000 |
| C | 4.393238000 | -4.661693000 | 0.227403000 |
| C | -5.753111000 | 4.338199000 | -0.259785000 |
| C | 5.743978000 | -4.293552000 | 0.293859000 |
| H | -6.557868000 | 5.060744000 | -0.285278000 |
| H | 6.548726000 | -5.016109000 | 0.319301000 |
| C | -6.053146000 | 2.982445000 | -0.261457000 |
| C | 6.044037000 | -2.937804000 | 0.295390000 |
| H | -7.088722000 | 2.660602000 | -0.275433000 |
| H | 7.079621000 | -2.615979000 | 0.309204000 |
| C | -1.462667000 | 1.193898000 | -0.347210000 |
| C | 1.453590000 | -1.149181000 | 0.381509000 |
| C | -0.724526000 | 0.012696000 | 0.021401000 |
| C | 0.715449000 | 0.032023000 | 0.012906000 |
| C | -1.427857000 | -1.186761000 | 0.399286000 |
| C | 1.418780000 | 1.231474000 | -0.364994000 |
| C | -0.651502000 | -2.247380000 | 0.966267000 |
| C | 0.642426000 | 2.292113000 | -0.931939000 |
| H | -1.154566000 | -3.062329000 | 1.472331000 |
| H | 1.145492000 | 3.107080000 | -1.437971000 |
| C | 0.712958000 | -2.230319000 | 0.956409000 |
| C | -0.722034000 | 2.275055000 | -0.922075000 |
| H | 1.245190000 | -3.033455000 | 1.451271000 |
| H | -1.254265000 | 3.078206000 | -1.416913000 |
| O | -3.980564000 | -6.085824000 | 0.229460000 |
| O | 3.935788000 | -5.928160000 | 0.172389000 |
| O | 3.971556000 | 6.130487000 | -0.195342000 |
| O | -3.944961000 | 5.972828000 | -0.137951000 |
| C | -4.890123000 | 7.059192000 | -0.136005000 |
| C | -4.130494000 | 8.391266000 | -0.058102000 |
| C | -5.187121000 | 9.513341000 | -0.053575000 |
| C | -3.211532000 | 8.548524000 | -1.284403000 |
| C | -3.301709000 | 8.454096000 | 1.238852000 |
| C | 2.597564000 | 6.551314000 | -0.117127000 |
| C | 2.537644000 | 8.085640000 | -0.143386000 |
| C | 1.052112000 | 8.481778000 | -0.035614000 |
| C | 3.318507000 | 8.667815000 | 1.050004000 |
| C | 3.121945000 | 8.612901000 | -1.467683000 |
| C | -2.606564000 | -6.506635000 | 0.151322000 |
| C | -2.546632000 | -8.040961000 | 0.177539000 |

| | | | |
|---|---|---|---|
| C | -3.131013000 | -8.568265000 | 1.501783000 |
| C | -3.327413000 | -8.623108000 | -1.015918000 |
| C | -1.061090000 | -8.437084000 | 0.069852000 |
| C | 4.880937000 | -7.014536000 | 0.170435000 |
| C | 4.121285000 | -8.346612000 | 0.092777000 |
| C | 3.292349000 | -8.409589000 | -1.204073000 |
| C | 3.202467000 | -8.503718000 | 1.319206000 |
| C | 5.177903000 | -9.468696000 | 0.088260000 |
| H | -5.560667000 | 6.947851000 | 0.725914000 |
| H | -5.489408000 | 7.013553000 | -1.054554000 |
| H | -5.858637000 | 9.427104000 | 0.809277000 |
| H | -5.798061000 | 9.492374000 | -0.964181000 |
| H | -4.698240000 | 10.492609000 | -0.000929000 |
| H | -3.788520000 | 8.515785000 | -2.217022000 |
| H | -2.459490000 | 7.753569000 | -1.322737000 |
| H | -2.687272000 | 9.510472000 | -1.247467000 |
| H | -3.944215000 | 8.358347000 | 2.123001000 |
| H | -2.775182000 | 9.412952000 | 1.308759000 |
| H | -2.555454000 | 7.653720000 | 1.273526000 |
| H | 2.045085000 | 6.132583000 | -0.968282000 |
| H | 2.157359000 | 6.164355000 | 0.811222000 |
| H | 0.949211000 | 9.572618000 | -0.056065000 |
| H | 0.607524000 | 8.121137000 | 0.900074000 |
| H | 0.466473000 | 8.075263000 | -0.869267000 |
| H | 2.909004000 | 8.309660000 | 2.002664000 |
| H | 3.257158000 | 9.762259000 | 1.047998000 |
| H | 4.375620000 | 8.386650000 | 1.007546000 |
| H | 2.566584000 | 8.219935000 | -2.328472000 |
| H | 4.171889000 | 8.324021000 | -1.580442000 |
| H | 3.064793000 | 9.706939000 | -1.501948000 |
| H | -2.054144000 | -6.087924000 | 1.002525000 |
| H | -2.166302000 | -6.119643000 | -0.776987000 |
| H | -2.575708000 | -8.175324000 | 2.362619000 |
| H | -3.073859000 | -9.662304000 | 1.536017000 |
| H | -4.180966000 | -8.279393000 | 1.614486000 |
| H | -4.384532000 | -8.341955000 | -0.973516000 |
| H | -3.266052000 | -9.717551000 | -1.013942000 |
| H | -2.917855000 | -8.264919000 | -1.968541000 |
| H | -0.475505000 | -8.030585000 | 0.903551000 |
| H | -0.616444000 | -8.076418000 | -0.865799000 |
| H | -0.958180000 | -9.527924000 | 0.090283000 |
| H | 5.480343000 | -6.968804000 | 1.088900000 |
| H | 5.551369000 | -6.903294000 | -0.691584000 |
| H | 2.765804000 | -9.368446000 | -1.273803000 |
| H | 3.934755000 | -8.313954000 | -2.088307000 |
| H | 2.546100000 | -7.609208000 | -1.238759000 |
| H | 2.450434000 | -7.708754000 | 1.357534000 |
| H | 3.779565000 | -8.470871000 | 2.251752000 |
| H | 2.678197000 | -9.465668000 | 1.282447000 |
| H | 5.849315000 | -9.382568000 | -0.774684000 |
| H | 4.689008000 | -10.447967000 | 0.035790000 |

| | | | |
|---|---|---|---|
| H | 5.788952000 | -9.447624000 | 0.998789000 |
| H | -4.752717000 | 0.726597000 | 5.158154000 |
| H | -7.212835000 | 0.782198000 | 5.701619000 |
| H | -4.062024000 | 0.377429000 | 2.790183000 |
| H | -10.146584000 | -0.444723000 | -2.121348000 |
| H | -11.399848000 | -0.140662000 | -0.002855000 |
| H | -7.125350000 | -0.933645000 | -5.658147000 |
| H | -4.673753000 | -0.801216000 | -5.089482000 |
| H | -4.018631000 | -0.427660000 | -2.715172000 |
| H | 7.202365000 | -0.737832000 | -5.668145000 |
| H | 8.836772000 | -0.443117000 | -3.820898000 |
| H | 11.390786000 | 0.185269000 | 0.035259000 |
| H | 10.138043000 | 0.489352000 | 2.154060000 |
| H | 8.778183000 | 0.730177000 | 3.860657000 |
| H | 7.117688000 | 0.978185000 | 5.691611000 |

Cartesian coordinates of DFT-optimized structure of **8b** complex by B3LYP/Def2-TZVP; 6-31G(d,p)/$CH_3CN$, charge = 2, multiplicity = 1

| | | | |
|---|---|---|---|
| Ru | 4.983738000 | -0.359031000 | -0.208774000 |
| N | 5.472466000 | 0.158341000 | 1.766846000 |
| N | 7.020202000 | -0.457726000 | -0.237979000 |
| N | 5.363386000 | -0.918401000 | -2.196774000 |
| C | 4.620147000 | 0.466566000 | 2.763512000 |
| H | 3.567970000 | 0.437026000 | 2.506207000 |
| C | 5.043437000 | 0.802280000 | 4.045244000 |
| H | 4.308709000 | 1.041683000 | 4.806079000 |
| C | 6.410614000 | 0.822101000 | 4.318944000 |
| H | 6.777866000 | 1.079491000 | 5.306923000 |
| C | 7.303179000 | 0.503985000 | 3.299130000 |
| H | 8.370357000 | 0.511346000 | 3.487834000 |
| C | 6.822439000 | 0.174556000 | 2.029548000 |
| C | 7.702596000 | -0.175534000 | 0.895806000 |
| C | 9.098815000 | -0.236320000 | 0.900933000 |
| H | 9.666163000 | -0.015056000 | 1.797302000 |
| C | 9.759475000 | -0.590750000 | -0.277218000 |
| C | 9.034203000 | -0.877903000 | -1.435505000 |
| H | 9.551591000 | -1.151881000 | -2.347405000 |
| C | 7.639369000 | -0.803164000 | -1.390397000 |
| C | 6.697391000 | -1.064851000 | -2.498005000 |
| C | 7.107447000 | -1.437512000 | -3.780368000 |
| H | 8.163065000 | -1.547863000 | -3.999524000 |
| C | 6.159474000 | -1.666313000 | -4.773785000 |
| H | 6.471656000 | -1.956678000 | -5.771510000 |
| C | 4.809032000 | -1.514595000 | -4.461097000 |
| H | 4.033103000 | -1.680695000 | -5.200255000 |
| C | 4.457076000 | -1.141263000 | -3.168255000 |
| H | 3.420496000 | -1.010328000 | -2.881004000 |
| C | -3.323694000 | 1.204830000 | -0.408935000 |
| C | 2.249032000 | -1.388353000 | 0.153318000 |

| | | | |
|---|---|---|---|
| C | -4.078685000 | 0.079949000 | -0.075265000 |
| C | 3.003951000 | -0.263327000 | -0.180097000 |
| C | -3.426548000 | -1.113283000 | 0.237273000 |
| C | 2.351719000 | 0.929773000 | -0.492844000 |
| C | -2.020281000 | -1.192969000 | 0.343298000 |
| C | 0.945467000 | 1.009250000 | -0.599307000 |
| C | -1.255332000 | -0.057095000 | -0.117445000 |
| C | 0.180565000 | -0.126691000 | -0.138669000 |
| C | -1.920089000 | 1.148142000 | -0.556950000 |
| C | 0.845382000 | -1.331870000 | 0.300897000 |
| C | -1.124975000 | 2.171558000 | -1.163105000 |
| C | 0.050216000 | -2.355379000 | 0.906852000 |
| H | -1.607708000 | 2.991072000 | -1.679672000 |
| H | 0.532897000 | -3.175031000 | 1.423251000 |
| C | 0.235518000 | 2.105633000 | -1.183188000 |
| C | -1.310272000 | -2.289446000 | 0.926963000 |
| H | 0.779831000 | 2.875394000 | -1.714865000 |
| H | -1.854506000 | -3.059347000 | 1.458506000 |
| H | 10.842855000 | -0.643354000 | -0.292726000 |
| N | -4.357252000 | -2.175108000 | 0.456919000 |
| N | -5.696063000 | -1.823468000 | 0.452385000 |
| C | -6.383992000 | -2.959232000 | 0.591627000 |
| C | -5.515040000 | -4.060994000 | 0.667710000 |
| C | -4.243386000 | -3.530411000 | 0.565746000 |
| H | -7.463896000 | -2.929926000 | 0.617505000 |
| H | -5.776518000 | -5.103042000 | 0.773564000 |
| H | -3.287905000 | -4.023973000 | 0.530568000 |
| Ru | -6.058426000 | 0.176120000 | -0.045124000 |
| C | -5.879763000 | 1.332519000 | 4.206890000 |
| C | -5.529000000 | 0.959024000 | 2.913766000 |
| N | -6.436224000 | 0.735738000 | 1.943251000 |
| C | -7.769952000 | 0.881927000 | 2.245758000 |
| C | -8.178838000 | 1.254745000 | 3.528448000 |
| C | -7.229935000 | 1.483959000 | 4.520883000 |
| C | -8.712973000 | 0.619791000 | 1.139141000 |
| N | -8.094866000 | 0.274590000 | -0.013910000 |
| C | -8.778332000 | -0.008108000 | -1.146928000 |
| C | -10.174592000 | 0.051940000 | -1.150551000 |
| C | -10.834161000 | 0.406203000 | 0.028258000 |
| C | -10.107789000 | 0.693850000 | 1.185736000 |
| C | -7.899212000 | -0.357950000 | -2.281549000 |
| N | -6.548968000 | -0.341324000 | -2.020177000 |
| C | -5.697553000 | -0.649232000 | -3.017727000 |
| C | -6.122017000 | -0.985038000 | -4.299042000 |
| C | -7.489460000 | -1.005302000 | -4.571378000 |
| C | -8.381104000 | -0.687502000 | -3.550661000 |
| H | -4.492672000 | 0.828320000 | 2.625500000 |
| H | -5.103149000 | 1.498938000 | 4.945256000 |
| H | -7.541200000 | 1.774441000 | 5.518861000 |
| H | -9.234264000 | 1.364936000 | 3.748603000 |
| H | -10.624334000 | 0.967649000 | 2.098170000 |

| | | | |
|---|---|---|---|
| H | -11.917551000 | 0.458251000 | 0.044932000 |
| H | -10.742794000 | -0.169816000 | -2.046256000 |
| H | -9.448466000 | -0.695164000 | -3.738320000 |
| H | -7.857615000 | -1.262765000 | -5.559000000 |
| H | -5.387966000 | -1.224171000 | -5.060615000 |
| H | -4.645129000 | -0.619349000 | -2.761474000 |
| N | -4.153337000 | 2.352167000 | -0.602028000 |
| N | -5.519174000 | 2.131912000 | -0.556364000 |
| C | -6.097712000 | 3.329175000 | -0.676609000 |
| C | -5.128805000 | 4.341642000 | -0.780771000 |
| C | -3.912040000 | 3.690194000 | -0.716505000 |
| H | -2.912618000 | 4.088823000 | -0.711100000 |
| H | -5.291154000 | 5.404261000 | -0.880384000 |
| H | -7.175662000 | 3.404680000 | -0.669742000 |
| N | 3.078826000 | -2.535501000 | 0.346819000 |
| C | 2.837736000 | -3.873559000 | 0.461327000 |
| C | 4.054601000 | -4.524745000 | 0.526434000 |
| C | 5.023364000 | -3.512085000 | 0.422749000 |
| N | 4.444639000 | -2.314962000 | 0.302020000 |
| N | 3.282291000 | 1.991810000 | -0.712004000 |
| N | 4.621189000 | 1.640462000 | -0.706547000 |
| C | 5.308970000 | 2.776401000 | -0.845174000 |
| C | 4.439827000 | 3.877974000 | -0.921719000 |
| C | 3.168210000 | 3.347094000 | -0.820730000 |
| H | 1.838399000 | -4.272393000 | 0.455289000 |
| H | 4.217120000 | -5.587319000 | 0.626245000 |
| H | 6.101334000 | -3.587367000 | 0.416512000 |
| H | 2.212583000 | 3.840424000 | -0.786216000 |
| H | 4.701128000 | 4.920096000 | -1.027278000 |
| H | 6.388897000 | 2.747327000 | -0.870373000 |

Cartesian coordinates of DFT-optimized structure of **9b** complex by B3LYP/Def2-TZVP; 6-31G(d,p)/$CH_3CN$, charge = 2, multiplicity = 1

| | | | |
|---|---|---|---|
| Ru | 4.987462000 | -0.190361000 | -0.000082000 |
| N | 4.491808000 | -2.214312000 | 0.306807000 |
| N | 5.460984000 | 0.129319000 | 2.016428000 |
| N | 7.027722000 | -0.271676000 | -0.020971000 |
| N | 5.392324000 | -0.540980000 | -2.026566000 |
| C | 4.596303000 | 0.334121000 | 3.030588000 |
| H | 3.547501000 | 0.327640000 | 2.758634000 |
| C | 5.004064000 | 0.541589000 | 4.343531000 |
| H | 4.259877000 | 0.701233000 | 5.116076000 |
| C | 6.368640000 | 0.539543000 | 4.633948000 |
| H | 6.723450000 | 0.698075000 | 5.646988000 |
| C | 7.273543000 | 0.330105000 | 3.597716000 |
| H | 8.338797000 | 0.325117000 | 3.797402000 |
| C | 6.808812000 | 0.126288000 | 2.295649000 |
| C | 7.701642000 | -0.103808000 | 1.142752000 |
| C | 9.097911000 | -0.161170000 | 1.154142000 |

| | | | |
|---|---|---|---|
| H | 9.656510000 | -0.029965000 | 2.073576000 |
| C | 9.771045000 | -0.392868000 | -0.047661000 |
| C | 9.056959000 | -0.563035000 | -1.235985000 |
| H | 9.583485000 | -0.742788000 | -2.166119000 |
| C | 7.661511000 | -0.496864000 | -1.197380000 |
| C | 6.730081000 | -0.647697000 | -2.332472000 |
| C | 7.151034000 | -0.882133000 | -3.644248000 |
| H | 8.209085000 | -0.962527000 | -3.864943000 |
| C | 6.212007000 | -1.011600000 | -4.663041000 |
| H | 6.532740000 | -1.193562000 | -5.683479000 |
| C | 4.857808000 | -0.902753000 | -4.345527000 |
| H | 4.088092000 | -0.996793000 | -5.103714000 |
| C | 4.494168000 | -0.668846000 | -3.024098000 |
| H | 3.455120000 | -0.576926000 | -2.731081000 |
| C | 5.368494000 | -3.238946000 | 0.350406000 |
| H | 6.417238000 | -2.963484000 | 0.360869000 |
| C | 4.997169000 | -4.568825000 | 0.370207000 |
| H | 5.739673000 | -5.357471000 | 0.405580000 |
| C | 3.628343000 | -4.878600000 | 0.307336000 |
| C | 2.704810000 | -3.829431000 | 0.272371000 |
| H | 1.651001000 | -4.037480000 | 0.175874000 |
| C | 3.140846000 | -2.494790000 | 0.311690000 |
| C | -3.333162000 | 1.258252000 | -0.310181000 |
| C | 2.279573000 | -1.302007000 | 0.255217000 |
| C | -4.076841000 | 0.121292000 | 0.008369000 |
| C | 3.014690000 | -0.120485000 | 0.013116000 |
| C | -3.412251000 | -1.061959000 | 0.332249000 |
| C | 2.362356000 | 1.108410000 | -0.227297000 |
| C | -2.005502000 | -1.122584000 | 0.455211000 |
| C | 0.949116000 | 1.135033000 | -0.383105000 |
| C | -1.250874000 | 0.026682000 | 0.015308000 |
| C | 0.187086000 | -0.022872000 | 0.015675000 |
| C | -1.924897000 | 1.224700000 | -0.425767000 |
| C | 0.867630000 | -1.229927000 | 0.415045000 |
| C | -1.129524000 | 2.264074000 | -1.002449000 |
| C | 0.078530000 | -2.261404000 | 1.016992000 |
| H | -1.610505000 | 3.079372000 | -1.527478000 |
| H | 0.573290000 | -3.078515000 | 1.527278000 |
| C | 0.233098000 | 2.221929000 | -0.978826000 |
| C | -1.284005000 | -2.210809000 | 1.038436000 |
| H | 0.783804000 | 3.007611000 | -1.480801000 |
| H | -1.819964000 | -2.987740000 | 1.568463000 |
| O | 3.309921000 | -6.187556000 | 0.277437000 |
| C | 1.927693000 | -6.579843000 | 0.199623000 |
| C | 1.838339000 | -8.113015000 | 0.200887000 |
| C | 0.344444000 | -8.479605000 | 0.106203000 |
| C | 2.592307000 | -8.688708000 | -1.012881000 |
| C | 2.429437000 | -8.674923000 | 1.507795000 |
| H | 1.387578000 | -6.163080000 | 1.059891000 |
| H | 1.490317000 | -6.170348000 | -0.720322000 |
| H | 0.222257000 | -9.568560000 | 0.107763000 |

| | | | |
|---|---|---|---|
| H | -0.105353000 | -8.093459000 | -0.816631000 |
| H | -0.221798000 | -8.077713000 | 0.955273000 |
| H | 2.182011000 | -8.300660000 | -1.953491000 |
| H | 2.503837000 | -9.781127000 | -1.033184000 |
| H | 3.656520000 | -8.434879000 | -0.976291000 |
| H | 1.893384000 | -8.286033000 | 2.382552000 |
| H | 3.486409000 | -8.409411000 | 1.611207000 |
| H | 2.349884000 | -9.768044000 | 1.523715000 |
| H | 10.854688000 | -0.440919000 | -0.058163000 |
| N | -4.330414000 | -2.137123000 | 0.537321000 |
| N | -5.674547000 | -1.805990000 | 0.506267000 |
| C | -6.347597000 | -2.951161000 | 0.638565000 |
| C | -5.463712000 | -4.039313000 | 0.737134000 |
| C | -4.198606000 | -3.490270000 | 0.656775000 |
| H | -7.428067000 | -2.937983000 | 0.643431000 |
| H | -5.711570000 | -5.084627000 | 0.843481000 |
| H | -3.235808000 | -3.970227000 | 0.643496000 |
| Ru | -6.058629000 | 0.184705000 | -0.004320000 |
| C | -5.992571000 | 1.362736000 | 4.244268000 |
| C | -5.607254000 | 0.988250000 | 2.961282000 |
| N | -6.489015000 | 0.744443000 | 1.972311000 |
| C | -7.831319000 | 0.871664000 | 2.244330000 |
| C | -8.274410000 | 1.245515000 | 3.515227000 |
| C | -7.351638000 | 1.494351000 | 4.527368000 |
| C | -8.745504000 | 0.588796000 | 1.118967000 |
| N | -8.096687000 | 0.248633000 | -0.018628000 |
| C | -8.750216000 | -0.051007000 | -1.164773000 |
| C | -10.146866000 | -0.014682000 | -1.199361000 |
| C | -10.837916000 | 0.334006000 | -0.036969000 |
| C | -10.142037000 | 0.639571000 | 1.134689000 |
| C | -7.840297000 | -0.389989000 | -2.277852000 |
| N | -6.496595000 | -0.347307000 | -1.987282000 |
| C | -5.618509000 | -0.643619000 | -2.965183000 |
| C | -6.009037000 | -0.991386000 | -4.254084000 |
| C | -7.369689000 | -1.037900000 | -4.555713000 |
| C | -8.288708000 | -0.733513000 | -3.555430000 |
| H | -4.562763000 | 0.875127000 | 2.695942000 |
| H | -5.235235000 | 1.545876000 | 4.998534000 |
| H | -7.689669000 | 1.785088000 | 5.516559000 |
| H | -9.336051000 | 1.341172000 | 3.710746000 |
| H | -10.683137000 | 0.908715000 | 2.034200000 |
| H | -11.922142000 | 0.367605000 | -0.044133000 |
| H | -10.691610000 | -0.250451000 | -2.106048000 |
| H | -9.351515000 | -0.762322000 | -3.765155000 |
| H | -7.711704000 | -1.305360000 | -5.550088000 |
| H | -5.254422000 | -1.219604000 | -4.998720000 |
| H | -4.572597000 | -0.595263000 | -2.686179000 |
| N | -4.177585000 | 2.391167000 | -0.522341000 |
| N | -5.540761000 | 2.147712000 | -0.507376000 |
| C | -6.136395000 | 3.334504000 | -0.644433000 |
| C | -5.182622000 | 4.363200000 | -0.729940000 |

| | | | |
|---|---|---|---|
| C | -3.956802000 | 3.733118000 | -0.636562000 |
| C | 3.302552000 | 2.240248000 | -0.289365000 |
| N | 4.637910000 | 1.866737000 | -0.295253000 |
| C | 5.576981000 | 2.825585000 | -0.339291000 |
| C | 5.306174000 | 4.187690000 | -0.351908000 |
| C | 3.963961000 | 4.584801000 | -0.280542000 |
| C | 2.971785000 | 3.595739000 | -0.244099000 |
| O | 3.533074000 | 5.860722000 | -0.233962000 |
| C | 4.497557000 | 6.929554000 | -0.246837000 |
| C | 3.760925000 | 8.274134000 | -0.159423000 |
| C | 4.834876000 | 9.379512000 | -0.176186000 |
| C | 2.955673000 | 8.352097000 | 1.151526000 |
| C | 2.822556000 | 8.443215000 | -1.369269000 |
| H | 5.083106000 | 6.872153000 | -1.173658000 |
| H | 5.178265000 | 6.807927000 | 0.605723000 |
| H | 5.430044000 | 9.346425000 | -1.096964000 |
| H | 5.520073000 | 9.286501000 | 0.675300000 |
| H | 4.362317000 | 10.366584000 | -0.118729000 |
| H | 3.610675000 | 8.240969000 | 2.024610000 |
| H | 2.193903000 | 7.567198000 | 1.196688000 |
| H | 2.450590000 | 9.321592000 | 1.231726000 |
| H | 3.382131000 | 8.401955000 | -2.312005000 |
| H | 2.312093000 | 9.412180000 | -1.323655000 |
| H | 2.059923000 | 7.658207000 | -1.392418000 |
| H | 6.125433000 | 4.893378000 | -0.391353000 |
| H | 6.604776000 | 2.479496000 | -0.354507000 |
| H | 1.944237000 | 3.915640000 | -0.144818000 |
| H | -2.964972000 | 4.149442000 | -0.610584000 |
| H | -5.360939000 | 5.422619000 | -0.836128000 |
| H | -7.215346000 | 3.391944000 | -0.662754000 |

Cartesian coordinates of DFT-optimized structure of **10b** complex by B3LYP/Def2-TZVP; 6-31G(d,p)/$CH_3CN$, charge = 2, multiplicity = 1

| | | | |
|---|---|---|---|
| Ru | 5.459480000 | 0.853480000 | -0.017192000 |
| N | 5.361377000 | -1.225610000 | 0.301772000 |
| N | 5.794623000 | 1.281547000 | 2.009187000 |
| N | 7.467708000 | 1.211454000 | 0.014018000 |
| N | 5.985538000 | 0.567521000 | -2.027467000 |
| C | 4.882827000 | 1.290275000 | 3.001659000 |
| H | 3.869182000 | 1.045284000 | 2.707465000 |
| C | 5.202645000 | 1.590174000 | 4.321421000 |
| H | 4.423723000 | 1.581890000 | 5.075940000 |
| C | 6.525334000 | 1.896040000 | 4.641268000 |
| H | 6.811811000 | 2.135375000 | 5.660070000 |
| C | 7.478765000 | 1.887877000 | 3.627349000 |
| H | 8.513584000 | 2.119611000 | 3.850866000 |
| C | 7.102199000 | 1.578908000 | 2.317977000 |
| C | 8.053187000 | 1.544753000 | 1.188675000 |
| C | 9.424317000 | 1.812033000 | 1.231992000 |

| | | | |
|---|---|---|---|
| H | 9.913756000 | 2.080425000 | 2.160986000 |
| C | 10.162733000 | 1.727853000 | 0.049471000 |
| C | 9.537768000 | 1.383609000 | -1.151233000 |
| H | 10.114889000 | 1.321271000 | -2.066436000 |
| C | 8.164341000 | 1.125884000 | -1.144032000 |
| C | 7.321374000 | 0.754930000 | -2.298372000 |
| C | 7.820357000 | 0.599091000 | -3.594108000 |
| H | 8.875527000 | 0.752269000 | -3.788198000 |
| C | 6.961651000 | 0.247370000 | -4.631614000 |
| H | 7.342981000 | 0.124769000 | -5.639982000 |
| C | 5.609010000 | 0.057377000 | -4.348836000 |
| H | 4.900684000 | -0.217500000 | -5.122650000 |
| C | 5.165965000 | 0.226743000 | -3.041614000 |
| H | 4.124256000 | 0.091117000 | -2.775940000 |
| C | 6.423139000 | -2.055664000 | 0.365493000 |
| H | 7.395406000 | -1.576600000 | 0.397928000 |
| C | 6.323694000 | -3.433064000 | 0.378158000 |
| H | 7.207764000 | -4.057819000 | 0.429135000 |
| C | 5.046039000 | -4.009272000 | 0.287246000 |
| C | 3.932765000 | -3.164138000 | 0.235034000 |
| H | 2.944395000 | -3.578593000 | 0.116939000 |
| C | 4.092412000 | -1.770400000 | 0.280736000 |
| C | -3.008643000 | 0.773636000 | -0.208367000 |
| C | 3.008788000 | -0.772283000 | 0.207332000 |
| C | -3.509820000 | -0.520812000 | 0.032232000 |
| C | 3.509985000 | 0.522135000 | -0.033338000 |
| C | -2.630872000 | -1.582694000 | 0.270377000 |
| C | 2.631050000 | 1.584017000 | -0.271505000 |
| C | -1.239787000 | -1.387993000 | 0.400884000 |
| C | 1.239953000 | 1.389337000 | -0.401960000 |
| C | -0.712142000 | -0.100631000 | 0.011120000 |
| C | 0.712298000 | 0.101993000 | -0.012152000 |
| C | -1.604864000 | 0.970066000 | -0.353781000 |
| C | 1.605015000 | -0.968707000 | 0.352743000 |
| C | -1.019643000 | 2.147266000 | -0.920732000 |
| C | 1.019809000 | -2.145938000 | 0.919636000 |
| H | -1.657259000 | 2.876091000 | -1.404771000 |
| H | 1.657445000 | -2.874800000 | 1.403602000 |
| C | 0.328366000 | 2.351077000 | -0.939648000 |
| C | -0.328198000 | -2.349759000 | 0.938528000 |
| H | 0.711244000 | 3.233606000 | -1.436598000 |
| H | -0.711073000 | -3.232328000 | 1.435409000 |
| O | 4.994180000 | -5.354533000 | 0.248552000 |
| C | 3.718136000 | -6.013899000 | 0.152661000 |
| C | 3.936893000 | -7.533639000 | 0.129106000 |
| C | 2.545454000 | -8.188811000 | 0.027411000 |
| C | 4.787252000 | -7.926371000 | -1.093912000 |
| C | 4.631301000 | -7.988146000 | 1.426829000 |
| H | 3.101776000 | -5.727249000 | 1.014923000 |
| H | 3.212825000 | -5.685402000 | -0.764793000 |
| H | 2.642282000 | -9.280137000 | 0.006100000 |

| | | | |
|---|---|---|---|
| H | 2.022679000 | -7.880847000 | -0.886405000 |
| H | 1.914953000 | -7.925492000 | 0.885473000 |
| H | 4.301846000 | -7.617836000 | -2.028229000 |
| H | 4.924648000 | -9.013248000 | -1.129827000 |
| H | 5.776774000 | -7.459761000 | -1.055096000 |
| H | 4.032264000 | -7.725309000 | 2.307540000 |
| H | 5.616123000 | -7.522344000 | 1.534465000 |
| H | 4.768105000 | -9.075715000 | 1.425933000 |
| H | 11.228064000 | 1.931839000 | 0.063465000 |
| N | 3.317077000 | 2.831183000 | -0.388459000 |
| C | 2.919066000 | 4.136436000 | -0.405069000 |
| C | 4.051082000 | 4.928893000 | -0.428160000 |
| C | 5.132579000 | 4.031924000 | -0.402430000 |
| N | 4.697622000 | 2.769820000 | -0.366378000 |
| N | -3.316871000 | -2.829887000 | 0.387219000 |
| N | -4.697418000 | -2.768560000 | 0.365064000 |
| C | -5.132341000 | -4.030675000 | 0.401045000 |
| C | -4.050824000 | -4.927617000 | 0.426824000 |
| C | -2.918825000 | -4.135130000 | 0.403785000 |
| C | -4.092314000 | 1.771730000 | -0.281708000 |
| C | -3.932754000 | 3.165473000 | -0.235808000 |
| C | -5.046065000 | 4.010556000 | -0.287983000 |
| C | -6.323686000 | 3.434300000 | -0.379043000 |
| C | -6.423062000 | 2.056898000 | -0.366533000 |
| N | -5.361260000 | 1.226888000 | -0.302844000 |
| O | -4.994272000 | 5.355813000 | -0.249105000 |
| C | -3.718259000 | 6.015212000 | -0.153058000 |
| C | -3.937072000 | 7.534941000 | -0.129314000 |
| C | -2.545661000 | 8.190150000 | -0.027461000 |
| C | -4.631429000 | 7.989592000 | -1.427014000 |
| C | -4.787508000 | 7.927480000 | 1.093712000 |
| H | -6.194376000 | -4.230394000 | 0.394465000 |
| H | -4.087133000 | -6.006349000 | 0.450066000 |
| H | -1.879529000 | -4.411370000 | 0.365480000 |
| H | -2.944422000 | 3.579969000 | -0.117566000 |
| H | -7.207784000 | 4.059017000 | -0.429990000 |
| H | -7.395302000 | 1.577784000 | -0.399061000 |
| H | -3.101842000 | 5.728699000 | -1.015325000 |
| H | -3.212984000 | 5.686612000 | 0.764379000 |
| H | -2.022921000 | 7.882080000 | 0.886339000 |
| H | -2.642530000 | 9.281469000 | -0.006006000 |
| H | -1.915108000 | 7.926971000 | -0.885527000 |
| H | -4.032339000 | 7.726892000 | -2.307728000 |
| H | -4.768273000 | 9.077157000 | -1.425983000 |
| H | -5.616230000 | 7.523769000 | -1.534760000 |
| H | -4.302142000 | 7.618835000 | 2.028012000 |
| H | -5.777013000 | 7.460845000 | 1.054780000 |
| H | -4.924940000 | 9.014348000 | 1.129767000 |
| Ru | -5.459303000 | -0.852223000 | 0.015963000 |
| C | -5.609081000 | -0.056378000 | 4.347640000 |
| C | -5.165967000 | -0.225654000 | 3.040433000 |

| | | | |
|---|---|---|---|
| N | -5.985478000 | -0.566418000 | 2.026227000 |
| C | -7.321318000 | -0.753903000 | 2.297059000 |
| C | -7.820367000 | -0.598155000 | 3.592778000 |
| C | -6.961728000 | -0.246449000 | 4.630344000 |
| C | -8.164212000 | -1.124829000 | 1.142655000 |
| N | -7.467523000 | -1.210281000 | -0.015370000 |
| C | -8.052928000 | -1.543535000 | -1.190076000 |
| C | -9.424039000 | -1.810896000 | -1.233471000 |
| C | -10.162514000 | -1.726840000 | -0.050979000 |
| C | -9.537622000 | -1.382639000 | 1.149776000 |
| C | -7.101888000 | -1.577557000 | -2.319339000 |
| N | -5.794339000 | -1.280161000 | -2.010466000 |
| C | -4.882495000 | -1.288766000 | -3.002897000 |
| C | -5.202239000 | -1.588568000 | -4.322696000 |
| C | -6.524901000 | -1.894467000 | -4.642631000 |
| C | -7.478376000 | -1.886432000 | -3.628753000 |
| H | -4.124251000 | -0.089968000 | 2.774811000 |
| H | -4.900806000 | 0.218491000 | 5.121501000 |
| H | -7.343112000 | -0.123920000 | 5.638699000 |
| H | -8.875540000 | -0.751391000 | 3.786805000 |
| H | -10.114788000 | -1.320399000 | 2.064958000 |
| H | -11.227831000 | -1.930893000 | -0.065034000 |
| H | -9.913417000 | -2.079257000 | -2.162506000 |
| H | -8.513176000 | -2.118193000 | -3.852333000 |
| H | -6.811321000 | -2.133729000 | -5.661465000 |
| H | -4.423283000 | -1.580185000 | -5.077178000 |
| H | -3.868875000 | -1.043752000 | -2.708632000 |
| H | 4.087413000 | 6.007624000 | -0.451415000 |
| H | 1.879778000 | 4.412704000 | -0.366744000 |
| H | 6.194620000 | 4.231609000 | -0.395911000 |

Cartesian coordinates of DFT-optimized structure of **11b** complex by B3LYP/Def2-TZVP; 6-31G(d,p)/$CH_3CN$, charge = 2, multiplicity = 1

| | | | |
|---|---|---|---|
| Ru | 5.629169000 | -0.949220000 | 0.031262000 |
| N | 5.169759000 | 1.081668000 | -0.276262000 |
| N | 6.069882000 | -1.291200000 | -1.991022000 |
| N | 7.670384000 | -0.950575000 | 0.034131000 |
| N | 6.063575000 | -0.592019000 | 2.052036000 |
| N | -5.077527000 | 0.799973000 | -0.257374000 |
| C | 5.189952000 | -1.449655000 | -2.999590000 |
| H | 4.144424000 | -1.392628000 | -2.721008000 |
| C | 5.578793000 | -1.673017000 | -4.315942000 |
| H | 4.822873000 | -1.793488000 | -5.084154000 |
| C | 6.939702000 | -1.737447000 | -4.614716000 |
| H | 7.280131000 | -1.910061000 | -5.630372000 |
| C | 7.860369000 | -1.575367000 | -3.583586000 |
| H | 8.923290000 | -1.620347000 | -3.790485000 |
| C | 7.414009000 | -1.352967000 | -2.278393000 |
| C | 8.324971000 | -1.168268000 | -1.130944000 |

| | | | |
|---|---|---|---|
| C | 9.722047000 | -1.200478000 | -1.149362000 |
| H | 10.266258000 | -1.374989000 | -2.070145000 |
| C | 10.414130000 | -1.004170000 | 0.047725000 |
| C | 9.718387000 | -0.781221000 | 1.238083000 |
| H | 10.260073000 | -0.631498000 | 2.164700000 |
| C | 8.321420000 | -0.758627000 | 1.205823000 |
| C | 7.407004000 | -0.545482000 | 2.345752000 |
| C | 7.849511000 | -0.309711000 | 3.649828000 |
| H | 8.911856000 | -0.275186000 | 3.861567000 |
| C | 6.925770000 | -0.117512000 | 4.673096000 |
| H | 7.263316000 | 0.065877000 | 5.687827000 |
| C | 5.565728000 | -0.164146000 | 4.367389000 |
| H | 4.807591000 | -0.019017000 | 5.129126000 |
| C | 5.180655000 | -0.401938000 | 3.052384000 |
| H | 4.135765000 | -0.445093000 | 2.768791000 |
| C | 6.070974000 | 2.083808000 | -0.337357000 |
| H | 7.111865000 | 1.780998000 | -0.364499000 |
| C | 5.733090000 | 3.422674000 | -0.355066000 |
| H | 6.494651000 | 4.192023000 | -0.406164000 |
| C | 4.374079000 | 3.767216000 | -0.270714000 |
| C | 3.424899000 | 2.741627000 | -0.215331000 |
| H | 2.379251000 | 2.977553000 | -0.099511000 |
| C | 3.824710000 | 1.396592000 | -0.258580000 |
| C | -2.695852000 | -2.327771000 | 0.254468000 |
| C | 2.932340000 | 0.224840000 | -0.192250000 |
| C | -3.442790000 | -1.163355000 | 0.032614000 |
| C | 3.651643000 | -0.964420000 | 0.026547000 |
| C | -2.791066000 | 0.063779000 | -0.182936000 |
| C | 2.971452000 | -2.167986000 | 0.253704000 |
| C | -3.744220000 | 1.186888000 | -0.241461000 |
| C | -3.427918000 | 2.544881000 | -0.190643000 |
| H | -2.404681000 | 2.874909000 | -0.083634000 |
| C | -4.429131000 | 3.524967000 | -0.233195000 |
| C | -5.766590000 | 3.115241000 | -0.315804000 |
| H | -6.592313000 | 3.812964000 | -0.360803000 |
| C | -6.024659000 | 1.750484000 | -0.306920000 |
| H | -7.049097000 | 1.394977000 | -0.329702000 |
| C | -1.370665000 | 0.096377000 | -0.314786000 |
| C | 1.571803000 | -2.218600000 | 0.401647000 |
| C | -0.614746000 | -1.075268000 | 0.045385000 |
| C | 0.823303000 | -1.034413000 | 0.044083000 |
| C | -1.295265000 | -2.299854000 | 0.401898000 |
| C | 1.511339000 | 0.177343000 | -0.320337000 |
| C | -0.511056000 | -3.377780000 | 0.926875000 |
| C | 0.721052000 | 1.235592000 | -0.866859000 |
| H | -1.003825000 | -4.220849000 | 1.394614000 |
| H | 1.211633000 | 2.066566000 | -1.357990000 |
| C | 0.849859000 | -3.338944000 | 0.927314000 |
| C | -0.643966000 | 1.198317000 | -0.862255000 |
| H | 1.389648000 | -4.152691000 | 1.395144000 |
| H | -1.183827000 | 2.002382000 | -1.346448000 |

| | | | |
|---|---|---|---|
| O | 4.088870000 | 5.083342000 | -0.242501000 |
| O | -4.009571000 | 4.804002000 | -0.181800000 |
| C | -4.981830000 | 5.865716000 | -0.213578000 |
| C | -4.255976000 | 7.215965000 | -0.123416000 |
| C | -5.337270000 | 8.313558000 | -0.164419000 |
| C | -3.299435000 | 7.384348000 | -1.319062000 |
| C | -3.472168000 | 7.308206000 | 1.199583000 |
| C | 2.717076000 | 5.511823000 | -0.162637000 |
| C | 2.666459000 | 7.046356000 | -0.190476000 |
| C | 1.182540000 | 7.452083000 | -0.094766000 |
| C | 3.441198000 | 7.624169000 | 1.009076000 |
| C | 3.264168000 | 7.570336000 | -1.510019000 |
| H | -5.673268000 | 5.743653000 | 0.630192000 |
| H | -5.553864000 | 5.798512000 | -1.148084000 |
| H | -6.035429000 | 8.221090000 | 0.676523000 |
| H | -5.917310000 | 8.270352000 | -1.094369000 |
| H | -4.872580000 | 9.304263000 | -0.105767000 |
| H | -3.842681000 | 7.330909000 | -2.270685000 |
| H | -2.529182000 | 6.606293000 | -1.323457000 |
| H | -2.798719000 | 8.358308000 | -1.272240000 |
| H | -4.139702000 | 7.196900000 | 2.063089000 |
| H | -2.976391000 | 8.282318000 | 1.281789000 |
| H | -2.704641000 | 6.529981000 | 1.261504000 |
| H | 2.161556000 | 5.094103000 | -1.012418000 |
| H | 2.275712000 | 5.129632000 | 0.767007000 |
| H | 1.087551000 | 8.543592000 | -0.114975000 |
| H | 0.728374000 | 7.093557000 | 0.837049000 |
| H | 0.601217000 | 7.049745000 | -0.933534000 |
| H | 3.024915000 | 7.265181000 | 1.958522000 |
| H | 3.382486000 | 8.718768000 | 1.009060000 |
| H | 4.498009000 | 7.340986000 | 0.972814000 |
| H | 2.714197000 | 7.179245000 | -2.375076000 |
| H | 4.313797000 | 7.277361000 | -1.613827000 |
| H | 3.211082000 | 8.664614000 | -1.544848000 |
| H | 11.498663000 | -1.026024000 | 0.053227000 |
| N | 3.865443000 | -3.277512000 | 0.351599000 |
| C | 3.702117000 | -4.632156000 | 0.327560000 |
| C | 4.955044000 | -5.214795000 | 0.346097000 |
| C | 5.862761000 | -4.141931000 | 0.358819000 |
| N | 5.213969000 | -2.974821000 | 0.349461000 |
| N | -3.526328000 | -3.486007000 | 0.346880000 |
| C | -3.287261000 | -4.829386000 | 0.324646000 |
| C | -4.505641000 | -5.481190000 | 0.337407000 |
| C | -5.472180000 | -4.460941000 | 0.344827000 |
| N | -4.889746000 | -3.259242000 | 0.337724000 |
| H | 6.943317000 | -4.152881000 | 0.361254000 |
| H | 5.179679000 | -6.270745000 | 0.341579000 |
| H | 2.727679000 | -5.084554000 | 0.265439000 |
| H | -2.288806000 | -5.226875000 | 0.268546000 |
| H | -4.670751000 | -6.548068000 | 0.332597000 |
| H | -6.550361000 | -4.533511000 | 0.342866000 |

| | | | |
|---|---|---|---|
| Ru | -5.418906000 | -1.258946000 | 0.029334000 |
| C | -5.292620000 | -1.927396000 | -4.325922000 |
| C | -4.927047000 | -1.697121000 | -3.004088000 |
| N | -5.821844000 | -1.604015000 | -2.000549000 |
| C | -7.157881000 | -1.742033000 | -2.299099000 |
| C | -7.581050000 | -1.974589000 | -3.610198000 |
| C | -6.645134000 | -2.069395000 | -4.635970000 |
| C | -8.086783000 | -1.625802000 | -1.157097000 |
| C | -9.479105000 | -1.743275000 | -1.187370000 |
| C | -10.190865000 | -1.603757000 | 0.006124000 |
| C | -9.519078000 | -1.352047000 | 1.204503000 |
| C | -8.126035000 | -1.242860000 | 1.183871000 |
| N | -7.455546000 | -1.381457000 | 0.015584000 |
| C | -7.234945000 | -0.986155000 | 2.333312000 |
| N | -5.889126000 | -0.947461000 | 2.049767000 |
| C | -5.026658000 | -0.713825000 | 3.058656000 |
| C | -5.435022000 | -0.512586000 | 4.372717000 |
| C | -6.797615000 | -0.551992000 | 4.668075000 |
| C | -7.700533000 | -0.790337000 | 3.635950000 |
| H | -4.525500000 | -1.993154000 | -5.089664000 |
| H | -3.888942000 | -1.580439000 | -2.716534000 |
| H | -8.637981000 | -2.079930000 | -3.825502000 |
| H | -6.967481000 | -2.249781000 | -5.656170000 |
| H | -10.004609000 | -1.939780000 | -2.114641000 |
| H | -11.272007000 | -1.692196000 | 0.002513000 |
| H | -10.076109000 | -1.246347000 | 2.128143000 |
| H | -3.979021000 | -0.691027000 | 2.782810000 |
| H | -4.692753000 | -0.329030000 | 5.141747000 |
| H | -7.153059000 | -0.399375000 | 5.681793000 |
| H | -8.764479000 | -0.822978000 | 3.839560000 |

Cartesian coordinates of DFT-optimized structure of **7aox** complex by B3LYP/Def2-TZVP; 6-31G(d,p)/$CH_3CN$, charge = 3, multiplicity = 2

| | | | |
|---|---|---|---|
| Os | -5.504546000 | -0.051799000 | 0.032316000 |
| Os | 5.495434000 | 0.096412000 | 0.001501000 |
| N | -5.064135000 | -2.104594000 | 0.293274000 |
| N | 5.055101000 | 2.149257000 | -0.259401000 |
| N | -5.975501000 | 0.236266000 | 2.059907000 |
| N | 5.965917000 | -0.191717000 | -2.026200000 |
| N | -7.584088000 | -0.081493000 | 0.045856000 |
| N | 7.575056000 | 0.126023000 | -0.012600000 |
| N | -5.992539000 | -0.354628000 | -1.989984000 |
| N | 5.984061000 | 0.399271000 | 2.023647000 |
| N | -5.125921000 | 2.012358000 | -0.236210000 |
| N | 5.116833000 | -1.967743000 | 0.270192000 |
| C | -5.084230000 | 0.398286000 | 3.064436000 |
| C | 5.074354000 | -0.353725000 | -3.030470000 |
| H | 4.031084000 | -0.324210000 | -2.743370000 |
| C | -5.466786000 | 0.590312000 | 4.385176000 |

| | | | |
|---|---|---|---|
| C | 5.456543000 | -0.545813000 | -4.351310000 |
| H | 4.694118000 | -0.669709000 | -5.112088000 |
| C | -6.825452000 | 0.619379000 | 4.700484000 |
| C | 6.815117000 | -0.574958000 | -4.666985000 |
| C | -7.753159000 | 0.454052000 | 3.677239000 |
| C | 7.743115000 | -0.409640000 | -3.644001000 |
| H | -8.814328000 | 0.473367000 | 3.894878000 |
| C | -7.320130000 | 0.263043000 | 2.363810000 |
| C | 7.310455000 | -0.218563000 | -2.330465000 |
| C | -8.235351000 | 0.079283000 | 1.225048000 |
| C | 8.225988000 | -0.034802000 | -1.191953000 |
| C | -9.631258000 | 0.055668000 | 1.258326000 |
| C | 9.621886000 | -0.011229000 | -1.225613000 |
| H | -10.169806000 | 0.180929000 | 2.190045000 |
| H | 10.160180000 | -0.136521000 | -2.157475000 |
| C | -10.330189000 | -0.134122000 | 0.064686000 |
| C | 10.321141000 | 0.178565000 | -0.032165000 |
| C | -9.641272000 | -0.297920000 | -1.138459000 |
| C | 9.632554000 | 0.342411000 | 1.171160000 |
| C | -8.245081000 | -0.268501000 | -1.123850000 |
| C | 8.236359000 | 0.313038000 | 1.156916000 |
| C | -7.339836000 | -0.422846000 | -2.274759000 |
| C | 7.331427000 | 0.467450000 | 2.308064000 |
| C | -7.785946000 | -0.625962000 | -3.581917000 |
| C | 7.777888000 | 0.670593000 | 3.615092000 |
| H | -8.849176000 | -0.676898000 | -3.783792000 |
| C | -6.868701000 | -0.762930000 | -4.618629000 |
| C | 6.860916000 | 0.807620000 | 4.652040000 |
| C | -5.507215000 | -0.692262000 | -4.322771000 |
| C | 5.499353000 | 0.736985000 | 4.356543000 |
| H | 4.744960000 | 0.837262000 | 5.128753000 |
| C | -5.111545000 | -0.488696000 | -3.007523000 |
| C | 5.103328000 | 0.533394000 | 3.041403000 |
| H | 4.057360000 | 0.471446000 | 2.769986000 |
| C | -5.961169000 | -3.114714000 | 0.321701000 |
| C | 5.952151000 | 3.159353000 | -0.287933000 |
| H | -7.004575000 | -2.822897000 | 0.338505000 |
| H | 6.995548000 | 2.867518000 | -0.304898000 |
| C | -5.611173000 | -4.449412000 | 0.319084000 |
| C | 5.602176000 | 4.494060000 | -0.285236000 |
| H | -6.368738000 | -5.223767000 | 0.342961000 |
| H | 6.359751000 | 5.268401000 | -0.309207000 |
| C | -4.249368000 | -4.785514000 | 0.248431000 |
| C | 4.240388000 | 4.830185000 | -0.214398000 |
| C | -3.306392000 | -3.750118000 | 0.224702000 |
| C | 3.297392000 | 3.794800000 | -0.190559000 |
| H | -2.257593000 | -3.976082000 | 0.117616000 |
| H | 2.248607000 | 4.020773000 | -0.083349000 |
| C | -3.716965000 | -2.412757000 | 0.288147000 |
| C | 3.707944000 | 2.457446000 | -0.254062000 |
| C | -2.826991000 | -1.236763000 | 0.244284000 |

| | | | |
|---|---|---|---|
| C | 2.817937000 | 1.281467000 | -0.210057000 |
| C | -3.539863000 | -0.025792000 | 0.023963000 |
| C | 3.530776000 | 0.070476000 | 0.010245000 |
| C | -2.860796000 | 1.203321000 | -0.201292000 |
| C | 2.851723000 | -1.158624000 | 0.235592000 |
| C | -3.780413000 | 2.356808000 | -0.244721000 |
| C | 3.771317000 | -2.312143000 | 0.278946000 |
| C | -3.417524000 | 3.700245000 | -0.197182000 |
| C | 3.408365000 | -3.655562000 | 0.231574000 |
| H | -2.382288000 | 3.995507000 | -0.106116000 |
| H | 2.373099000 | -3.950791000 | 0.140727000 |
| C | -4.387538000 | 4.715227000 | -0.223014000 |
| C | 4.378354000 | -4.670579000 | 0.257303000 |
| C | -5.738374000 | 4.347558000 | -0.278154000 |
| C | 5.729211000 | -4.302955000 | 0.312180000 |
| H | -6.542056000 | 5.070910000 | -0.305206000 |
| H | 6.532871000 | -5.026333000 | 0.339146000 |
| C | -6.044032000 | 2.994343000 | -0.265871000 |
| C | 6.034916000 | -2.949750000 | 0.299756000 |
| H | -7.079778000 | 2.675672000 | -0.270569000 |
| H | 7.070674000 | -2.631116000 | 0.304256000 |
| C | -1.458743000 | 1.197493000 | -0.363158000 |
| C | 1.449680000 | -1.152764000 | 0.397528000 |
| C | -0.717988000 | 0.012241000 | 0.017878000 |
| C | 0.708930000 | 0.032497000 | 0.016491000 |
| C | -1.424543000 | -1.193221000 | 0.401010000 |
| C | 1.415483000 | 1.237953000 | -0.366685000 |
| C | -0.651658000 | -2.251997000 | 0.982728000 |
| C | 0.642595000 | 2.296746000 | -0.948364000 |
| H | -1.160132000 | -3.058473000 | 1.496195000 |
| H | 1.151067000 | 3.103230000 | -1.461823000 |
| C | 0.708777000 | -2.233165000 | 0.980282000 |
| C | -0.717838000 | 2.277902000 | -0.945905000 |
| H | 1.242091000 | -3.025894000 | 1.489546000 |
| H | -1.251152000 | 3.070630000 | -1.455167000 |
| O | -3.954861000 | -6.094793000 | 0.199273000 |
| O | 3.918199000 | -5.931626000 | 0.217705000 |
| O | 3.945902000 | 6.139457000 | -0.165191000 |
| O | -3.927434000 | 5.976296000 | -0.183239000 |
| C | -4.869004000 | 7.069529000 | -0.193139000 |
| C | -4.101997000 | 8.398202000 | -0.142567000 |
| C | -5.154642000 | 9.524226000 | -0.146182000 |
| C | -3.194168000 | 8.532214000 | -1.379831000 |
| C | -3.261662000 | 8.477637000 | 1.145962000 |
| C | 2.570090000 | 6.560596000 | -0.076097000 |
| C | 2.514238000 | 8.095057000 | -0.062909000 |
| C | 1.029064000 | 8.490487000 | 0.051562000 |
| C | 3.292726000 | 8.643263000 | 1.148101000 |
| C | 3.103157000 | 8.655923000 | -1.371222000 |
| C | -2.579037000 | -6.515911000 | 0.110350000 |
| C | -2.523158000 | -8.050373000 | 0.097293000 |

| | | | |
|---|---|---|---|
| C | -3.112141000 | -8.611133000 | 1.405623000 |
| C | -3.301573000 | -8.598697000 | -1.113709000 |
| C | -1.037974000 | -8.445795000 | -0.017062000 |
| C | 4.859731000 | -7.024898000 | 0.227513000 |
| C | 4.092664000 | -8.353544000 | 0.177180000 |
| C | 3.252090000 | -8.433039000 | -1.111189000 |
| C | 3.185060000 | -8.487434000 | 1.414622000 |
| C | 5.145269000 | -9.479606000 | 0.180682000 |
| H | -5.532913000 | 6.973128000 | 0.675234000 |
| H | -5.472742000 | 7.009201000 | -1.107400000 |
| H | -5.817579000 | 9.455680000 | 0.724814000 |
| H | -5.774726000 | 9.490744000 | -1.050181000 |
| H | -4.661004000 | 10.501949000 | -0.115038000 |
| H | -3.780492000 | 8.490174000 | -2.306169000 |
| H | -2.447308000 | 7.732184000 | -1.414366000 |
| H | -2.663706000 | 9.491170000 | -1.361216000 |
| H | -3.896152000 | 8.394818000 | 2.037101000 |
| H | -2.733971000 | 9.436901000 | 1.197440000 |
| H | -2.515476000 | 7.677378000 | 1.184991000 |
| H | 2.019043000 | 6.162717000 | -0.937867000 |
| H | 2.131740000 | 6.151121000 | 0.842989000 |
| H | 0.929185000 | 9.581602000 | 0.063173000 |
| H | 0.580753000 | 8.103387000 | 0.974752000 |
| H | 0.445833000 | 8.110269000 | -0.795917000 |
| H | 2.882352000 | 8.257488000 | 2.089532000 |
| H | 3.229465000 | 9.737107000 | 1.177199000 |
| H | 4.350491000 | 8.365550000 | 1.099387000 |
| H | 2.549692000 | 8.287064000 | -2.243740000 |
| H | 4.153183000 | 8.369228000 | -1.488703000 |
| H | 3.047407000 | 9.750478000 | -1.376288000 |
| H | -2.028078000 | -6.117957000 | 0.972142000 |
| H | -2.140603000 | -6.106498000 | -0.808724000 |
| H | -2.558726000 | -8.242193000 | 2.278139000 |
| H | -3.056381000 | -9.705686000 | 1.410786000 |
| H | -4.162176000 | -8.324435000 | 1.523022000 |
| H | -4.359342000 | -8.320986000 | -1.065081000 |
| H | -3.238302000 | -9.692544000 | -1.142702000 |
| H | -2.891148000 | -8.213007000 | -2.055154000 |
| H | -0.454788000 | -8.065483000 | 0.830406000 |
| H | -0.589621000 | -8.058788000 | -0.940270000 |
| H | -0.938079000 | -9.536911000 | -0.028554000 |
| H | 5.463639000 | -6.964520000 | 1.141659000 |
| H | 5.523481000 | -6.928582000 | -0.640990000 |
| H | 2.724341000 | -9.392280000 | -1.162496000 |
| H | 3.886422000 | -8.350321000 | -2.002450000 |
| H | 2.505936000 | -7.632745000 | -1.150145000 |
| H | 2.438221000 | -7.687387000 | 1.449226000 |
| H | 3.771554000 | -8.445327000 | 2.340849000 |
| H | 2.654576000 | -9.446381000 | 1.396183000 |
| H | 5.808054000 | -9.411141000 | -0.690437000 |
| H | 4.651589000 | -10.457313000 | 0.149688000 |

| | | | |
|---|---|---|---|
| H | 5.765514000 | -9.446089000 | 1.084568000 |
| H | -4.704578000 | 0.714222000 | 5.146170000 |
| H | -7.157836000 | 0.767634000 | 5.722273000 |
| H | -4.040881000 | 0.368834000 | 2.777613000 |
| H | -10.186483000 | -0.444773000 | -2.063163000 |
| H | -11.414438000 | -0.154586000 | 0.072322000 |
| H | -7.211052000 | -0.920971000 | -5.635651000 |
| H | -4.752617000 | -0.792496000 | -5.094788000 |
| H | -4.065652000 | -0.426722000 | -2.735820000 |
| H | 7.147216000 | -0.723266000 | -5.688860000 |
| H | 8.804223000 | -0.429021000 | -3.861930000 |
| H | 11.405388000 | 0.198996000 | -0.040093000 |
| H | 10.178012000 | 0.489266000 | 2.095717000 |
| H | 8.841172000 | 0.721501000 | 3.816686000 |
| H | 7.203537000 | 0.965681000 | 5.668967000 |

Cartesian coordinates of DFT-optimized structure of **8aox** complex by B3LYP/Def2-TZVP; 6-31G(d,p)/$CH_3CN$, charge = 3, multiplicity = 2

| | | | |
|---|---|---|---|
| Os | 5.421125000 | 0.949889000 | 0.030352000 |
| N | 5.317845000 | -1.116797000 | 0.369302000 |
| N | 5.828650000 | 1.385108000 | 2.050678000 |
| N | 7.474476000 | 1.286099000 | 0.036246000 |
| N | 5.957453000 | 0.669181000 | -1.987265000 |
| C | 4.924125000 | 1.419245000 | 3.053796000 |
| H | 3.901765000 | 1.197356000 | 2.775843000 |
| C | 5.268184000 | 1.716888000 | 4.366247000 |
| H | 4.496735000 | 1.729094000 | 5.127834000 |
| C | 6.600462000 | 1.992686000 | 4.671191000 |
| H | 6.903076000 | 2.228684000 | 5.685682000 |
| C | 7.542231000 | 1.959602000 | 3.647176000 |
| H | 8.584105000 | 2.169081000 | 3.857667000 |
| C | 7.147099000 | 1.655932000 | 2.343713000 |
| C | 8.081231000 | 1.600296000 | 1.205019000 |
| C | 9.458364000 | 1.830720000 | 1.233769000 |
| H | 9.963476000 | 2.082636000 | 2.158404000 |
| C | 10.181161000 | 1.729459000 | 0.043976000 |
| C | 9.535071000 | 1.403981000 | -1.149729000 |
| H | 10.099290000 | 1.327232000 | -2.071312000 |
| C | 8.156253000 | 1.182978000 | -1.128850000 |
| C | 7.295034000 | 0.833311000 | -2.272685000 |
| C | 7.773663000 | 0.672712000 | -3.573638000 |
| H | 8.829094000 | 0.806678000 | -3.778114000 |
| C | 6.897374000 | 0.341894000 | -4.602866000 |
| H | 7.265118000 | 0.215666000 | -5.615433000 |
| C | 5.545037000 | 0.178057000 | -4.305619000 |
| H | 4.822043000 | -0.078887000 | -5.071489000 |
| C | 5.116685000 | 0.349216000 | -2.995382000 |
| H | 4.075415000 | 0.233159000 | -2.723351000 |
| N | 4.021824000 | -1.616320000 | 0.385821000 |

| | | | |
|---|---|---|---|
| C | -2.990920000 | 0.782690000 | -0.232863000 |
| C | 2.973308000 | -0.660351000 | 0.262346000 |
| C | -3.498072000 | -0.509906000 | 0.004774000 |
| C | 3.480462000 | 0.632247000 | 0.024747000 |
| C | -2.606643000 | -1.573603000 | 0.246374000 |
| C | 2.589036000 | 1.695942000 | -0.216895000 |
| C | -1.227555000 | -1.360334000 | 0.381680000 |
| C | 1.209952000 | 1.482660000 | -0.352231000 |
| C | -0.712074000 | -0.053940000 | 0.012711000 |
| C | 0.694467000 | 0.176265000 | 0.016737000 |
| C | -1.615066000 | 1.020299000 | -0.360316000 |
| C | 1.597455000 | -0.897974000 | 0.389766000 |
| C | -1.048959000 | 2.227560000 | -0.885773000 |
| C | 1.031356000 | -2.105254000 | 0.915188000 |
| H | -1.690437000 | 2.955949000 | -1.363583000 |
| H | 1.672846000 | -2.833656000 | 1.392965000 |
| C | 0.291457000 | 2.446941000 | -0.881937000 |
| C | -0.309060000 | -2.324635000 | 0.911352000 |
| H | 0.669915000 | 3.342260000 | -1.356828000 |
| H | -0.687525000 | -3.219969000 | 1.386211000 |
| H | 11.251393000 | 1.904761000 | 0.047032000 |
| N | -3.296370000 | -2.814065000 | 0.364867000 |
| N | -4.683902000 | -2.753749000 | 0.340669000 |
| C | -5.117790000 | -4.015623000 | 0.383756000 |
| C | -4.035210000 | -4.910784000 | 0.415184000 |
| C | -2.903648000 | -4.122074000 | 0.387895000 |
| H | -6.179232000 | -4.216541000 | 0.377548000 |
| H | -4.073265000 | -5.989014000 | 0.443288000 |
| H | -1.865805000 | -4.403470000 | 0.349621000 |
| Os | -5.438742000 | -0.827508000 | -0.000730000 |
| C | -5.562465000 | -0.055741000 | 4.335250000 |
| C | -5.134161000 | -0.226970000 | 3.025009000 |
| N | -5.975003000 | -0.546762000 | 2.016895000 |
| C | -7.312610000 | -0.710645000 | 2.302329000 |
| C | -7.791193000 | -0.549978000 | 3.603292000 |
| C | -6.914831000 | -0.219330000 | 4.632512000 |
| C | -8.173905000 | -1.060129000 | 1.158495000 |
| N | -7.492139000 | -1.163435000 | -0.006591000 |
| C | -8.098965000 | -1.477452000 | -1.175375000 |
| C | -9.476157000 | -1.707516000 | -1.204142000 |
| C | -10.198941000 | -1.606081000 | -0.014355000 |
| C | -9.552781000 | -1.280771000 | 1.179357000 |
| C | -7.164847000 | -1.533280000 | -2.314072000 |
| N | -5.846345000 | -1.262698000 | -2.021050000 |
| C | -4.941835000 | -1.297017000 | -3.024173000 |
| C | -5.285963000 | -1.594600000 | -4.336621000 |
| C | -6.618293000 | -1.870153000 | -4.641552000 |
| C | -7.560047000 | -1.836890000 | -3.617527000 |
| H | -4.092873000 | -0.111108000 | 2.752963000 |
| H | -4.839415000 | 0.201062000 | 5.101116000 |
| H | -7.282541000 | -0.093046000 | 5.645084000 |

| | | | |
|---|---|---|---|
| H | -8.846644000 | -0.683761000 | 3.807780000 |
| H | -10.116993000 | -1.203871000 | 2.100933000 |
| H | -11.269218000 | -1.781108000 | -0.017425000 |
| H | -9.981325000 | -1.959285000 | -2.128786000 |
| H | -8.601960000 | -2.046188000 | -3.828006000 |
| H | -6.920959000 | -2.106100000 | -5.656039000 |
| H | -4.514523000 | -1.606953000 | -5.098215000 |
| H | -3.919429000 | -1.075321000 | -2.746236000 |
| N | -4.039433000 | 1.738665000 | -0.356310000 |
| N | -5.335463000 | 1.239150000 | -0.339718000 |
| C | -6.148734000 | 2.296902000 | -0.386507000 |
| C | -5.407844000 | 3.490497000 | -0.412842000 |
| C | -4.084058000 | 3.103641000 | -0.378549000 |
| N | 4.666294000 | 2.876102000 | -0.311098000 |
| H | -3.190304000 | 3.701182000 | -0.334814000 |
| H | -5.787483000 | 4.500362000 | -0.442224000 |
| H | -7.218864000 | 2.148964000 | -0.386345000 |
| N | 3.278758000 | 2.936407000 | -0.335370000 |
| C | 5.100178000 | 4.137972000 | -0.354321000 |
| C | 4.017596000 | 5.033125000 | -0.385878000 |
| C | 2.886035000 | 4.244412000 | -0.358609000 |
| C | 4.066449000 | -2.981300000 | 0.407862000 |
| C | 5.390235000 | -3.368153000 | 0.442175000 |
| C | 6.131121000 | -2.174553000 | 0.415961000 |
| H | 4.055650000 | 6.111352000 | -0.414067000 |
| H | 1.848193000 | 4.525814000 | -0.320402000 |
| H | 6.161618000 | 4.338900000 | -0.348059000 |
| H | 7.201251000 | -2.026605000 | 0.415852000 |
| H | 5.769876000 | -4.378020000 | 0.471477000 |
| H | 3.172692000 | -3.578833000 | 0.364065000 |

Cartesian coordinates of DFT-optimized structure of **9aox** complex by B3LYP/Def2-TZVP; 6-31G(d,p)/$CH_3CN$, charge = 3, multiplicity = 2

| | | | |
|---|---|---|---|
| Os | 5.500286000 | 0.877132000 | -0.243143000 |
| N | 5.418244000 | -1.197673000 | 0.179266000 |
| N | 5.953177000 | 1.387030000 | 1.746754000 |
| N | 7.571201000 | 1.198635000 | -0.273936000 |
| N | 6.029123000 | 0.509351000 | -2.245272000 |
| C | 5.054219000 | 1.468297000 | 2.753542000 |
| H | 4.028015000 | 1.245803000 | 2.491621000 |
| C | 5.411296000 | 1.814397000 | 4.050111000 |
| H | 4.645483000 | 1.863061000 | 4.815807000 |
| C | 6.748178000 | 2.090272000 | 4.334820000 |
| H | 7.059946000 | 2.362484000 | 5.337334000 |
| C | 7.682147000 | 2.010714000 | 3.306582000 |
| H | 8.726825000 | 2.221541000 | 3.501089000 |
| C | 7.275327000 | 1.659329000 | 2.018870000 |
| C | 8.196219000 | 1.552828000 | 0.874338000 |
| C | 9.575249000 | 1.773105000 | 0.878125000 |

| | | | |
|---|---|---|---|
| H | 10.093135000 | 2.056768000 | 1.786252000 |
| C | 10.282753000 | 1.621193000 | -0.315530000 |
| C | 9.619802000 | 1.255239000 | -1.488038000 |
| H | 10.171855000 | 1.137200000 | -2.412573000 |
| C | 8.239826000 | 1.045512000 | -1.441740000 |
| C | 7.362290000 | 0.654160000 | -2.557794000 |
| C | 7.819890000 | 0.433747000 | -3.857532000 |
| H | 8.872572000 | 0.553536000 | -4.083516000 |
| C | 6.926420000 | 0.061280000 | -4.856909000 |
| H | 7.277419000 | -0.110581000 | -5.868640000 |
| C | 5.578543000 | -0.085734000 | -4.531131000 |
| H | 4.842986000 | -0.375170000 | -5.272978000 |
| C | 5.170235000 | 0.145058000 | -3.224084000 |
| H | 4.133640000 | 0.042862000 | -2.930621000 |
| C | 6.471014000 | -2.038746000 | 0.264658000 |
| H | 7.450703000 | -1.576342000 | 0.243122000 |
| C | 6.347215000 | -3.409549000 | 0.365285000 |
| H | 7.222566000 | -4.044477000 | 0.432159000 |
| C | 5.059739000 | -3.969626000 | 0.344261000 |
| C | 3.956285000 | -3.108328000 | 0.264543000 |
| H | 2.958247000 | -3.511048000 | 0.196654000 |
| C | 4.141411000 | -1.722298000 | 0.221626000 |
| C | -2.904073000 | 0.741640000 | -0.522856000 |
| C | 3.067098000 | -0.714597000 | 0.107608000 |
| C | -3.425006000 | -0.500976000 | -0.119978000 |
| C | 3.560319000 | 0.576600000 | -0.218814000 |
| C | -2.542621000 | -1.526976000 | 0.262836000 |
| C | 2.691903000 | 1.656381000 | -0.527427000 |
| C | -1.158211000 | -1.312633000 | 0.368112000 |
| C | 1.310109000 | 1.403473000 | -0.671849000 |
| C | -0.631239000 | -0.070321000 | -0.159839000 |
| C | 0.779877000 | 0.146805000 | -0.180420000 |
| C | -1.522658000 | 0.953751000 | -0.666946000 |
| C | 1.677533000 | -0.889403000 | 0.291291000 |
| C | -0.940152000 | 2.080269000 | -1.332190000 |
| C | 1.099378000 | -2.008454000 | 0.974642000 |
| H | -1.569572000 | 2.747501000 | -1.906093000 |
| H | 1.741256000 | -2.677696000 | 1.533270000 |
| C | 0.402912000 | 2.296062000 | -1.330070000 |
| C | -0.245791000 | -2.207194000 | 1.014679000 |
| H | 0.797927000 | 3.128311000 | -1.898341000 |
| H | -0.629476000 | -3.027698000 | 1.606706000 |
| O | 4.985686000 | -5.307854000 | 0.394820000 |
| C | 3.698100000 | -5.957514000 | 0.364290000 |
| C | 3.901073000 | -7.476667000 | 0.453974000 |
| C | 2.501846000 | -8.121403000 | 0.408409000 |
| C | 4.740129000 | -7.970744000 | -0.739805000 |
| C | 4.597745000 | -7.839151000 | 1.779272000 |
| H | 3.098250000 | -5.597598000 | 1.209867000 |
| H | 3.188165000 | -5.691029000 | -0.570074000 |
| H | 2.587603000 | -9.211766000 | 0.473684000 |

| | | | |
|---|---|---|---|
| H | 1.980096000 | -7.881830000 | -0.526109000 |
| H | 1.876958000 | -7.783793000 | 1.244152000 |
| H | 4.257060000 | -7.721964000 | -1.692848000 |
| H | 4.857655000 | -9.059626000 | -0.696887000 |
| H | 5.738487000 | -7.521685000 | -0.737328000 |
| H | 4.005607000 | -7.505989000 | 2.640674000 |
| H | 5.587456000 | -7.376244000 | 1.847244000 |
| H | 4.724176000 | -8.924965000 | 1.858254000 |
| H | 11.354119000 | 1.788241000 | -0.331917000 |
| N | -3.244048000 | -2.733808000 | 0.546552000 |
| N | -4.631797000 | -2.663241000 | 0.512938000 |
| C | -5.078244000 | -3.902914000 | 0.730296000 |
| C | -4.004825000 | -4.796127000 | 0.886067000 |
| C | -2.865054000 | -4.030403000 | 0.751343000 |
| H | -6.141786000 | -4.091193000 | 0.749815000 |
| H | -4.053741000 | -5.859861000 | 1.062304000 |
| H | -1.830557000 | -4.325781000 | 0.752853000 |
| Os | -5.378351000 | -0.804201000 | -0.095036000 |
| C | -5.518667000 | 0.593183000 | 4.085837000 |
| C | -5.079948000 | 0.228353000 | 2.819592000 |
| N | -5.911767000 | -0.230880000 | 1.858245000 |
| C | -7.255035000 | -0.339910000 | 2.151295000 |
| C | -7.744558000 | 0.014914000 | 3.409725000 |
| C | -6.875847000 | 0.485560000 | 4.389348000 |
| C | -8.110736000 | -0.845641000 | 1.063581000 |
| N | -7.419931000 | -1.121141000 | -0.070187000 |
| C | -8.022138000 | -1.597141000 | -1.187785000 |
| C | -9.401095000 | -1.818386000 | -1.193533000 |
| C | -10.131997000 | -1.541669000 | -0.036595000 |
| C | -9.491390000 | -1.052198000 | 1.103339000 |
| C | -7.079952000 | -1.822934000 | -2.297780000 |
| N | -5.760213000 | -1.519008000 | -2.037216000 |
| C | -4.852138000 | -1.705205000 | -3.020986000 |
| C | -5.190360000 | -2.187686000 | -4.278648000 |
| C | -6.523175000 | -2.497601000 | -4.549152000 |
| C | -7.469460000 | -2.311310000 | -3.546346000 |
| H | -4.035436000 | 0.297726000 | 2.543157000 |
| H | -4.800996000 | 0.954741000 | 4.813744000 |
| H | -7.252457000 | 0.762155000 | 5.368313000 |
| H | -8.803490000 | -0.077604000 | 3.619596000 |
| H | -10.062182000 | -0.839682000 | 1.999360000 |
| H | -11.203637000 | -1.707967000 | -0.023234000 |
| H | -9.902247000 | -2.197549000 | -2.076135000 |
| H | -8.511380000 | -2.544429000 | -3.730389000 |
| H | -6.821941000 | -2.876602000 | -5.520647000 |
| H | -4.414952000 | -2.314926000 | -5.025755000 |
| H | -3.829483000 | -1.453232000 | -2.770212000 |
| N | -3.945141000 | 1.678703000 | -0.783611000 |
| N | -5.245247000 | 1.191555000 | -0.713864000 |
| C | -6.051364000 | 2.236880000 | -0.916579000 |
| C | -5.302930000 | 3.412073000 | -1.098322000 |

| | | | |
|---|---|---|---|
| C | -3.981753000 | 3.027929000 | -0.995365000 |
| C | 3.405621000 | 2.942888000 | -0.665046000 |
| N | 4.789417000 | 2.832060000 | -0.654901000 |
| C | 5.529328000 | 3.948541000 | -0.761746000 |
| C | 4.999361000 | 5.227942000 | -0.855576000 |
| C | 3.606320000 | 5.364773000 | -0.801870000 |
| C | 2.820591000 | 4.203819000 | -0.699257000 |
| O | 2.938428000 | 6.527183000 | -0.831277000 |
| C | 3.674774000 | 7.765828000 | -0.916359000 |
| C | 2.686154000 | 8.940028000 | -0.903133000 |
| C | 3.522154000 | 10.231736000 | -0.996705000 |
| C | 1.878287000 | 8.937424000 | 0.408523000 |
| C | 1.736460000 | 8.846573000 | -2.112373000 |
| H | 4.261997000 | 7.765327000 | -1.843258000 |
| H | 4.361367000 | 7.828599000 | -0.062884000 |
| H | 4.113686000 | 10.260808000 | -1.919868000 |
| H | 4.210413000 | 10.327065000 | -0.148036000 |
| H | 2.863902000 | 11.107733000 | -0.993420000 |
| H | 2.539993000 | 9.010030000 | 1.280507000 |
| H | 1.284529000 | 8.023020000 | 0.507746000 |
| H | 1.192816000 | 9.792187000 | 0.434803000 |
| H | 2.295758000 | 8.857054000 | -3.056028000 |
| H | 1.046479000 | 9.698115000 | -2.121158000 |
| H | 1.141546000 | 7.928126000 | -2.080005000 |
| H | 5.668537000 | 6.073490000 | -0.942310000 |
| H | 6.603764000 | 3.806676000 | -0.762651000 |
| H | 1.750482000 | 4.326075000 | -0.613494000 |
| H | -3.085603000 | 3.622654000 | -1.023319000 |
| H | -5.675922000 | 4.410171000 | -1.270592000 |
| H | -7.122284000 | 2.094657000 | -0.908651000 |

Cartesian coordinates of DFT-optimized structure of **10aox** complex by B3LYP/Def2-TZVP; 6-31G(d,p)/$CH_3CN$, charge = 3, multiplicity = 2

| | | | |
|---|---|---|---|
| Os | -2.286521000 | -1.285446000 | 0.065875000 |
| N | -3.054971000 | 0.671383000 | 0.284719000 |
| N | -2.575741000 | -1.761471000 | 2.096055000 |
| N | -4.019239000 | -2.437026000 | 0.040129000 |
| N | -2.806462000 | -1.323335000 | -1.972550000 |
| C | -1.792267000 | -1.366035000 | 3.124270000 |
| H | -0.945694000 | -0.745379000 | 2.860202000 |
| C | -2.044474000 | -1.723333000 | 4.442441000 |
| H | -1.378159000 | -1.375396000 | 5.223707000 |
| C | -3.151230000 | -2.522290000 | 4.728297000 |
| H | -3.376727000 | -2.819487000 | 5.746813000 |
| C | -3.968296000 | -2.932923000 | 3.679702000 |
| H | -4.836285000 | -3.551224000 | 3.874555000 |
| C | -3.673567000 | -2.547994000 | 2.371001000 |
| C | -4.490237000 | -2.937061000 | 1.208255000 |
| C | -5.638793000 | -3.731653000 | 1.212517000 |

| | | | |
|---|---|---|---|
| H | -6.031690000 | -4.140152000 | 2.135846000 |
| C | -6.278330000 | -3.995165000 | -0.000107000 |
| C | -5.774939000 | -3.472033000 | -1.192489000 |
| H | -6.272988000 | -3.680492000 | -2.131726000 |
| C | -4.624105000 | -2.682225000 | -1.147539000 |
| C | -3.939178000 | -2.043678000 | -2.284535000 |
| C | -4.384914000 | -2.140373000 | -3.603720000 |
| H | -5.277184000 | -2.712304000 | -3.828269000 |
| C | -3.686165000 | -1.503005000 | -4.624253000 |
| H | -4.028975000 | -1.575491000 | -5.650758000 |
| C | -2.542747000 | -0.773163000 | -4.299906000 |
| H | -1.963797000 | -0.257842000 | -5.057949000 |
| C | -2.137780000 | -0.707427000 | -2.973229000 |
| H | -1.255320000 | -0.153366000 | -2.680106000 |
| C | -4.362192000 | 1.014805000 | 0.277423000 |
| H | -5.067187000 | 0.192065000 | 0.288950000 |
| C | -4.810931000 | 2.318894000 | 0.246603000 |
| H | -5.870960000 | 2.543216000 | 0.241685000 |
| C | -3.862217000 | 3.352891000 | 0.186592000 |
| C | -2.503160000 | 3.014746000 | 0.204292000 |
| H | -1.754945000 | 3.784618000 | 0.106786000 |
| C | -2.103343000 | 1.675892000 | 0.293642000 |
| C | 5.429594000 | 2.119191000 | 0.142571000 |
| C | -0.708676000 | 1.190186000 | 0.298808000 |
| C | 5.361638000 | 3.522216000 | 0.342092000 |
| C | -0.640697000 | -0.212857000 | 0.099473000 |
| C | 4.113020000 | 4.162863000 | 0.503448000 |
| C | 0.607926000 | -0.853419000 | -0.062228000 |
| C | 2.920556000 | 3.433600000 | 0.604892000 |
| C | 1.800315000 | -0.124069000 | -0.164014000 |
| C | 2.966759000 | 2.029055000 | 0.248384000 |
| C | 1.754118000 | 1.280488000 | 0.192461000 |
| C | 4.234666000 | 1.389215000 | -0.048132000 |
| C | 0.486233000 | 1.920297000 | 0.489076000 |
| C | 4.191479000 | 0.063232000 | -0.592422000 |
| C | 0.529363000 | 3.246383000 | 1.033104000 |
| H | 5.085089000 | -0.356000000 | -1.035824000 |
| H | -0.364303000 | 3.665702000 | 1.476320000 |
| C | 3.039991000 | -0.658394000 | -0.642344000 |
| C | 1.680841000 | 3.968031000 | 1.082988000 |
| H | 3.055071000 | -1.628832000 | -1.121915000 |
| H | 1.665719000 | 4.938574000 | 1.562350000 |
| O | -4.340369000 | 4.604889000 | 0.106928000 |
| C | -3.425590000 | 5.718136000 | 0.050919000 |
| C | -4.228586000 | 7.024602000 | -0.021987000 |
| C | -3.207662000 | 8.178161000 | -0.076972000 |
| C | -5.099572000 | 7.039568000 | -1.292503000 |
| C | -5.112864000 | 7.172585000 | 1.230639000 |
| H | -2.792894000 | 5.703497000 | 0.947876000 |
| H | -2.786290000 | 5.609874000 | -0.834413000 |
| H | -3.729000000 | 9.140410000 | -0.130449000 |

| | | | |
|---|---|---|---|
| H | -2.559261000 | 8.098820000 | -0.958110000 |
| H | -2.569883000 | 8.192120000 | 0.815312000 |
| H | -4.483478000 | 6.944459000 | -2.195282000 |
| H | -5.654957000 | 7.981847000 | -1.362490000 |
| H | -5.823423000 | 6.218308000 | -1.286945000 |
| H | -4.505722000 | 7.173225000 | 2.144333000 |
| H | -5.837529000 | 6.355388000 | 1.304660000 |
| H | -5.668367000 | 8.116763000 | 1.195597000 |
| H | -7.171012000 | -4.610797000 | -0.016081000 |
| N | 0.477973000 | -2.267330000 | -0.137895000 |
| C | 1.363031000 | -3.306831000 | -0.074971000 |
| C | 0.643562000 | -4.483538000 | -0.084849000 |
| C | -0.707442000 | -4.097304000 | -0.131881000 |
| N | -0.815652000 | -2.766952000 | -0.151271000 |
| H | -1.599246000 | -4.707204000 | -0.138919000 |
| H | 1.040280000 | -5.486703000 | -0.051624000 |
| H | 2.423639000 | -3.143285000 | 0.005291000 |
| N | 4.243038000 | 5.576739000 | 0.579443000 |
| N | 5.536677000 | 6.076272000 | 0.593879000 |
| C | 5.428546000 | 7.406634000 | 0.574446000 |
| C | 4.077602000 | 7.792945000 | 0.526313000 |
| C | 3.358081000 | 6.616274000 | 0.515808000 |
| C | 6.824234000 | 1.633362000 | 0.148304000 |
| C | 7.223920000 | 0.294450000 | 0.237321000 |
| C | 8.582945000 | -0.043812000 | 0.255518000 |
| C | 9.531767000 | 0.990130000 | 0.196429000 |
| C | 9.083136000 | 2.294265000 | 0.165956000 |
| N | 7.775949000 | 2.637790000 | 0.158130000 |
| O | 9.060937000 | -1.295886000 | 0.334856000 |
| C | 8.146005000 | -2.409064000 | 0.389833000 |
| C | 8.948813000 | -3.715660000 | 0.462424000 |
| C | 7.927723000 | -4.869126000 | 0.516344000 |
| C | 9.833646000 | -3.863097000 | -0.789873000 |
| C | 9.819211000 | -3.731395000 | 1.733332000 |
| H | 6.320360000 | 8.016507000 | 0.582286000 |
| H | 3.680971000 | 8.796134000 | 0.492802000 |
| H | 2.297534000 | 6.452747000 | 0.434656000 |
| H | 6.475625000 | -0.475415000 | 0.334185000 |
| H | 10.591778000 | 0.765732000 | 0.201790000 |
| H | 9.788208000 | 3.116950000 | 0.155182000 |
| H | 7.513738000 | -2.393864000 | -0.507417000 |
| H | 7.506297000 | -2.301199000 | 1.274919000 |
| H | 7.278936000 | -4.790176000 | 1.397234000 |
| H | 8.448927000 | -5.831462000 | 0.569554000 |
| H | 7.290339000 | -4.882549000 | -0.376232000 |
| H | 9.226924000 | -3.863193000 | -1.703846000 |
| H | 10.389027000 | -4.807354000 | -0.755065000 |
| H | 10.558431000 | -3.045938000 | -0.863135000 |
| H | 9.202712000 | -3.636667000 | 2.635874000 |
| H | 10.543173000 | -2.910228000 | 1.728533000 |
| H | 10.374442000 | -4.673782000 | 1.803092000 |

| | | | |
|---|---|---|---|
| Os | 7.007534000 | 4.594592000 | 0.377341000 |
| C | 7.259651000 | 4.078910000 | 4.742902000 |
| C | 6.855912000 | 4.014303000 | 3.415794000 |
| N | 7.525583000 | 4.630994000 | 2.416259000 |
| C | 8.658073000 | 5.350971000 | 2.729886000 |
| C | 9.102558000 | 5.446572000 | 4.049566000 |
| C | 8.402803000 | 4.808416000 | 5.068919000 |
| C | 9.344178000 | 5.990268000 | 1.594024000 |
| N | 8.740477000 | 5.745937000 | 0.405594000 |
| C | 9.212688000 | 6.246692000 | -0.761721000 |
| C | 10.361330000 | 7.041167000 | -0.764319000 |
| C | 10.999671000 | 7.303809000 | 0.449121000 |
| C | 10.495042000 | 6.779935000 | 1.640652000 |
| C | 8.397137000 | 5.858480000 | -1.925534000 |
| N | 7.298947000 | 5.071909000 | -1.652208000 |
| C | 6.516473000 | 4.677207000 | -2.681468000 |
| C | 6.770058000 | 5.035338000 | -3.999151000 |
| C | 7.877187000 | 5.834362000 | -4.283366000 |
| C | 8.693241000 | 6.244217000 | -3.233681000 |
| H | 5.973678000 | 3.460553000 | 3.121400000 |
| H | 6.679961000 | 3.562997000 | 5.499975000 |
| H | 8.744653000 | 4.880034000 | 6.095804000 |
| H | 9.994663000 | 6.018245000 | 4.275417000 |
| H | 10.992182000 | 6.987730000 | 2.580521000 |
| H | 11.892396000 | 7.919343000 | 0.466399000 |
| H | 10.755182000 | 7.450233000 | -1.686989000 |
| H | 9.561507000 | 6.862536000 | -3.427243000 |
| H | 8.103750000 | 6.132192000 | -5.301460000 |
| H | 6.104505000 | 4.687976000 | -4.781321000 |
| H | 5.669585000 | 4.056438000 | -2.418674000 |

Cartesian coordinates of DFT-optimized structure of **11aox** complex by B3LYP/Def2-TZVP; 6-31G(d,p)/$CH_3CN$, charge = 3, multiplicity = 2

| | | | |
|---|---|---|---|
| Os | 2.853548000 | 0.446678000 | 0.038747000 |
| N | 1.726936000 | -1.329058000 | 0.237479000 |
| N | 3.319315000 | 0.532313000 | 2.088749000 |
| N | 4.806674000 | -0.253486000 | 0.105164000 |
| N | 3.270705000 | 0.016147000 | -1.976842000 |
| N | -7.789139000 | 2.401076000 | 0.035473000 |
| C | 2.493743000 | 0.943487000 | 3.077342000 |
| H | 1.504839000 | 1.254174000 | 2.765351000 |
| C | 2.874150000 | 0.972138000 | 4.412554000 |
| H | 2.166219000 | 1.313084000 | 5.159600000 |
| C | 4.160991000 | 0.560671000 | 4.759662000 |
| H | 4.489847000 | 0.569818000 | 5.793261000 |
| C | 5.021295000 | 0.135028000 | 3.752582000 |
| H | 6.025731000 | -0.190706000 | 3.995221000 |
| C | 4.592875000 | 0.124556000 | 2.423943000 |
| C | 5.441282000 | -0.312523000 | 1.301905000 |

| | | | |
|---|---|---|---|
| C | 6.765170000 | -0.752405000 | 1.366522000 |
| H | 7.290299000 | -0.805157000 | 2.312717000 |
| C | 7.408841000 | -1.124530000 | 0.184858000 |
| C | 6.736999000 | -1.056291000 | -1.037040000 |
| H | 7.240489000 | -1.344865000 | -1.951883000 |
| C | 5.413605000 | -0.610339000 | -1.053620000 |
| C | 4.537613000 | -0.465782000 | -2.229216000 |
| C | 4.933981000 | -0.790775000 | -3.527749000 |
| H | 5.933587000 | -1.169186000 | -3.705182000 |
| C | 4.047654000 | -0.631537000 | -4.588243000 |
| H | 4.352013000 | -0.882443000 | -5.598669000 |
| C | 2.766554000 | -0.147060000 | -4.324365000 |
| H | 2.038349000 | -0.007088000 | -5.115317000 |
| C | 2.418286000 | 0.162222000 | -3.016137000 |
| H | 1.434352000 | 0.539820000 | -2.768818000 |
| C | 2.228583000 | -2.583419000 | 0.277958000 |
| H | 3.307603000 | -2.658578000 | 0.343256000 |
| C | 1.449775000 | -3.721118000 | 0.229863000 |
| H | 1.899830000 | -4.706143000 | 0.264891000 |
| C | 0.058954000 | -3.576804000 | 0.097624000 |
| C | -0.478707000 | -2.284196000 | 0.064371000 |
| H | -1.536565000 | -2.142163000 | -0.088133000 |
| C | 0.354230000 | -1.163832000 | 0.175585000 |
| C | -4.463327000 | 4.577171000 | -0.337309000 |
| C | -0.085082000 | 0.244678000 | 0.134772000 |
| C | -5.570813000 | 3.716961000 | -0.166630000 |
| C | 1.010263000 | 1.131958000 | -0.020650000 |
| C | -5.380818000 | 2.324585000 | 0.020460000 |
| C | 0.787945000 | 2.512999000 | -0.216847000 |
| C | -6.661257000 | 1.588059000 | 0.024943000 |
| C | -6.813260000 | 0.208808000 | -0.073348000 |
| H | -5.956667000 | -0.440557000 | -0.181359000 |
| C | -8.087308000 | -0.382416000 | -0.080710000 |
| C | -9.211201000 | 0.450074000 | -0.000761000 |
| H | -10.223479000 | 0.068626000 | 0.006907000 |
| C | -9.002294000 | 1.821525000 | 0.037226000 |
| H | -9.849491000 | 2.497078000 | 0.059838000 |
| C | -4.070542000 | 1.821077000 | 0.187028000 |
| C | -0.499256000 | 3.031482000 | -0.398598000 |
| C | -2.946809000 | 2.681849000 | -0.123542000 |
| C | -1.619974000 | 2.160023000 | -0.094190000 |
| C | -3.161567000 | 4.077853000 | -0.460988000 |
| C | -1.394403000 | 0.769893000 | 0.243935000 |
| C | -2.043830000 | 4.839130000 | -0.938878000 |
| C | -2.511497000 | 0.027433000 | 0.748203000 |
| H | -2.210938000 | 5.804390000 | -1.398967000 |
| H | -2.339341000 | -0.927258000 | 1.228497000 |
| C | -0.780118000 | 4.342286000 | -0.908856000 |
| C | -3.778242000 | 0.523914000 | 0.719986000 |
| H | 0.018223000 | 4.929288000 | -1.344441000 |
| H | -4.572993000 | -0.051804000 | 1.176539000 |

| | | | |
|---|---|---|---|
| O | -0.656322000 | -4.709584000 | 0.003394000 |
| O | -8.115955000 | -1.720667000 | -0.174428000 |
| C | -9.387707000 | -2.402075000 | -0.202361000 |
| C | -9.145526000 | -3.911943000 | -0.337883000 |
| C | -10.530249000 | -4.588698000 | -0.362356000 |
| C | -8.335460000 | -4.428933000 | 0.866026000 |
| C | -8.396746000 | -4.212755000 | -1.650177000 |
| C | -2.089284000 | -4.641398000 | -0.137494000 |
| C | -2.656615000 | -6.066901000 | -0.194084000 |
| C | -4.184770000 | -5.937969000 | -0.349227000 |
| C | -2.073304000 | -6.821760000 | -1.403646000 |
| C | -2.328089000 | -6.818583000 | 1.109759000 |
| H | -9.972799000 | -2.026594000 | -1.051286000 |
| H | -9.929215000 | -2.179554000 | 0.725729000 |
| H | -11.132088000 | -4.241444000 | -1.211015000 |
| H | -11.091534000 | -4.387152000 | 0.558153000 |
| H | -10.417525000 | -5.674735000 | -0.454457000 |
| H | -8.859461000 | -4.232004000 | 1.809458000 |
| H | -7.351716000 | -3.950978000 | 0.917277000 |
| H | -8.183461000 | -5.511390000 | 0.785609000 |
| H | -8.962309000 | -3.854891000 | -2.519410000 |
| H | -8.251032000 | -5.292847000 | -1.765883000 |
| H | -7.412254000 | -3.733973000 | -1.664955000 |
| H | -2.505275000 | -4.094523000 | 0.718404000 |
| H | -2.331235000 | -4.094240000 | -1.057554000 |
| H | -4.644575000 | -6.931672000 | -0.391277000 |
| H | -4.450830000 | -5.406659000 | -1.271176000 |
| H | -4.630045000 | -5.398362000 | 0.495614000 |
| H | -2.302266000 | -6.301919000 | -2.342213000 |
| H | -2.499961000 | -7.829506000 | -1.465038000 |
| H | -0.985687000 | -6.918345000 | -1.325108000 |
| H | -2.748497000 | -6.300672000 | 1.980777000 |
| H | -1.246702000 | -6.906477000 | 1.255528000 |
| H | -2.750977000 | -7.829387000 | 1.083406000 |
| H | 8.437068000 | -1.467935000 | 0.216208000 |
| N | 2.006622000 | 3.248982000 | -0.245199000 |
| C | 2.315756000 | 4.578083000 | -0.185741000 |
| C | 3.689894000 | 4.698076000 | -0.141411000 |
| C | 4.181772000 | 3.381967000 | -0.152567000 |
| N | 3.175916000 | 2.504569000 | -0.201245000 |
| N | -4.854499000 | 5.945056000 | -0.401402000 |
| C | -4.179150000 | 7.130593000 | -0.343359000 |
| C | -5.105427000 | 8.153562000 | -0.347184000 |
| C | -6.360159000 | 7.522786000 | -0.385358000 |
| N | -6.217990000 | 6.195060000 | -0.404563000 |
| H | 5.200850000 | 3.025347000 | -0.115588000 |
| H | 4.259642000 | 5.613636000 | -0.096079000 |
| H | 1.552953000 | 5.335942000 | -0.144259000 |
| H | -3.106245000 | 7.169092000 | -0.270889000 |
| H | -4.901643000 | 9.212935000 | -0.316083000 |
| H | -7.349939000 | 7.955787000 | -0.388157000 |

| | | | |
|---|---|---|---|
| Os | -7.386524000 | 4.467233000 | -0.174086000 |
| C | -7.183360000 | 4.893401000 | 4.203238000 |
| C | -6.885710000 | 4.603349000 | 2.878075000 |
| N | -7.739932000 | 4.863639000 | 1.863370000 |
| C | -8.956105000 | 5.439381000 | 2.157902000 |
| C | -9.301253000 | 5.747972000 | 3.474408000 |
| C | -8.412584000 | 5.476109000 | 4.509985000 |
| C | -9.838861000 | 5.694102000 | 1.006390000 |
| C | -11.105980000 | 6.280911000 | 1.029778000 |
| C | -11.793289000 | 6.443076000 | -0.174262000 |
| C | -11.220839000 | 6.023996000 | -1.376376000 |
| C | -9.951791000 | 5.441667000 | -1.349372000 |
| N | -9.301105000 | 5.294533000 | -0.170721000 |
| C | -9.181098000 | 4.936187000 | -2.498861000 |
| N | -7.937887000 | 4.421017000 | -2.204585000 |
| C | -7.184777000 | 3.932189000 | -3.214923000 |
| C | -7.611043000 | 3.932356000 | -4.536739000 |
| C | -8.866892000 | 4.454962000 | -4.843713000 |
| C | -9.653389000 | 4.957919000 | -3.811867000 |
| H | -6.456377000 | 4.661938000 | 4.973449000 |
| H | -5.943611000 | 4.150650000 | 2.597254000 |
| H | -10.263282000 | 6.199161000 | 3.685383000 |
| H | -8.676704000 | 5.714272000 | 5.534667000 |
| H | -11.553101000 | 6.607441000 | 1.960990000 |
| H | -12.777975000 | 6.897442000 | -0.175756000 |
| H | -11.757349000 | 6.151739000 | -2.308783000 |
| H | -6.217016000 | 3.536580000 | -2.934811000 |
| H | -6.961131000 | 3.527311000 | -5.304068000 |
| H | -9.229674000 | 4.470495000 | -5.865763000 |
| H | -10.634334000 | 5.366202000 | -4.023059000 |

Cartesian coordinates of DFT-optimized structure of **7box** complex by B3LYP/Def2-TZVP; 6-31G(d,p)/$CH_3CN$, charge = 3, multiplicity = 2

| | | | |
|---|---|---|---|
| Ru | -5.468994000 | -0.050317000 | 0.032798000 |
| Ru | 5.481207000 | 0.097058000 | 0.020057000 |
| N | -5.060009000 | -2.103965000 | 0.306338000 |
| N | 5.049038000 | 2.141015000 | -0.258108000 |
| N | -5.914155000 | 0.250776000 | 2.066988000 |
| N | 5.973222000 | -0.196041000 | -2.000480000 |
| N | -7.561349000 | -0.072076000 | 0.080284000 |
| N | 7.542736000 | 0.130500000 | 0.051233000 |
| N | -5.997816000 | -0.364460000 | -1.979480000 |
| N | 5.902622000 | 0.406597000 | 2.055778000 |
| N | -5.117729000 | 2.011372000 | -0.257422000 |
| N | 5.111690000 | -1.961432000 | 0.286935000 |
| C | -5.003819000 | 0.413117000 | 3.047007000 |
| C | 5.107107000 | -0.359363000 | -3.020039000 |
| H | 4.057285000 | -0.332609000 | -2.754801000 |
| C | -5.360412000 | 0.617140000 | 4.376043000 |

| | | | |
|---|---|---|---|
| C | 5.516525000 | -0.550327000 | -4.335375000 |
| H | 4.772007000 | -0.676174000 | -5.113540000 |
| C | -6.711627000 | 0.656118000 | 4.712969000 |
| C | 6.881196000 | -0.574944000 | -4.619576000 |
| C | -7.660212000 | 0.488326000 | 3.707481000 |
| C | 7.786745000 | -0.407199000 | -3.575694000 |
| H | -8.716920000 | 0.514795000 | 3.944620000 |
| C | -7.247699000 | 0.285887000 | 2.390138000 |
| C | 7.320554000 | -0.218940000 | -2.272860000 |
| C | -8.184971000 | 0.097316000 | 1.263948000 |
| C | 8.213790000 | -0.032235000 | -1.111051000 |
| C | -9.581046000 | 0.078913000 | 1.325138000 |
| C | 9.611185000 | -0.009225000 | -1.117566000 |
| H | -10.104830000 | 0.211010000 | 2.264144000 |
| H | 10.170620000 | -0.137417000 | -2.036613000 |
| C | -10.297712000 | -0.116154000 | 0.143142000 |
| C | 10.283340000 | 0.184190000 | 0.091025000 |
| C | -9.630553000 | -0.289908000 | -1.070602000 |
| C | 9.569579000 | 0.351470000 | 1.279420000 |
| C | -8.233206000 | -0.264285000 | -1.073127000 |
| C | 8.172996000 | 0.320654000 | 1.231965000 |
| C | -7.343705000 | -0.429509000 | -2.240979000 |
| C | 7.239942000 | 0.476491000 | 2.366667000 |
| C | -7.812173000 | -0.639145000 | -3.538300000 |
| C | 7.661798000 | 0.681813000 | 3.681845000 |
| H | -8.877990000 | -0.688459000 | -3.726062000 |
| C | -6.907622000 | -0.784615000 | -4.586851000 |
| C | 6.721674000 | 0.819137000 | 4.699248000 |
| C | -5.543497000 | -0.715622000 | -4.312401000 |
| C | 5.367469000 | 0.746364000 | 4.376219000 |
| H | 4.597002000 | 0.846775000 | 5.132563000 |
| C | -5.130349000 | -0.504949000 | -3.000792000 |
| C | 5.002732000 | 0.539945000 | 3.049978000 |
| H | 3.962599000 | 0.475962000 | 2.754781000 |
| C | -5.962325000 | -3.103469000 | 0.339677000 |
| C | 5.947499000 | 3.146064000 | -0.279861000 |
| H | -7.004007000 | -2.805070000 | 0.367597000 |
| H | 6.990570000 | 2.850669000 | -0.285829000 |
| C | -5.620526000 | -4.442309000 | 0.329769000 |
| C | 5.602181000 | 4.483638000 | -0.282832000 |
| H | -6.381501000 | -5.213035000 | 0.360595000 |
| H | 6.361277000 | 5.256801000 | -0.301937000 |
| C | -4.261966000 | -4.785053000 | 0.244173000 |
| C | 4.240837000 | 4.822543000 | -0.223788000 |
| C | -3.312152000 | -3.753697000 | 0.210500000 |
| C | 3.295164000 | 3.790009000 | -0.204253000 |
| H | -2.265560000 | -3.984234000 | 0.090948000 |
| H | 2.246028000 | 4.018798000 | -0.104654000 |
| C | -3.720350000 | -2.417306000 | 0.282170000 |
| C | 3.706890000 | 2.451358000 | -0.263257000 |
| C | -2.828023000 | -1.239995000 | 0.230726000 |

| | | | |
|---|---|---|---|
| C | 2.819484000 | 1.272946000 | -0.227358000 |
| C | -3.531132000 | -0.028871000 | 0.006330000 |
| C | 3.532115000 | 0.066989000 | -0.003942000 |
| C | -2.860343000 | 1.198425000 | -0.227312000 |
| C | 2.853343000 | -1.158997000 | 0.216114000 |
| C | -3.778369000 | 2.356345000 | -0.266322000 |
| C | 3.772721000 | -2.312037000 | 0.267888000 |
| C | -3.412517000 | 3.697787000 | -0.215491000 |
| C | 3.412954000 | -3.656853000 | 0.202815000 |
| H | -2.377418000 | 3.994690000 | -0.127630000 |
| H | 2.380381000 | -3.955678000 | 0.091497000 |
| C | -4.386904000 | 4.711337000 | -0.234172000 |
| C | 4.387592000 | -4.666577000 | 0.235437000 |
| C | -5.736618000 | 4.341677000 | -0.287024000 |
| C | 5.735370000 | -4.293110000 | 0.317319000 |
| H | -6.541660000 | 5.063666000 | -0.308894000 |
| H | 6.541968000 | -5.013195000 | 0.350474000 |
| C | -6.038083000 | 2.985518000 | -0.279534000 |
| C | 6.032920000 | -2.936322000 | 0.323332000 |
| H | -7.073506000 | 2.664144000 | -0.282696000 |
| H | 7.067332000 | -2.612286000 | 0.348934000 |
| C | -1.457360000 | 1.190446000 | -0.393334000 |
| C | 1.448723000 | -1.156614000 | 0.372458000 |
| C | -0.719393000 | 0.005871000 | -0.007320000 |
| C | 0.708673000 | 0.026080000 | -0.008886000 |
| C | -1.424009000 | -1.198266000 | 0.382812000 |
| C | 1.415072000 | 1.229509000 | -0.390292000 |
| C | -0.651211000 | -2.258887000 | 0.960084000 |
| C | 0.644195000 | 2.287180000 | -0.975941000 |
| H | -1.158740000 | -3.066941000 | 1.471880000 |
| H | 1.154401000 | 3.094103000 | -1.487133000 |
| C | 0.709531000 | -2.238342000 | 0.954486000 |
| C | -0.716849000 | 2.270246000 | -0.976008000 |
| H | 1.245186000 | -3.033172000 | 1.458187000 |
| H | -1.249878000 | 3.063138000 | -1.485257000 |
| O | -3.973408000 | -6.094291000 | 0.193033000 |
| O | 3.934119000 | -5.930854000 | 0.175104000 |
| O | 3.948777000 | 6.134229000 | -0.181745000 |
| O | -3.928577000 | 5.971456000 | -0.191012000 |
| C | -4.871171000 | 7.064800000 | -0.189666000 |
| C | -4.104109000 | 8.392986000 | -0.131063000 |
| C | -5.156850000 | 9.518929000 | -0.125376000 |
| C | -3.197888000 | 8.535412000 | -1.368592000 |
| C | -3.262078000 | 8.463449000 | 1.156949000 |
| C | 2.573780000 | 6.557503000 | -0.105302000 |
| C | 2.518476000 | 8.091996000 | -0.120409000 |
| C | 1.033450000 | 8.490925000 | -0.015584000 |
| C | 3.295889000 | 8.662726000 | 1.080788000 |
| C | 3.109562000 | 8.627602000 | -1.438332000 |
| C | -2.599696000 | -6.523317000 | 0.094867000 |
| C | -2.550782000 | -8.057832000 | 0.107086000 |

| | | | |
|---|---|---|---|
| C | -3.125056000 | -8.593090000 | 1.432548000 |
| C | -3.346933000 | -8.623810000 | -1.084029000 |
| C | -1.068599000 | -8.461434000 | -0.019443000 |
| C | 4.880923000 | -7.018287000 | 0.183508000 |
| C | 4.121977000 | -8.349940000 | 0.097797000 |
| C | 3.305483000 | -8.412308000 | -1.206979000 |
| C | 3.191868000 | -8.507735000 | 1.315602000 |
| C | 5.179256000 | -9.471505000 | 0.102618000 |
| H | -5.531730000 | 6.960572000 | 0.680182000 |
| H | -5.477400000 | 7.010390000 | -1.102544000 |
| H | -5.819162000 | 9.443282000 | 0.745455000 |
| H | -5.777363000 | 9.492447000 | -1.029256000 |
| H | -4.663091000 | 10.496289000 | -0.086768000 |
| H | -3.785168000 | 8.498395000 | -2.294527000 |
| H | -2.449916000 | 7.736678000 | -1.408910000 |
| H | -2.668593000 | 9.494881000 | -1.344650000 |
| H | -3.895813000 | 8.377992000 | 2.048372000 |
| H | -2.731666000 | 9.420956000 | 1.212680000 |
| H | -2.517824000 | 7.661114000 | 1.191302000 |
| H | 2.024844000 | 6.145467000 | -0.961746000 |
| H | 2.131216000 | 6.164439000 | 0.819078000 |
| H | 0.933984000 | 9.582144000 | -0.026769000 |
| H | 0.583810000 | 8.123451000 | 0.914998000 |
| H | 0.450449000 | 8.093798000 | -0.855510000 |
| H | 2.881691000 | 8.298203000 | 2.028967000 |
| H | 3.236864000 | 9.757205000 | 1.086863000 |
| H | 4.352616000 | 8.379812000 | 1.040456000 |
| H | 2.557154000 | 8.242316000 | -2.304445000 |
| H | 4.159519000 | 8.337867000 | -1.548754000 |
| H | 3.054571000 | 9.721903000 | -1.464401000 |
| H | -2.037681000 | -6.114288000 | 0.943921000 |
| H | -2.171148000 | -6.130291000 | -0.835796000 |
| H | -2.559750000 | -8.210296000 | 2.291367000 |
| H | -3.072569000 | -9.687516000 | 1.456503000 |
| H | -4.172734000 | -8.301074000 | 1.557859000 |
| H | -4.402058000 | -8.337321000 | -1.028926000 |
| H | -3.292002000 | -9.718454000 | -1.092072000 |
| H | -2.944282000 | -8.259713000 | -2.037301000 |
| H | -0.472528000 | -8.067884000 | 0.812922000 |
| H | -0.631029000 | -8.093808000 | -0.955671000 |
| H | -0.973147000 | -9.552996000 | -0.011621000 |
| H | 5.469239000 | -6.970534000 | 1.108703000 |
| H | 5.559413000 | -6.905057000 | -0.671564000 |
| H | 2.780158000 | -9.371368000 | -1.281927000 |
| H | 3.956096000 | -8.316060000 | -2.085069000 |
| H | 2.558999000 | -7.612437000 | -1.248377000 |
| H | 2.439666000 | -7.712618000 | 1.347818000 |
| H | 3.760320000 | -8.476142000 | 2.253441000 |
| H | 2.667659000 | -9.469381000 | 1.272816000 |
| H | 5.858537000 | -9.384907000 | -0.754050000 |
| H | 4.690875000 | -10.450688000 | 0.045204000 |

| | | | |
|---|---|---|---|
| H | 5.781720000 | -9.450716000 | 1.018792000 |
| H | -4.584180000 | 0.741547000 | 5.122471000 |
| H | -7.025597000 | 0.813560000 | 5.739291000 |
| H | -3.965363000 | 0.374996000 | 2.743060000 |
| H | -10.191436000 | -0.440742000 | -1.985036000 |
| H | -11.381877000 | -0.133236000 | 0.168265000 |
| H | -7.264928000 | -0.947851000 | -5.597972000 |
| H | -4.800109000 | -0.821651000 | -5.094279000 |
| H | -4.080139000 | -0.443364000 | -2.744898000 |
| H | 7.236867000 | -0.721342000 | -5.634001000 |
| H | 8.852454000 | -0.422003000 | -3.770988000 |
| H | 11.367710000 | 0.204885000 | 0.106692000 |
| H | 10.096205000 | 0.501221000 | 2.214528000 |
| H | 8.720352000 | 0.734284000 | 3.907116000 |
| H | 7.042985000 | 0.979113000 | 5.723063000 |

Cartesian coordinates of DFT-optimized structure of **8box** complex by B3LYP/Def2-TZVP; 6-31G(d,p)/$CH_3CN$, charge = 3, multiplicity = 2

| | | | |
|---|---|---|---|
| Ru | 4.933161000 | -0.356676000 | -0.207831000 |
| N | 5.455098000 | 0.154196000 | 1.767323000 |
| N | 7.004172000 | -0.456924000 | -0.238097000 |
| N | 5.345524000 | -0.912751000 | -2.196645000 |
| C | 4.598454000 | 0.459028000 | 2.760449000 |
| H | 3.546418000 | 0.431623000 | 2.505307000 |
| C | 5.021639000 | 0.790699000 | 4.043648000 |
| H | 4.285523000 | 1.027852000 | 4.803377000 |
| C | 6.387284000 | 0.808751000 | 4.318352000 |
| H | 6.753441000 | 1.063068000 | 5.307309000 |
| C | 7.281995000 | 0.492916000 | 3.299034000 |
| H | 8.348603000 | 0.499053000 | 3.488969000 |
| C | 6.802415000 | 0.167975000 | 2.029460000 |
| C | 7.682322000 | -0.179237000 | 0.893914000 |
| C | 9.078591000 | -0.239325000 | 0.901490000 |
| H | 9.645395000 | -0.021506000 | 1.798573000 |
| C | 9.737783000 | -0.589296000 | -0.278028000 |
| C | 9.013775000 | -0.872286000 | -1.437340000 |
| H | 9.530501000 | -1.142581000 | -2.350249000 |
| C | 7.619048000 | -0.797219000 | -1.388997000 |
| C | 6.676997000 | -1.056201000 | -2.497930000 |
| C | 7.085743000 | -1.424099000 | -3.780499000 |
| H | 8.140781000 | -1.532929000 | -4.001446000 |
| C | 6.135380000 | -1.650582000 | -4.772861000 |
| H | 6.446279000 | -1.937638000 | -5.771723000 |
| C | 4.786453000 | -1.501131000 | -4.458486000 |
| H | 4.009043000 | -1.664989000 | -5.196105000 |
| C | 4.434861000 | -1.131997000 | -3.163953000 |
| H | 3.398276000 | -1.003484000 | -2.878294000 |
| C | -3.308983000 | 1.213845000 | -0.417853000 |
| C | 2.234282000 | -1.397335000 | 0.163484000 |

| | | | |
|---|---|---|---|
| C | -4.065537000 | 0.079221000 | -0.074886000 |
| C | 2.990819000 | -0.262663000 | -0.179368000 |
| C | -3.412413000 | -1.123748000 | 0.247087000 |
| C | 2.337673000 | 0.940264000 | -0.501457000 |
| C | -2.015589000 | -1.193349000 | 0.360143000 |
| C | 0.940858000 | 1.009785000 | -0.614679000 |
| C | -1.249128000 | -0.057307000 | -0.116748000 |
| C | 0.174401000 | -0.126277000 | -0.137823000 |
| C | -1.915944000 | 1.148197000 | -0.572383000 |
| C | 0.841222000 | -1.331763000 | 0.317849000 |
| C | -1.123886000 | 2.166365000 | -1.196883000 |
| C | 0.049156000 | -2.349976000 | 0.942269000 |
| H | -1.611359000 | 2.973664000 | -1.727221000 |
| H | 0.536621000 | -3.157310000 | 1.472561000 |
| C | 0.232820000 | 2.100633000 | -1.216969000 |
| C | -1.307551000 | -2.284243000 | 0.962354000 |
| H | 0.780163000 | 2.857796000 | -1.762625000 |
| H | -1.854892000 | -3.041441000 | 1.507963000 |
| H | 10.821044000 | -0.641755000 | -0.293852000 |
| N | -4.338747000 | -2.180965000 | 0.467242000 |
| N | -5.677740000 | -1.829793000 | 0.458424000 |
| C | -6.365350000 | -2.962957000 | 0.595805000 |
| C | -5.495536000 | -4.065773000 | 0.675919000 |
| C | -4.224988000 | -3.537914000 | 0.576688000 |
| H | -7.445321000 | -2.935326000 | 0.618469000 |
| H | -5.759118000 | -5.107067000 | 0.781447000 |
| H | -3.269843000 | -4.032654000 | 0.543901000 |
| Ru | -6.007865000 | 0.173426000 | -0.045776000 |
| C | -5.859251000 | 1.317933000 | 4.204804000 |
| C | -5.508238000 | 0.948743000 | 2.910130000 |
| N | -6.419327000 | 0.729533000 | 1.943215000 |
| C | -7.750661000 | 0.873078000 | 2.245066000 |
| C | -8.158835000 | 1.241038000 | 3.527799000 |
| C | -7.208035000 | 1.467482000 | 4.519752000 |
| C | -8.693205000 | 0.614141000 | 1.136545000 |
| N | -8.078845000 | 0.273844000 | -0.014631000 |
| C | -8.757502000 | -0.003800000 | -1.146350000 |
| C | -10.153773000 | 0.056324000 | -1.153305000 |
| C | -10.812436000 | 0.406291000 | 0.026509000 |
| C | -10.087909000 | 0.689242000 | 1.185507000 |
| C | -7.878102000 | -0.351014000 | -2.282290000 |
| N | -6.530672000 | -0.337340000 | -2.020725000 |
| C | -5.674472000 | -0.642174000 | -3.014234000 |
| C | -6.098226000 | -0.973742000 | -4.297272000 |
| C | -7.463988000 | -0.991684000 | -4.571396000 |
| C | -8.358245000 | -0.675846000 | -3.551682000 |
| H | -4.471784000 | 0.820163000 | 2.624024000 |
| H | -5.081516000 | 1.481759000 | 4.942086000 |
| H | -7.518490000 | 1.754587000 | 5.518738000 |
| H | -9.213771000 | 1.349952000 | 3.749186000 |
| H | -10.604227000 | 0.959526000 | 2.098652000 |

| | | | |
|---|---|---|---|
| H | -11.895688000 | 0.458773000 | 0.042813000 |
| H | -10.720978000 | -0.161473000 | -2.050139000 |
| H | -9.424934000 | -0.681891000 | -3.741170000 |
| H | -7.830581000 | -1.245915000 | -5.560215000 |
| H | -5.362450000 | -1.210899000 | -5.057329000 |
| H | -4.622325000 | -0.614846000 | -2.759546000 |
| N | -4.134853000 | 2.356023000 | -0.611873000 |
| N | -5.500677000 | 2.136018000 | -0.562555000 |
| C | -6.079242000 | 3.330565000 | -0.681114000 |
| C | -5.109611000 | 4.344174000 | -0.788858000 |
| C | -3.893690000 | 3.695751000 | -0.726767000 |
| H | -2.894621000 | 4.095742000 | -0.723268000 |
| H | -5.274261000 | 5.406223000 | -0.888088000 |
| H | -7.157015000 | 3.407566000 | -0.671492000 |
| N | 3.060196000 | -2.539448000 | 0.357686000 |
| C | 2.819105000 | -3.879201000 | 0.472433000 |
| C | 4.035062000 | -4.527529000 | 0.534832000 |
| C | 5.004642000 | -3.513834000 | 0.427429000 |
| N | 4.426013000 | -2.319325000 | 0.308779000 |
| N | 3.263975000 | 1.997549000 | -0.721426000 |
| N | 4.602997000 | 1.646494000 | -0.712195000 |
| C | 5.290551000 | 2.779703000 | -0.849479000 |
| C | 4.420667000 | 3.882438000 | -0.929934000 |
| C | 3.150137000 | 3.354481000 | -0.831017000 |
| H | 1.820066000 | -4.279264000 | 0.468618000 |
| H | 4.199771000 | -5.589570000 | 0.634043000 |
| H | 6.082424000 | -3.590746000 | 0.418115000 |
| H | 2.194945000 | 3.849152000 | -0.798545000 |
| H | 4.684191000 | 4.923745000 | -1.035478000 |
| H | 6.370531000 | 2.752160000 | -0.871831000 |

Cartesian coordinates of DFT-optimized structure of **9box** complex by B3LYP/Def2-TZVP; 6-31G(d,p)/$CH_3CN$, charge = 3, multiplicity = 2

| | | | |
|---|---|---|---|
| Ru | 4.930937000 | -0.193225000 | 0.004791000 |
| N | 4.479142000 | -2.232709000 | 0.314151000 |
| N | 5.443253000 | 0.111303000 | 2.023320000 |
| N | 7.030764000 | -0.262614000 | -0.001237000 |
| N | 5.408923000 | -0.547795000 | -2.013707000 |
| C | 4.559642000 | 0.298238000 | 3.022954000 |
| H | 3.513627000 | 0.283601000 | 2.744293000 |
| C | 4.953238000 | 0.499482000 | 4.341943000 |
| H | 4.198314000 | 0.644799000 | 5.106136000 |
| C | 6.312598000 | 0.508023000 | 4.645977000 |
| H | 6.654614000 | 0.661358000 | 5.663868000 |
| C | 7.233019000 | 0.315626000 | 3.618771000 |
| H | 8.295421000 | 0.319026000 | 3.830636000 |
| C | 6.784286000 | 0.118484000 | 2.312732000 |
| C | 7.688020000 | -0.093850000 | 1.163522000 |
| C | 9.084789000 | -0.132214000 | 1.188064000 |

| | | | |
|---|---|---|---|
| H | 9.634802000 | 0.000748000 | 2.111658000 |
| C | 9.766356000 | -0.347213000 | -0.010978000 |
| C | 9.064413000 | -0.520229000 | -1.204946000 |
| H | 9.598215000 | -0.688101000 | -2.132415000 |
| C | 7.668068000 | -0.472408000 | -1.170230000 |
| C | 6.745074000 | -0.634663000 | -2.312011000 |
| C | 7.172721000 | -0.865758000 | -3.619602000 |
| H | 8.231755000 | -0.930992000 | -3.838010000 |
| C | 6.235786000 | -1.012338000 | -4.639348000 |
| H | 6.561299000 | -1.191676000 | -5.658406000 |
| C | 4.881293000 | -0.925083000 | -4.325999000 |
| H | 4.113849000 | -1.033258000 | -5.083874000 |
| C | 4.509154000 | -0.692736000 | -3.005898000 |
| H | 3.467771000 | -0.617231000 | -2.720057000 |
| C | 5.359438000 | -3.251147000 | 0.353320000 |
| H | 6.407662000 | -2.975523000 | 0.367069000 |
| C | 4.986722000 | -4.581426000 | 0.367773000 |
| H | 5.729846000 | -5.369150000 | 0.401752000 |
| C | 3.619596000 | -4.893714000 | 0.302783000 |
| C | 2.692142000 | -3.841575000 | 0.267653000 |
| H | 1.638651000 | -4.049573000 | 0.167993000 |
| C | 3.132412000 | -2.514583000 | 0.312142000 |
| C | -3.317521000 | 1.265241000 | -0.316524000 |
| C | 2.267738000 | -1.316980000 | 0.253138000 |
| C | -4.064870000 | 0.123500000 | 0.003654000 |
| C | 2.992192000 | -0.125838000 | 0.003777000 |
| C | -3.399723000 | -1.068010000 | 0.323967000 |
| C | 2.353170000 | 1.112323000 | -0.246569000 |
| C | -1.998995000 | -1.125453000 | 0.451162000 |
| C | 0.948881000 | 1.132551000 | -0.411515000 |
| C | -1.241600000 | 0.023461000 | 0.003862000 |
| C | 0.187149000 | -0.027492000 | 0.003384000 |
| C | -1.916006000 | 1.223418000 | -0.442891000 |
| C | 0.865314000 | -1.238319000 | 0.418742000 |
| C | -1.124084000 | 2.257928000 | -1.035687000 |
| C | 0.077365000 | -2.267698000 | 1.028059000 |
| H | -1.609609000 | 3.065932000 | -1.567011000 |
| H | 0.573085000 | -3.078368000 | 1.546668000 |
| C | 0.236115000 | 2.217357000 | -1.016655000 |
| C | -1.282382000 | -2.211956000 | 1.046845000 |
| H | 0.788769000 | 2.993724000 | -1.529846000 |
| H | -1.823973000 | -2.980916000 | 1.582176000 |
| O | 3.301416000 | -6.195887000 | 0.271602000 |
| C | 1.917487000 | -6.596103000 | 0.192336000 |
| C | 1.838446000 | -8.129306000 | 0.194250000 |
| C | 0.346186000 | -8.503193000 | 0.099422000 |
| C | 2.595630000 | -8.700789000 | -1.019536000 |
| C | 2.432219000 | -8.687153000 | 1.501694000 |
| H | 1.377676000 | -6.180750000 | 1.052757000 |
| H | 1.482562000 | -6.188444000 | -0.728893000 |
| H | 0.230422000 | -9.592729000 | 0.101339000 |

| | | | |
|---|---|---|---|
| H | -0.105415000 | -8.120006000 | -0.823699000 |
| H | -0.222225000 | -8.104327000 | 0.948385000 |
| H | 2.183935000 | -8.314713000 | -1.960270000 |
| H | 2.511847000 | -9.793443000 | -1.039518000 |
| H | 3.658906000 | -8.442979000 | -0.982589000 |
| H | 1.894452000 | -8.300364000 | 2.376244000 |
| H | 3.488234000 | -8.417719000 | 1.605034000 |
| H | 2.357230000 | -9.780460000 | 1.517677000 |
| H | 10.850411000 | -0.380572000 | -0.014806000 |
| N | -4.316262000 | -2.139331000 | 0.525992000 |
| N | -5.660481000 | -1.804730000 | 0.500519000 |
| C | -6.334569000 | -2.947656000 | 0.628089000 |
| C | -5.452042000 | -4.039556000 | 0.717359000 |
| C | -4.186964000 | -3.495067000 | 0.635857000 |
| H | -7.414960000 | -2.933259000 | 0.635672000 |
| H | -5.703257000 | -5.084613000 | 0.816857000 |
| H | -3.225991000 | -3.978715000 | 0.614688000 |
| Ru | -6.026190000 | 0.191339000 | 0.000549000 |
| C | -5.949459000 | 1.343432000 | 4.256522000 |
| C | -5.570133000 | 0.976237000 | 2.969382000 |
| N | -6.457481000 | 0.740446000 | 1.984034000 |
| C | -7.797352000 | 0.868003000 | 2.262129000 |
| C | -8.234393000 | 1.234509000 | 3.536564000 |
| C | -7.306396000 | 1.475422000 | 4.546186000 |
| C | -8.717646000 | 0.594970000 | 1.138566000 |
| N | -8.077689000 | 0.262019000 | -0.003491000 |
| C | -8.735271000 | -0.025940000 | -1.147644000 |
| C | -10.132010000 | 0.015187000 | -1.176627000 |
| C | -10.815715000 | 0.356455000 | -0.008086000 |
| C | -10.113981000 | 0.650198000 | 1.162716000 |
| C | -7.831868000 | -0.359975000 | -2.268309000 |
| N | -6.487527000 | -0.325619000 | -1.985021000 |
| C | -5.614859000 | -0.617748000 | -2.968405000 |
| C | -6.013762000 | -0.953621000 | -4.258167000 |
| C | -7.375470000 | -0.991763000 | -4.552862000 |
| C | -8.288422000 | -0.691338000 | -3.545501000 |
| H | -4.526731000 | 0.863439000 | 2.700844000 |
| H | -5.188202000 | 1.520373000 | 5.008118000 |
| H | -7.639661000 | 1.760536000 | 5.538529000 |
| H | -9.294826000 | 1.330966000 | 3.737310000 |
| H | -10.649794000 | 0.913621000 | 2.066828000 |
| H | -11.899791000 | 0.393630000 | -0.009805000 |
| H | -10.681773000 | -0.211173000 | -2.082455000 |
| H | -9.352197000 | -0.713916000 | -3.750073000 |
| H | -7.723802000 | -1.249766000 | -5.547443000 |
| H | -5.263650000 | -1.178992000 | -5.008000000 |
| H | -4.567112000 | -0.576604000 | -2.696365000 |
| N | -4.157123000 | 2.397844000 | -0.520774000 |
| N | -5.521521000 | 2.157313000 | -0.497764000 |
| C | -6.114340000 | 3.344107000 | -0.629094000 |
| C | -5.157886000 | 4.371846000 | -0.718580000 |

| | | | |
|---|---|---|---|
| C | -3.933806000 | 3.741045000 | -0.633163000 |
| C | 3.296426000 | 2.249155000 | -0.305010000 |
| N | 4.628331000 | 1.876469000 | -0.304455000 |
| C | 5.569190000 | 2.830202000 | -0.341723000 |
| C | 5.296082000 | 4.192567000 | -0.357125000 |
| C | 3.954792000 | 4.590113000 | -0.294253000 |
| C | 2.959065000 | 3.597232000 | -0.261397000 |
| O | 3.521758000 | 5.858110000 | -0.253765000 |
| C | 4.481767000 | 6.936359000 | -0.266978000 |
| C | 3.734719000 | 8.274937000 | -0.187447000 |
| C | 4.802533000 | 9.386509000 | -0.204978000 |
| C | 2.924710000 | 8.352675000 | 1.120592000 |
| C | 2.799917000 | 8.432757000 | -1.401598000 |
| H | 5.068847000 | 6.875654000 | -1.191930000 |
| H | 5.157554000 | 6.818641000 | 0.589295000 |
| H | 5.400673000 | 9.353793000 | -1.123752000 |
| H | 5.485285000 | 9.301105000 | 0.649171000 |
| H | 4.323113000 | 10.370425000 | -0.152768000 |
| H | 3.577088000 | 8.248409000 | 1.996382000 |
| H | 2.166372000 | 7.564395000 | 1.166016000 |
| H | 2.414405000 | 9.319744000 | 1.194998000 |
| H | 3.362874000 | 8.389799000 | -2.342149000 |
| H | 2.284704000 | 9.399326000 | -1.362104000 |
| H | 2.040758000 | 7.644270000 | -1.423509000 |
| H | 6.115380000 | 4.898056000 | -0.392839000 |
| H | 6.597035000 | 2.485545000 | -0.351225000 |
| H | 1.931035000 | 3.916696000 | -0.168016000 |
| H | -2.941591000 | 4.156907000 | -0.610931000 |
| H | -5.335661000 | 5.431640000 | -0.820582000 |
| H | -7.193154000 | 3.405197000 | -0.640121000 |

Cartesian coordinates of DFT-optimized structure of **10box** complex by B3LYP/Def2-TZVP; 6-31G(d,p)/$CH_3CN$, charge = 3, multiplicity = 2

| | | | |
|---|---|---|---|
| Ru | 5.410683000 | 0.838151000 | -0.025072000 |
| N | 5.345468000 | -1.242360000 | 0.297304000 |
| N | 5.776024000 | 1.259457000 | 2.004861000 |
| N | 7.451902000 | 1.211724000 | 0.010534000 |
| N | 5.972178000 | 0.565598000 | -2.034235000 |
| C | 4.861387000 | 1.251379000 | 2.993613000 |
| H | 3.849449000 | 1.000969000 | 2.700591000 |
| C | 5.179815000 | 1.542529000 | 4.316195000 |
| H | 4.400443000 | 1.521071000 | 5.069546000 |
| C | 6.498880000 | 1.855121000 | 4.638216000 |
| H | 6.783458000 | 2.087604000 | 5.658935000 |
| C | 7.453655000 | 1.862609000 | 3.624763000 |
| H | 8.486537000 | 2.099067000 | 3.850542000 |
| C | 7.079201000 | 1.562197000 | 2.314433000 |
| C | 8.030190000 | 1.543342000 | 1.183600000 |
| C | 9.398306000 | 1.825499000 | 1.230511000 |

| | | | |
|---|---|---|---|
| H | 9.885230000 | 2.092815000 | 2.160671000 |
| C | 10.134740000 | 1.757749000 | 0.046472000 |
| C | 9.513462000 | 1.416401000 | -1.156214000 |
| H | 10.089565000 | 1.367718000 | -2.072375000 |
| C | 8.142884000 | 1.143444000 | -1.146379000 |
| C | 7.303262000 | 0.768970000 | -2.303289000 |
| C | 7.803442000 | 0.622569000 | -3.598061000 |
| H | 8.856310000 | 0.788269000 | -3.792422000 |
| C | 6.947271000 | 0.262771000 | -4.635661000 |
| H | 7.329386000 | 0.147251000 | -5.644347000 |
| C | 5.598631000 | 0.055427000 | -4.353057000 |
| H | 4.892543000 | -0.226341000 | -5.125999000 |
| C | 5.153196000 | 0.216653000 | -3.045113000 |
| H | 4.113212000 | 0.067360000 | -2.782774000 |
| C | 6.409956000 | -2.067021000 | 0.358753000 |
| H | 7.381415000 | -1.586759000 | 0.386796000 |
| C | 6.310609000 | -3.444556000 | 0.374959000 |
| H | 7.195535000 | -4.067917000 | 0.424173000 |
| C | 5.034436000 | -4.023905000 | 0.289505000 |
| C | 3.917510000 | -3.178935000 | 0.237321000 |
| H | 2.929580000 | -3.595385000 | 0.121997000 |
| C | 4.080377000 | -1.788923000 | 0.279526000 |
| C | -2.995636000 | 0.788548000 | -0.205442000 |
| C | 2.995742000 | -0.787298000 | 0.204425000 |
| C | -3.496001000 | -0.511953000 | 0.041805000 |
| C | 3.496109000 | 0.513209000 | -0.042730000 |
| C | -2.614888000 | -1.587796000 | 0.280895000 |
| C | 2.614996000 | 1.589057000 | -0.281779000 |
| C | -1.232813000 | -1.387869000 | 0.415463000 |
| C | 1.232931000 | 1.389120000 | -0.416420000 |
| C | -0.705613000 | -0.100743000 | 0.011448000 |
| C | 0.705727000 | 0.101987000 | -0.012439000 |
| C | -1.603307000 | 0.974803000 | -0.364410000 |
| C | 1.603419000 | -0.973565000 | 0.363391000 |
| C | -1.020556000 | 2.147809000 | -0.947246000 |
| C | 1.020680000 | -2.146585000 | 0.946205000 |
| H | -1.661088000 | 2.866744000 | -1.441051000 |
| H | 1.661223000 | -2.865538000 | 1.439970000 |
| C | 0.324389000 | 2.348984000 | -0.968035000 |
| C | -0.324266000 | -2.347749000 | 0.967039000 |
| H | 0.712592000 | 3.221827000 | -1.477134000 |
| H | -0.712468000 | -3.220603000 | 1.476125000 |
| O | 4.983807000 | -5.365203000 | 0.255380000 |
| C | 3.708848000 | -6.031948000 | 0.160904000 |
| C | 3.936441000 | -7.550132000 | 0.139253000 |
| C | 2.547965000 | -8.212031000 | 0.039081000 |
| C | 4.788137000 | -7.939792000 | -1.083819000 |
| C | 4.633632000 | -7.998989000 | 1.437455000 |
| H | 3.093515000 | -5.746477000 | 1.023842000 |
| H | 3.203477000 | -5.706089000 | -0.757058000 |
| H | 2.650596000 | -9.302779000 | 0.019763000 |

| | | | |
|---|---|---|---|
| H | 2.023453000 | -7.908563000 | -0.875209000 |
| H | 1.916518000 | -7.950504000 | 0.896944000 |
| H | 4.300970000 | -7.634584000 | -2.018260000 |
| H | 4.930252000 | -9.026011000 | -1.118332000 |
| H | 5.775781000 | -7.469133000 | -1.045906000 |
| H | 4.033746000 | -7.737867000 | 2.318040000 |
| H | 5.616477000 | -7.528729000 | 1.543937000 |
| H | 4.775486000 | -9.085817000 | 1.437731000 |
| H | 11.197679000 | 1.973279000 | 0.060737000 |
| N | 3.297137000 | 2.829692000 | -0.394305000 |
| C | 2.897607000 | 4.137395000 | -0.401384000 |
| C | 4.027881000 | 4.926873000 | -0.422840000 |
| C | 5.111537000 | 4.028248000 | -0.403314000 |
| N | 4.678209000 | 2.769066000 | -0.373432000 |
| N | -3.297034000 | -2.828414000 | 0.393573000 |
| N | -4.678107000 | -2.767779000 | 0.372775000 |
| C | -5.111437000 | -4.026957000 | 0.402792000 |
| C | -4.027785000 | -4.925587000 | 0.422359000 |
| C | -2.897507000 | -4.136118000 | 0.400785000 |
| C | -4.080284000 | 1.790164000 | -0.280606000 |
| C | -3.917432000 | 3.180183000 | -0.238512000 |
| C | -5.034366000 | 4.025138000 | -0.290777000 |
| C | -6.310533000 | 3.445771000 | -0.376189000 |
| C | -6.409868000 | 2.068236000 | -0.359860000 |
| N | -5.345372000 | 1.243589000 | -0.298341000 |
| O | -4.983746000 | 5.366437000 | -0.256773000 |
| C | -3.708791000 | 6.033196000 | -0.162346000 |
| C | -3.936389000 | 7.551382000 | -0.140843000 |
| C | -2.547918000 | 8.213295000 | -0.040708000 |
| C | -4.633557000 | 8.000112000 | -1.439101000 |
| C | -4.788109000 | 7.941156000 | 1.082175000 |
| H | -6.173044000 | -4.228796000 | 0.396870000 |
| H | -4.066149000 | -6.004091000 | 0.438919000 |
| H | -1.858335000 | -4.412878000 | 0.357632000 |
| H | -2.929508000 | 3.596653000 | -0.123217000 |
| H | -7.195464000 | 4.069119000 | -0.425458000 |
| H | -7.381323000 | 1.587963000 | -0.387854000 |
| H | -3.093446000 | 5.747645000 | -1.025249000 |
| H | -3.203430000 | 5.707426000 | 0.755653000 |
| H | -2.023422000 | 7.909918000 | 0.873622000 |
| H | -2.650552000 | 9.304044000 | -0.021499000 |
| H | -1.916452000 | 7.951685000 | -0.898533000 |
| H | -4.033656000 | 7.738905000 | -2.319650000 |
| H | -4.775411000 | 9.086940000 | -1.439485000 |
| H | -5.616400000 | 7.529841000 | -1.545555000 |
| H | -4.300957000 | 7.636040000 | 2.016655000 |
| H | -5.775750000 | 7.470487000 | 1.044290000 |
| H | -4.930230000 | 9.027377000 | 1.116580000 |
| Ru | -5.410571000 | -0.836888000 | 0.024250000 |
| C | -5.598343000 | -0.053692000 | 4.352153000 |
| C | -5.152966000 | -0.215063000 | 3.044207000 |

| | | | |
|---|---|---|---|
| N | -5.971995000 | -0.564124000 | 2.033406000 |
| C | -7.303067000 | -0.767466000 | 2.302540000 |
| C | -7.803188000 | -0.620923000 | 3.597318000 |
| C | -6.946970000 | -0.261006000 | 4.634840000 |
| C | -8.142737000 | -1.142062000 | 1.145707000 |
| N | -7.451808000 | -1.210461000 | -0.011228000 |
| C | -8.030147000 | -1.542196000 | -1.184232000 |
| C | -9.398266000 | -1.824358000 | -1.231054000 |
| C | -10.134647000 | -1.756484000 | -0.046990000 |
| C | -9.513315000 | -1.415015000 | 1.155634000 |
| C | -7.079211000 | -1.561171000 | -2.315106000 |
| N | -5.776021000 | -1.258402000 | -2.005622000 |
| C | -4.861424000 | -1.250430000 | -2.994412000 |
| C | -5.179910000 | -1.541721000 | -4.316950000 |
| C | -6.498989000 | -1.854344000 | -4.638880000 |
| C | -7.453722000 | -1.861720000 | -3.625386000 |
| H | -4.112994000 | -0.065796000 | 2.781803000 |
| H | -4.892220000 | 0.228166000 | 5.125031000 |
| H | -7.329040000 | -0.145372000 | 5.643530000 |
| H | -8.856046000 | -0.786601000 | 3.791746000 |
| H | -10.089377000 | -1.366235000 | 2.071815000 |
| H | -11.197588000 | -1.972014000 | -0.061186000 |
| H | -9.885231000 | -2.091769000 | -2.161165000 |
| H | -8.486614000 | -2.098200000 | -3.851096000 |
| H | -6.783611000 | -2.086935000 | -5.659563000 |
| H | -4.400569000 | -1.520346000 | -5.070336000 |
| H | -3.849472000 | -0.999993000 | -2.701459000 |
| H | 4.066240000 | 6.005379000 | -0.439289000 |
| H | 1.858436000 | 4.414151000 | -0.358233000 |
| H | 6.173142000 | 4.230093000 | -0.397330000 |

Cartesian coordinates of DFT-optimized structure of **11box** complex by B3LYP/Def2-TZVP; 6-31G(d,p)/$CH_3CN$, charge = 3, multiplicity = 2

| | | | |
|---|---|---|---|
| Ru | 5.585845000 | -0.946404000 | 0.034714000 |
| N | 5.145442000 | 1.089885000 | -0.269824000 |
| N | 6.041458000 | -1.276529000 | -1.991963000 |
| N | 7.652075000 | -0.948604000 | 0.029920000 |
| N | 6.050131000 | -0.592822000 | 2.054799000 |
| N | -5.055348000 | 0.810868000 | -0.265451000 |
| C | 5.156032000 | -1.427222000 | -2.995953000 |
| H | 4.111207000 | -1.370709000 | -2.716382000 |
| C | 5.540854000 | -1.643615000 | -4.315112000 |
| H | 4.781761000 | -1.758090000 | -5.080777000 |
| C | 6.899621000 | -1.708136000 | -4.618291000 |
| H | 7.236492000 | -1.874984000 | -5.635939000 |
| C | 7.824756000 | -1.553676000 | -3.589349000 |
| H | 8.886641000 | -1.598687000 | -3.800078000 |
| C | 7.382821000 | -1.338687000 | -2.282648000 |
| C | 8.298310000 | -1.163602000 | -1.135976000 |

| | | | |
|---|---|---|---|
| C | 9.695186000 | -1.204082000 | -1.160651000 |
| H | 10.235687000 | -1.376499000 | -2.083672000 |
| C | 10.390365000 | -1.019273000 | 0.035912000 |
| C | 9.700334000 | -0.799944000 | 1.229670000 |
| H | 10.245227000 | -0.659521000 | 2.155492000 |
| C | 8.303346000 | -0.768942000 | 1.199093000 |
| C | 7.392926000 | -0.555716000 | 2.343525000 |
| C | 7.840631000 | -0.326702000 | 3.645846000 |
| H | 8.903428000 | -0.299001000 | 3.854943000 |
| C | 6.920094000 | -0.131804000 | 4.672098000 |
| H | 7.261599000 | 0.046301000 | 5.686289000 |
| C | 5.560017000 | -0.168525000 | 4.370402000 |
| H | 4.804393000 | -0.020993000 | 5.133837000 |
| C | 5.169229000 | -0.400059000 | 3.055535000 |
| H | 4.123034000 | -0.435498000 | 2.777524000 |
| C | 6.045667000 | 2.091616000 | -0.326973000 |
| H | 7.087083000 | 1.791691000 | -0.354377000 |
| C | 5.702835000 | 3.429716000 | -0.340551000 |
| H | 6.462716000 | 4.200776000 | -0.388185000 |
| C | 4.344072000 | 3.772697000 | -0.255475000 |
| C | 3.395799000 | 2.742792000 | -0.201897000 |
| H | 2.349638000 | 2.975597000 | -0.083302000 |
| C | 3.803087000 | 1.403438000 | -0.250383000 |
| C | -2.684306000 | -2.346291000 | 0.265519000 |
| C | 2.913320000 | 0.225551000 | -0.184847000 |
| C | -3.424907000 | -1.168131000 | 0.038745000 |
| C | 3.637885000 | -0.968943000 | 0.038596000 |
| C | -2.773067000 | 0.067516000 | -0.186767000 |
| C | 2.959919000 | -2.183528000 | 0.263698000 |
| C | -3.725297000 | 1.196482000 | -0.248268000 |
| C | -3.401314000 | 2.548175000 | -0.198425000 |
| H | -2.377820000 | 2.876408000 | -0.089038000 |
| C | -4.403838000 | 3.532159000 | -0.246690000 |
| C | -5.740549000 | 3.122561000 | -0.328993000 |
| H | -6.565298000 | 3.821018000 | -0.376154000 |
| C | -6.002958000 | 1.758134000 | -0.316458000 |
| H | -7.027792000 | 1.404921000 | -0.338643000 |
| C | -1.366933000 | 0.089062000 | -0.331718000 |
| C | 1.567130000 | -2.231801000 | 0.412629000 |
| C | -0.609971000 | -1.088664000 | 0.041895000 |
| C | 0.815956000 | -1.047586000 | 0.042598000 |
| C | -1.291318000 | -2.313885000 | 0.414941000 |
| C | 1.505830000 | 0.169325000 | -0.328210000 |
| C | -0.509957000 | -3.390398000 | 0.950251000 |
| C | 0.719025000 | 1.225130000 | -0.894183000 |
| H | -1.005629000 | -4.225093000 | 1.429051000 |
| H | 1.216396000 | 2.045534000 | -1.395536000 |
| C | 0.848167000 | -3.350556000 | 0.949283000 |
| C | -0.641317000 | 1.188865000 | -0.894067000 |
| H | 1.391326000 | -4.156566000 | 1.425825000 |
| H | -1.184242000 | 1.981104000 | -1.393183000 |

| | | | |
|---|---|---|---|
| O | 4.055903000 | 5.084518000 | -0.223982000 |
| O | -3.981947000 | 4.804659000 | -0.200905000 |
| C | -4.950064000 | 5.874450000 | -0.238849000 |
| C | -4.214905000 | 7.219837000 | -0.161105000 |
| C | -5.290808000 | 8.322804000 | -0.205499000 |
| C | -3.262942000 | 7.373698000 | -1.362371000 |
| C | -3.425180000 | 7.316943000 | 1.158030000 |
| C | 2.682491000 | 5.513367000 | -0.139568000 |
| C | 2.633218000 | 7.047767000 | -0.161183000 |
| C | 1.149252000 | 7.452776000 | -0.062419000 |
| C | 3.409168000 | 7.620098000 | 1.040232000 |
| C | 3.229699000 | 7.576900000 | -1.479222000 |
| H | -5.638286000 | 5.759903000 | 0.608015000 |
| H | -5.522617000 | 5.800669000 | -1.171969000 |
| H | -5.985364000 | 8.240685000 | 0.639403000 |
| H | -5.875170000 | 8.276010000 | -1.132495000 |
| H | -4.819836000 | 9.310950000 | -0.156428000 |
| H | -3.810736000 | 7.316743000 | -2.311108000 |
| H | -2.496848000 | 6.591447000 | -1.364672000 |
| H | -2.756535000 | 8.344973000 | -1.324445000 |
| H | -4.089157000 | 7.213522000 | 2.025164000 |
| H | -2.925868000 | 8.289821000 | 1.231525000 |
| H | -2.659543000 | 6.536909000 | 1.221531000 |
| H | 2.126844000 | 5.098307000 | -0.990261000 |
| H | 2.244898000 | 5.127300000 | 0.789937000 |
| H | 1.054580000 | 8.544311000 | -0.077747000 |
| H | 0.695761000 | 7.090358000 | 0.868215000 |
| H | 0.567441000 | 7.054333000 | -0.902641000 |
| H | 2.994264000 | 7.256586000 | 1.988528000 |
| H | 3.349677000 | 8.714555000 | 1.045088000 |
| H | 4.466238000 | 7.338172000 | 1.001389000 |
| H | 2.679177000 | 7.189230000 | -2.345424000 |
| H | 4.279493000 | 7.285214000 | -1.585074000 |
| H | 3.176021000 | 8.671201000 | -1.509276000 |
| H | 11.474678000 | -1.047594000 | 0.038378000 |
| N | 3.854418000 | -3.286481000 | 0.358567000 |
| C | 3.695767000 | -4.643162000 | 0.332627000 |
| C | 4.949733000 | -5.219013000 | 0.348039000 |
| C | 5.854395000 | -4.141670000 | 0.359166000 |
| N | 5.202281000 | -2.978865000 | 0.352680000 |
| N | -3.514745000 | -3.497349000 | 0.356747000 |
| C | -3.279413000 | -4.842554000 | 0.327443000 |
| C | -4.498715000 | -5.488341000 | 0.337983000 |
| C | -5.462874000 | -4.464232000 | 0.349016000 |
| N | -4.876714000 | -3.266964000 | 0.347676000 |
| H | 6.934945000 | -4.149930000 | 0.358374000 |
| H | 5.179674000 | -6.273609000 | 0.341175000 |
| H | 2.723384000 | -5.100409000 | 0.271506000 |
| H | -2.282326000 | -5.243687000 | 0.269768000 |
| H | -4.668911000 | -6.554069000 | 0.329056000 |
| H | -6.541113000 | -4.535214000 | 0.347659000 |

| | | | |
|---|---|---|---|
| Ru | -5.363058000 | -1.252899000 | 0.030146000 |
| C | -5.287482000 | -1.888637000 | -4.323209000 |
| C | -4.918924000 | -1.662871000 | -3.000823000 |
| N | -5.816082000 | -1.586309000 | -1.999179000 |
| C | -7.148630000 | -1.734459000 | -2.291949000 |
| C | -7.573489000 | -1.961709000 | -3.601306000 |
| C | -6.637899000 | -2.041015000 | -4.629676000 |
| C | -8.073274000 | -1.635158000 | -1.143966000 |
| C | -9.463670000 | -1.774163000 | -1.170957000 |
| C | -10.168927000 | -1.652139000 | 0.027375000 |
| C | -9.496058000 | -1.396686000 | 1.223393000 |
| C | -8.105054000 | -1.265519000 | 1.191823000 |
| N | -7.444782000 | -1.386513000 | 0.022593000 |
| C | -7.211994000 | -0.996572000 | 2.337821000 |
| N | -5.871885000 | -0.937010000 | 2.047859000 |
| C | -5.001691000 | -0.687248000 | 3.045038000 |
| C | -5.405529000 | -0.489822000 | 4.361641000 |
| C | -6.763632000 | -0.550471000 | 4.665869000 |
| C | -7.671602000 | -0.805239000 | 3.641162000 |
| H | -4.521143000 | -1.941647000 | -5.088145000 |
| H | -3.880826000 | -1.538928000 | -2.719441000 |
| H | -8.629576000 | -2.075117000 | -3.814409000 |
| H | -6.961217000 | -2.217476000 | -5.649990000 |
| H | -9.990460000 | -1.973739000 | -2.096286000 |
| H | -11.248401000 | -1.757458000 | 0.029387000 |
| H | -10.048785000 | -1.304966000 | 2.150533000 |
| H | -3.956176000 | -0.648836000 | 2.766263000 |
| H | -4.660041000 | -0.293287000 | 5.123735000 |
| H | -7.114160000 | -0.401353000 | 5.681549000 |
| H | -8.733023000 | -0.853378000 | 3.852284000 |

Cartesian coordinates of DFT-optimized structure of **12** complex by B3LYP/Def2-TZVP; 6-31G(d,p)/$CH_3CN$, charge = 2, multiplicity = 1

| | | | |
|---|---|---|---|
| Ru | -5.533455000 | -0.048539000 | 0.032201000 |
| Os | 5.539396000 | 0.094213000 | 0.001424000 |
| N | -5.080682000 | -2.087615000 | 0.301356000 |
| N | 5.069060000 | 2.134228000 | -0.256314000 |
| N | -5.986330000 | 0.247223000 | 2.056049000 |
| N | 6.001073000 | -0.195696000 | -2.021306000 |
| N | -7.574028000 | -0.077861000 | 0.021893000 |
| N | 7.583709000 | 0.122360000 | 0.011582000 |
| N | -5.957314000 | -0.356465000 | -1.997991000 |
| N | 5.972453000 | 0.395549000 | 2.030387000 |
| N | -5.139508000 | 2.003216000 | -0.232282000 |
| N | 5.127573000 | -1.958657000 | 0.255013000 |
| C | -5.110341000 | 0.409945000 | 3.068163000 |
| C | 5.124932000 | -0.355633000 | -3.041150000 |
| H | 4.078585000 | -0.324433000 | -2.763439000 |
| C | -5.504672000 | 0.604165000 | 4.387156000 |

| | | | |
|---|---|---|---|
| C | 5.522175000 | -0.547367000 | -4.356529000 |
| H | 4.769860000 | -0.669740000 | -5.128006000 |
| C | -6.867177000 | 0.633556000 | 4.686110000 |
| C | 6.886459000 | -0.579246000 | -4.655836000 |
| C | -7.783583000 | 0.467037000 | 3.652175000 |
| C | 7.800541000 | -0.416944000 | -3.621136000 |
| H | -8.847384000 | 0.486066000 | 3.858554000 |
| C | -7.332159000 | 0.274730000 | 2.343670000 |
| C | 7.352676000 | -0.225943000 | -2.310681000 |
| C | -8.237007000 | 0.088261000 | 1.192380000 |
| C | 8.254189000 | -0.043828000 | -1.163593000 |
| C | -9.634151000 | 0.066885000 | 1.210940000 |
| C | 9.650186000 | -0.023422000 | -1.176770000 |
| H | -10.184299000 | 0.197334000 | 2.135610000 |
| H | 10.199705000 | -0.153462000 | -2.101953000 |
| C | -10.319751000 | -0.127843000 | 0.009600000 |
| C | 10.337219000 | 0.169139000 | 0.024293000 |
| C | -9.616973000 | -0.297809000 | -1.185461000 |
| C | 9.633144000 | 0.338413000 | 1.218906000 |
| C | -8.220062000 | -0.268883000 | -1.154046000 |
| C | 8.237334000 | 0.311867000 | 1.192505000 |
| C | -7.299159000 | -0.426423000 | -2.296796000 |
| C | 7.320061000 | 0.466764000 | 2.330995000 |
| C | -7.733689000 | -0.632397000 | -3.608847000 |
| C | 7.751307000 | 0.671051000 | 3.644912000 |
| H | -8.794693000 | -0.685078000 | -3.823507000 |
| C | -6.804309000 | -0.769884000 | -4.635410000 |
| C | 6.824525000 | 0.805575000 | 4.672251000 |
| C | -5.446002000 | -0.696604000 | -4.325393000 |
| C | 5.464454000 | 0.730956000 | 4.361934000 |
| H | 4.702438000 | 0.829451000 | 5.127314000 |
| C | -5.068685000 | -0.490047000 | -3.003181000 |
| C | 5.083867000 | 0.527063000 | 3.043332000 |
| H | 4.041321000 | 0.462151000 | 2.757525000 |
| C | -5.978960000 | -3.093900000 | 0.332298000 |
| C | 5.966616000 | 3.146835000 | -0.288417000 |
| H | -7.021812000 | -2.797101000 | 0.347004000 |
| H | 7.009686000 | 2.852650000 | -0.297098000 |
| C | -5.636464000 | -4.431797000 | 0.334523000 |
| C | 5.619436000 | 4.481178000 | -0.298532000 |
| H | -6.396159000 | -5.204262000 | 0.362077000 |
| H | 6.377773000 | 5.254877000 | -0.325902000 |
| C | -4.274971000 | -4.770592000 | 0.265160000 |
| C | 4.256003000 | 4.817910000 | -0.237133000 |
| C | -3.329777000 | -3.740770000 | 0.236591000 |
| C | 3.313997000 | 3.786783000 | -0.208920000 |
| H | -2.281675000 | -3.970761000 | 0.130701000 |
| H | 2.265181000 | 4.015231000 | -0.108269000 |
| C | -3.735929000 | -2.397550000 | 0.295841000 |
| C | 3.718108000 | 2.442383000 | -0.260276000 |
| C | -2.847814000 | -1.223629000 | 0.252803000 |

| | | | |
|---|---|---|---|
| C | 2.830303000 | 1.272430000 | -0.217049000 |
| C | -3.558703000 | -0.022528000 | 0.036307000 |
| C | 3.544244000 | 0.068468000 | -0.002143000 |
| C | -2.880642000 | 1.195394000 | -0.187962000 |
| C | 2.862885000 | -1.152328000 | 0.220820000 |
| C | -3.796475000 | 2.348076000 | -0.229138000 |
| C | 3.778428000 | -2.301532000 | 0.261904000 |
| C | -3.436076000 | 3.695822000 | -0.170214000 |
| C | 3.419680000 | -3.650236000 | 0.211275000 |
| H | -2.401274000 | 3.991660000 | -0.073304000 |
| H | 2.383937000 | -3.944089000 | 0.120420000 |
| C | -4.406655000 | 4.706650000 | -0.191208000 |
| C | 4.386727000 | -4.662880000 | 0.231977000 |
| C | -5.757385000 | 4.338569000 | -0.257561000 |
| C | 5.739443000 | -4.296977000 | 0.289801000 |
| H | -6.562068000 | 5.061188000 | -0.282826000 |
| H | 6.542644000 | -5.021157000 | 0.314884000 |
| C | -6.057536000 | 2.982821000 | -0.259128000 |
| C | 6.044834000 | -2.944814000 | 0.283158000 |
| H | -7.093120000 | 2.661057000 | -0.272362000 |
| H | 7.080749000 | -2.625914000 | 0.290426000 |
| C | -1.466800000 | 1.194536000 | -0.346448000 |
| C | 1.450079000 | -1.149673000 | 0.380104000 |
| C | -0.728136000 | 0.013315000 | 0.021196000 |
| C | 0.711964000 | 0.032861000 | 0.013553000 |
| C | -1.431930000 | -1.186433000 | 0.397257000 |
| C | 1.415099000 | 1.233593000 | -0.361791000 |
| C | -0.655969000 | -2.247352000 | 0.963854000 |
| C | 0.638183000 | 2.293684000 | -0.929027000 |
| H | -1.159280000 | -3.062292000 | 1.469720000 |
| H | 1.140735000 | 3.108443000 | -1.435749000 |
| C | 0.708438000 | -2.230532000 | 0.954564000 |
| C | -0.726284000 | 2.276100000 | -0.920133000 |
| H | 1.239615000 | -3.033396000 | 1.450849000 |
| H | -1.258218000 | 3.079216000 | -1.415377000 |
| O | -3.984120000 | -6.085663000 | 0.223156000 |
| O | 3.927019000 | -5.928760000 | 0.184172000 |
| O | 3.963335000 | 6.132629000 | -0.200928000 |
| O | -3.949177000 | 5.973133000 | -0.135964000 |
| C | -4.894376000 | 7.059464000 | -0.134739000 |
| C | -4.135115000 | 8.391701000 | -0.055836000 |
| C | -5.192181000 | 9.513372000 | -0.051631000 |
| C | -3.215437000 | 8.549834000 | -1.281464000 |
| C | -3.307218000 | 8.454393000 | 1.241672000 |
| C | 2.588310000 | 6.550830000 | -0.127070000 |
| C | 2.525398000 | 8.085117000 | -0.150815000 |
| C | 1.038746000 | 8.478027000 | -0.047890000 |
| C | 3.300617000 | 8.666577000 | 1.046616000 |
| C | 3.113697000 | 8.615927000 | -1.471926000 |
| C | -2.610157000 | -6.506079000 | 0.142823000 |
| C | -2.550311000 | -8.040614000 | 0.159673000 |

| | | | |
|---|---|---|---|
| C | -3.136307000 | -8.576196000 | 1.479869000 |
| C | -3.329768000 | -8.615109000 | -1.038371000 |
| C | -1.064764000 | -8.436140000 | 0.051161000 |
| C | 4.870343000 | -7.016852000 | 0.183611000 |
| C | 4.108279000 | -8.348062000 | 0.115178000 |
| C | 3.274065000 | -8.415893000 | -1.178005000 |
| C | 3.194331000 | -8.498201000 | 1.346077000 |
| C | 5.163378000 | -9.471593000 | 0.111639000 |
| H | -5.565847000 | 6.947992000 | 0.726449000 |
| H | -5.492716000 | 7.013926000 | -1.053920000 |
| H | -5.863973000 | 9.426983000 | 0.811003000 |
| H | -5.802851000 | 9.492257000 | -0.962432000 |
| H | -4.703709000 | 10.492846000 | 0.001151000 |
| H | -3.791800000 | 8.517188000 | -2.214467000 |
| H | -2.462980000 | 7.755260000 | -1.319574000 |
| H | -2.691660000 | 9.512016000 | -1.243816000 |
| H | -3.950088000 | 8.356828000 | 2.125355000 |
| H | -2.782114000 | 9.413930000 | 1.312803000 |
| H | -2.559934000 | 7.654975000 | 1.275910000 |
| H | 2.039873000 | 6.132274000 | -0.980976000 |
| H | 2.145521000 | 6.161319000 | 0.798996000 |
| H | 0.933869000 | 9.568684000 | -0.066801000 |
| H | 0.591463000 | 8.114903000 | 0.885531000 |
| H | 0.457032000 | 8.071788000 | -0.884405000 |
| H | 2.889324000 | 8.304537000 | 1.997041000 |
| H | 3.235603000 | 9.760813000 | 1.047346000 |
| H | 4.358797000 | 8.389059000 | 1.006756000 |
| H | 2.562012000 | 8.223934000 | -2.335515000 |
| H | 4.164473000 | 8.328827000 | -1.581422000 |
| H | 3.054994000 | 9.709948000 | -1.504181000 |
| H | -2.057888000 | -6.092333000 | 0.996617000 |
| H | -2.169550000 | -6.113719000 | -0.783072000 |
| H | -2.581783000 | -8.189034000 | 2.343817000 |
| H | -3.079532000 | -9.670458000 | 1.507183000 |
| H | -4.186276000 | -8.287677000 | 1.593247000 |
| H | -4.387240000 | -8.335491000 | -0.994637000 |
| H | -3.267108000 | -9.709472000 | -1.044151000 |
| H | -2.920065000 | -8.249724000 | -1.988211000 |
| H | -0.480358000 | -8.035680000 | 0.888571000 |
| H | -0.618888000 | -8.068811000 | -0.881285000 |
| H | -0.962164000 | -9.527104000 | 0.063862000 |
| H | 5.473249000 | -6.967862000 | 1.099603000 |
| H | 5.537636000 | -6.910883000 | -0.681501000 |
| H | 2.747028000 | -9.374888000 | -1.241738000 |
| H | 3.912870000 | -8.323904000 | -2.065224000 |
| H | 2.527946000 | -7.615409000 | -1.212764000 |
| H | 2.444015000 | -7.701611000 | 1.384173000 |
| H | 3.775431000 | -8.462528000 | 2.276025000 |
| H | 2.668071000 | -9.459278000 | 1.315589000 |
| H | 5.831081000 | -9.390932000 | -0.754716000 |
| H | 4.672910000 | -10.450441000 | 0.066355000 |

| | | | |
|---|---|---|---|
| H | 5.778506000 | -9.446831000 | 1.019343000 |
| H | -4.752024000 | 0.729455000 | 5.157832000 |
| H | -7.211558000 | 0.782999000 | 5.704118000 |
| H | -4.063775000 | 0.379899000 | 2.789165000 |
| H | -10.153120000 | -0.448349000 | -2.115325000 |
| H | -11.404314000 | -0.147272000 | 0.004843000 |
| H | -7.135566000 | -0.930361000 | -5.656095000 |
| H | -4.683446000 | -0.797354000 | -5.089934000 |
| H | -4.025968000 | -0.425693000 | -2.715910000 |
| H | 7.230925000 | -0.727393000 | -5.673850000 |
| H | 8.864687000 | -0.437821000 | -3.825591000 |
| H | 11.421645000 | 0.187274000 | 0.029205000 |
| H | 10.168792000 | 0.486996000 | 2.149451000 |
| H | 8.812671000 | 0.724448000 | 3.857635000 |
| H | 7.156087000 | 0.964616000 | 5.692907000 |

Cartesian coordinates of DFT-optimized structure of **13** complex by B3LYP/Def2-TZVP; 6-31G(d,p)/$CH_3CN$, charge = 2, multiplicity = 1

| | | | |
|---|---|---|---|
| Ru | 4.994221000 | -0.359596000 | -0.209237000 |
| N | 5.481123000 | 0.159256000 | 1.766317000 |
| N | 7.030483000 | -0.457727000 | -0.236939000 |
| N | 5.375215000 | -0.920043000 | -2.196643000 |
| C | 4.627950000 | 0.467990000 | 2.762087000 |
| H | 3.575977000 | 0.437333000 | 2.504040000 |
| C | 5.050203000 | 0.805586000 | 4.043657000 |
| H | 4.314840000 | 1.045403000 | 4.803749000 |
| C | 6.417215000 | 0.826803000 | 4.318169000 |
| H | 6.783649000 | 1.085734000 | 5.306047000 |
| C | 7.310658000 | 0.508100000 | 3.299302000 |
| H | 8.377732000 | 0.516593000 | 3.488541000 |
| C | 6.830949000 | 0.176758000 | 2.029834000 |
| C | 7.712024000 | -0.174099000 | 0.897039000 |
| C | 9.108254000 | -0.234439000 | 0.903187000 |
| H | 9.674895000 | -0.012079000 | 1.799746000 |
| C | 9.769830000 | -0.590107000 | -0.274080000 |
| C | 9.045445000 | -0.878786000 | -1.432556000 |
| H | 9.563541000 | -1.153612000 | -2.343809000 |
| C | 7.650580000 | -0.804228000 | -1.388574000 |
| C | 6.709448000 | -1.066951000 | -2.496677000 |
| C | 7.120450000 | -1.440874000 | -3.778369000 |
| H | 8.176228000 | -1.551687000 | -3.996521000 |
| C | 6.173187000 | -1.670441000 | -4.772295000 |
| H | 6.486076000 | -1.961820000 | -5.769502000 |
| C | 4.822514000 | -1.518170000 | -4.460816000 |
| H | 4.047152000 | -1.684836000 | -5.200443000 |
| C | 4.469609000 | -1.143593000 | -3.168594000 |
| H | 3.432852000 | -1.012194000 | -2.882163000 |
| C | -3.313390000 | 1.205481000 | -0.411569000 |
| C | 2.259211000 | -1.389270000 | 0.150934000 |

| | | | |
|---|---|---|---|
| C | -4.071288000 | 0.078909000 | -0.076463000 |
| C | 3.014276000 | -0.264261000 | -0.181801000 |
| C | -3.416280000 | -1.116317000 | 0.237013000 |
| C | 2.361804000 | 0.928702000 | -0.494211000 |
| C | -2.011021000 | -1.195211000 | 0.341044000 |
| C | 0.955477000 | 1.008191000 | -0.600362000 |
| C | -1.245984000 | -0.058280000 | -0.119326000 |
| C | 0.189846000 | -0.127820000 | -0.140672000 |
| C | -1.910890000 | 1.148008000 | -0.558204000 |
| C | 0.855452000 | -1.333046000 | 0.297978000 |
| C | -1.114553000 | 2.171332000 | -1.163435000 |
| C | 0.060718000 | -2.357155000 | 0.902920000 |
| H | -1.595982000 | 2.990883000 | -1.680836000 |
| H | 0.543538000 | -3.177104000 | 1.418688000 |
| C | 0.245815000 | 2.104986000 | -1.183262000 |
| C | -1.299683000 | -2.291731000 | 0.923535000 |
| H | 0.790087000 | 2.875074000 | -1.714465000 |
| H | -1.842487000 | -3.061795000 | 1.455994000 |
| H | 10.853233000 | -0.642483000 | -0.288765000 |
| N | -4.349661000 | -2.174927000 | 0.459070000 |
| N | -5.693723000 | -1.825537000 | 0.442006000 |
| C | -6.382368000 | -2.962693000 | 0.592071000 |
| C | -5.511364000 | -4.059189000 | 0.686794000 |
| C | -4.239344000 | -3.529130000 | 0.586442000 |
| H | -7.462117000 | -2.933272000 | 0.611082000 |
| H | -5.771970000 | -5.100238000 | 0.803580000 |
| H | -3.284257000 | -4.023677000 | 0.562898000 |
| Os | -6.069743000 | 0.176238000 | -0.044847000 |
| C | -5.903101000 | 1.316927000 | 4.214066000 |
| C | -5.549331000 | 0.948199000 | 2.923229000 |
| N | -6.456414000 | 0.727605000 | 1.944657000 |
| C | -7.795562000 | 0.875426000 | 2.249666000 |
| C | -8.201069000 | 1.244717000 | 3.534709000 |
| C | -7.254580000 | 1.469549000 | 4.528783000 |
| C | -8.736582000 | 0.618996000 | 1.146591000 |
| N | -8.113260000 | 0.275704000 | -0.012035000 |
| C | -8.803787000 | -0.003732000 | -1.149522000 |
| C | -10.198839000 | 0.058152000 | -1.147746000 |
| C | -10.858627000 | 0.409066000 | 0.032153000 |
| C | -10.130399000 | 0.692568000 | 1.189710000 |
| C | -7.927917000 | -0.348341000 | -2.281851000 |
| N | -6.571666000 | -0.331669000 | -2.020119000 |
| C | -5.722068000 | -0.637401000 | -3.026988000 |
| C | -6.151106000 | -0.967701000 | -4.305347000 |
| C | -7.520399000 | -0.987964000 | -4.576373000 |
| C | -8.408290000 | -0.674218000 | -3.552760000 |
| H | -4.513185000 | 0.817187000 | 2.636269000 |
| H | -5.126806000 | 1.479919000 | 4.953555000 |
| H | -7.566056000 | 1.757132000 | 5.527373000 |
| H | -9.256807000 | 1.355322000 | 3.753147000 |
| H | -10.645891000 | 0.963759000 | 2.103578000 |

| | | | |
|---|---|---|---|
| H | -11.941833000 | 0.461669000 | 0.049592000 |
| H | -10.767250000 | -0.160420000 | -2.044159000 |
| H | -9.476229000 | -0.681479000 | -3.737124000 |
| H | -7.890122000 | -1.242142000 | -5.564098000 |
| H | -5.418370000 | -1.203844000 | -5.069139000 |
| H | -4.669437000 | -0.608004000 | -2.773583000 |
| N | -4.146085000 | 2.349986000 | -0.606265000 |
| N | -5.516525000 | 2.132692000 | -0.546543000 |
| C | -6.095916000 | 3.331490000 | -0.677158000 |
| C | -5.126006000 | 4.338371000 | -0.801163000 |
| C | -3.908919000 | 3.687236000 | -0.739629000 |
| H | -2.910099000 | 4.086753000 | -0.746963000 |
| H | -5.287872000 | 5.399931000 | -0.911731000 |
| H | -7.173486000 | 3.407034000 | -0.662341000 |
| N | 3.089010000 | -2.536467000 | 0.344562000 |
| C | 2.848073000 | -3.874673000 | 0.457883000 |
| C | 4.064977000 | -4.525715000 | 0.523460000 |
| C | 5.033663000 | -3.512825000 | 0.421328000 |
| N | 4.454871000 | -2.315694000 | 0.301068000 |
| N | 3.292305000 | 1.991012000 | -0.712855000 |
| N | 4.631296000 | 1.639871000 | -0.707180000 |
| C | 5.318931000 | 2.775897000 | -0.845680000 |
| C | 4.449663000 | 3.877345000 | -0.922478000 |
| C | 3.178108000 | 3.346297000 | -0.821772000 |
| H | 1.848829000 | -4.273728000 | 0.450961000 |
| H | 4.227582000 | -5.588337000 | 0.622643000 |
| H | 6.111642000 | -3.588004000 | 0.415945000 |
| H | 2.222485000 | 3.839639000 | -0.787651000 |
| H | 4.710855000 | 4.919497000 | -1.028033000 |
| H | 6.398870000 | 2.747046000 | -0.870546000 |

Cartesian coordinates of DFT-optimized structure of **14** complex by B3LYP/Def2-TZVP; 6-31G(d,p)/$CH_3CN$, charge = 2, multiplicity = 1

| | | | |
|---|---|---|---|
| Ru | 4.788695000 | -0.232542000 | -0.207043000 |
| N | 5.321905000 | 0.678685000 | 1.607143000 |
| N | 6.824542000 | -0.329496000 | -0.269043000 |
| N | 5.122953000 | -1.184768000 | -2.047839000 |
| C | 4.492425000 | 1.183362000 | 2.541089000 |
| H | 3.434460000 | 1.099691000 | 2.322320000 |
| C | 4.944999000 | 1.777537000 | 3.714505000 |
| H | 4.227592000 | 2.166216000 | 4.428841000 |
| C | 6.318461000 | 1.858678000 | 3.941526000 |
| H | 6.708356000 | 2.315580000 | 4.845136000 |
| C | 7.187718000 | 1.341281000 | 2.985200000 |
| H | 8.259267000 | 1.392523000 | 3.138883000 |
| C | 6.677981000 | 0.754600000 | 1.824483000 |
| C | 7.532567000 | 0.181793000 | 0.764419000 |
| C | 8.928933000 | 0.127398000 | 0.745464000 |
| H | 9.516784000 | 0.529636000 | 1.562155000 |

| | | | |
|---|---|---|---|
| C | 9.562816000 | -0.460066000 | -0.351565000 |
| C | 8.811265000 | -0.981413000 | -1.406866000 |
| H | 9.308075000 | -1.435801000 | -2.256059000 |
| C | 7.417442000 | -0.902836000 | -1.341541000 |
| C | 6.450011000 | -1.388150000 | -2.346702000 |
| C | 6.831010000 | -2.015408000 | -3.535263000 |
| H | 7.881659000 | -2.167407000 | -3.752990000 |
| C | 5.860604000 | -2.443587000 | -4.436814000 |
| H | 6.150166000 | -2.931343000 | -5.361766000 |
| C | 4.517280000 | -2.232971000 | -4.127578000 |
| H | 3.724514000 | -2.547940000 | -4.797155000 |
| C | 4.194650000 | -1.603737000 | -2.929784000 |
| H | 3.164489000 | -1.420077000 | -2.648464000 |
| C | -3.546478000 | 1.277339000 | -0.463874000 |
| C | 2.071693000 | -1.178208000 | 0.421484000 |
| C | -4.280915000 | 0.174069000 | 0.033623000 |
| C | 2.808807000 | -0.139373000 | -0.147492000 |
| C | -3.622100000 | -0.990478000 | 0.497736000 |
| C | 2.138647000 | 0.964042000 | -0.677060000 |
| C | -2.204666000 | -0.995623000 | 0.613267000 |
| C | 0.728519000 | 1.016686000 | -0.758316000 |
| C | -1.452628000 | 0.050655000 | -0.035125000 |
| C | -0.015614000 | -0.012150000 | -0.071329000 |
| C | -2.141968000 | 1.160929000 | -0.646829000 |
| C | 0.668953000 | -1.103984000 | 0.579141000 |
| C | -1.368854000 | 2.045675000 | -1.464646000 |
| C | -0.108928000 | -1.992970000 | 1.384682000 |
| H | -1.877207000 | 2.752905000 | -2.107921000 |
| H | 0.387080000 | -2.685672000 | 2.052503000 |
| C | -0.008340000 | 1.976511000 | -1.520459000 |
| C | -1.471517000 | -1.941457000 | 1.398781000 |
| H | 0.512436000 | 2.628927000 | -2.209565000 |
| H | -2.005742000 | -2.596968000 | 2.075245000 |
| H | 10.645862000 | -0.511848000 | -0.384089000 |
| Os | -6.273210000 | 0.257306000 | 0.071909000 |
| C | -6.099900000 | 1.850616000 | 4.175959000 |
| C | -5.750297000 | 1.351844000 | 2.929374000 |
| N | -6.662088000 | 1.011995000 | 1.987911000 |
| C | -8.001876000 | 1.172862000 | 2.288649000 |
| C | -8.402214000 | 1.672142000 | 3.531527000 |
| C | -7.451990000 | 2.014944000 | 4.486522000 |
| C | -8.944367000 | 0.786628000 | 1.228214000 |
| N | -8.317301000 | 0.332163000 | 0.106689000 |
| C | -9.013243000 | -0.077882000 | -0.990958000 |
| C | -10.409018000 | -0.039604000 | -0.984236000 |
| C | -11.069140000 | 0.422941000 | 0.156722000 |
| C | -10.338921000 | 0.840060000 | 1.272255000 |
| C | -8.137879000 | -0.524310000 | -2.084547000 |
| N | -6.780347000 | -0.459638000 | -1.831114000 |
| C | -5.928590000 | -0.857230000 | -2.805584000 |
| C | -6.356216000 | -1.323055000 | -4.040535000 |

| | | | |
|---|---|---|---|
| C | -7.726655000 | -1.390320000 | -4.303379000 |
| C | -8.616002000 | -0.986728000 | -3.314131000 |
| H | -4.714851000 | 1.208812000 | 2.646036000 |
| H | -5.320482000 | 2.104564000 | 4.886001000 |
| H | -7.759616000 | 2.402372000 | 5.452038000 |
| H | -9.457927000 | 1.791076000 | 3.746040000 |
| H | -10.854167000 | 1.198250000 | 2.155986000 |
| H | -12.152975000 | 0.458492000 | 0.176527000 |
| H | -10.978174000 | -0.362381000 | -1.848364000 |
| H | -9.684381000 | -1.028693000 | -3.491511000 |
| H | -8.094640000 | -1.749497000 | -5.258645000 |
| H | -5.622146000 | -1.627025000 | -4.778718000 |
| H | -4.876408000 | -0.788519000 | -2.558627000 |
| N | 2.916161000 | -2.257763000 | 0.824334000 |
| C | 2.691040000 | -3.545015000 | 1.217964000 |
| C | 3.915188000 | -4.162744000 | 1.386080000 |
| C | 4.871575000 | -3.188143000 | 1.053570000 |
| N | 4.278961000 | -2.044385000 | 0.703534000 |
| N | 3.054876000 | 1.962589000 | -1.129598000 |
| N | 4.396815000 | 1.622910000 | -1.089315000 |
| C | 5.071354000 | 2.708321000 | -1.475161000 |
| C | 4.191661000 | 3.768999000 | -1.751473000 |
| C | 2.927469000 | 3.266948000 | -1.510460000 |
| H | 1.696176000 | -3.942009000 | 1.318766000 |
| H | 4.090400000 | -5.181340000 | 1.697822000 |
| H | 5.949758000 | -3.257869000 | 1.036852000 |
| H | 1.969504000 | 3.755082000 | -1.552231000 |
| H | 4.442112000 | 4.768361000 | -2.073942000 |
| H | 6.150478000 | 2.677798000 | -1.523462000 |
| C | -4.564252000 | -2.072807000 | 0.812549000 |
| C | -4.229330000 | -3.416995000 | 1.046034000 |
| C | -5.221501000 | -4.363796000 | 1.312330000 |
| C | -6.563496000 | -3.945433000 | 1.330940000 |
| C | -6.843256000 | -2.627067000 | 1.040472000 |
| N | -5.897290000 | -1.698271000 | 0.767992000 |
| O | -4.998035000 | -5.671210000 | 1.548812000 |
| C | -3.650049000 | -6.174549000 | 1.532349000 |
| C | -3.669681000 | -7.677468000 | 1.847520000 |
| C | -2.209099000 | -8.167606000 | 1.807706000 |
| C | -4.259343000 | -7.914704000 | 3.250789000 |
| C | -4.498957000 | -8.429878000 | 0.789533000 |
| C | -4.404170000 | 2.438929000 | -0.738865000 |
| N | -5.768173000 | 2.180437000 | -0.636169000 |
| C | -6.635768000 | 3.185705000 | -0.862303000 |
| C | -6.263443000 | 4.486739000 | -1.164363000 |
| C | -4.892897000 | 4.781582000 | -1.205527000 |
| C | -3.977317000 | 3.745195000 | -0.985028000 |
| O | -4.367906000 | 6.001319000 | -1.433749000 |
| C | -5.250771000 | 7.118785000 | -1.646039000 |
| C | -4.414130000 | 8.389870000 | -1.852139000 |
| C | -5.403494000 | 9.550701000 | -2.073955000 |

| | | | |
|---|---|---|---|
| C | -3.510388000 | 8.233916000 | -3.089791000 |
| C | -3.558285000 | 8.668502000 | -0.601950000 |
| H | -3.196421000 | -3.719183000 | 0.978128000 |
| H | -7.358077000 | -4.651536000 | 1.541720000 |
| H | -7.868818000 | -2.277818000 | 1.010399000 |
| H | -3.056016000 | -5.632884000 | 2.280149000 |
| H | -3.213065000 | -5.995495000 | 0.541265000 |
| H | -1.761210000 | -8.012996000 | 0.818496000 |
| H | -2.164516000 | -9.239391000 | 2.031371000 |
| H | -1.590276000 | -7.644987000 | 2.547290000 |
| H | -3.671043000 | -7.397010000 | 4.018691000 |
| H | -4.256728000 | -8.984395000 | 3.490029000 |
| H | -5.291329000 | -7.554880000 | 3.313226000 |
| H | -4.090970000 | -8.273784000 | -0.216863000 |
| H | -5.540957000 | -8.094391000 | 0.789352000 |
| H | -4.488658000 | -9.506988000 | 0.992840000 |
| H | -7.685705000 | 2.927409000 | -0.784496000 |
| H | -7.029154000 | 5.232228000 | -1.333421000 |
| H | -2.926082000 | 3.995304000 | -0.975957000 |
| H | -5.906714000 | 7.227699000 | -0.772642000 |
| H | -5.872509000 | 6.920976000 | -2.528896000 |
| H | -6.032353000 | 9.378635000 | -2.956037000 |
| H | -4.857566000 | 10.487981000 | -2.230106000 |
| H | -6.062466000 | 9.687623000 | -1.207829000 |
| H | -4.105295000 | 8.046045000 | -3.992204000 |
| H | -2.808844000 | 7.402665000 | -2.966363000 |
| H | -2.928767000 | 9.148211000 | -3.255071000 |
| H | -4.187568000 | 8.789688000 | 0.288529000 |
| H | -2.980570000 | 9.590719000 | -0.733106000 |
| H | -2.855262000 | 7.851139000 | -0.412029000 |

Cartesian coordinates of DFT-optimized structure of **15** complex by B3LYP/Def2-TZVP; 6-31G(d,p)/$CH_3CN$, charge = 2, multiplicity = 1

| | | | |
|---|---|---|---|
| Os | 4.796470000 | -0.234276000 | -0.206483000 |
| N | 5.342060000 | 0.680094000 | 1.603206000 |
| N | 6.839380000 | -0.332534000 | -0.270652000 |
| N | 5.138489000 | -1.191346000 | -2.043950000 |
| C | 4.515227000 | 1.191314000 | 2.543550000 |
| H | 3.456993000 | 1.108217000 | 2.328303000 |
| C | 4.973010000 | 1.786866000 | 3.711120000 |
| H | 4.257411000 | 2.178145000 | 4.425875000 |
| C | 6.348385000 | 1.868414000 | 3.935873000 |
| H | 6.740412000 | 2.327674000 | 4.837187000 |
| C | 7.213213000 | 1.347942000 | 2.978937000 |
| H | 8.285316000 | 1.398635000 | 3.128858000 |
| C | 6.704276000 | 0.757317000 | 1.819576000 |
| C | 7.555097000 | 0.183592000 | 0.763816000 |
| C | 8.950160000 | 0.127105000 | 0.739115000 |
| H | 9.538770000 | 0.532091000 | 1.553964000 |

| | | | |
|---|---|---|---|
| C | 9.583656000 | -0.463728000 | -0.356568000 |
| C | 8.829679000 | -0.988218000 | -1.408797000 |
| H | 9.324976000 | -1.445324000 | -2.257469000 |
| C | 7.436757000 | -0.911531000 | -1.346001000 |
| C | 6.470829000 | -1.397826000 | -2.344844000 |
| C | 6.847826000 | -2.029892000 | -3.532437000 |
| H | 7.898758000 | -2.182396000 | -3.748453000 |
| C | 5.879334000 | -2.461331000 | -4.432639000 |
| H | 6.168662000 | -2.952416000 | -5.355727000 |
| C | 4.534797000 | -2.248866000 | -4.122919000 |
| H | 3.741947000 | -2.566340000 | -4.791240000 |
| C | 4.209677000 | -1.616778000 | -2.930381000 |
| H | 3.179694000 | -1.432561000 | -2.650648000 |
| C | -3.559943000 | 1.275603000 | -0.464807000 |
| C | 2.058014000 | -1.176480000 | 0.430978000 |
| C | -4.291258000 | 0.175938000 | 0.034234000 |
| C | 2.797906000 | -0.139039000 | -0.145256000 |
| C | -3.635740000 | -0.984918000 | 0.500399000 |
| C | 2.125306000 | 0.963779000 | -0.681528000 |
| C | -2.217715000 | -0.991502000 | 0.617013000 |
| C | 0.716220000 | 1.016283000 | -0.760533000 |
| C | -1.464559000 | 0.052227000 | -0.033526000 |
| C | -0.027584000 | -0.010912000 | -0.069296000 |
| C | -2.154430000 | 1.160025000 | -0.647965000 |
| C | 0.656238000 | -1.101417000 | 0.585764000 |
| C | -1.382463000 | 2.043786000 | -1.467315000 |
| C | -0.123820000 | -1.988310000 | 1.392397000 |
| H | -1.891604000 | 2.751069000 | -2.110055000 |
| H | 0.370251000 | -2.679364000 | 2.063000000 |
| C | -0.022069000 | 1.974230000 | -1.524431000 |
| C | -1.486314000 | -1.936761000 | 1.404325000 |
| H | 0.497308000 | 2.624841000 | -2.216033000 |
| H | -2.022038000 | -2.592223000 | 2.079790000 |
| H | 10.666515000 | -0.515573000 | -0.390375000 |
| Ru | -6.263356000 | 0.258545000 | 0.071914000 |
| C | -6.077959000 | 1.855398000 | 4.172958000 |
| C | -5.731526000 | 1.354976000 | 2.922882000 |
| N | -6.643014000 | 1.016795000 | 1.988551000 |
| C | -7.977168000 | 1.175470000 | 2.287722000 |
| C | -8.381014000 | 1.674550000 | 3.528992000 |
| C | -7.428339000 | 2.018548000 | 4.483227000 |
| C | -8.923296000 | 0.786614000 | 1.223373000 |
| N | -8.303617000 | 0.333945000 | 0.106375000 |
| C | -8.991991000 | -0.075202000 | -0.986820000 |
| C | -10.389068000 | -0.038740000 | -0.984926000 |
| C | -11.047794000 | 0.424365000 | 0.156400000 |
| C | -10.318848000 | 0.841963000 | 1.272364000 |
| C | -8.113399000 | -0.525500000 | -2.084107000 |
| N | -6.761733000 | -0.463608000 | -1.831646000 |
| C | -5.909899000 | -0.860958000 | -2.798386000 |
| C | -6.334590000 | -1.329054000 | -4.036797000 |

| | | | |
|---|---|---|---|
| C | -7.703187000 | -1.394083000 | -4.299884000 |
| C | -8.594923000 | -0.987730000 | -3.311876000 |
| H | -4.695890000 | 1.211856000 | 2.638361000 |
| H | -5.298127000 | 2.109882000 | 4.882349000 |
| H | -7.735799000 | 2.406261000 | 5.448845000 |
| H | -9.436361000 | 1.793146000 | 3.745452000 |
| H | -10.834637000 | 1.200304000 | 2.155631000 |
| H | -12.131788000 | 0.459916000 | 0.176241000 |
| H | -10.958823000 | -0.361967000 | -1.848403000 |
| H | -9.662953000 | -1.028484000 | -3.491596000 |
| H | -8.071020000 | -1.753759000 | -5.255189000 |
| H | -5.600144000 | -1.634763000 | -4.773881000 |
| H | -4.857501000 | -0.792914000 | -2.549886000 |
| N | 2.905861000 | -2.250295000 | 0.841311000 |
| C | 2.685934000 | -3.530291000 | 1.261381000 |
| C | 3.910657000 | -4.146128000 | 1.432408000 |
| C | 4.867278000 | -3.182687000 | 1.076303000 |
| N | 4.273012000 | -2.042537000 | 0.707347000 |
| N | 3.044510000 | 1.955951000 | -1.141285000 |
| N | 4.391776000 | 1.620164000 | -1.089616000 |
| C | 5.067095000 | 2.702021000 | -1.493203000 |
| C | 4.185418000 | 3.753057000 | -1.790399000 |
| C | 2.920755000 | 3.253598000 | -1.546236000 |
| H | 1.692217000 | -3.925664000 | 1.376227000 |
| H | 4.086001000 | -5.159111000 | 1.761701000 |
| H | 5.944988000 | -3.253231000 | 1.053618000 |
| H | 1.963275000 | 3.741026000 | -1.599938000 |
| H | 4.435057000 | 4.747293000 | -2.128782000 |
| H | 6.146120000 | 2.671391000 | -1.536557000 |
| C | -4.578582000 | -2.070955000 | 0.815511000 |
| C | -4.241701000 | -3.415632000 | 1.042013000 |
| C | -5.237014000 | -4.361128000 | 1.307200000 |
| C | -6.576965000 | -3.939748000 | 1.330214000 |
| C | -6.851658000 | -2.616664000 | 1.045834000 |
| N | -5.905151000 | -1.693299000 | 0.777824000 |
| O | -5.015346000 | -5.669793000 | 1.539320000 |
| C | -3.668441000 | -6.175776000 | 1.519348000 |
| C | -3.690525000 | -7.679226000 | 1.831922000 |
| C | -2.230961000 | -8.172132000 | 1.789100000 |
| C | -4.278529000 | -7.917844000 | 3.235665000 |
| C | -4.522849000 | -8.428173000 | 0.773881000 |
| C | -4.417175000 | 2.440915000 | -0.740636000 |
| N | -5.775567000 | 2.179085000 | -0.645535000 |
| C | -6.643618000 | 3.179090000 | -0.868224000 |
| C | -6.275382000 | 4.485027000 | -1.164543000 |
| C | -4.906281000 | 4.782703000 | -1.200316000 |
| C | -3.987707000 | 3.747432000 | -0.980158000 |
| O | -4.382788000 | 6.004110000 | -1.423696000 |
| C | -5.267291000 | 7.119909000 | -1.637022000 |
| C | -4.432815000 | 8.393068000 | -1.839131000 |
| C | -5.423940000 | 9.552154000 | -2.062141000 |

| | | | |
|---|---|---|---|
| C | -3.525363000 | 8.240662000 | -3.074496000 |
| C | -3.580945000 | 8.671815000 | -0.586245000 |
| H | -3.209481000 | -3.719993000 | 0.971099000 |
| H | -7.372934000 | -4.644892000 | 1.539352000 |
| H | -7.876764000 | -2.264149000 | 1.018803000 |
| H | -3.071749000 | -5.636823000 | 2.266974000 |
| H | -3.232847000 | -5.996001000 | 0.527783000 |
| H | -1.784210000 | -8.016580000 | 0.799518000 |
| H | -2.188063000 | -9.244409000 | 2.010766000 |
| H | -1.610050000 | -7.652007000 | 2.528702000 |
| H | -3.687931000 | -7.402805000 | 4.003589000 |
| H | -4.277797000 | -8.987971000 | 3.472970000 |
| H | -5.309630000 | -7.555911000 | 3.300365000 |
| H | -4.115904000 | -8.271320000 | -0.232827000 |
| H | -5.564119000 | -8.090435000 | 0.775641000 |
| H | -4.514614000 | -9.505644000 | 0.975395000 |
| H | -7.693717000 | 2.918091000 | -0.793719000 |
| H | -7.042222000 | 5.229600000 | -1.332847000 |
| H | -2.936896000 | 3.999823000 | -0.967281000 |
| H | -5.926067000 | 7.226306000 | -0.765419000 |
| H | -5.886188000 | 6.921963000 | -2.521856000 |
| H | -6.050092000 | 9.379878000 | -2.946116000 |
| H | -4.879558000 | 10.490760000 | -2.215701000 |
| H | -6.085553000 | 9.686645000 | -1.197649000 |
| H | -4.117427000 | 8.053158000 | -3.978863000 |
| H | -2.822729000 | 7.410434000 | -2.950408000 |
| H | -2.944882000 | 9.156229000 | -3.236746000 |
| H | -4.212899000 | 8.790716000 | 0.302655000 |
| H | -3.004651000 | 9.595318000 | -0.714621000 |
| H | -2.876905000 | 7.855557000 | -0.395356000 |

Cartesian coordinates of DFT-optimized structure of **16** complex by B3LYP/Def2-TZVP; 6-31G(d,p)/$CH_3CN$, charge = 2, multiplicity = 1

| | | | |
|---|---|---|---|
| Os | 5.643674000 | -0.955395000 | 0.034536000 |
| N | 5.170269000 | 1.076201000 | -0.271282000 |
| N | 6.094360000 | -1.301404000 | -1.985134000 |
| N | 7.690504000 | -0.952141000 | 0.036693000 |
| N | 6.087409000 | -0.596651000 | 2.052949000 |
| N | -5.084343000 | 0.803343000 | -0.251643000 |
| C | 5.214800000 | -1.468035000 | -2.999954000 |
| H | 4.169309000 | -1.414849000 | -2.722416000 |
| C | 5.607050000 | -1.693061000 | -4.312062000 |
| H | 4.851681000 | -1.819295000 | -5.079903000 |
| C | 6.969686000 | -1.752856000 | -4.611823000 |
| H | 7.310674000 | -1.927201000 | -5.626839000 |
| C | 7.887648000 | -1.583964000 | -3.581156000 |
| H | 8.950991000 | -1.625562000 | -3.786613000 |
| C | 7.444253000 | -1.359103000 | -2.274981000 |
| C | 8.352178000 | -1.167388000 | -1.133191000 |

| | | | |
|---|---|---|---|
| C | 9.748312000 | -1.187942000 | -1.148356000 |
| H | 10.292414000 | -1.359825000 | -2.069752000 |
| C | 10.441485000 | -0.983757000 | 0.047014000 |
| C | 9.744287000 | -0.763384000 | 1.237171000 |
| H | 10.285331000 | -0.606897000 | 2.163104000 |
| C | 8.348209000 | -0.750918000 | 1.211402000 |
| C | 7.436593000 | -0.542046000 | 2.347359000 |
| C | 7.875871000 | -0.301692000 | 3.652087000 |
| H | 8.938587000 | -0.262090000 | 3.861108000 |
| C | 6.954648000 | -0.112034000 | 4.676308000 |
| H | 7.292524000 | 0.074631000 | 5.690173000 |
| C | 5.592951000 | -0.165477000 | 4.371293000 |
| H | 4.835245000 | -0.022022000 | 5.133790000 |
| C | 5.204652000 | -0.406969000 | 3.060865000 |
| H | 4.159870000 | -0.454766000 | 2.779576000 |
| C | 6.070941000 | 2.084026000 | -0.341593000 |
| H | 7.112024000 | 1.783577000 | -0.366137000 |
| C | 5.729292000 | 3.419386000 | -0.370865000 |
| H | 6.489952000 | 4.189110000 | -0.427891000 |
| C | 4.368818000 | 3.763009000 | -0.289789000 |
| C | 3.422385000 | 2.736980000 | -0.228042000 |
| H | 2.376625000 | 2.972593000 | -0.114333000 |
| C | 3.818889000 | 1.390226000 | -0.260474000 |
| C | -2.704824000 | -2.326740000 | 0.255901000 |
| C | 2.925526000 | 0.223867000 | -0.189048000 |
| C | -3.451261000 | -1.161685000 | 0.035249000 |
| C | 3.646539000 | -0.968560000 | 0.030683000 |
| C | -2.798596000 | 0.065105000 | -0.177371000 |
| C | 2.961737000 | -2.172871000 | 0.262133000 |
| C | -3.750704000 | 1.189014000 | -0.233850000 |
| C | -3.432882000 | 2.546545000 | -0.178799000 |
| H | -2.409183000 | 2.874818000 | -0.070621000 |
| C | -4.433039000 | 3.527799000 | -0.218030000 |
| C | -5.770904000 | 3.119577000 | -0.301936000 |
| H | -6.595948000 | 3.818208000 | -0.344463000 |
| C | -6.030419000 | 1.755097000 | -0.297973000 |
| H | -7.055291000 | 1.401010000 | -0.322130000 |
| C | -1.377927000 | 0.096963000 | -0.308457000 |
| C | 1.563390000 | -2.220954000 | 0.408788000 |
| C | -0.622326000 | -1.075083000 | 0.051086000 |
| C | 0.815778000 | -1.035030000 | 0.050559000 |
| C | -1.304573000 | -2.299358000 | 0.405669000 |
| C | 1.504591000 | 0.176136000 | -0.314943000 |
| C | -0.521926000 | -3.377853000 | 0.931671000 |
| C | 0.714124000 | 1.233866000 | -0.862198000 |
| H | -1.016002000 | -4.220340000 | 1.399168000 |
| H | 1.204068000 | 2.063371000 | -1.356207000 |
| C | 0.838835000 | -3.340151000 | 0.934357000 |
| C | -0.651011000 | 1.198101000 | -0.856050000 |
| H | 1.376139000 | -4.153712000 | 1.405092000 |
| H | -1.190387000 | 2.002685000 | -1.340056000 |

| | | | |
|---|---|---|---|
| O | 4.082644000 | 5.079130000 | -0.270238000 |
| O | -4.012324000 | 4.806311000 | -0.162185000 |
| C | -4.983830000 | 5.868880000 | -0.187425000 |
| C | -4.257211000 | 7.218153000 | -0.089111000 |
| C | -5.338033000 | 8.316471000 | -0.121411000 |
| C | -3.302236000 | 7.394174000 | -1.284902000 |
| C | -3.471734000 | 7.301304000 | 1.233492000 |
| C | 2.710389000 | 5.505789000 | -0.190624000 |
| C | 2.656676000 | 7.040088000 | -0.227243000 |
| C | 1.172400000 | 7.443622000 | -0.129249000 |
| C | 3.434010000 | 7.626479000 | 0.966444000 |
| C | 3.249283000 | 7.557345000 | -1.551749000 |
| H | -5.675251000 | 5.742229000 | 0.655681000 |
| H | -5.556032000 | 5.807815000 | -1.122272000 |
| H | -6.034939000 | 8.218861000 | 0.719980000 |
| H | -5.919476000 | 8.279571000 | -1.050746000 |
| H | -4.872787000 | 9.306560000 | -0.057052000 |
| H | -3.846864000 | 7.347237000 | -2.236078000 |
| H | -2.532136000 | 6.616017000 | -1.295754000 |
| H | -2.801197000 | 8.367676000 | -1.232322000 |
| H | -4.138381000 | 7.185399000 | 2.097087000 |
| H | -2.974809000 | 8.274364000 | 1.321121000 |
| H | -2.705008000 | 6.521859000 | 1.289751000 |
| H | 2.154234000 | 5.082148000 | -1.037087000 |
| H | 2.271388000 | 5.127768000 | 0.741892000 |
| H | 1.075552000 | 8.534820000 | -0.155717000 |
| H | 0.721848000 | 7.089948000 | 0.806159000 |
| H | 0.589231000 | 7.035282000 | -0.963790000 |
| H | 3.021772000 | 7.271733000 | 1.919240000 |
| H | 3.372364000 | 8.720912000 | 0.960489000 |
| H | 4.491406000 | 7.345778000 | 0.928225000 |
| H | 2.696748000 | 7.160866000 | -2.412723000 |
| H | 4.298890000 | 7.264929000 | -1.657505000 |
| H | 3.194878000 | 8.651359000 | -1.592536000 |
| H | 11.526005000 | -0.997204000 | 0.051241000 |
| N | 3.857303000 | -3.280306000 | 0.364943000 |
| C | 3.697253000 | -4.635731000 | 0.361797000 |
| C | 4.950231000 | -5.218263000 | 0.380848000 |
| C | 5.859982000 | -4.149310000 | 0.372900000 |
| N | 5.210711000 | -2.979613000 | 0.350141000 |
| N | -3.535720000 | -3.484924000 | 0.343712000 |
| C | -3.296611000 | -4.828297000 | 0.319981000 |
| C | -4.515030000 | -5.480027000 | 0.327142000 |
| C | -5.481600000 | -4.459762000 | 0.332839000 |
| N | -4.899206000 | -3.258045000 | 0.329979000 |
| H | 6.940227000 | -4.159702000 | 0.369228000 |
| H | 5.173734000 | -6.274370000 | 0.389573000 |
| H | 2.723191000 | -5.089779000 | 0.311149000 |
| H | -2.297922000 | -5.225706000 | 0.267015000 |
| H | -4.680196000 | -6.546885000 | 0.319791000 |
| H | -6.559763000 | -4.532422000 | 0.326867000 |

| | | | |
|---|---|---|---|
| Ru | -5.427463000 | -1.256557000 | 0.026537000 |
| C | -5.286314000 | -1.906534000 | -4.331130000 |
| C | -4.925140000 | -1.682204000 | -3.007067000 |
| N | -5.823310000 | -1.593214000 | -2.006191000 |
| C | -7.158422000 | -1.728966000 | -2.309927000 |
| C | -7.577207000 | -1.955601000 | -3.623453000 |
| C | -6.637834000 | -2.046577000 | -4.646412000 |
| C | -8.091241000 | -1.616552000 | -1.170740000 |
| C | -9.483580000 | -1.732370000 | -1.206435000 |
| C | -10.199476000 | -1.596628000 | -0.014978000 |
| C | -9.531722000 | -1.350284000 | 1.186770000 |
| C | -8.138505000 | -1.242615000 | 1.171601000 |
| N | -7.463942000 | -1.377438000 | 0.005163000 |
| C | -7.251231000 | -0.991422000 | 2.325220000 |
| N | -5.904411000 | -0.952649000 | 2.046471000 |
| C | -5.045144000 | -0.723967000 | 3.059212000 |
| C | -5.457819000 | -0.527761000 | 4.372671000 |
| C | -6.821473000 | -0.567216000 | 4.663210000 |
| C | -7.721098000 | -0.800605000 | 3.627076000 |
| H | -4.516620000 | -1.969239000 | -5.092540000 |
| H | -3.888021000 | -1.566966000 | -2.715403000 |
| H | -8.633455000 | -2.059377000 | -3.842776000 |
| H | -6.956779000 | -2.222405000 | -5.668476000 |
| H | -10.005993000 | -1.924622000 | -2.136355000 |
| H | -11.280695000 | -1.683802000 | -0.022811000 |
| H | -10.091950000 | -1.247502000 | 2.108807000 |
| H | -3.996588000 | -0.701011000 | 2.786883000 |
| H | -4.718044000 | -0.348011000 | 5.144993000 |
| H | -7.180220000 | -0.418470000 | 5.676339000 |
| H | -8.785749000 | -0.833341000 | 3.826957000 |

Cartesian coordinates of DFT-optimized structure of **17** complex by B3LYP/Def2-TZVP; 6-31G(d,p)/$CH_3CN$, charge = 2, multiplicity = 1

| | | | |
|---|---|---|---|
| Os | 5.473418000 | 0.860289000 | -0.025549000 |
| N | 5.362290000 | -1.223937000 | 0.276579000 |
| N | 5.806119000 | 1.278220000 | 2.003647000 |
| N | 7.487105000 | 1.217775000 | 0.019797000 |
| N | 6.020411000 | 0.590834000 | -2.032951000 |
| C | 4.888526000 | 1.283222000 | 2.998176000 |
| H | 3.876500000 | 1.040542000 | 2.698354000 |
| C | 5.204196000 | 1.575833000 | 4.317633000 |
| H | 4.421294000 | 1.564254000 | 5.067991000 |
| C | 6.526716000 | 1.879797000 | 4.647261000 |
| H | 6.807750000 | 2.113752000 | 5.668666000 |
| C | 7.483529000 | 1.875814000 | 3.638320000 |
| H | 8.517577000 | 2.105643000 | 3.867307000 |
| C | 7.117622000 | 1.574326000 | 2.323790000 |
| C | 8.072431000 | 1.544368000 | 1.204670000 |
| C | 9.443325000 | 1.805626000 | 1.253610000 |

| | | | |
|---|---|---|---|
| H | 9.927034000 | 2.067378000 | 2.187542000 |
| C | 10.190624000 | 1.724935000 | 0.076207000 |
| C | 9.571467000 | 1.388213000 | -1.129801000 |
| H | 10.154027000 | 1.327953000 | -2.041737000 |
| C | 8.198433000 | 1.134829000 | -1.137894000 |
| C | 7.364599000 | 0.774781000 | -2.295396000 |
| C | 7.869027000 | 0.623195000 | -3.589907000 |
| H | 8.926482000 | 0.773068000 | -3.773896000 |
| C | 7.018701000 | 0.281626000 | -4.635886000 |
| H | 7.406977000 | 0.163194000 | -5.641941000 |
| C | 5.661617000 | 0.095943000 | -4.363323000 |
| H | 4.958069000 | -0.171369000 | -5.144119000 |
| C | 5.207187000 | 0.258310000 | -3.062073000 |
| H | 4.163307000 | 0.125207000 | -2.805912000 |
| C | 6.424603000 | -2.059937000 | 0.343914000 |
| H | 7.397384000 | -1.582959000 | 0.372984000 |
| C | 6.320749000 | -3.434705000 | 0.364132000 |
| H | 7.204041000 | -4.060262000 | 0.417356000 |
| C | 5.041202000 | -4.009871000 | 0.278764000 |
| C | 3.930616000 | -3.163457000 | 0.223886000 |
| H | 2.941903000 | -3.577244000 | 0.108244000 |
| C | 4.086938000 | -1.768841000 | 0.262225000 |
| C | -3.016168000 | 0.770669000 | -0.215250000 |
| C | 3.003096000 | -0.775938000 | 0.187466000 |
| C | -3.517258000 | -0.524120000 | 0.023964000 |
| C | 3.505299000 | 0.522654000 | -0.049697000 |
| C | -2.637843000 | -1.586479000 | 0.256447000 |
| C | 2.621418000 | 1.585210000 | -0.287553000 |
| C | -1.246043000 | -1.392344000 | 0.383001000 |
| C | 1.232128000 | 1.388593000 | -0.417369000 |
| C | -0.718703000 | -0.104272000 | -0.004936000 |
| C | 0.705770000 | 0.098648000 | -0.030390000 |
| C | -1.612952000 | 0.967053000 | -0.364650000 |
| C | 1.599383000 | -0.972548000 | 0.331445000 |
| C | -1.029506000 | 2.145538000 | -0.930684000 |
| C | 1.014293000 | -2.150473000 | 0.897012000 |
| H | -1.668386000 | 2.874793000 | -1.412468000 |
| H | 1.651578000 | -2.879633000 | 1.380785000 |
| C | 0.318112000 | 2.350375000 | -0.951820000 |
| C | -0.333688000 | -2.354949000 | 0.916694000 |
| H | 0.698439000 | 3.233526000 | -1.449422000 |
| H | -0.715316000 | -3.238723000 | 1.412459000 |
| O | 4.987775000 | -5.355222000 | 0.246858000 |
| C | 3.709944000 | -6.012863000 | 0.164543000 |
| C | 3.925640000 | -7.533132000 | 0.151416000 |
| C | 2.532270000 | -8.186441000 | 0.065344000 |
| C | 4.766022000 | -7.936990000 | -1.074865000 |
| C | 4.629034000 | -7.978713000 | 1.447399000 |
| H | 3.099938000 | -5.718001000 | 1.028649000 |
| H | 3.199099000 | -5.690506000 | -0.752052000 |
| H | 2.627163000 | -9.278046000 | 0.051737000 |

| | | | |
|---|---|---|---|
| H | 2.003203000 | -7.884617000 | -0.846898000 |
| H | 1.908667000 | -7.915376000 | 0.926048000 |
| H | 4.273833000 | -7.635388000 | -2.007887000 |
| H | 4.901814000 | -9.024292000 | -1.103007000 |
| H | 5.756388000 | -7.471326000 | -1.047423000 |
| H | 4.037485000 | -7.707264000 | 2.330548000 |
| H | 5.615735000 | -7.514418000 | 1.543654000 |
| H | 4.763281000 | -9.066568000 | 1.454293000 |
| H | 11.256364000 | 1.925182000 | 0.098397000 |
| N | 3.308856000 | 2.831254000 | -0.402873000 |
| C | 2.913505000 | 4.137140000 | -0.435826000 |
| C | 4.045171000 | 4.930307000 | -0.452734000 |
| C | 5.128115000 | 4.038597000 | -0.407385000 |
| N | 4.693632000 | 2.773652000 | -0.364555000 |
| N | -3.323646000 | -2.833804000 | 0.372600000 |
| N | -4.704296000 | -2.772137000 | 0.355064000 |
| C | -5.139341000 | -4.034288000 | 0.389470000 |
| C | -4.057892000 | -4.931474000 | 0.409277000 |
| C | -2.925807000 | -4.139140000 | 0.384822000 |
| C | -4.099659000 | 1.769481000 | -0.282106000 |
| C | -3.939022000 | 3.163004000 | -0.233897000 |
| C | -5.051897000 | 4.008958000 | -0.280437000 |
| C | -6.330269000 | 3.433723000 | -0.367288000 |
| C | -6.430488000 | 2.056351000 | -0.357056000 |
| N | -5.369005000 | 1.225449000 | -0.299462000 |
| O | -4.998762000 | 5.354110000 | -0.239886000 |
| C | -3.721542000 | 6.012262000 | -0.151740000 |
| C | -3.938752000 | 7.532219000 | -0.128509000 |
| C | -2.546179000 | 8.186354000 | -0.036244000 |
| C | -4.640881000 | 7.985905000 | -1.422383000 |
| C | -4.781156000 | 7.926979000 | 1.099345000 |
| H | -6.201447000 | -4.233899000 | 0.385836000 |
| H | -4.094300000 | -6.010260000 | 0.429864000 |
| H | -1.886685000 | -4.415528000 | 0.342764000 |
| H | -2.950034000 | 3.576623000 | -0.118449000 |
| H | -7.214171000 | 4.059090000 | -0.413576000 |
| H | -7.403237000 | 1.578066000 | -0.386574000 |
| H | -3.110249000 | 5.724209000 | -1.017161000 |
| H | -3.211372000 | 5.684082000 | 0.763145000 |
| H | -2.018016000 | 7.878979000 | 0.874657000 |
| H | -2.642177000 | 9.277753000 | -0.015532000 |
| H | -1.921202000 | 7.921627000 | -0.897922000 |
| H | -4.047810000 | 7.721157000 | -2.306555000 |
| H | -4.776274000 | 9.073648000 | -1.422020000 |
| H | -5.626971000 | 7.521273000 | -1.523203000 |
| H | -4.289831000 | 7.619696000 | 2.030970000 |
| H | -5.770971000 | 7.460439000 | 1.067506000 |
| H | -4.918142000 | 9.013929000 | 1.134540000 |
| Ru | -5.467051000 | -0.854580000 | 0.014345000 |
| C | -5.597545000 | -0.067003000 | 4.348098000 |
| C | -5.160288000 | -0.233915000 | 3.038626000 |

| | | | |
|---|---|---|---|
| N | -5.984276000 | -0.573134000 | 2.027529000 |
| C | -7.318811000 | -0.761471000 | 2.304031000 |
| C | -7.812075000 | -0.608086000 | 3.602242000 |
| C | -6.948872000 | -0.257936000 | 4.636527000 |
| C | -8.166704000 | -1.130359000 | 1.152705000 |
| N | -7.475265000 | -1.212508000 | -0.008750000 |
| C | -8.065836000 | -1.543878000 | -1.181429000 |
| C | -9.436847000 | -1.812597000 | -1.219040000 |
| C | -10.169903000 | -1.731912000 | -0.032952000 |
| C | -9.539773000 | -1.389688000 | 1.165626000 |
| C | -7.119991000 | -1.574895000 | -2.315109000 |
| N | -5.811169000 | -1.277465000 | -2.011649000 |
| C | -4.903989000 | -1.283497000 | -3.008365000 |
| C | -5.229701000 | -1.580794000 | -4.327275000 |
| C | -6.553691000 | -1.886715000 | -4.641711000 |
| C | -7.502445000 | -1.881173000 | -3.623407000 |
| H | -4.119836000 | -0.097420000 | 2.768462000 |
| H | -4.885892000 | 0.206750000 | 5.119247000 |
| H | -7.325757000 | -0.137210000 | 5.646793000 |
| H | -8.866343000 | -0.761787000 | 3.800746000 |
| H | -10.112722000 | -1.330299000 | 2.083652000 |
| H | -11.235037000 | -1.937168000 | -0.042477000 |
| H | -9.930193000 | -2.079530000 | -2.146389000 |
| H | -8.538182000 | -2.112940000 | -3.842607000 |
| H | -6.844730000 | -2.124059000 | -5.659682000 |
| H | -4.454246000 | -1.570535000 | -5.085331000 |
| H | -3.889127000 | -1.038623000 | -2.718294000 |
| H | 4.079945000 | 6.008818000 | -0.484444000 |
| H | 1.874246000 | 4.414097000 | -0.410998000 |
| H | 6.189636000 | 4.238771000 | -0.390981000 |

Cartesian coordinates of DFT-optimized structure of **12ox** complex by B3LYP/Def2-TZVP; 6-31G(d,p)/$CH_3CN$, charge = 3, multiplicity = 2

| | | | |
|---|---|---|---|
| Ru | -5.501257000 | -0.053157000 | 0.014894000 |
| Os | 5.486605000 | 0.095074000 | -0.006564000 |
| N | -5.060105000 | -2.093406000 | 0.295771000 |
| N | 5.060350000 | 2.156334000 | -0.261995000 |
| N | -5.993814000 | 0.242838000 | 2.033441000 |
| N | 5.927031000 | -0.190287000 | -2.043773000 |
| N | -7.553737000 | -0.089140000 | -0.024379000 |
| N | 7.586978000 | 0.115015000 | -0.074786000 |
| N | -5.906076000 | -0.365439000 | -2.022223000 |
| N | 6.050262000 | 0.398174000 | 1.996698000 |
| N | -5.124748000 | 2.002579000 | -0.251933000 |
| N | 5.117624000 | -1.973671000 | 0.275513000 |
| C | -5.133056000 | 0.409180000 | 3.057255000 |
| C | 5.004077000 | -0.345361000 | -3.019739000 |
| H | 3.969208000 | -0.307088000 | -2.705595000 |
| C | -5.548066000 | 0.601390000 | 4.370514000 |

| | | | |
|---|---|---|---|
| C | 5.348948000 | -0.541011000 | -4.350694000 |
| H | 4.564402000 | -0.659028000 | -5.089315000 |
| C | -6.914296000 | 0.624348000 | 4.649012000 |
| C | 6.697224000 | -0.580288000 | -4.703285000 |
| C | -7.814869000 | 0.453880000 | 3.601473000 |
| C | 7.655422000 | -0.420780000 | -3.706986000 |
| H | -8.881508000 | 0.467604000 | 3.792202000 |
| C | -7.342911000 | 0.264256000 | 2.300514000 |
| C | 7.260499000 | -0.225909000 | -2.383280000 |
| C | -8.231017000 | 0.074655000 | 1.135581000 |
| C | 8.205364000 | -0.047132000 | -1.268077000 |
| C | -9.628366000 | 0.049830000 | 1.135701000 |
| C | 9.599895000 | -0.029533000 | -1.339066000 |
| H | -10.191722000 | 0.178812000 | 2.052337000 |
| H | 10.113003000 | -0.155017000 | -2.284744000 |
| C | -10.295730000 | -0.146228000 | -0.075278000 |
| C | 10.329363000 | 0.155706000 | -0.163577000 |
| C | -9.576525000 | -0.314164000 | -1.260458000 |
| C | 9.672691000 | 0.321646000 | 1.056683000 |
| C | -8.180203000 | -0.281482000 | -1.208154000 |
| C | 8.276576000 | 0.298791000 | 1.075552000 |
| C | -7.242645000 | -0.437371000 | -2.338686000 |
| C | 7.401914000 | 0.459248000 | 2.249139000 |
| C | -7.659043000 | -0.644553000 | -3.655730000 |
| C | 7.878655000 | 0.662687000 | 3.544292000 |
| H | -8.716726000 | -0.698623000 | -3.885062000 |
| C | -6.715051000 | -0.781784000 | -4.669364000 |
| C | 6.984465000 | 0.806482000 | 4.600573000 |
| C | -5.361867000 | -0.707170000 | -4.340967000 |
| C | 5.617048000 | 0.742497000 | 4.335953000 |
| H | 4.880222000 | 0.847694000 | 5.124027000 |
| C | -5.002859000 | -0.499025000 | -3.013597000 |
| C | 5.189877000 | 0.538423000 | 3.030303000 |
| H | 4.137696000 | 0.480948000 | 2.783973000 |
| C | -5.957375000 | -3.100200000 | 0.317529000 |
| C | 5.958246000 | 3.163858000 | -0.295477000 |
| H | -7.000852000 | -2.805993000 | 0.321776000 |
| H | 7.001064000 | 2.871402000 | -0.326881000 |
| C | -5.611111000 | -4.437279000 | 0.322067000 |
| C | 5.608911000 | 4.499191000 | -0.282384000 |
| H | -6.369633000 | -5.211098000 | 0.340082000 |
| H | 6.366581000 | 5.273063000 | -0.312431000 |
| C | -4.249186000 | -4.775032000 | 0.265463000 |
| C | 4.248611000 | 4.835550000 | -0.195229000 |
| C | -3.304749000 | -3.742223000 | 0.248117000 |
| C | 3.304191000 | 3.799085000 | -0.164784000 |
| H | -2.255184000 | -3.970393000 | 0.151848000 |
| H | 2.256520000 | 4.024394000 | -0.046126000 |
| C | -3.717093000 | -2.402792000 | 0.304651000 |
| C | 3.714575000 | 2.464484000 | -0.237801000 |
| C | -2.830869000 | -1.224358000 | 0.269771000 |

| | | | |
|---|---|---|---|
| C | 2.820343000 | 1.288234000 | -0.185993000 |
| C | -3.544410000 | -0.021190000 | 0.044609000 |
| C | 3.521775000 | 0.075183000 | 0.036403000 |
| C | -2.865519000 | 1.202861000 | -0.174478000 |
| C | 2.852542000 | -1.153065000 | 0.271502000 |
| C | -3.785478000 | 2.354412000 | -0.230707000 |
| C | 3.771532000 | -2.311439000 | 0.303394000 |
| C | -3.427742000 | 3.700737000 | -0.169153000 |
| C | 3.401662000 | -3.651099000 | 0.262957000 |
| H | -2.395737000 | 4.000864000 | -0.055995000 |
| H | 2.364127000 | -3.942539000 | 0.189611000 |
| C | -4.402379000 | 4.709246000 | -0.208571000 |
| C | 4.369646000 | -4.671161000 | 0.274238000 |
| C | -5.749840000 | 4.334503000 | -0.292583000 |
| C | 5.722143000 | -4.309373000 | 0.309177000 |
| H | -6.556858000 | 5.053910000 | -0.330534000 |
| H | 6.523234000 | -5.035698000 | 0.325694000 |
| C | -6.046231000 | 2.977666000 | -0.294250000 |
| C | 6.032716000 | -2.956995000 | 0.291781000 |
| H | -7.080340000 | 2.652760000 | -0.320910000 |
| H | 7.069713000 | -2.642685000 | 0.282085000 |
| C | -1.458189000 | 1.202201000 | -0.327081000 |
| C | 1.448672000 | -1.143693000 | 0.440558000 |
| C | -0.718239000 | 0.021369000 | 0.054536000 |
| C | 0.712568000 | 0.041826000 | 0.054080000 |
| C | -1.424355000 | -1.180683000 | 0.434099000 |
| C | 1.415097000 | 1.246547000 | -0.336564000 |
| C | -0.654708000 | -2.236790000 | 1.022196000 |
| C | 0.641841000 | 2.305855000 | -0.913502000 |
| H | -1.165509000 | -3.043732000 | 1.532865000 |
| H | 1.148138000 | 3.114724000 | -1.425172000 |
| C | 0.707047000 | -2.221370000 | 1.023762000 |
| C | -0.719525000 | 2.283349000 | -0.908841000 |
| H | 1.237951000 | -3.014252000 | 1.535131000 |
| H | -1.254775000 | 3.078538000 | -1.412441000 |
| O | -3.956583000 | -6.087426000 | 0.223122000 |
| O | 3.903565000 | -5.927597000 | 0.242485000 |
| O | 3.953879000 | 6.142158000 | -0.139215000 |
| O | -3.950298000 | 5.975193000 | -0.152406000 |
| C | -4.898712000 | 7.060472000 | -0.171452000 |
| C | -4.142920000 | 8.394572000 | -0.094977000 |
| C | -5.202765000 | 9.513633000 | -0.108646000 |
| C | -3.212646000 | 8.545707000 | -1.313470000 |
| C | -3.327212000 | 8.468630000 | 1.209643000 |
| C | 2.577829000 | 6.564917000 | -0.038162000 |
| C | 2.523536000 | 8.099226000 | -0.028845000 |
| C | 1.039346000 | 8.495138000 | 0.098285000 |
| C | 3.313346000 | 8.650590000 | 1.173343000 |
| C | 3.100446000 | 8.655805000 | -1.344310000 |
| C | -2.581721000 | -6.509264000 | 0.146831000 |
| C | -2.525903000 | -8.043971000 | 0.146123000 |

| | | | |
|---|---|---|---|
| C | -3.122496000 | -8.593916000 | 1.455612000 |
| C | -3.298182000 | -8.601780000 | -1.064462000 |
| C | -1.040560000 | -8.441695000 | 0.043185000 |
| C | 4.839550000 | -7.027508000 | 0.239550000 |
| C | 4.063708000 | -8.351118000 | 0.198916000 |
| C | 3.208987000 | -8.426480000 | -1.080369000 |
| C | 3.168947000 | -8.478486000 | 1.446365000 |
| C | 5.109969000 | -9.483138000 | 0.191972000 |
| H | -5.579204000 | 6.953244000 | 0.682911000 |
| H | -5.485181000 | 7.004718000 | -1.097442000 |
| H | -5.881871000 | 9.432517000 | 0.748726000 |
| H | -5.805271000 | 9.484089000 | -1.024576000 |
| H | -4.716730000 | 10.494440000 | -0.059240000 |
| H | -3.780935000 | 8.507596000 | -2.251170000 |
| H | -2.459783000 | 7.751024000 | -1.340495000 |
| H | -2.689331000 | 9.508103000 | -1.276731000 |
| H | -3.977699000 | 8.374872000 | 2.088102000 |
| H | -2.806195000 | 9.430486000 | 1.278858000 |
| H | -2.577247000 | 7.672329000 | 1.256200000 |
| H | 2.019729000 | 6.165034000 | -0.894105000 |
| H | 2.149102000 | 6.157695000 | 0.886174000 |
| H | 0.939922000 | 9.586299000 | 0.107578000 |
| H | 0.599612000 | 8.110857000 | 1.026756000 |
| H | 0.448185000 | 8.112668000 | -0.742657000 |
| H | 2.910104000 | 8.269481000 | 2.119700000 |
| H | 3.252426000 | 9.744636000 | 1.198393000 |
| H | 4.370197000 | 8.370788000 | 1.116826000 |
| H | 2.539471000 | 8.283765000 | -2.210633000 |
| H | 4.149630000 | 8.369525000 | -1.470248000 |
| H | 3.044044000 | 9.750262000 | -1.352375000 |
| H | -2.035202000 | -6.105512000 | 1.008955000 |
| H | -2.135725000 | -6.107229000 | -0.772119000 |
| H | -2.573501000 | -8.218413000 | 2.328162000 |
| H | -3.067883000 | -9.688480000 | 1.469972000 |
| H | -4.172783000 | -8.305018000 | 1.564907000 |
| H | -4.355516000 | -8.321131000 | -1.024487000 |
| H | -3.237173000 | -9.696016000 | -1.083409000 |
| H | -2.881409000 | -8.225319000 | -2.006864000 |
| H | -0.461441000 | -8.054413000 | 0.890331000 |
| H | -0.586880000 | -8.063146000 | -0.880969000 |
| H | -0.941090000 | -9.532961000 | 0.041849000 |
| H | 5.455128000 | -6.969529000 | 1.145793000 |
| H | 5.491442000 | -6.933495000 | -0.637889000 |
| H | 2.673390000 | -9.381691000 | -1.124076000 |
| H | 3.834526000 | -8.350442000 | -1.978394000 |
| H | 2.468450000 | -7.620664000 | -1.113179000 |
| H | 2.426325000 | -7.674853000 | 1.487975000 |
| H | 3.765572000 | -8.438125000 | 2.366149000 |
| H | 2.633582000 | -9.434787000 | 1.434867000 |
| H | 5.763937000 | -9.418693000 | -0.686047000 |
| H | 4.610155000 | -10.457835000 | 0.166370000 |

| | | | |
|---|---|---|---|
| H | 5.739647000 | -9.453029000 | 1.089407000 |
| H | -4.807228000 | 0.729677000 | 5.151886000 |
| H | -7.274518000 | 0.771721000 | 5.661734000 |
| H | -4.082120000 | 0.383959000 | 2.795749000 |
| H | -10.099236000 | -0.465947000 | -2.197542000 |
| H | -11.380022000 | -0.168364000 | -0.095344000 |
| H | -7.032158000 | -0.943194000 | -5.694308000 |
| H | -4.588496000 | -0.807713000 | -5.094422000 |
| H | -3.964033000 | -0.433753000 | -2.713586000 |
| H | 6.999671000 | -0.731584000 | -5.733788000 |
| H | 8.709679000 | -0.448108000 | -3.954541000 |
| H | 11.413077000 | 0.171440000 | -0.198900000 |
| H | 10.240952000 | 0.465355000 | 1.967606000 |
| H | 8.946279000 | 0.708496000 | 3.722060000 |
| H | 7.350065000 | 0.964710000 | 5.609315000 |

Cartesian coordinates of DFT-optimized structure of **13ox** complex by B3LYP/Def2-TZVP; 6-31G(d,p)/$CH_3CN$, charge = 3, multiplicity = 2

| | | | |
|---|---|---|---|
| Ru | 4.965342000 | -0.358095000 | -0.208565000 |
| N | 5.465851000 | 0.165107000 | 1.766057000 |
| N | 7.014801000 | -0.457134000 | -0.238225000 |
| N | 5.355851000 | -0.924279000 | -2.196095000 |
| C | 4.613474000 | 0.476207000 | 2.761157000 |
| H | 3.560951000 | 0.447424000 | 2.506293000 |
| C | 5.038033000 | 0.814717000 | 4.041975000 |
| H | 4.303644000 | 1.056477000 | 4.802188000 |
| C | 6.404758000 | 0.833962000 | 4.314663000 |
| H | 6.772770000 | 1.093290000 | 5.301750000 |
| C | 7.296905000 | 0.512811000 | 3.295081000 |
| H | 8.364074000 | 0.519784000 | 3.482875000 |
| C | 6.815064000 | 0.181024000 | 2.027280000 |
| C | 7.695056000 | -0.171966000 | 0.893485000 |
| C | 9.091379000 | -0.231779000 | 0.899553000 |
| H | 9.658822000 | -0.008052000 | 1.795068000 |
| C | 9.751053000 | -0.589457000 | -0.277811000 |
| C | 9.026246000 | -0.880173000 | -1.435146000 |
| H | 9.543368000 | -1.156365000 | -2.346350000 |
| C | 7.631426000 | -0.804997000 | -1.388703000 |
| C | 6.689136000 | -1.069825000 | -2.496149000 |
| C | 7.099948000 | -1.444619000 | -3.776908000 |
| H | 8.155607000 | -1.554153000 | -3.995435000 |
| C | 6.151933000 | -1.676723000 | -4.769887000 |
| H | 6.464574000 | -1.968840000 | -5.766857000 |
| C | 4.801876000 | -1.526197000 | -4.457871000 |
| H | 4.025963000 | -1.694875000 | -5.196251000 |
| C | 4.449080000 | -1.150251000 | -3.165766000 |
| H | 3.412032000 | -1.020481000 | -2.880672000 |
| C | -3.300169000 | 1.215969000 | -0.424194000 |
| C | 2.248799000 | -1.392031000 | 0.161887000 |

| | | | |
|---|---|---|---|
| C | -4.051979000 | 0.078263000 | -0.075183000 |
| C | 3.006387000 | -0.263447000 | -0.180067000 |
| C | -3.403588000 | -1.127302000 | 0.252834000 |
| C | 2.351489000 | 0.933437000 | -0.501250000 |
| C | -2.005932000 | -1.194871000 | 0.364496000 |
| C | 0.951069000 | 1.006186000 | -0.612958000 |
| C | -1.243143000 | -0.057847000 | -0.117201000 |
| C | 0.184158000 | -0.126946000 | -0.138416000 |
| C | -1.906492000 | 1.148256000 | -0.577636000 |
| C | 0.851790000 | -1.329494000 | 0.314875000 |
| C | -1.112753000 | 2.164405000 | -1.200805000 |
| C | 0.061675000 | -2.346157000 | 0.941762000 |
| H | -1.597018000 | 2.971232000 | -1.734394000 |
| H | 0.550284000 | -3.153869000 | 1.470412000 |
| C | 0.244596000 | 2.095218000 | -1.217756000 |
| C | -1.295916000 | -2.283904000 | 0.965164000 |
| H | 0.793040000 | 2.852648000 | -1.761921000 |
| H | -1.839801000 | -3.041030000 | 1.513936000 |
| H | 10.834390000 | -0.641855000 | -0.293482000 |
| N | -4.332835000 | -2.182923000 | 0.473122000 |
| N | -5.677688000 | -1.839297000 | 0.448387000 |
| C | -6.361697000 | -2.976663000 | 0.589641000 |
| C | -5.486553000 | -4.071322000 | 0.687848000 |
| C | -4.217703000 | -3.538054000 | 0.596513000 |
| H | -7.441775000 | -2.953663000 | 0.603305000 |
| H | -5.745730000 | -5.112787000 | 0.800639000 |
| H | -3.260629000 | -4.029505000 | 0.578523000 |
| Os | -6.014495000 | 0.173469000 | -0.045467000 |
| C | -5.894503000 | 1.273472000 | 4.210584000 |
| C | -5.542690000 | 0.919115000 | 2.914202000 |
| N | -6.458985000 | 0.709818000 | 1.944003000 |
| C | -7.792218000 | 0.850713000 | 2.250579000 |
| C | -8.195003000 | 1.204330000 | 3.538159000 |
| C | -7.243023000 | 1.419646000 | 4.530521000 |
| C | -8.730938000 | 0.604595000 | 1.141909000 |
| N | -8.111521000 | 0.275522000 | -0.013601000 |
| C | -8.794674000 | 0.010042000 | -1.149376000 |
| C | -10.189775000 | 0.071337000 | -1.154324000 |
| C | -10.849604000 | 0.409164000 | 0.027888000 |
| C | -10.124588000 | 0.679405000 | 1.189117000 |
| C | -7.918326000 | -0.324651000 | -2.285562000 |
| N | -6.569046000 | -0.314541000 | -2.019684000 |
| C | -5.707243000 | -0.610764000 | -3.016802000 |
| C | -6.130937000 | -0.927470000 | -4.301374000 |
| C | -7.496340000 | -0.941262000 | -4.580153000 |
| C | -8.392387000 | -0.635663000 | -3.559802000 |
| H | -4.506992000 | 0.793748000 | 2.626780000 |
| H | -5.114932000 | 1.428842000 | 4.947478000 |
| H | -7.551188000 | 1.695431000 | 5.533124000 |
| H | -9.249591000 | 1.310480000 | 3.761537000 |
| H | -10.639509000 | 0.940473000 | 2.105510000 |

| | | | |
|---|---|---|---|
| H | -11.932612000 | 0.462020000 | 0.044299000 |
| H | -10.755122000 | -0.137014000 | -2.054350000 |
| H | -9.458575000 | -0.638402000 | -3.751004000 |
| H | -7.860067000 | -1.184397000 | -5.572536000 |
| H | -5.392844000 | -1.156999000 | -5.061255000 |
| H | -4.656037000 | -0.587143000 | -2.761051000 |
| N | -4.129151000 | 2.356846000 | -0.618025000 |
| N | -5.499575000 | 2.144904000 | -0.552265000 |
| C | -6.074304000 | 3.343284000 | -0.674722000 |
| C | -5.100734000 | 4.348276000 | -0.800998000 |
| C | -3.887247000 | 3.694645000 | -0.747021000 |
| H | -2.887024000 | 4.091197000 | -0.758639000 |
| H | -5.261228000 | 5.410075000 | -0.907621000 |
| H | -7.151458000 | 3.424892000 | -0.655819000 |
| N | 3.075719000 | -2.535280000 | 0.360811000 |
| C | 2.834683000 | -3.874141000 | 0.484002000 |
| C | 4.050883000 | -4.522602000 | 0.551235000 |
| C | 5.020324000 | -3.509608000 | 0.438952000 |
| N | 4.442390000 | -2.314837000 | 0.311665000 |
| N | 3.278633000 | 1.991895000 | -0.725885000 |
| N | 4.618534000 | 1.640644000 | -0.716185000 |
| C | 5.305022000 | 2.774275000 | -0.862120000 |
| C | 4.434913000 | 3.876163000 | -0.947741000 |
| C | 3.164333000 | 3.347913000 | -0.844120000 |
| H | 1.835963000 | -4.274927000 | 0.481753000 |
| H | 4.214866000 | -5.584311000 | 0.656481000 |
| H | 6.098106000 | -3.586356000 | 0.430633000 |
| H | 2.209412000 | 3.843146000 | -0.813446000 |
| H | 4.697365000 | 4.917244000 | -1.059386000 |
| H | 6.384965000 | 2.746733000 | -0.885280000 |

Cartesian coordinates of DFT-optimized structure of **14ox** complex by B3LYP/Def2-TZVP; 6-31G(d,p)/$CH_3CN$, charge = 3, multiplicity = 2

| | | | |
|---|---|---|---|
| Ru | 4.757149000 | -0.222118000 | -0.203062000 |
| N | 5.288475000 | 0.688820000 | 1.615488000 |
| N | 6.803758000 | -0.311786000 | -0.257082000 |
| N | 5.111463000 | -1.170654000 | -2.045571000 |
| C | 4.453592000 | 1.189833000 | 2.546091000 |
| H | 3.396494000 | 1.104554000 | 2.324854000 |
| C | 4.900360000 | 1.783682000 | 3.722132000 |
| H | 4.179000000 | 2.169409000 | 4.433916000 |
| C | 6.272205000 | 1.867864000 | 3.954353000 |
| H | 6.657582000 | 2.324552000 | 4.859925000 |
| C | 7.146656000 | 1.353862000 | 3.000649000 |
| H | 8.217347000 | 1.407767000 | 3.158441000 |
| C | 6.642710000 | 0.767630000 | 1.837774000 |
| C | 7.503610000 | 0.199137000 | 0.779786000 |
| C | 8.900326000 | 0.150207000 | 0.767599000 |
| H | 9.483148000 | 0.552256000 | 1.587764000 |

| | | | |
|---|---|---|---|
| C | 9.540435000 | -0.431850000 | -0.328365000 |
| C | 8.795822000 | -0.952944000 | -1.388361000 |
| H | 9.297893000 | -1.402955000 | -2.236592000 |
| C | 7.401438000 | -0.879424000 | -1.327704000 |
| C | 6.440073000 | -1.366457000 | -2.338628000 |
| C | 6.829539000 | -1.988210000 | -3.526733000 |
| H | 7.881701000 | -2.134504000 | -3.740287000 |
| C | 5.864977000 | -2.418417000 | -4.433879000 |
| H | 6.161120000 | -2.901979000 | -5.358865000 |
| C | 4.519798000 | -2.215337000 | -4.130285000 |
| H | 3.731342000 | -2.531912000 | -4.804002000 |
| C | 4.188630000 | -1.591113000 | -2.931963000 |
| H | 3.156041000 | -1.414002000 | -2.656269000 |
| C | -3.543740000 | 1.273289000 | -0.486071000 |
| C | 2.055643000 | -1.181136000 | 0.419240000 |
| C | -4.262524000 | 0.164459000 | 0.025318000 |
| C | 2.793191000 | -0.136719000 | -0.151750000 |
| C | -3.616778000 | -1.004451000 | 0.500854000 |
| C | 2.119544000 | 0.967855000 | -0.687817000 |
| C | -2.206961000 | -1.001847000 | 0.621873000 |
| C | 0.714101000 | 1.009750000 | -0.777897000 |
| C | -1.459024000 | 0.043194000 | -0.046329000 |
| C | -0.029506000 | -0.017063000 | -0.081654000 |
| C | -2.147840000 | 1.150157000 | -0.677870000 |
| C | 0.658005000 | -1.104133000 | 0.579551000 |
| C | -1.378787000 | 2.028352000 | -1.506932000 |
| C | -0.113915000 | -1.986762000 | 1.399391000 |
| H | -1.890012000 | 2.723210000 | -2.160626000 |
| H | 0.388561000 | -2.669385000 | 2.072263000 |
| C | -0.020261000 | 1.958283000 | -1.557902000 |
| C | -1.474487000 | -1.941018000 | 1.416672000 |
| H | 0.504198000 | 2.600398000 | -2.253323000 |
| H | -2.008580000 | -2.587760000 | 2.101011000 |
| H | 10.623747000 | -0.479563000 | -0.356442000 |
| Os | -6.226671000 | 0.250654000 | 0.077216000 |
| C | -6.063731000 | 1.788882000 | 4.186855000 |
| C | -5.728006000 | 1.297970000 | 2.931866000 |
| N | -6.657275000 | 0.986284000 | 2.000739000 |
| C | -7.987162000 | 1.161226000 | 2.307959000 |
| C | -8.373020000 | 1.650340000 | 3.556125000 |
| C | -7.408561000 | 1.968421000 | 4.507499000 |
| C | -8.938324000 | 0.799781000 | 1.243084000 |
| N | -8.328078000 | 0.348798000 | 0.122255000 |
| C | -9.024224000 | -0.037948000 | -0.972008000 |
| C | -10.419439000 | 0.023261000 | -0.969360000 |
| C | -11.067878000 | 0.486653000 | 0.176284000 |
| C | -10.331539000 | 0.879091000 | 1.295156000 |
| C | -8.157526000 | -0.498777000 | -2.069745000 |
| N | -6.805841000 | -0.462616000 | -1.814955000 |
| C | -5.951785000 | -0.881344000 | -2.775759000 |
| C | -6.385785000 | -1.342398000 | -4.011762000 |

| | | | |
|---|---|---|---|
| C | -7.753375000 | -1.379108000 | -4.280243000 |
| C | -8.641038000 | -0.953480000 | -3.296800000 |
| H | -4.696239000 | 1.142950000 | 2.644656000 |
| H | -5.274926000 | 2.021831000 | 4.892876000 |
| H | -7.703677000 | 2.348575000 | 5.479417000 |
| H | -9.424711000 | 1.781470000 | 3.780263000 |
| H | -10.837983000 | 1.237058000 | 2.183162000 |
| H | -12.150691000 | 0.541363000 | 0.197636000 |
| H | -10.993614000 | -0.282453000 | -1.835466000 |
| H | -9.708643000 | -0.974000000 | -3.479023000 |
| H | -8.124380000 | -1.732719000 | -5.236046000 |
| H | -5.653916000 | -1.664272000 | -4.743704000 |
| H | -4.899222000 | -0.835899000 | -2.528817000 |
| N | 2.899346000 | -2.255666000 | 0.823404000 |
| C | 2.676839000 | -3.545579000 | 1.214787000 |
| C | 3.901653000 | -4.157473000 | 1.386052000 |
| C | 4.856796000 | -3.178609000 | 1.056953000 |
| N | 4.262711000 | -2.037826000 | 0.706031000 |
| N | 3.031044000 | 1.966700000 | -1.137202000 |
| N | 4.374959000 | 1.631974000 | -1.088763000 |
| C | 5.046256000 | 2.717622000 | -1.473885000 |
| C | 4.163259000 | 3.775295000 | -1.757641000 |
| C | 2.900681000 | 3.271147000 | -1.521843000 |
| H | 1.683483000 | -3.947839000 | 1.310949000 |
| H | 4.080230000 | -5.175864000 | 1.696199000 |
| H | 5.935118000 | -3.246065000 | 1.043063000 |
| H | 1.941975000 | 3.757545000 | -1.568943000 |
| H | 4.412708000 | 4.774750000 | -2.080262000 |
| H | 6.125724000 | 2.692152000 | -1.516296000 |
| C | -4.563959000 | -2.093910000 | 0.818805000 |
| C | -4.218998000 | -3.430233000 | 1.043771000 |
| C | -5.212249000 | -4.384604000 | 1.308389000 |
| C | -6.553240000 | -3.968831000 | 1.327791000 |
| C | -6.837829000 | -2.648378000 | 1.044813000 |
| N | -5.892994000 | -1.721129000 | 0.780788000 |
| O | -4.982689000 | -5.684572000 | 1.540765000 |
| C | -3.631392000 | -6.190638000 | 1.523782000 |
| C | -3.655408000 | -7.693383000 | 1.836952000 |
| C | -2.195079000 | -8.184767000 | 1.796992000 |
| C | -4.245966000 | -7.931188000 | 3.239762000 |
| C | -4.485138000 | -8.442644000 | 0.777046000 |
| C | -4.405356000 | 2.444234000 | -0.757576000 |
| N | -5.765862000 | 2.191829000 | -0.648055000 |
| C | -6.631153000 | 3.196586000 | -0.862666000 |
| C | -6.253636000 | 4.497566000 | -1.165391000 |
| C | -4.883989000 | 4.785799000 | -1.216994000 |
| C | -3.968000000 | 3.740220000 | -1.002569000 |
| O | -4.352987000 | 5.993837000 | -1.448918000 |
| C | -5.226150000 | 7.124193000 | -1.661880000 |
| C | -4.374665000 | 8.383511000 | -1.874205000 |
| C | -5.352845000 | 9.553918000 | -2.096819000 |

| | | | |
|---|---|---|---|
| C | -3.476841000 | 8.213091000 | -3.114287000 |
| C | -3.512443000 | 8.656418000 | -0.627129000 |
| H | -3.185193000 | -3.728810000 | 0.974121000 |
| H | -7.346240000 | -4.677359000 | 1.534896000 |
| H | -7.864101000 | -2.301838000 | 1.018179000 |
| H | -3.040112000 | -5.649356000 | 2.273172000 |
| H | -3.197697000 | -6.009828000 | 0.532249000 |
| H | -1.746587000 | -8.029621000 | 0.808204000 |
| H | -2.152752000 | -9.256840000 | 2.019119000 |
| H | -1.576036000 | -7.664355000 | 2.537839000 |
| H | -3.657680000 | -7.415024000 | 4.008602000 |
| H | -4.243892000 | -9.001161000 | 3.477120000 |
| H | -5.278044000 | -7.571551000 | 3.302454000 |
| H | -4.076976000 | -8.285045000 | -0.228978000 |
| H | -5.527250000 | -8.107396000 | 0.777501000 |
| H | -4.475126000 | -9.519921000 | 0.978704000 |
| H | -7.681696000 | 2.944112000 | -0.778495000 |
| H | -7.017044000 | 5.246582000 | -1.327726000 |
| H | -2.915707000 | 3.985147000 | -1.001383000 |
| H | -5.875400000 | 7.238984000 | -0.785028000 |
| H | -5.851084000 | 6.926715000 | -2.541781000 |
| H | -5.985476000 | 9.386253000 | -2.976952000 |
| H | -4.796717000 | 10.484373000 | -2.256882000 |
| H | -6.007883000 | 9.700480000 | -1.229379000 |
| H | -4.076401000 | 8.027290000 | -4.013949000 |
| H | -2.782261000 | 7.376082000 | -2.989768000 |
| H | -2.887297000 | 9.121149000 | -3.284877000 |
| H | -4.137528000 | 8.786710000 | 0.264921000 |
| H | -2.926081000 | 9.572274000 | -0.763244000 |
| H | -2.816446000 | 7.832999000 | -0.437099000 |

Cartesian coordinates of DFT-optimized structure of **15ox** complex by B3LYP/Def2-TZVP; 6-31G(d,p)/$CH_3CN$, charge = 3, multiplicity = 2

| | | | |
|---|---|---|---|
| Os | 4.738395000 | -0.227765000 | -0.204972000 |
| N | 5.323604000 | 0.662134000 | 1.613907000 |
| N | 6.831846000 | -0.322156000 | -0.261342000 |
| N | 5.143542000 | -1.162344000 | -2.050333000 |
| C | 4.481411000 | 1.158880000 | 2.546229000 |
| H | 3.425272000 | 1.078191000 | 2.324416000 |
| C | 4.929534000 | 1.741935000 | 3.724876000 |
| H | 4.205836000 | 2.123126000 | 4.436172000 |
| C | 6.300559000 | 1.821925000 | 3.962420000 |
| H | 6.683444000 | 2.271099000 | 4.872369000 |
| C | 7.177018000 | 1.313663000 | 3.008128000 |
| H | 8.247124000 | 1.364237000 | 3.168280000 |
| C | 6.678529000 | 0.736970000 | 1.840040000 |
| C | 7.535039000 | 0.177395000 | 0.779790000 |
| C | 8.930541000 | 0.126047000 | 0.766631000 |
| H | 9.512190000 | 0.519316000 | 1.591328000 |

| | | | |
|---|---|---|---|
| C | 9.569740000 | -0.445676000 | -0.334345000 |
| C | 8.824104000 | -0.955114000 | -1.398302000 |
| H | 9.323390000 | -1.397908000 | -2.251454000 |
| C | 7.430929000 | -0.880939000 | -1.336781000 |
| C | 6.472099000 | -1.358266000 | -2.348467000 |
| C | 6.853590000 | -1.972023000 | -3.541456000 |
| H | 7.904645000 | -2.118038000 | -3.758529000 |
| C | 5.885266000 | -2.394262000 | -4.447662000 |
| H | 6.176978000 | -2.871595000 | -5.376866000 |
| C | 4.541707000 | -2.191337000 | -4.137560000 |
| H | 3.749739000 | -2.501357000 | -4.809612000 |
| C | 4.211478000 | -1.575942000 | -2.936672000 |
| H | 3.180305000 | -1.400245000 | -2.659255000 |
| C | -3.548914000 | 1.276335000 | -0.472839000 |
| C | 2.040927000 | -1.191450000 | 0.426609000 |
| C | -4.281873000 | 0.171714000 | 0.029581000 |
| C | 2.776572000 | -0.139167000 | -0.152637000 |
| C | -3.624410000 | -0.995017000 | 0.496074000 |
| C | 2.107982000 | 0.975363000 | -0.695881000 |
| C | -2.215331000 | -0.996928000 | 0.616179000 |
| C | 0.708023000 | 1.014691000 | -0.782733000 |
| C | -1.460770000 | 0.047319000 | -0.042770000 |
| C | -0.034475000 | -0.015023000 | -0.079431000 |
| C | -2.153581000 | 1.155659000 | -0.663804000 |
| C | 0.649559000 | -1.107127000 | 0.587244000 |
| C | -1.386409000 | 2.034738000 | -1.497172000 |
| C | -0.127049000 | -1.990791000 | 1.404435000 |
| H | -1.902061000 | 2.730812000 | -2.146427000 |
| H | 0.371810000 | -2.670609000 | 2.082626000 |
| C | -0.029641000 | 1.967559000 | -1.557361000 |
| C | -1.485905000 | -1.937980000 | 1.415239000 |
| H | 0.491008000 | 2.606353000 | -2.258396000 |
| H | -2.025467000 | -2.582865000 | 2.097300000 |
| H | 10.652688000 | -0.494549000 | -0.363163000 |
| Ru | -6.230893000 | 0.256040000 | 0.074319000 |
| C | -6.040302000 | 1.852742000 | 4.175204000 |
| C | -5.699118000 | 1.352393000 | 2.923218000 |
| N | -6.615786000 | 1.016524000 | 1.993995000 |
| C | -7.947293000 | 1.176746000 | 2.297606000 |
| C | -8.345805000 | 1.675372000 | 3.539809000 |
| C | -7.388355000 | 2.017371000 | 4.490549000 |
| C | -8.899302000 | 0.790666000 | 1.235964000 |
| N | -8.288713000 | 0.337552000 | 0.117750000 |
| C | -8.979364000 | -0.067744000 | -0.971730000 |
| C | -10.376456000 | -0.026702000 | -0.966216000 |
| C | -11.028185000 | 0.437550000 | 0.178166000 |
| C | -10.294513000 | 0.851389000 | 1.291946000 |
| C | -8.106211000 | -0.522241000 | -2.073093000 |
| N | -6.754565000 | -0.466858000 | -1.827001000 |
| C | -5.907587000 | -0.869571000 | -2.794981000 |
| C | -6.340067000 | -1.337255000 | -4.031294000 |

| | | | |
|---|---|---|---|
| C | -7.709126000 | -1.395282000 | -4.288561000 |
| C | -8.595281000 | -0.983149000 | -3.297399000 |
| H | -4.664415000 | 1.208833000 | 2.636736000 |
| H | -5.257012000 | 2.105375000 | 4.881180000 |
| H | -7.691580000 | 2.404827000 | 5.457484000 |
| H | -9.399871000 | 1.795608000 | 3.760601000 |
| H | -10.805672000 | 1.210606000 | 2.177237000 |
| H | -12.111901000 | 0.477008000 | 0.202141000 |
| H | -10.950559000 | -0.346958000 | -1.827607000 |
| H | -9.664003000 | -1.018835000 | -3.472814000 |
| H | -8.082735000 | -1.754102000 | -5.241818000 |
| H | -5.609816000 | -1.647495000 | -4.770397000 |
| H | -4.853611000 | -0.807714000 | -2.553045000 |
| N | 2.885251000 | -2.265370000 | 0.825936000 |
| C | 2.661457000 | -3.554275000 | 1.218980000 |
| C | 3.883344000 | -4.173212000 | 1.380849000 |
| C | 4.842902000 | -3.203348000 | 1.044417000 |
| N | 4.252212000 | -2.057970000 | 0.698515000 |
| N | 3.023431000 | 1.969802000 | -1.143193000 |
| N | 4.371261000 | 1.640129000 | -1.086562000 |
| C | 5.043451000 | 2.726844000 | -1.471383000 |
| C | 4.158510000 | 3.778467000 | -1.763495000 |
| C | 2.896143000 | 3.272651000 | -1.533119000 |
| H | 1.666592000 | -3.950364000 | 1.323937000 |
| H | 4.058358000 | -5.191996000 | 1.690695000 |
| H | 5.920492000 | -3.277037000 | 1.023524000 |
| H | 1.936955000 | 3.757044000 | -1.588508000 |
| H | 4.407607000 | 4.776713000 | -2.089108000 |
| H | 6.122990000 | 2.702985000 | -1.508548000 |
| C | -4.568831000 | -2.081642000 | 0.817530000 |
| C | -4.230509000 | -3.423658000 | 1.042343000 |
| C | -5.228033000 | -4.367602000 | 1.314802000 |
| C | -6.565880000 | -3.941544000 | 1.341881000 |
| C | -6.840135000 | -2.618158000 | 1.056850000 |
| N | -5.891786000 | -1.698986000 | 0.784847000 |
| O | -5.008607000 | -5.673518000 | 1.548825000 |
| C | -3.662666000 | -6.186127000 | 1.526064000 |
| C | -3.691600000 | -7.687990000 | 1.844593000 |
| C | -2.234275000 | -8.187469000 | 1.799462000 |
| C | -4.276599000 | -7.918247000 | 3.250963000 |
| C | -4.530361000 | -8.436947000 | 0.791650000 |
| C | -4.407202000 | 2.443741000 | -0.749710000 |
| N | -5.762818000 | 2.179852000 | -0.651501000 |
| C | -6.631962000 | 3.177883000 | -0.874054000 |
| C | -6.262998000 | 4.483207000 | -1.173932000 |
| C | -4.894885000 | 4.782558000 | -1.212767000 |
| C | -3.974804000 | 3.746529000 | -0.990373000 |
| O | -4.371868000 | 6.000474000 | -1.439639000 |
| C | -5.255560000 | 7.118399000 | -1.656597000 |
| C | -4.418556000 | 8.388877000 | -1.863092000 |
| C | -5.408342000 | 9.548550000 | -2.089520000 |

| | | | |
|---|---|---|---|
| C | -3.512025000 | 8.230740000 | -3.098432000 |
| C | -3.565863000 | 8.670415000 | -0.611388000 |
| H | -3.199556000 | -3.731221000 | 0.965165000 |
| H | -7.362740000 | -4.644538000 | 1.554411000 |
| H | -7.864479000 | -2.263915000 | 1.033073000 |
| H | -3.062157000 | -5.646442000 | 2.269826000 |
| H | -3.231184000 | -6.011990000 | 0.531925000 |
| H | -1.789676000 | -8.037808000 | 0.808020000 |
| H | -2.195899000 | -9.259047000 | 2.025015000 |
| H | -1.609043000 | -7.667558000 | 2.535538000 |
| H | -3.681702000 | -7.402753000 | 4.015223000 |
| H | -4.279549000 | -8.987429000 | 3.492173000 |
| H | -5.306129000 | -7.552126000 | 3.317246000 |
| H | -4.125770000 | -8.285655000 | -0.216833000 |
| H | -5.570274000 | -8.095050000 | 0.795338000 |
| H | -4.526049000 | -9.513581000 | 0.997309000 |
| H | -7.681823000 | 2.917711000 | -0.796874000 |
| H | -7.030348000 | 5.227242000 | -1.341950000 |
| H | -2.924111000 | 3.999612000 | -0.977498000 |
| H | -5.912974000 | 7.227281000 | -0.784570000 |
| H | -5.874197000 | 6.917023000 | -2.540548000 |
| H | -6.035058000 | 9.374284000 | -2.972649000 |
| H | -4.862410000 | 10.485638000 | -2.246433000 |
| H | -6.069215000 | 9.686950000 | -1.225125000 |
| H | -4.104715000 | 8.040369000 | -4.001738000 |
| H | -2.809791000 | 7.400528000 | -2.971638000 |
| H | -2.930975000 | 9.145227000 | -3.264311000 |
| H | -4.197143000 | 8.792819000 | 0.277466000 |
| H | -2.988814000 | 9.592897000 | -0.743199000 |
| H | -2.862251000 | 7.854242000 | -0.418352000 |

Cartesian coordinates of DFT-optimized structure of **16ox** complex by B3LYP/Def2-TZVP; 6-31G(d,p)/$CH_3CN$, charge = 3, multiplicity = 2

| | | | |
|---|---|---|---|
| Os | 5.590435000 | -0.947881000 | 0.037260000 |
| N | 5.152323000 | 1.098247000 | -0.262039000 |
| N | 6.070032000 | -1.244338000 | -1.992007000 |
| N | 7.690794000 | -0.976550000 | 0.018949000 |
| N | 6.112967000 | -0.582314000 | 2.043291000 |
| N | -5.067875000 | 0.802771000 | -0.248977000 |
| C | 5.168446000 | -1.358490000 | -2.992096000 |
| H | 4.128014000 | -1.277569000 | -2.705674000 |
| C | 5.541133000 | -1.570788000 | -4.313229000 |
| H | 4.773141000 | -1.656669000 | -5.073330000 |
| C | 6.895648000 | -1.666735000 | -4.628216000 |
| H | 7.219754000 | -1.830419000 | -5.650192000 |
| C | 7.832387000 | -1.546580000 | -3.605863000 |
| H | 8.891088000 | -1.614566000 | -3.824687000 |
| C | 7.409251000 | -1.336342000 | -2.293550000 |
| C | 8.330599000 | -1.193994000 | -1.152854000 |

| | | | |
|---|---|---|---|
| C | 9.724999000 | -1.262132000 | -1.183682000 |
| H | 10.255655000 | -1.435700000 | -2.111904000 |
| C | 10.430135000 | -1.102675000 | 0.010182000 |
| C | 9.750371000 | -0.877006000 | 1.207910000 |
| H | 10.301095000 | -0.751708000 | 2.132116000 |
| C | 8.355259000 | -0.816452000 | 1.186190000 |
| C | 7.458512000 | -0.583089000 | 2.331407000 |
| C | 7.909721000 | -0.363419000 | 3.632864000 |
| H | 8.972813000 | -0.365339000 | 3.840743000 |
| C | 6.995425000 | -0.139831000 | 4.658055000 |
| H | 7.341472000 | 0.030217000 | 5.671761000 |
| C | 5.634691000 | -0.136019000 | 4.355754000 |
| H | 4.883333000 | 0.035878000 | 5.117886000 |
| C | 5.233507000 | -0.357599000 | 3.044553000 |
| H | 4.186912000 | -0.362992000 | 2.769366000 |
| C | 6.051372000 | 2.103561000 | -0.330303000 |
| H | 7.093056000 | 1.807125000 | -0.361110000 |
| C | 5.703405000 | 3.438610000 | -0.352565000 |
| H | 6.461742000 | 4.210242000 | -0.409762000 |
| C | 4.344088000 | 3.779639000 | -0.264884000 |
| C | 3.398099000 | 2.746722000 | -0.200556000 |
| H | 2.351923000 | 2.977424000 | -0.080731000 |
| C | 3.804913000 | 1.409344000 | -0.240872000 |
| C | -2.693138000 | -2.335655000 | 0.283656000 |
| C | 2.909684000 | 0.234935000 | -0.167319000 |
| C | -3.440453000 | -1.165453000 | 0.059048000 |
| C | 3.624880000 | -0.965605000 | 0.061190000 |
| C | -2.784525000 | 0.065026000 | -0.157064000 |
| C | 2.953901000 | -2.182267000 | 0.297762000 |
| C | -3.737081000 | 1.190917000 | -0.223637000 |
| C | -3.416767000 | 2.545914000 | -0.170621000 |
| H | -2.394248000 | 2.875174000 | -0.053792000 |
| C | -4.417657000 | 3.528000000 | -0.224051000 |
| C | -5.753872000 | 3.117385000 | -0.315223000 |
| H | -6.578932000 | 3.815344000 | -0.366378000 |
| C | -6.014350000 | 1.752857000 | -0.305922000 |
| H | -7.038946000 | 1.398963000 | -0.334753000 |
| C | -1.372326000 | 0.092552000 | -0.294268000 |
| C | 1.560651000 | -2.226010000 | 0.449777000 |
| C | -0.614818000 | -1.080168000 | 0.074770000 |
| C | 0.814621000 | -1.039739000 | 0.074493000 |
| C | -1.298181000 | -2.304326000 | 0.439349000 |
| C | 1.501441000 | 0.178306000 | -0.305200000 |
| C | -0.519088000 | -3.378859000 | 0.980020000 |
| C | 0.711913000 | 1.232808000 | -0.865549000 |
| H | -1.016967000 | -4.214360000 | 1.455339000 |
| H | 1.203971000 | 2.053880000 | -1.370649000 |
| C | 0.839774000 | -3.342839000 | 0.985600000 |
| C | -0.649358000 | 1.191345000 | -0.859088000 |
| H | 1.379143000 | -4.148001000 | 1.467626000 |
| H | -1.194566000 | 1.985053000 | -1.353681000 |

| | | | |
|---|---|---|---|
| O | 4.052776000 | 5.087858000 | -0.242470000 |
| O | -3.997471000 | 4.804453000 | -0.173615000 |
| C | -4.967837000 | 5.869393000 | -0.219415000 |
| C | -4.238688000 | 7.218130000 | -0.137127000 |
| C | -5.317613000 | 8.317640000 | -0.189270000 |
| C | -3.278943000 | 7.375188000 | -1.331748000 |
| C | -3.458223000 | 7.318663000 | 1.187251000 |
| C | 2.677707000 | 5.516657000 | -0.153870000 |
| C | 2.627393000 | 7.050546000 | -0.191215000 |
| C | 1.143812000 | 7.454801000 | -0.084072000 |
| C | 3.412777000 | 7.636331000 | 0.997507000 |
| C | 3.212039000 | 7.565861000 | -1.520030000 |
| H | -5.663219000 | 5.754844000 | 0.621933000 |
| H | -5.534211000 | 5.794700000 | -1.156550000 |
| H | -6.017559000 | 8.233719000 | 0.651027000 |
| H | -5.895776000 | 8.268222000 | -1.120030000 |
| H | -4.850511000 | 9.307554000 | -0.137793000 |
| H | -3.820230000 | 7.316749000 | -2.284148000 |
| H | -2.510813000 | 6.594967000 | -1.329096000 |
| H | -2.775538000 | 8.347945000 | -1.290455000 |
| H | -4.127951000 | 7.214141000 | 2.049864000 |
| H | -2.961917000 | 8.292933000 | 1.263640000 |
| H | -2.691240000 | 6.540494000 | 1.256717000 |
| H | 2.118709000 | 5.092065000 | -0.997263000 |
| H | 2.248195000 | 5.138624000 | 0.782386000 |
| H | 1.048410000 | 8.546005000 | -0.110477000 |
| H | 0.698873000 | 7.102269000 | 0.854390000 |
| H | 0.555239000 | 7.046909000 | -0.914929000 |
| H | 3.006374000 | 7.282388000 | 1.953028000 |
| H | 3.351325000 | 8.730632000 | 0.991021000 |
| H | 4.470014000 | 7.355878000 | 0.952726000 |
| H | 2.653788000 | 7.169390000 | -2.377228000 |
| H | 4.261017000 | 7.273486000 | -1.632344000 |
| H | 3.157826000 | 8.659744000 | -1.560676000 |
| H | 11.513362000 | -1.153304000 | 0.006871000 |
| N | 3.849966000 | -3.286967000 | 0.387721000 |
| C | 3.686528000 | -4.641673000 | 0.369409000 |
| C | 4.937125000 | -5.226522000 | 0.368157000 |
| C | 5.848584000 | -4.158681000 | 0.361096000 |
| N | 5.202257000 | -2.989414000 | 0.359987000 |
| N | -3.522149000 | -3.490237000 | 0.369559000 |
| C | -3.283660000 | -4.835180000 | 0.348857000 |
| C | -4.501781000 | -5.483993000 | 0.353604000 |
| C | -5.468479000 | -4.462004000 | 0.353301000 |
| N | -4.886364000 | -3.262086000 | 0.349356000 |
| H | 6.928798000 | -4.173132000 | 0.346776000 |
| H | 5.159561000 | -6.282462000 | 0.363858000 |
| H | 2.710857000 | -5.093742000 | 0.326265000 |
| H | -2.285634000 | -5.234952000 | 0.299172000 |
| H | -4.668802000 | -6.550452000 | 0.347081000 |
| H | -6.546537000 | -4.535059000 | 0.342839000 |

| | | | |
|---|---|---|---|
| Ru | -5.398204000 | -1.258573000 | 0.036363000 |
| C | -5.236898000 | -1.917474000 | -4.319897000 |
| C | -4.884417000 | -1.690545000 | -2.993648000 |
| N | -5.789903000 | -1.600131000 | -2.000178000 |
| C | -7.121961000 | -1.737125000 | -2.311814000 |
| C | -7.532161000 | -1.965863000 | -3.627009000 |
| C | -6.585643000 | -2.058228000 | -4.643619000 |
| C | -8.063078000 | -1.624741000 | -1.178518000 |
| C | -9.454849000 | -1.744739000 | -1.224800000 |
| C | -10.178458000 | -1.610138000 | -0.038235000 |
| C | -9.520230000 | -1.361335000 | 1.167881000 |
| C | -8.127162000 | -1.249600000 | 1.160817000 |
| N | -7.446763000 | -1.382631000 | 0.000106000 |
| C | -7.248599000 | -0.994025000 | 2.321076000 |
| N | -5.900753000 | -0.950837000 | 2.053097000 |
| C | -5.049375000 | -0.717450000 | 3.070801000 |
| C | -5.473034000 | -0.520317000 | 4.380897000 |
| C | -6.838074000 | -0.564231000 | 4.660855000 |
| C | -7.729270000 | -0.802731000 | 3.618261000 |
| H | -4.461907000 | -1.981211000 | -5.075645000 |
| H | -3.848973000 | -1.574856000 | -2.697147000 |
| H | -8.586714000 | -2.070397000 | -3.853186000 |
| H | -6.897880000 | -2.235774000 | -5.667373000 |
| H | -9.970404000 | -1.939384000 | -2.157850000 |
| H | -11.259298000 | -1.700410000 | -0.053303000 |
| H | -10.086889000 | -1.259766000 | 2.085883000 |
| H | -3.998620000 | -0.691059000 | 2.808459000 |
| H | -4.739543000 | -0.336681000 | 5.158097000 |
| H | -7.205158000 | -0.415052000 | 5.670839000 |
| H | -8.795108000 | -0.838971000 | 3.810194000 |

Cartesian coordinates of DFT-optimized structure of **17ox** complex by B3LYP/Def2-TZVP; 6-31G(d,p)/$CH_3CN$, charge = 3, multiplicity = 2

| | | | |
|---|---|---|---|
| Os | 5.429809000 | 0.849766000 | -0.023629000 |
| N | 5.355301000 | -1.241659000 | 0.285695000 |
| N | 5.898977000 | 1.236641000 | 1.992332000 |
| N | 7.496816000 | 1.223704000 | -0.055327000 |
| N | 5.957914000 | 0.580011000 | -2.044151000 |
| C | 5.014818000 | 1.206668000 | 3.013975000 |
| H | 3.995328000 | 0.953961000 | 2.753109000 |
| C | 5.379320000 | 1.480890000 | 4.326026000 |
| H | 4.625790000 | 1.443249000 | 5.104318000 |
| C | 6.707402000 | 1.796010000 | 4.608936000 |
| H | 7.024653000 | 2.014101000 | 5.622888000 |
| C | 7.627073000 | 1.823324000 | 3.564761000 |
| H | 8.666228000 | 2.060267000 | 3.758117000 |
| C | 7.212913000 | 1.542466000 | 2.262977000 |
| C | 8.119944000 | 1.542512000 | 1.102271000 |
| C | 9.487103000 | 1.826928000 | 1.102932000 |

| | | | |
|---|---|---|---|
| H | 10.003942000 | 2.083322000 | 2.019713000 |
| C | 10.182755000 | 1.775615000 | -0.105938000 |
| C | 9.520577000 | 1.444452000 | -1.288934000 |
| H | 10.063900000 | 1.404838000 | -2.225207000 |
| C | 8.152767000 | 1.167667000 | -1.237015000 |
| C | 7.279175000 | 0.795112000 | -2.362666000 |
| C | 7.729932000 | 0.651431000 | -3.674861000 |
| H | 8.773495000 | 0.826515000 | -3.906401000 |
| C | 6.840807000 | 0.281741000 | -4.679698000 |
| H | 7.186448000 | 0.168735000 | -5.701480000 |
| C | 5.505547000 | 0.059621000 | -4.346589000 |
| H | 4.774350000 | -0.231689000 | -5.091890000 |
| C | 5.103456000 | 0.217307000 | -3.026548000 |
| H | 4.075486000 | 0.057572000 | -2.728392000 |
| C | 6.414779000 | -2.077893000 | 0.334164000 |
| H | 7.390579000 | -1.607128000 | 0.341759000 |
| C | 6.302191000 | -3.452779000 | 0.363433000 |
| H | 7.182903000 | -4.082523000 | 0.402652000 |
| C | 5.019708000 | -4.021844000 | 0.306708000 |
| C | 3.909595000 | -3.166361000 | 0.266451000 |
| H | 2.916815000 | -3.575186000 | 0.171705000 |
| C | 4.080931000 | -1.778804000 | 0.295774000 |
| C | -3.000321000 | 0.790801000 | -0.190218000 |
| C | 2.995790000 | -0.775486000 | 0.237417000 |
| C | -3.500119000 | -0.509472000 | 0.038507000 |
| C | 3.491799000 | 0.529571000 | -0.008759000 |
| C | -2.615362000 | -1.576360000 | 0.281688000 |
| C | 2.617880000 | 1.610194000 | -0.239565000 |
| C | -1.234225000 | -1.373458000 | 0.432304000 |
| C | 1.232959000 | 1.412041000 | -0.370066000 |
| C | -0.705682000 | -0.082768000 | 0.044958000 |
| C | 0.709461000 | 0.121739000 | 0.028521000 |
| C | -1.601597000 | 0.988496000 | -0.328284000 |
| C | 1.604106000 | -0.958092000 | 0.401293000 |
| C | -1.020310000 | 2.167116000 | -0.896886000 |
| C | 1.017447000 | -2.132638000 | 0.978022000 |
| H | -1.660475000 | 2.890819000 | -1.384276000 |
| H | 1.653774000 | -2.851983000 | 1.476508000 |
| C | 0.325230000 | 2.375043000 | -0.911819000 |
| C | -0.327619000 | -2.332562000 | 0.989104000 |
| H | 0.711002000 | 3.254758000 | -1.410817000 |
| H | -0.718401000 | -3.207807000 | 1.492371000 |
| O | 4.954799000 | -5.360632000 | 0.287307000 |
| C | 3.670205000 | -6.016512000 | 0.234889000 |
| C | 3.882824000 | -7.536741000 | 0.232601000 |
| C | 2.485613000 | -8.185669000 | 0.177107000 |
| C | 4.700067000 | -7.954580000 | -1.004546000 |
| C | 4.607262000 | -7.971669000 | 1.520558000 |
| H | 3.080289000 | -5.709943000 | 1.108169000 |
| H | 3.146564000 | -5.699635000 | -0.675720000 |
| H | 2.577553000 | -9.277482000 | 0.173065000 |

| | | | |
|---|---|---|---|
| H | 1.941987000 | -7.891838000 | -0.729133000 |
| H | 1.877665000 | -7.904162000 | 1.045582000 |
| H | 4.192839000 | -7.659638000 | -1.931526000 |
| H | 4.830845000 | -9.042539000 | -1.024591000 |
| H | 5.692917000 | -7.493385000 | -0.998734000 |
| H | 4.032505000 | -7.689213000 | 2.411223000 |
| H | 5.597549000 | -7.510911000 | 1.594692000 |
| H | 4.737173000 | -9.059825000 | 1.535463000 |
| H | 11.244767000 | 1.994050000 | -0.126113000 |
| N | 3.304200000 | 2.851440000 | -0.349159000 |
| C | 2.904921000 | 4.157514000 | -0.334162000 |
| C | 4.033188000 | 4.951350000 | -0.347004000 |
| C | 5.118650000 | 4.058977000 | -0.345377000 |
| N | 4.688015000 | 2.795229000 | -0.334387000 |
| N | -3.294985000 | -2.822581000 | 0.382015000 |
| N | -4.676650000 | -2.767100000 | 0.341342000 |
| C | -5.104982000 | -4.029346000 | 0.365522000 |
| C | -4.018510000 | -4.922948000 | 0.400272000 |
| C | -2.890705000 | -4.127990000 | 0.395356000 |
| C | -4.087791000 | 1.786846000 | -0.273222000 |
| C | -3.933178000 | 3.178994000 | -0.217240000 |
| C | -5.051283000 | 4.019204000 | -0.281006000 |
| C | -6.323600000 | 3.435349000 | -0.391930000 |
| C | -6.416879000 | 2.057400000 | -0.387988000 |
| N | -5.350926000 | 1.234170000 | -0.314571000 |
| O | -5.007502000 | 5.362397000 | -0.235634000 |
| C | -3.737027000 | 6.032080000 | -0.121869000 |
| C | -3.969530000 | 7.549629000 | -0.096104000 |
| C | -2.584838000 | 8.216327000 | 0.021666000 |
| C | -4.654588000 | 8.001868000 | -1.399631000 |
| C | -4.835713000 | 7.931043000 | 1.119383000 |
| H | -6.165649000 | -4.234997000 | 0.343722000 |
| H | -4.051783000 | -6.001787000 | 0.415727000 |
| H | -1.850062000 | -4.400959000 | 0.367282000 |
| H | -2.949271000 | 3.598996000 | -0.081897000 |
| H | -7.210311000 | 4.055518000 | -0.451015000 |
| H | -7.385890000 | 1.573484000 | -0.435934000 |
| H | -3.108791000 | 5.753124000 | -0.977901000 |
| H | -3.241284000 | 5.704084000 | 0.800769000 |
| H | -2.068628000 | 7.909812000 | 0.939644000 |
| H | -2.691736000 | 9.306590000 | 0.045681000 |
| H | -1.943671000 | 7.961618000 | -0.831053000 |
| H | -4.045052000 | 7.745669000 | -2.275050000 |
| H | -4.799279000 | 9.088364000 | -1.397291000 |
| H | -5.635009000 | 7.529485000 | -1.518178000 |
| H | -4.356425000 | 7.625299000 | 2.057744000 |
| H | -5.820292000 | 7.455088000 | 1.069826000 |
| H | -4.984039000 | 9.016396000 | 1.156074000 |
| Ru | -5.428468000 | -0.847494000 | -0.007686000 |
| C | -5.669042000 | -0.085596000 | 4.327076000 |
| C | -5.201692000 | -0.239858000 | 3.026134000 |

| | | | |
|---|---|---|---|
| N | -6.000732000 | -0.581998000 | 1.996758000 |
| C | -7.337830000 | -0.786119000 | 2.242933000 |
| C | -7.860652000 | -0.645900000 | 3.530311000 |
| C | -7.023384000 | -0.292752000 | 4.585056000 |
| C | -8.158419000 | -1.156875000 | 1.071588000 |
| N | -7.445965000 | -1.223770000 | -0.075299000 |
| C | -8.007151000 | -1.553885000 | -1.260036000 |
| C | -9.374281000 | -1.836633000 | -1.327950000 |
| C | -10.130850000 | -1.770876000 | -0.156145000 |
| C | -9.528851000 | -1.430353000 | 1.056925000 |
| C | -7.038137000 | -1.569600000 | -2.375244000 |
| N | -5.739121000 | -1.264017000 | -2.044368000 |
| C | -4.811410000 | -1.255601000 | -3.021300000 |
| C | -5.108380000 | -1.546411000 | -4.348693000 |
| C | -6.422629000 | -1.860642000 | -4.691338000 |
| C | -7.392006000 | -1.869986000 | -3.692266000 |
| H | -4.157112000 | -0.089990000 | 2.781420000 |
| H | -4.976794000 | 0.191040000 | 5.114507000 |
| H | -7.423292000 | -0.182336000 | 5.587516000 |
| H | -8.917033000 | -0.812024000 | 3.705359000 |
| H | -10.120229000 | -1.383004000 | 1.963597000 |
| H | -11.193344000 | -1.986992000 | -0.188160000 |
| H | -9.846265000 | -2.103054000 | -2.266265000 |
| H | -8.420998000 | -2.108400000 | -3.934086000 |
| H | -6.691347000 | -2.093113000 | -5.716464000 |
| H | -4.317852000 | -1.524240000 | -5.090574000 |
| H | -3.804311000 | -1.004750000 | -2.711004000 |
| H | 4.068071000 | 6.029910000 | -0.348247000 |
| H | 1.865062000 | 4.430521000 | -0.283692000 |
| H | 6.179469000 | 4.263959000 | -0.340360000 |
